# Sulfur‐Directed Construction of Vinyl Cyclopropanes from 1,3‐Dienes

**DOI:** 10.1002/anie.4468205

**Published:** 2026-04-20

**Authors:** Anna Keimer, Jonas Rettig, Tim‐Niclas Streit, Bob Barthel, Mélissa Calmels, Moritz Malischewski, Franz‐Lucas Haut

**Affiliations:** ^1^ Institute of Chemistry and Biochemistry Freie Universität Berlin Berlin Germany

**Keywords:** carbenes, copper catalysis, pericyclic reaction, vinyl cyclopropanes, ylides

## Abstract

The stereoselective cyclopropanation of 1,3‐dienes remains a long‐standing challenge in the preparation of vinyl cyclopropanes (VCPs) due to the intrinsic electronic and steric bias of the diene scaffold. We report a sulfur‐directed strategy that enables highly site‐ and diastereoselective cyclopropanation of *S*‐substituted 1,3‐dienes with diazo reagents under Cu‐catalyzed reaction conditions. This unprecedented approach overrides the innate reactivity of the 1,3‐diene through a thioether‐directed reaction mode, providing rapid access to a broad library of highly functionalized *S*‐VCPs obtained as single regio‐ and diastereomers. Preliminary mechanistic studies indicate a pericyclic cascade involving a 6π‐electrocyclization and a [2,3]‐sigmatropic rearrangement and postmodifications permit streamlined access to complex VCPs that remain inaccessible through conventional cyclopropanation techniques.

## Graphical abstract

**Sulfur takes control**. A pending thioether acts as a molecular compass, directing carbene addition to the 1,3‐diene scaffold with exceptional regio‑ and diastereocontrol to forge sulfur‐substituted vinyl cyclopropanes (*S*‐VCPs) — molecular architectures inaccessible via conventional cyclopropanation strategies.

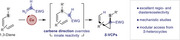

## 1 Introduction

**FIGURE 1:**
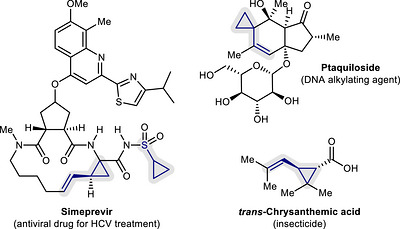
Selected examples of biologically relevant vinyl cyclopropanes.

Cyclopropanes are the smallest, all‐carbon ring system, and their controllable synthesis has sparked the interest of the chemistry community for a long time [1]. The presence of the cyclopropane motif in various natural products, drugs, and agrochemicals underscores the significance of developing efficient cyclopropanation reactions to advance pharmaceutical and agrochemical research programs (Figure 1) [2, 3, 4, 5]. However, achieving complete stereocontrol of all six positions of the three‐membered carbocycle still displays a major challenge in the field [6].

A particular fascinating subclass is that of vinyl cyclopropanes (VCPs), which have become remarkably versatile synthetic intermediates in organic synthesis [7]. Due to their ambivalent reactivity – they undergo rearrangement reactions or ring‐opening with nucleophiles, electrophiles and radical species – the construction of polysubstituted VCPs and installation of functional groups along the strained ring‐system is challenging [8, 9, 10, 11, 12]. Significant advances have been made in the field of transition metal‐mediated cross‐coupling methodologies, facilitating the attachment of vinyl residues to cyclopropanes or cyclopropenes [13, 14]. However, a straightforward approach is represented by the direct cyclopropanation of 1,3‐dienes. Achieving chemoselectivity in this transformation requires a highly site‐selective and stereospecific process, typically involving the generation of a transient carbene species as coupling partner. Diazo compounds have been identified as adaptable and user‐friendly carbene precursors in synthetic applications allowing for the efficient construction of cyclopropanes and related carbocyclic systems [15, 16].

**SCHEME 1:**
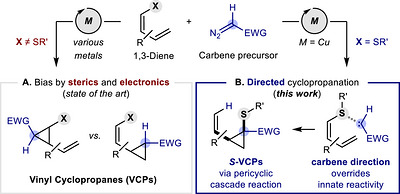
Steric and electronic bias in the cyclopropanation of 1,3‐dienes (A), *state of the art* and the sulfur‐directed approach presented in this work (B).

In this context, the application of transition metal catalysis predominantly based on Cu, Rh, Pd, and Ni has been found as a powerful strategy to assemble VCPs from 1,3‐diene precursors and diazo reagents with high stereocontrol (Scheme 1A, *state of the art*) [17, 18, 19, 20, 21, 22, 23, 24]. More recently, the merger of organo‐ and photocatalysis has been demonstrated for efficient VCP formation [25]. Despite these advances in the synthesis of VCPs, the chemoselectivity is usually dictated by the innate electronic and steric properties of the 1,3‐diene scaffold. Moreover, the substrate scope remains limited to simple precursors omitting the incorporation of heteroatoms and functional handles at the backbone for further derivatization. The underlying factors for this could be attributed to the restricted synthetic access to structurally divers 1,3‐diene precursors as well as their capability to effectively participate in the envisioned ring formation step. Presently, addressing these limitations is essential to expand the accessible chemical space in VCP synthesis. To tackle these challenges, we herein demonstrate the feasibility of 1,3‐dienes bearing thioethers (X = SR’) – readily obtained from the ring‐opening of 2,5‐dihydrothiophenes (DHTs) [26, 27, 28] – for the site‐ and diastereoselective synthesis of *S*‐substituted VCPs (Scheme 1B, *this work*). In contrast to previous work, the pending thioether directs the transient carbene species to a specific olefinic site, thereby overcoming substrate‐dependent steric and electronic bias. Eventually, highly substituted *S*‐VCPs are assembled through an unprecedented pericyclic reaction cascade cumulating in cyclopropane formation accompanied by migration of the thioether moiety (for mechanistic details, see Scheme 4). The latter could be employed as a useful synthetic handle for further diversification of the cyclopropane [29]. Notably, the reaction could be conducted utilizing a commercial and inexpensive Cu(I)‐catalyst, and the products are obtained as single regio‐ and diastereomers in all cases.

## 2 Results and Discussion

At the outset of our investigation, it was unclear whether the sterically encumbered 1,3‐diene **1a** would undergo site‐selective, *S*‐directed cyclopropanation with ethyl diazoacetate (**2a**) under Cu‐catalyzed conditions. The result of the reaction was also erratic in terms of stereoselectivity and regioselectivity, as well as the potential overreaction of the vinyl unit of the cyclopropane or the pending thioether. To our delight, commercially available tetrakis(acetonitrile)copper(I) hexafluorophosphate ([Cu(MeCN)_4_]PF_6_) catalyzed the envisioned transformation in chloroform (0.1 M) at room temperature (RT) under Ar atmosphere delivering VCP **3a** as a single diastereomeric product in 50% yield (Table 1, entry 1). Further screening revealed that [Cu(MeCN)_4_]OTf and (CuOTf)_2_·PhH complex also promoted product formation albeit in lower yields (up to 38%, entries 2–3). Other Cu(I)‐sources such as CuBr·DMS complex, CuCN or CuCl (entry 4) proved ineffective for the desired cyclization cascade. Notably, ethyl acetate and hexafluoro‐2‐propanol (HFIP) were competent solvents for the reaction (21%–49%, entries 5–6), but **3a** was formed in only 6% yield when employing acetonitrile as solvent (entry 7). Product formation could be further enhanced to 84% yield upon reduced catalyst loading (2.5 mol%) at lower concentration (0.05 M) inhibiting undesired dimerization of **2a** (entries 8–11) [30]. Eventually, **3a** was consistently isolated in 77% yield when utilizing three equivalents of **2a** and running the reaction overnight (15 h, entry 11). In addition, the necessity of the Cu(I)‐catalyst was confirmed as no VCP was formed in the absence of [Cu(MeCN)_4_]PF_6_ (entry 12).

**TABLE 1:** Optimization studies^a^.

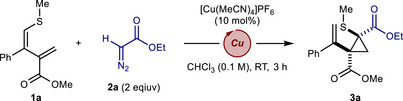		
Entry	Deviation from conditions above	3a (%)^b^
1	None	50
2	[Cu(MeCN)_4_]OTf (10 mol%) as catalyst	22
3	(CuOTf)_2_·PhH (10 mol%) as catalyst	38
4	CuBr·Me_2_S, CuCN or CuCl (10 mol%) as catalyst	0
5	EtOAc as solvent	49
6	HFIP as solvent	21
7	MeCN as solvent	6
8	[Cu(MeCN)_4_]PF_6_ (2.5 mol%)	68
9	[Cu(MeCN)_4_]PF_6_ (2.5 mol%), **2a** (3 equiv)	79
10	[Cu(MeCN)_4_]PF_6_ (2.5 mol%), **2a** (3 equiv), 15 h	84
**11**	**[Cu(MeCN)_4_]PF_6_ (2.5 mol%), 2a (3 equiv), 15 h**	**77** ^c^
12	no [Cu(MeCN)_4_]PF_6_	0

^a^ Reaction conditions: **1a** (0.05 mmol, limiting reagent), **2a** (0.1 mmol, 2 equiv), [Cu(MeCN)_4_]PF_6_ (10 mol%) in CHCl_3_ (0.1 M) at RT (ca. 21°C) under Ar atmosphere for 3 h.

^b^ ^1^H NMR yield using CH_2_Br_2_ as internal standard.

^c^ Isolated yield, 0.2 mmol scale.

**SCHEME 2:**
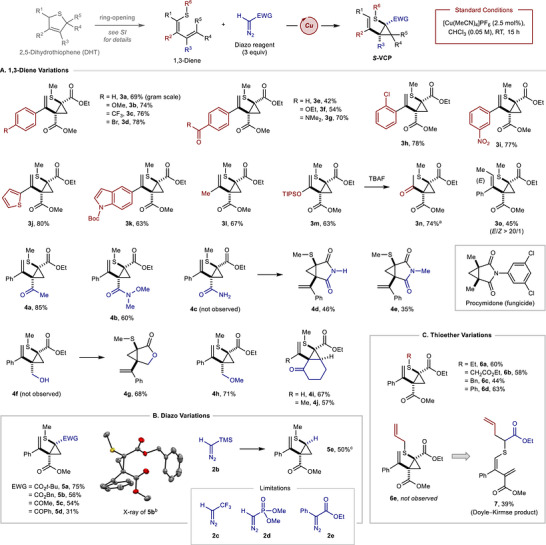
Synthetic scope. Reaction conditions: 1,3‐diene (0.2 mmol, limiting reagent), diazo reagent (0.6 mmol, 3 equiv), [Cu(MeCN)_4_]PF_6_ (5.0 µmol, 2.5 mol%) in CHCl_3_ (0.05 M) at RT (ca. 21°C) under Ar atmosphere for 15 h. All products were obtained as single diastereomers (*dr* >20:1). ^a^ TBAF (1.1 equiv), THF (0.1 M), RT, 10 min. ^b^ *H*‐atoms are omitted for clarity. ^c^ 1,2‐Dichloroethane as solvent, 85°C.

With the optimized reaction conditions in hand, we set out to investigate the scalability and generality of our developed cyclopropanation method. As illustrated in Scheme 2A, the operational simplicity of the method allowed us to prepare more than one gram of VCP **3a** (69%, 5 mmol scale) in a single run. Substrates bearing electron‐rich (**3b**) and electron‐poor arenes (**3c**, **3e**–**3g**) effectively participated in the reaction accessing the corresponding products in synthetically useful yields (42–76%). Our cyclopropanation protocol also allowed the incorporation of halogens (**3c**, **3d,** **3h**), nitro groups (**3i**), and heterocycles (**3j**–**3k**) in up to 77% yield. Notably, the reaction could be expanded beyond (hetero)styryl substituents (R^2^) enabling the efficient construction of the alkyl‐substituted VCP **3l** (67%). Following our protocol, **3m** could be synthesized without detectable cyclopropanation of the electron‐rich silyl enol ether in 63% yield. Subsequent rapid hydrolysis of **3m** with tetra‐*n*‐butylammonium fluoride (TBAF) revealed acetylated cyclopropane **3n** in 74% yield. When subjecting tetrasubstituted vinyl sulfides (R^1^ = Me) to the reaction conditions, VCP **3o** could be obtained as a single diastereomer (45%, *E*/*Z* > 20/1). Variation of R^3^ revealed that ketones (**4a**), and Weinreb amides (**4b**) were also tolerated under the reaction conditions in up to 85% yield. In the case of a primary amide, intramolecular cyclization of **4c** with the ester function occurred concurrently, delivering the cyclopropane‐fused succinimide **4d** in 46% yield. The reaction was also found to applicable to secondary amides (**4e**, 35%) granting access to bicyclic architectures, which present important structural motifs in medicinal chemistry and agrochemicals as featured in fungicide procymidone [2, 31]. A similar cyclization could be observed when employing primary alcohols (**4f**) to the reaction, which exclusively led to the formation of lactone **4g** in 68% yield. Notably, protection as methyl ether prevented ring‐closure enabling access to VCP **4h** (71%), thereby highlighting that an electron‐withdrawing groups (EWG) is not necessary for the reaction to occur. The modularity of our method was further demonstrated by the efficient assembly of cyclohexanone‐fused VCPs **4i**–**4j** allowing full stereocontrol over the substitution pattern of the cyclopropane (up to 67%). Having established the synthetic scope of various 1,3‐dienes for VCP synthesis, different diazo reagents were applied to our developed protocol (Scheme 2B). Fortunately, this strategy enabled incorporation of EWGs such as *tert*‐butyl and benzyl esters (**5a**–**5b**) and ketones (**5c**–**5d**) at the cyclopropane backbone in excellent diastereoselectivity (*dr* > 20/1, up to 75%). Notably, the relative configuration of the cyclopropane substitution pattern was ultimately confirmed through X‐ray analysis of **5b** (CCDC‐2496555) [32]. To our delight, exposure of **1a** to trimethylsilyldiazomethane (**2b**) effectively formed trisubstituted VCP **5e** in 50% yield thereby realizing the formal incorporation of diazomethane (CH_2_N_2_) without the necessity of an EWG attached to the diazo moiety. The formation of the product was observed only at elevated temperatures (85°C) when 1,2‐dichloroethane was utilized as a solvent. However, the application of **2c**, Seyferth–Gilbert reagent (**2d**) or **2e** did not lead to significant VCP production [33]. Finally, we turned our attention to the thioether moiety of the 1,3‐diene component (Scheme 2C). Alkyl (**6a**–**6b**), benzyl (**6c**), and phenyl residues (**6d**) are well tolerated leading to the desired products in 44%–63% yield. In the case of an allyl thioether, the corresponding VCP **6e** was not observed but Doyle–Kirmse product **7** was formed via [2,3]‐sigmatropic rearrangement [34, 35].

**SCHEME 3:**
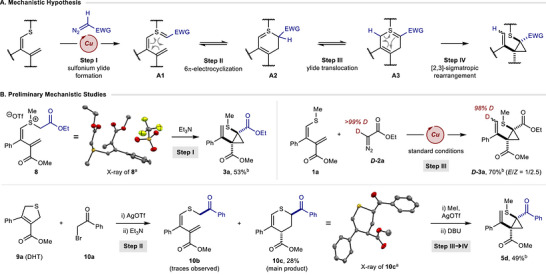
Mechanistic hypothesis supported by preliminary experimental investigations. For experimental details, see Supporting Information Section S6.^a^ *H*‐atoms are omitted for clarity. ^b^ ^1^H NMR yield using CH_2_Br_2_ as internal standard.

Sparked by the exclusive formation of **7**, we sought to gain insight into the mechanism of the developed cyclopropanation reaction. As illustrated in Scheme 3A, the reaction cascade might be initiated by Cu‐mediated carbene transfer leading to sulfonium ylide intermediate **A1** [36, 37]. From there, a 6π‐electrocyclization could take place forming a second sulfonium ylide species **A2** upon ring‐closure [38]. Subsequent translocation of the *S*‐ylide intermediate to **A3** would then set the stage for cyclopropane formation via [2,3]‐sigmatropic rearrangement [39, 40, 41]. To underpin our mechanistic hypothesis, preliminary experimental studies were conducted to investigate the elementary steps of the reaction sequence (Scheme 3B). The initial sulfonium ylide formation (Step I) was tested through an alternative pathway relying on the deprotonation of a sulfonium salt. For this purpose, sulfonium triflate **8** was synthesized and rapidly underwent VCP formation (**3a**, 53%) upon treatment with Et_3_N as a base. These results are also in line with the formation of Doyle–Kirmse product **7** presumably arising from an analogous sulfonium ylide intermediate [42, 43, 44]. To probe the protonation/deprotonation process that is essential for ylide translocation (**A2**→**A3**, Step III) [45, 46], deuterated diazo reagent ***D*‐2a** was subjected to the reaction conditions. To our delight, almost quantitative *D*‐incorporation (***D*‐3a**, 98% *D*) could be observed in agreement with our mechanistic hypothesis. Furthermore, the attempted synthesis of 1,3‐diene **10b** from DHT (**9a**) and bromide **10a** resulted in the formation of 3,4‐dihydro‐2*H*‐thiopyrane **10c** as the predominant product, with only traces of **10b** being observed. This finding underscores the rapid intramolecular C–C bond formation upon ring‐opening under basic reaction conditions (**10b**→**10c**, Step II). Moreover, the competent ring‐contraction of **10b** to VCP **5d** (49%) through *S*‐methylation and base‐promoted cyclopropane formation utilizing 1,8‐diazabicyclo[5.4.0]undec‐7‐ene (DBU) further emphasizes six‐membered *S*‐heterocycles (**A2**, **A3**) as transient intermediates within the proposed reaction pathway [39, 40, 41]. The high degree of preorganization within the cyclic intermediates could also rationalize the exclusive formation of a single diastereomer, as the vinyl and thioether substituents both originate from the thiopyrane species thus adopting a *cis*‑orientation within the cyclopropane scaffold.

**SCHEME 4:**
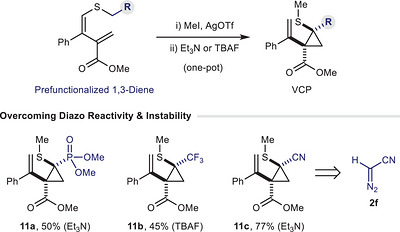
Expanding the synthetic scope through base‐promoted cyclopropane formation. For experimental details, see Supporting Information Section S7.

Encouraged by the efficiency of the base‐promoted cyclopropanation (**8**→**3a**, Scheme 3B), we elaborated this process as an alternative synthetic strategy to access structurally complex VCPs from prefunctionalized 1,3‐diene precursors (Scheme 4). For this purpose, we established a one‐pot procedure consisting of (i) sulfonium salt generation through *S*‐methylation and (ii) VCP formation upon subsequent addition of an external base. This strategy allowed us to synthesize VCPs bearing phosphonates or CF_3_‐groups (**11a**–**11b**, up to 50% yield), substrates out of reach of the Cu‐catalyzed protocol when employing the corresponding diazo reagents (**2c**–**2d**). Noteworthy, judicious choice of TBAF as both nucleophilic fluoride source as well as base proved critical for the formation of **11b**, as it prevents decomposition pathways based on β‐fluoride elimination [47]. In contrast, insufficient product formation was observed in the presence of Cs_2_CO_3_ or amine bases. Furthermore, nitrile **11c** was obtained in 77% yield, thus circumventing the necessity of thermally unstable and explosive diazoacetonitrile (**2f**) [48].

**SCHEME 5:**
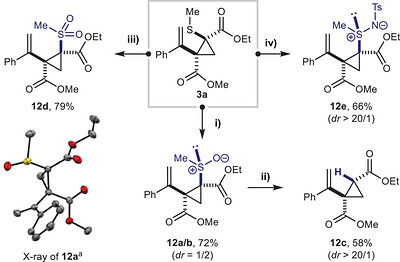
VCP diversification via *S*‐modifications. For experimental details, see Supporting Information Section S8. Reaction conditions: i) *m*‐CPBA (1.1 equiv), CH_2_Cl_2_ (0.15 M), −78°C to RT, 7 h. ii) PhSiH_3_ (5.0 equiv), KOH (1.0 equiv), THF (0.08 M), RT, 2 h. iii) *m*‐CPBA (2.4 equiv), CH_2_Cl_2_ (0.15 M), −78°C to RT, 7 h. iv) Chloramine‐T trihydrate (1.2 equiv), MeCN/HFIP (9/1, 0.07 M), RT, 30 min. ^a^ *H*‐atoms are omitted for clarity.

The versatility of our method in expanding the synthetic space in VCP chemistry could was further demonstrated by chemoselective functionalization of the thioether moiety as illustrated in Scheme 5. Sulfoxides **12a** and **12b** were obtained as separable mixture of two diastereomers (*dr* = 1/2, 72% yield) when utilizing *meta*‐chloro‐peroxybenzoic acid (*m*‐CPBA, 1.1 equivalents). Subsequent desulfinylation employing an excess of PhSiH_3_ and one equivalent of KOH furnished trisubstituted VCP **12c** with complete stereoretention (58% yield) [49]. Notably, the stereoselective synthesis of **12c** remains elusive when using 1,3‐dienes lacking the thioether moiety, further underscoring the necessity of the sulfur‑directed strategy [50]. A simple variation to 2.4 equivalents *m*‐CPBA resulted in sulfone **12d** from **3a** in 79% yield, without detectable epoxidation of the styrene motif. In addition, exposure of **3a** to chloramine‐T trihydrate (1.2 equivalents) in a solvent mixture of MeCN and HFIP (9/1) effectively generated *N*‐tosyl sulfilimine **12e** as a single diastereomer (66% yield).

## 3 Conclusion

In summary, we have demonstrated that thioether residues can direct a highly regio‐ and diastereoselective cyclopropanation of 1,3‐dienes. This stands in sharp contrast to common methods relying on substrate‐specific, innate reactivity based on the electronic and steric properties of the 1,3‐diene precursors. The developed reaction proceeds under mild, Cu‐catalyzed reaction conditions and preliminary mechanistic studies are in full agreement with a mechanism that involves initial sulfonium ylide formation followed by a pericyclic cascade reaction featuring a 6π‐electrocyclization and a [2,3]‐sigmatropic rearrangement. Our methodology provides a modular approach to highly substituted VCPs bearing *S*‐substituents, which are out of reach using conventional cyclopropanation strategies, thereby expanding the synthetic space in cyclopropane chemistry. Further studies on related carbocyclic frameworks are currently underway in our laboratory.

## Conflicts of Interest

The authors declare no conflicts of interest.

## Supporting information



The authors have cited additional references within the Supporting Information [51, 52, 53, 54, 55, 56, 57, 58, 59].

**Supporting File**: anie72273‐sup‐0002‐SuppMat.Pdf.

## Data Availability

The data that support the findings of this study are available from the corresponding author upon reasonable request.
