# Supplementary material for: Sulfur‐Directed Construction of Vinyl Cyclopropanes from 1,3‐Dienes
Source: Angew Chem Int Ed Engl. 2026 Apr 20;65(25):e4468205. doi: 10.1002/anie.4468205 (PMC13266940; doi:10.1002/anie.4468205)

Supporting Information

Sulfur-Directed Construction of Vinyl Cyclopropanes from 1,3-Dienes

A. Keimer, J. Rettig, T.-N. Streit, B. Barthel, M. Calmels, M. Malischewski, F.-L. Haut*

E-mail: f.haut@fu-berlin.de

1. General Experimental Information **2**

2. Optimization Studies **4**

3. General Procedures **7**

4. Synthesis of Starting Materials and Reagents **9**

4.1. Synthesis of 2,5-Dihydrothiophenes (DHTs) 10

4.2. Synthesis of *S*-Substituted 1,3-Dienes 15

5. Synthesis of Vinyl Cyclopropanes **22**

5.1. Substrate Scope 22

5.2. Limitations 39

6. Mechanistic Studies **40**

6.1. Doyle−Kirmse Reaction 40

6.2. Base-promoted Cyclopropanation from Sulfonium Salt 41

6.3. Isotope Labeling Studies 42

6.4. Synthesis and Ring-Contraction of Thiopyrane Species 44

6.5. Control Experiment: Non-Directed Cyclopropanation 46

7. Alternative Vinyl Cyclopropane Formation **48**

8. Postmodifications **50**

9. References **54**

10. X-Ray Crystallographic Data **56**

11. NMR Spectra **62**

13. HRMS Spectra **137**

1. General Experimental Information

All reactions were performed in oven-dry glassware fitted with rubber septa under a positive pressure of argon, unless stated otherwise. Air- and moisture-sensitive liquids were transferred via syringe through rubber septa. Solids were added under argon counter flow or were dissolved in appropriate solvents. Room temperature (RT) refers to temperatures between 21 °C and 25 °C. Reactions at low temperatures were carried out in a Dewar vessel filled with a cooling agent: acetone/dry ice (−78 °C) or distilled water/ice (0 °C). Reaction temperatures above RT were conducted in preheated aluminum blocks, sand or oil baths filled with silicone oil (Carl Roth, M 350). The reactions were magnetically stirred and monitored by ^{1}H NMR spectroscopy or analytical thin-layer chromatography (TLC) using aluminum plates precoated with silica gel [0.20 mm, 60 Å pore size, Merck, impregnated with a fluorescent indicator (254 nm)]. TLC plates were visualized by exposure to ultraviolet light (UV), were stained by submersion in ceric ammonium molybdate solution (CAM) or aqueous potassium permanganate solution ($KMnO_4$) and were developed by heating with a heat gun. The TLC plates intended for the monitoring of acid-labile compounds were deactivated using a mixture of diethyl ether and triethylamine (20% Et_3N in Et_2O) followed by drying under air prior to use.

Solvents, reagents and starting materials were used as received unless otherwise stated. Water is de-ionised, and solvents were anhydrous unless stated otherwise. Anhydrous toluene (PhMe), dichloromethane (CH_2Cl_2) and tetrahydrofuran (THF) were provided by purification with a MBraun SPS-800 solvent system using solvents of HPLC grade purchased from Thermo Fisher Scientific and Carl Roth. CH_2Cl_2, $CHCl_3$ and THF were additionally dried using activated 4 Å molecular sieves. Methyl 4-oxotetrahydrothiophene-3-carboxylate (CAS: 2689-68-1, F042131), tetrakis(triphenylphosphin)palladium(0) ($Pd(PPh_3)_4$, CAS: 14221-01-3, F034279), trimethyloxonium tetrafluoroborate (Me_3OBF_4, CAS: 420-37-1, PC7865) and *tert*-butyl 2-diazoacetate (CAS: 35059-50-8, F448618) were purchased from Fluorochem. Tetrakis(acetonitrile)copper(I) hexafluorophosphate ($[Cu(MeCN)_4]PF_6$, CAS: 64443-05-6, BD153541 98%) was purchased from BLD pharm. Ethyl diazo acetate (CAS: 623-73-4, E22201) and benzyl diazo acetate (CAS: 52267-51-3, 752185), triflic anhydride (Tf_2O, CAS: 358-23-6, 91737), *N*,*N*-diisopropylethylamine (DIPEA, CAS: 7087-68-5, 387649) and triethylamine (Et_3N, CAS: 121-44-8, 90340) were purchased from Sigma-Aldrich. Phenyl boronic acid (CAS: 98-80-6, AB113849) was purchased from ABCR. TMS diazomethane (CAS: 18107-18-1, product no.: 10413962), acetone (CAS: 67-64-1, 650501, extra dry) and chloroform ($CHCl_3$, CAS: 67-66-3, 10353582, extra dry) were purchased from Thermo Fisher Scientific.

Flash-column chromatography was carried out using silica gel (Macherey-Nagel, pore size 60 Å, 0.04–0.063 mm). Solvents such as ethyl acetate (EtOAc), cyclohexane (CyH), petroleum ether (PE), *n*-pentane, diethyl ether (Et_2O), CH_2Cl_2, isopropanol (*i*-PrOH) and methanol (MeOH) were purchased on technical grade and distilled prior to use. *Tert*-butyl methyl ether (MTBE) was purchased on HPLC

grade from Sigma-Aldrich and was used without further purification. Flash-column chromatography was performed adding Et_3N (1−2%) to the solvent mixtures to facilitate the purification of acid-sensitive compounds. The necessity of silica deactivation is indicated in the experiment, if applicable.

NMR Spectroscopy: ^{1}H and ^{13}C NMR spectra were acquired at different field strengths, as indicated, using Bruker Avance 500 MHz or Bruker Avance 700 MHz spectrometers. ^{19}F and ^{31}P NMR spectra were acquired using Jeol ECX 400 MHz. All NMR spectra were recorded at RT (20 °C). Chemical shifts (δ) are given in parts per million (ppm) and referenced to $CDCl_3$ (^{1}H: 7.26 ppm referring to residual $CHCl_3$, ^{13}C: 77.16 ppm). Coupling constants (*J*) are given in Hertz (Hz) and refer to apparent multiplicities (s = singlet, bs = broad singlet, d = doublet, bd = broad doublet, t = triplet, q = quartet, m = multiplet, dd = doublet of doublets, etc.). The ^{1}H NMR spectra are reported as follows: chemical shift (multiplicity, coupling constants, number of protons). All raw fid files were processed, and the spectra analyzed using the software MestReNova 14.3.0 from Mestrelab Research SL.

High-resolution mass spectra (HRMS) were recorded on an Agilent 6230 ESI-TOF spectrometer by Agilent Technologies, Santa Clara, CA, USA, using electrospray ionisation (ESI). The flow rate was 10 µL/min and the spray voltage 4 kV. The desolvatisation gas was set to 15 psi (1 bar). All other parameters were optimized for maximal abundance of the respective $[M+H]^{+}$. The data was processed using Masshunter 7 or 8 by Agilent.

X-Ray data were collected on a Bruker D8 Venture system. Data were collected at 100(2) or 150(2) K using graphite monochromated Mo K_{α} radiation (λ_{α} = 0.71073 Å). The strategy for data collection was evaluated by using the Smart software. The data were collected by the standard "ψ-ω scan techniques" and were scaled and reduced using Saint^{+} software. The structure was solved by using Olex2,[1] the structure was solved with the XT[2] structure solution program using Intrinsic Phasing and refined with the XL refinement package[3,4] using Least Squares minimization. Bond length and angles were measured with Diamond Crystal and Molecular Structure Visualization Version 4.6.2.[5] Drawings were generated with POV-Ray.[6]

2. Optimization Studies

Table S1. Solvent Screening.

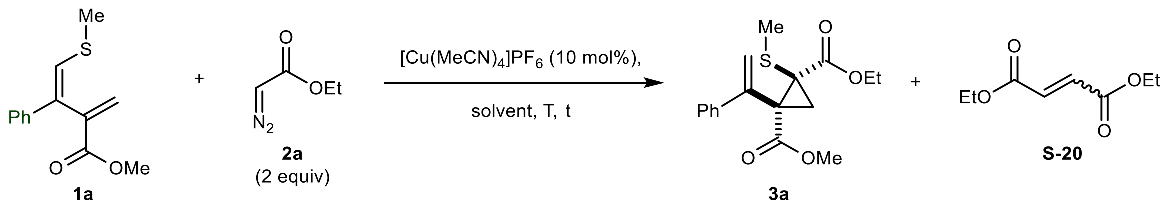

Entry	Solvent (M)	t (h)	T (°C)	Conversion (%)[a]	3a (%)[a]	S-20 [% (*E/Z*)][a]
1	CH_2Cl_2 (0.1)	2	21	95	13	12 (1.4/1)
2	CH_2Cl_2 (0.1)	2	0	42	34	20 (1.3/1)
3	DCE (0.1)	2	21	75	43	19 (0.9/1)
4	$CHCl_3$ (0.1)	2	21	50	50	16 (1.0/1)
5	$CHCl_3$ (0.1)	2	0	15	15	16 (1.1/1)
6	$CHCl_3$ (0.1)	6.25	21	50	50	19 (1.1/1)
7	$CHCl_3$ (0.1)	6.25	35	50	50	15 (0.9/1)
8	$CHCl_3$ (0.1)	0.5	21	74	30	15 (0.9/1)
9	Et_2O (0.1)	2	21	55	44	18 (1.2/1)
10	MeCN (0.1)	3	21	94	6	0
11	1,4-dioxane (0.1)	2	21	37	20	18 (1.9/1)
12	PhH (0.1)	2	21	66	40	18 (0.6/1)
13	$PhCF_3$ (0.1)	2	21	72	38	10 (0.8/1)
14	EtOAc (0.05)	3	21	84	49	14 (0.8/1)
15	MTBE (0.05)	3	21	49	49	14 (0.9/1)
16	HFIP (0.05)	3	21	40	21	2 (1.0:/1)

All reactions were run on 0.05 mmol scale of 1,3-diene **1a**. [a] ^1H NMR yield using CH_2Br_2 as internal standard.

Table S2. Catalyst Screening.

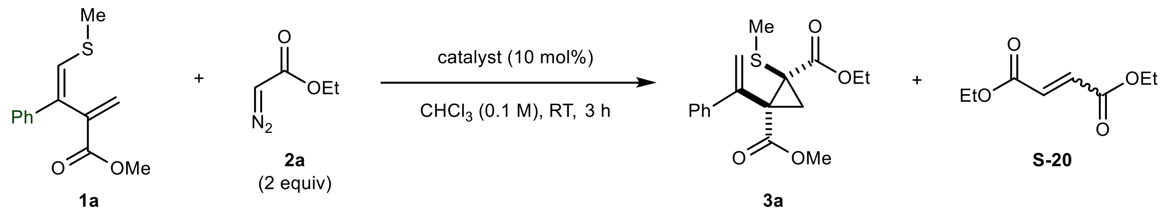

Entry	Catalyst	Conversion (%)	3a (%)[a]	S-20 [% (*E/Z*)][a]
1	$[Cu(MeCN)_4]PF_6$	50	50	16 (1.0/1)
2	$[Cu(MeCN)_4]BF_4$	80	33	23 (0.9/1)
3	$CuBr{\cdot}Me_2S$	36	0	0
4	CuCN	2	0	0
5	CuCl	66	0	0
6	$[Cu(MeCN)_4]OTf$	85	22	2 (0.3/1)
7	$Cu_2OTf_2{\cdot}PhH$	62	38	7 (0.6/1)
8	CuTC	85	43	6 (0.5/1)
9	CuI	27	3	1 (1.0/1)
10	$Cu(acac)_2$	12	0	0
11	$Cu(OAc)_2$	26	0	0
12	$Rh_2(esp)_2$[b]	70	46	62 (0.4/1)
13	$Rh_2(OAc)_4$[b]	67	44	60 (1.0/1)
14	**none**	0	0	0

All reactions were run on 0.05 mmol scale of 1,3-diene **1a**. [a] ^{1}H NMR yield using CH_2Br_2 as internal standard. [b] using 2.5 mol% of catalyst.

Table S3. Catalyst Loading.

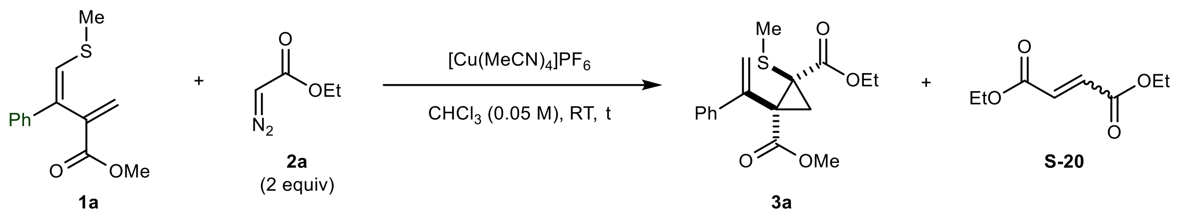

Entry	$[Cu(MeCN)_4]PF_6$ (mol%)	t (h)	Conversion (%)	3a (%)[a]	S-20 [% (*E/Z*)][a]
1	20	2	68	40	24 (1.0/1)
2	10 + 10[b]	2	73	43	17 (0.8/1)
3	20	3	75	63	29 (1.2/1)
4	10	3	88	69	35 (1.1/1)
5	5	3	86	65	36 (1.1/1)
6	2.5	3	76	68	36 (1.2/1)
7[c]	2.5	3	80	79	63 (1.3/1)
8[c]	2.5	15	83	84	61 (1.1/1)
**9[c]**	**2.5**	**15**	**85**	**77[d]**	**67 (1.2/1)**

All reactions were run on 0.05 mmol scale of 1,3-diene **1a**. [a] ^1H NMR yield using CH_2Br_2 as internal standard. [b] Addition of 10 mol% $[Cu(MeCN)_4]PF_6$ after one hour. [c] Using 3 equiv of **2a**. [d] Isolated yield, 0.2 mmol scale.

3. General Procedures

General Procedure 1 (GP1): Vinyl cyclopropane formation

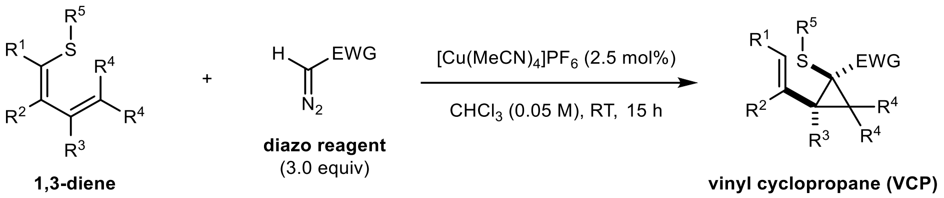

In a 20 mL reaction vial equipped with a magnetic stir bar and a red rubber septum PTFE screw cap, 1,3-diene (0.2 mmol, 1.0 equiv) and tetrakis(acetonitrile)copper(I) hexafluorophosphate ([Cu(MeCN)$_4$]PF$_6$, 1.86 mg, 5.00 µmol, 2.5 mol%) were placed, the atmosphere was changed to argon (three cycles) and dry $CHCl_3$ (1.0 mL, 0.2 M) was added. A solution of the diazo reagent in dry $CHCl_3$ (3 mL, 0.6 mmol, 3.0 equiv, 0.2 M) was added dropwise at RT. After 15 hours, the reaction mixture was filtered over a plug of deactivated silica eluted with Et_2O or MTBE containing 10% of Et_3N and the filtrate was concentrated under reduced pressure. The crude product was purified by flash-column chromatography on deactivated silica gel to furnish the pure vinyl cyclopropane (**VCP**).

Note: Due to observed instability of the VCPs under acidic conditions, flash-column and thin layer chromatography were performed using deactivated silica and TLC plates, respectively.

General Procedure 2 (GP2): Suzuki−Miyaura coupling for 2,5-dihydrothiophene diversification

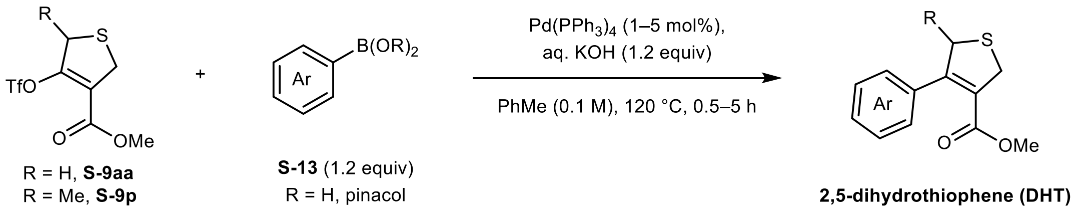

In a round-bottomed flask or reaction vial equipped with a magnetic stir bar, reflux condenser and rubber septum, triflate **S-9aa** (1.0 equiv) was dissolved in PhMe (0.1 M, technical grade) under argon atmosphere at RT. The solution was degassed by sparking with argon for 10 – 20 minutes. Then, the aryl boronic acid (R = H) or pinacol ester derivative (R = pinacol, 1.2 equiv), Pd(PPh$_3$)$_4$ (1.0 – 5.0 mol%) and aqueous KOH solution (4.0 M, 1.2 equiv) were added at RT. Upon complete addition, the reaction vessel was transferred to a preheated aluminum block (120 °C). After the indicated time (30 minutes to five hours), full conversion was confirmed via TLC analysis. The reaction mixture was allowed to cool to RT, was filtered over a plug of Celite® and rinsed with Et_2O. Water was added, the layers were separated, and the aqueous layer was extracted twice with Et_2O. The combined organic layers were dried over Na_2SO_4. The dried solution was filtered, and the filtrate was concentrated under reduced pressure. The crude product was purified by flash-column chromatography on silica gel to furnish the corresponding 2,5-dihydrothiophene (**DHT**).

General Procedure 3 (GP3): Ring-opening towards *S*-substituted 1,3-dienes

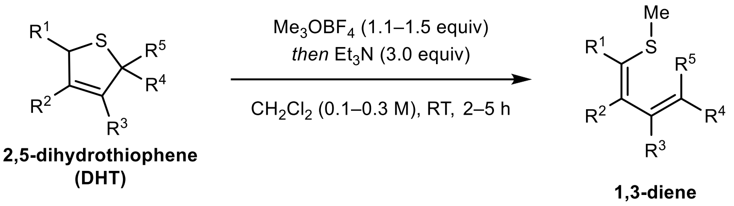

In a round-bottomed flask equipped with a rubber septum and magnetic stir bar, or a 20 mL reaction vial equipped with a red rubber septum PTFE screw cap and a magnetic stir bar, 2,5-dihydrothiophene (**DHT**) was dissolved in CH_2Cl_2 (0.1 – 0.3 M) under argon atmosphere and Me_3OBF_4 (1.1 – 1.5 equiv) was added in one portion at RT. Upon addition, the reaction mixture was kept stirring until full conversion of the starting material was observed through TLC analysis. After the indicated time (two to five hours), Et_3N (3.0 equiv) was added dropwise at RT. After additional 30 minutes to two hours, the solvent was removed under reduced pressure, and the crude product was purified by flash-column chromatography on silica gel to furnish the corresponding 1,3-diene.

4. Synthesis of Starting Materials and Reagents

The preparation of literature-known 1,3-dienes and diazo reagents depicted in Figure S1 was carried out according to reported procedures.[7−12]

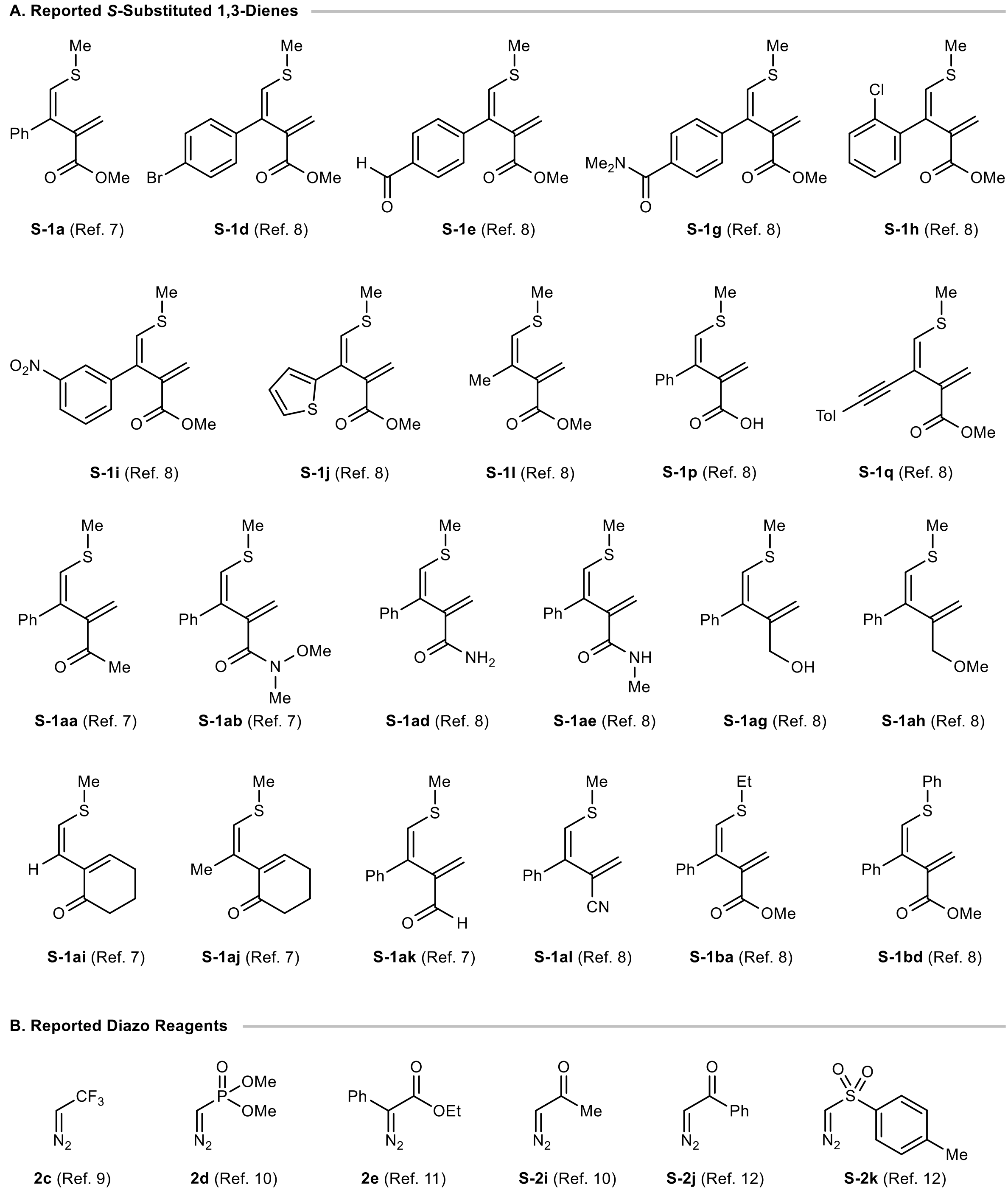

Figure S1. Synthesized 1,3-dienes (A) and diazo reagents (B) based on reported procedures.

4.1. Synthesis of 2,5-Dihydrothiophenes (DHTs)

Methyl 4-(4-methoxyphenyl)-2,5-dihydrothiophene-3-carboxylate (S-9b)

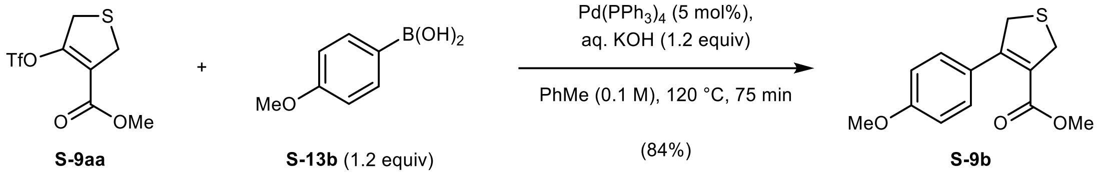

Following GP2, triflate **S-9aa** (500 mg, 1.71 mmol, 1.00 equiv) and boronic acid **S-13b** (312 mg, 2.05 mmol, 1.20 equiv) were coupled utilizing $Pd(PPh_3)_4$ (5 mol%) over 75 minutes. Purification by flash-column chromatography (10% MTBE in PE) furnished DHT **S-9b** (359 mg, 1.44 mmol, 84%) as a white solid.

TLC (20% MTBE in PE): R_f = 0.49 (UV, CAM).

^{1}H NMR (500 MHz, $CDCl_3$): δ_H [ppm] = 7.20 (d, J = 8.7 Hz, 2H), 6.88 (d, J = 8.8 Hz, 2H), 4.15 (bs, 4H), 3.82 (s, 3H), 3.62 (s, 3H).

^{13}C NMR (126 MHz, $CDCl_3$): δ_C [ppm] = 165.2, 159.9, 151.7, 129.1, 127.9, 126.8, 113.6, 55.4, 51.7, 44.7, 40.2.

HRMS (ESI^+) calc. for $C_{13}H_{14}NaO_3S$ $[M+Na]^+$: 273.0556, found: 273.0553.

Methyl 4-(4-(trifluoromethyl)phenyl)-2,5-dihydrothiophene-3-carboxylate (S-9c)

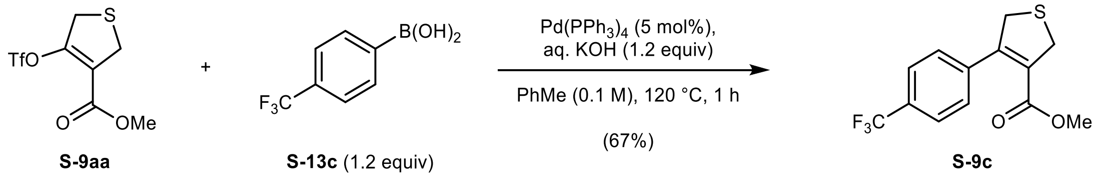

Following GP2, triflate **S-9aa** (500 mg, 1.71 mmol, 1.00 equiv) and boronic acid **S-13c** (390 mg, 2.05 mmol, 1.20 equiv) were coupled utilizing of $Pd(PPh_3)_4$ (5 mol%) over one hour. Purification by flash-column chromatography (2%→5% Et_2O in PE) furnished **S-9c** (331 mg, 1.15 mmol, 67%) as a colorless solid.

TLC (5% Et_2O in PE): R_f = 0.24 (UV, $KMnO_4$)

^{1}H NMR (500 MHz, $CDCl_3$): δ_H [ppm] = 7.63 (d, J = 7.9 Hz, 2H), 7.35 (d, J = 7.9 Hz, 2H), 4.20 – 4.10 (m, 4H), 3.60 (s, 3H).

^{13}C NMR (126 MHz, $CDCl_3$): δ_C [ppm] = 164.4, 150.9, 139.9, 130.5 (q, J = 32.5 Hz), 129.4, 127.9, 125.3 (q, J = 3.8 Hz), 124.1 (q, J = 272.5 Hz), 52.0, 45.0, 40.1.

^{19}F NMR (376 MHz, $CDCl_3$): δ_F [ppm] = −62.67.

HRMS (ESI^+) calc. for $C_{13}H_{11}F_3NaO_2S$ $[M+Na]^+$: 311.0324, found: 311.0311.

Methyl 4-(4-(ethoxycarbonyl)phenyl)-2,5-dihydrothiophene-3-carboxylate (S-9f)

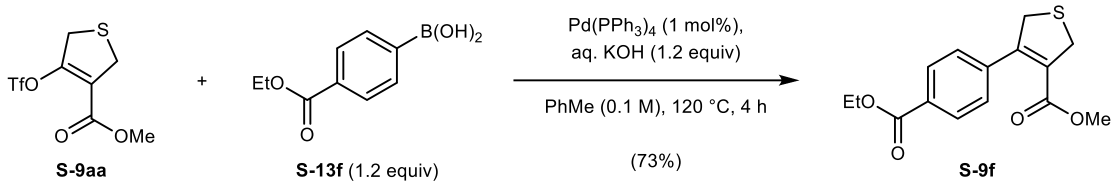

Following GP2, triflate **S-9aa** (292 mg, 1.00 mmol, 1.00 equiv) and boronic acid **S-13f** (312 mg, 2.05 mmol, 1.20 equiv) were coupled utilizing $Pd(PPh_3)_4$ (1 mol%) over four hours. Purification by flash-column chromatography (10% Et_2O in *n*-pentane) furnished **S-9f** (214 mg, 0.732 mmol, 73%) as a yellow solid.

TLC (10% EtOAc in PE): R_f = 0.30 (UV, $KMnO_4$).

^{1}H NMR (500 MHz, $CDCl_3$): δ_H [ppm] = 8.04 – 8.00 (m, 2H), 7.28 (d, *J* = 7.9 Hz, 2H), 4.36 (q, *J* = 7.1 Hz, 2H), 4.18 – 4.11 (m, 4H), 3.56 (s, 3H), 1.37 (t, *J* = 7.1 Hz, 3H).

^{13}C NMR (126 MHz, $CDCl_3$): δ_C [ppm] = 166.1, 164.5, 151.0, 140.7, 130.3, 129.4, 129.1, 127.4, 61.1, 51.7, 44.8, 40.1, 14.4.

HRMS (ESI^+) calc. for $C_{16}H_{18}NaO_4S$ $[M+Na]^+$: 315.0662; found: 315.0652.

***Tert*-butyl 5-(4-(methoxycarbonyl)-2,5-dihydrothiophen-3-yl)-1*H*-indole-1-carboxylate (S-9k)**

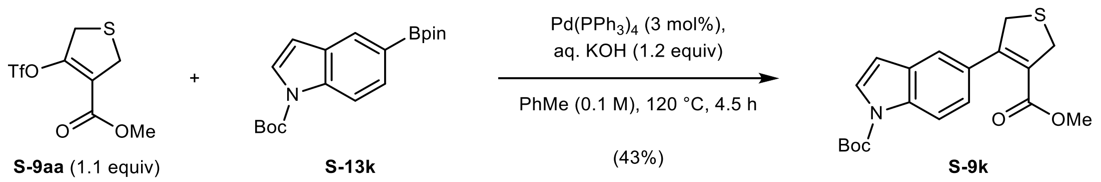

Following GP2, triflate **S-9aa** (400 mg, 1.37 mmol, 1.11 equiv) and boronic acid **S-13k** (425 mg, 1.24 mmol, 1.00 equiv) were coupled utilizing $Pd(PPh_3)_4$ (3 mol%) over 4.5 hours. Purification by flash-column chromatography (4%→20% MTBE in PE) furnished **S-9k** (191 mg, 0.530 mmol, 43%) as a colorless oil.

TLC (10% EtOAc in PE): R_f = 0.33 (UV, CAM).

^{1}H NMR (500 MHz, $CDCl_3$): δ_H [ppm] = 8.11 (m, 1H), 7.60 (d, J= 3.0 Hz, 1H), 7.44 (d, J= 1.4 Hz, 1H), 7.18 (dd, J= 8.6/1.6 Hz, 1H), 6.55 (d, J = 3.8 Hz, 1H), 4.20 (s, 4H), 3.58 (s, 3H), 1.67 (s, 9H).

^{13}C NMR (126 MHz, $CDCl_3$): δ_C [ppm] = 165.3, 152.4, 149.7, 135.1, 130.4, 130.3, 127.4, 126.7, 124.0, 119.9, 115.0, 107.5, 84.1, 51.7, 45.2, 40.3, 28.3, 25.0.

HRMS (ESI^+) calc. for $C_{19}H_{21}NNaO_4S^+$ $[M+Na]^+$: 382.1084; found: 382.1078.

Methyl 4-((triisopropylsilyl)oxy)-2,5-dihydrothiophene-3-carboxylate (S-9m)

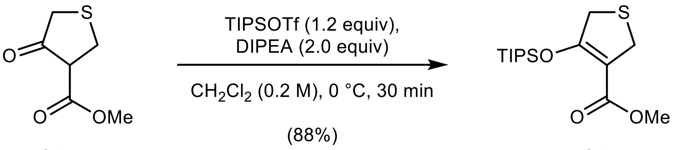

S-9ab **S-9m**

To a solution of methyl 4-oxotetrahydrothiophene-3-carboxylate (**S-9ab**, 500 mg, 3.12 mmol, 1.00 equiv) in CH_2Cl_2 (15 mL, 0.2 M), DIPEA (1.0 mL, 6.2 mmol, 2.0 equiv) and tri-*iso*-propylsilyl trifluoromethanesulfonate (TIPSOTf, 1.0 mL, 3.8 mmol, 1.2 equiv) were added at 0 °C. After 30 minutes, water (15 mL) was added, the layers were separated and the aqueous layer was extracted with CH_2Cl_2 (2 × 10 mL). The combined organic layers were dried over Na_2SO_4. The dried solution was filtered, and the filtrate was concentrated under reduced pressure. Filtration over a plug of silica gel (10% MTBE in PE) furnished DHT **S-9m** as a colorless oil (872 mg, 2.75 mmol, 88%).

TLC (10% MTBE in PE): R_f = 0.78 (UV, $KMnO_4$).

^{1}H NMR (500 MHz, $CDCl_3$): δ_H [ppm] = 3.84 – 3.82 (m, 2H), 3.78 – 3.74 (m, 2H), 3.71 (s, 3H), 1.27 – 1.17 (m, 3H), 1.11 (d, J = 6.6 Hz, 18H).

^{13}C NMR (126 MHz, $CDCl_3$): δ_C [ppm] = 164.6, 161.7, 107.3, 51.0, 38.8, 33.1, 17.8, 13.2.

HRMS (ESI$^+$) calc. for $C_{15}H_{28}NaO_3SSi$ $[M+Na]^+$: 339.1421; found: 339.1428.

Methyl 2-mercaptopropanoate (S-9s)

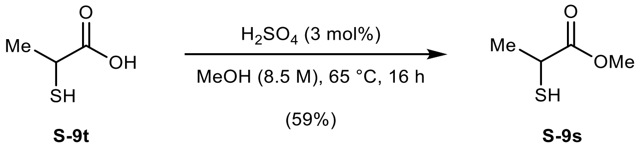

S-9t **S-9s**

Methyl 2-mercaptopropanoate (**S-9s**) was prepared according to a literature procedure.[13] To a solution of 2-mercaptopropanoic acid **S-9t** (1.5 mL, 17 mmol, 1.0 equiv) in methanol (2.0 mL, 8.5 M), concentrated sulfuric acid (52 µL, 0.50 mmol, 3.0 mol%) was added and the mixture was heated to 65 °C overnight. The solution was cooled to RT and sodium acetate (0.400 g, 4.88 mmol, 0.287 equiv) was added. Water (10 mL) and CH_2Cl_2 (20 mL) were added to the mixture. The layers were separated, and the aqueous layer was extracted with CH_2Cl_2 (3 × 20 mL). The combined organic layers were dried over Na_2SO_4. The dried solution was filtered, and the filtrate was concentrated under reduced pressure to furnish **S-9s** (1.19 g, 9.91 mmol, 59%) as a colorless liquid. The analytical data obtained were in full agreement with those reported in the literature.[13]

TLC (10% EtOAc in PE): R_f= 0.65 (UV, CAM).

Methyl 4-hydroxy-5-methyl-2,5-dihydrothiophene-3-carboxylate (S-9q)

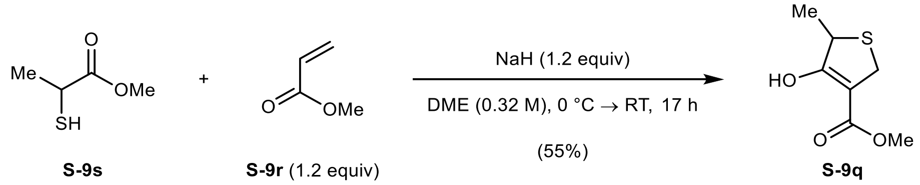

To a solution of methyl 2-mercaptopropanoate **S-9s** (1.0 mL, 9.5 mmol, 1.0 equiv) in dry 1,2-dimethoxyethane (DME, 30 mL, 0.32 M) in a 100 mL Schlenk flask, methyl acrylate **S-9r** (1.0 mL, 11 mmol, 1.2 equiv) was added at RT. Upon addition, the solution was cooled to 0 °C. After 30 minutes, sodium hydride (60% dispersion in mineral oil, 460 mg, 11.4 mmol, 1.20 equiv) was added in small portions (caution: gas extrusion!). After complete addition, the reaction mixture was allowed to warm to RT. After 17 hours, saturated aq. NH_4Cl solution (30 mL) and MTBE (50 mL) were added, the layers were separated and the aqueous layer was extracted with MTBE (3 × 20 mL). The combined organic layers were dried over Na_2SO_4. The dried solution was filtered, and the filtrate was concentrated under reduced pressure. Purification by flash-column chromatography on silica gel (5% MTBE in PE) to furnish DHT **S-9q** (920 mg, 5.27 mmol, 55%) as a slightly pink oil.

TLC (10% MTBE in PE): R_f= 0.43 (UV, $KMnO_4$, CAM).

^{1}H NMR (500 MHz, $CDCl_3$): δ_H [ppm] = 10.97 (s, 1H), 4.21 (ddd, J = 6.9, 4.5, 2.1 Hz, 1H), 3.79 (s, 3H, *signals contributing to the minor keto tautomer*), 3.74 (dd, J = 12.1, 4.5 Hz, 1H), 3.68 (dd, J = 12.1, 2.0 Hz, 1H), 1.51 (d, J = 6.9 Hz, 3H).

^{13}C NMR (126 MHz, $CDCl_3$): δ_C [ppm] = 175.4, 170.0, 98.2, 51.8, 45.9, 30.0, 21.4.

HRMS (ESI^+) calc. for $C_7H_{10}NaO_3S$ $[M+Na]^+$: 197.0243; found: 197.0250.

Methyl 5-methyl-4-(((trifluoromethyl)sulfonyl)oxy)-2,5-dihydrothiophene-3-carboxylate (S-9p)

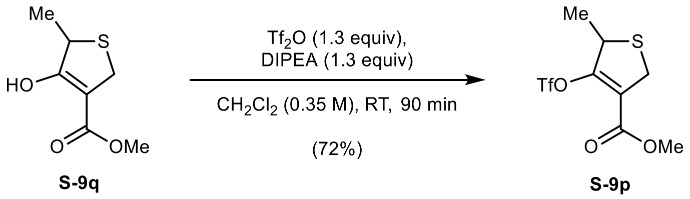

To a solution of enol **S-9q** (910 mg, 5.20 mmol, 1.00 equiv) in CH_2Cl_2 (15 mL, 0.35 M) placed in a 50 mL Schlenk flask, DIPEA (0.880 g, 6.80 mmol, 1.15 mL, 1.30 equiv) was added at RT and the solution was cooled to 0 °C. After 30 minutes, triflic anhydride (Tf_2O, 1.10 mL, 6.80 mmol, 1.30 equiv) was added dropwise and upon addition, the reaction mixture was allowed to warm to RT. After one hour, water (20 mL) and CH_2Cl_2 (2 mL) were added, the organic layer was separated, and the aqueous layer was extracted with CH_2Cl_2 (2 × 20 mL). The combined organic layers were dried over Na_2SO_4. The dried solution was filtered, and the filtrate was concentrated under reduced pressure. Purification by flash-column chromatography on silica gel (1% MTBE in PE) to furnish triflate **S-9p** (1.15 g, 3.74 mmol, 72%) as a colorless oil.

TLC (10% MTBE in PE): R_f = 0.47 (UV, $KMnO_4$, CAM).

^{1}H NMR ($CDCl_3$, 500 MHz): δ_H [ppm] = 4.42 – 4.33 (m, 1H), 3.98 – 3.86 (m, 2H), 3.83 (s, 3H), 1.54 (d, J = 6.8 Hz, 3H).

^{13}C NMR (126 MHz, $CDCl_3$): δ_C [ppm] = 161.9, 154.3, 122.4, 118.4 (q, *J* = 320.3 Hz), 52.5, 45.4, 31.4, 21.6.

^{19}F NMR (376 MHz, $CDCl_3$): δ_F [ppm] = −74.07.

HRMS (ESI^+) calc. for $C_8H_9F_3NaO_5S_2$ $[M+Na]^+$: 328.9736; found: 328.9730.

Methyl 5-methyl-4-phenyl-2,5-dihydrothiophene-3-carboxylate (S-9o)

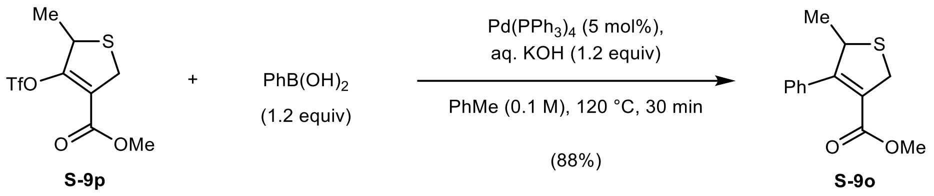

Following GP2, triflate **S-9p** (400 mg, 1.31 mmol, 1.00 equiv) and boronic acid **S-13a** (117 mg, 0.958 mmol, 1.20 equiv) were coupled utilizing $Pd(PPh_3)_4$ (5 mol%) over 30 minutes. Purification by flash-column chromatography on silica gel (5% Et_2O in PE) furnished the title compound **S-9o** (268 mg, 1.15 mmol, 88%) as a colorless oil.

TLC (10% Et_2O in PE): R_f= 0.43 (UV, $KMnO_4$, CAM).

^{1}H NMR (500 MHz, $CDCl_3$): δ_H [ppm] = 7.40 – 7.31 (m, 3H), 7.20 – 7.12 (m, 2H), 4.61 - 4.52 (m, 1H), 4.17 (dd, J = 15.0, 5.8 Hz, 1H), 4.07 (dd, J = 15.0, 2.7 Hz, 1H), 3.56 (s, 3H), 1.29 (d, J = 6.9 Hz, 3H).

^{13}C NMR (126 MHz, $CDCl_3$): δ_C [ppm] = 165.1, 157.0, 135.8, 128.3, 128.2, 127.7, 127.5, 54.1, 51.7, 38.4, 22.9.

HRMS (ESI^+) calc. for $C_{13}H_{14}NaO_2S$ $[M+Na]^+$: 257.0607; found: 257.0609.

4.2. Synthesis of *S*-Substituted 1,3-Dienes

Methyl (*Z*)-3-(4-methoxyphenyl)-2-methylene-4-(methylthio)but-3-enoate (S-1b)

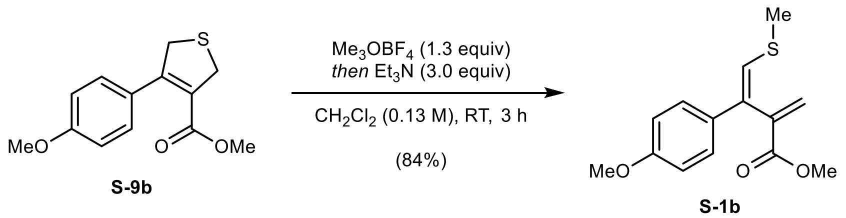

Following GP3, DHT **S-9b** (160 mg, 0.640 mmol, 1.00 equiv) and Me_3OBF_4 (118 mg, 0.800 mmol, 1.30 equiv) were converted into 1,3-diene **S-1b** (1 hour for *S*-methylation, 3 hours in total). Purification by flash-column chromatography on silica gel (10%→50% Et_2O in PE) furnished **S-1b** (142 mg, 0.540 mmol, 84%) as a yellow oil.

TLC (20% MTBE in PE): R_f = 0.38 (UV, CAM).

^{1}H NMR (500 MHz, $CDCl_3$): δ_H [ppm] = 7.22 – 7.17 (m, 2H), 6.83 – 6.79 (m, 2H), 6.59 (s, 1H), 6.45 (s, 1H), 5.79 (s, 1H), 3.77 (s, 3H), 3.68 (s, 3H), 2.34 (s, 3H).

^{13}C NMR (126 MHz, $CDCl_3$): δ_C [ppm] = 166.7, 158.8, 138.8, 134.1, 132.4, 130.7, 127.4, 126.7, 113.8, 55.2, 52.2, 17.6.

HRMS (ESI$^+$) calc. for $C_{14}H_{16}NaO_3S$ $[M+Na]^+$: 287.0712; found: 287.0721.

Methyl (*Z*)-2-methylene-4-(methylthio)-3-(4-(trifluoromethyl)phenyl)but-3-enoate (S-1c)

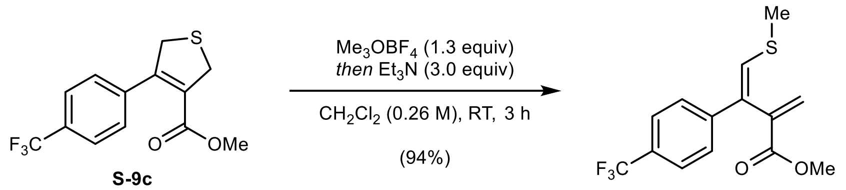

Following GP3, DHT **S-9c** (150 mg, 0.520 mmol, 1.00 equiv) and Me_3OBF_4 (97 mg, 0.65 mmol, 1.3 equiv) were converted into 1,3-diene **S-1c** (1 hour for *S*-methylation, 3 hours in total). Purification by filtration over a plug of silica gel (Et_2O) furnished **S-1c** (148 mg, 0.490 mmol, 94%) as a pale-yellow solid.

TLC (20% MTBE in PE): R_f = 0.68 (UV, CAM).

^{1}H NMR (500 MHz, $CDCl_3$): δ_H [ppm] = 7.53 (d, J = 8.2 Hz, 2H), 7.36 (d, J = 8.1 Hz, 2H), 6.72 (s, 1H), 6.69 (d, J = 1.3 Hz, 1H), 5.88 (d, J = 1.3 Hz, 1H), 3.69 (s, 3H), 2.41 (s, 3H).

^{13}C NMR (126 MHz, $CDCl_3$): δ_C [ppm] = 166.3, 143.0, 138.0, 132.7, 132.7, 131.9, 128.8 (q, J = 32.6), 125.6, 125.6, 125.6, 125.5, 123.3, 52.4, 17.8.

^{19}F NMR (376 MHz, $CDCl_3$): δ_F [ppm] = –62.28.

HRMS (ESI$^+$) calc. for $C_{14}H_{13}F_3NaO_2S$ $[M+Na]^+$: 325.0481, found: 325.0485.

Ethyl (*Z*)-4-(3-(methoxycarbonyl)-1-(methylthio)buta-1,3-dien-2-yl)benzoate (S-1f)

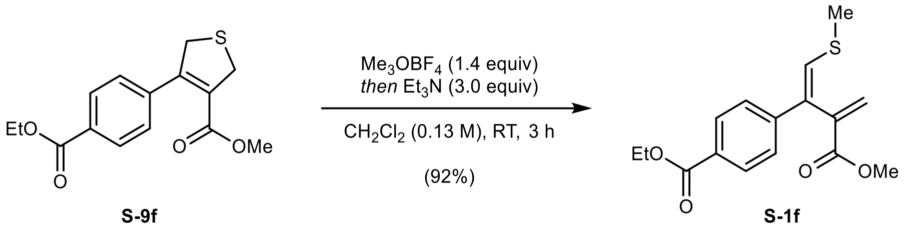

Following GP3, DHT **S-9f** (150 mg, 0.510 mmol, 1.00 equiv) and Me_3OBF_4 (103 mg, 0.700 mmol, 1.36 equiv) were converted into 1,3-diene **S-1f** (70 minutes for *S*-methylation, 3 hours in total). Purification by flash-column chromatography on silica gel (10%→50% Et_2O in PE) furnished **S-1f** (145 mg, 0.470 mmol, 92%) as a colorless oil.

TLC (20% MTBE in PE): R_f = 0.29 (UV, CAM).

^{1}H NMR (500 MHz, $CDCl_3$): δ_H [ppm] = 7.95 (d, J = 8.6 Hz, 2H), 7.31 (d, J = 8.5 Hz, 2H), 6.76 (s, 1H), 6.69 (d, J = 1.4 Hz, 2H), 5.88 (s, 1H), 4.36 (q, J = 7.1 Hz, 2H), 3.68 (s, 3H), 2.42 (s, 3H), 1.38 (t, J = 7.1 Hz, 3H).

^{13}C NMR (126 MHz, $CDCl_3$): δ_C [ppm] = 166.5, 166.4, 143.8, 138.1, 133.1, 132.6, 131.8, 129.9, 128.8, 125.2, 61.0, 52.4, 17.8, 14.5.

HRMS (ESI^+) calc. for $C_{16}H_{18}NaO_4S$ $[M+Na]^+$: 329.0818, found: 329.0813.

***Tert*-butyl (*Z*)-5-(3-(methoxycarbonyl)-1-(methylthio)buta-1,3-dien-2-yl)-1H-indole-1-carboxylate (S-1k)**

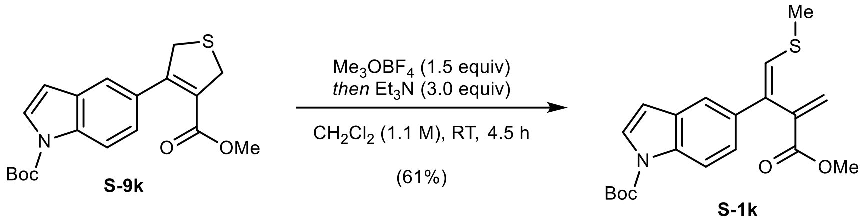

Following GP3, DHT **S-9k** (190 mg, 0.529 mmol, 1.00 equiv) and Me_3OBF_4 (118 mg, 0.978 mmol, 1.51 equiv) were converted into 1,3-diene **S-1k** (4 hours for *S*-methylation, 4.5 hours in total). Purification by flash-column chromatography on silica gel (10%→20% Et_2O in PE) furnished **S-1k** (120 mg, 0.320 mmol, 61%) as a yellow oil.

TLC (15% EtOAc in PE): R_f = 0.43 (UV, CAM).

^{1}H NMR (500 MHz, $CDCl_3$): δ_H [ppm] = 8.03 – 7.99 (m, 1H), 7.56 (d, J = 3.5 Hz, 1H), 7.42 (d, J = 1.8 Hz, 1H), 7.28 (dd, J = 8.7, 2.0 Hz, 1H), 6.65 (d, J = 1.5 Hz, 1H), 6.57 (s, 1H), 6.52 (d, J = 3.8 Hz, 1H), 5.86 (d, J = 1.5 Hz, 1H), 3.67 (s, 3H), 2.39 (s, 3H), 1.66 (s, 9H).

^{13}C NMR (126 MHz, $CDCl_3$): δ_C [ppm] = 166.9, 149.8, 139.0, 134.9, 134.7, 130.9, 128.6, 128.6, 126.5, 122.3, 118.1, 115.2, 107.6, 107.6, 83.8, 52.4, 28.3, 17.8.

HRMS (ESI^+) calc. for $C_{20}H_{23}NNaO_4S$ $[M+Na]^+$: 396.1240, found: 396.1257.

Methyl (*E*)-2-methylene-4-(methylthio)-3-((triisopropylsilyl)oxy)but-3-enoate (S-1m)

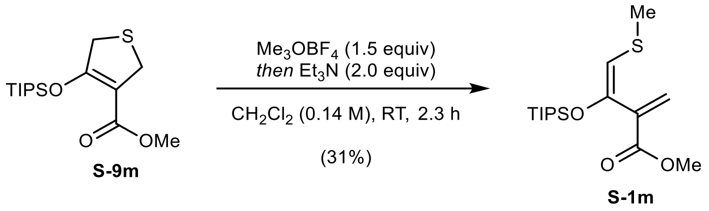

Following GP3, DHT **S-9m** (1.19 g, 92%, 3.45 mmol, 1.00 equiv) and Me_3OBF_4 (765 mg, 5.17 mmol, 1.50 equiv) were converted into 1,3-diene **S-1m**. After 2 hours, the mixture was cooled to 0 °C before Et_3N (0.96 ml, 6.9 mmol, 2.0 equiv) was added and stirred for 20 min. Purification by flash-column chromatography on silica gel (10%→20% Et_2O in PE) furnished **S-1m** (355 mg, 1.07 mmol, 31%) as a colorless oil.

TLC (5% MTBE in PE): $R_f = 0.29$ (UV, $KMnO_4$).

^{1}H NMR (500 MHz, $CDCl_3$): δ_H [ppm] = 6.20 (s, 1H), 5.77 (s, 1H), 5.44 (s, 1H), 3.75 (s, 3H), 2.10 (s, 3H), 1.21 – 1.13 (m, 3H), 1.09 – 1.03 (m, 18H).

^{13}C NMR (126 MHz, $CDCl_3$): δ_C [ppm] = 166.9, 150.1, 138.2, 127.6, 106.2, 52.2, 18.8, 18.0, 12.7.

HRMS (ESI^+) calc. for $C_{16}H_{30}NaO_3SSi$ $[M+Na]^+$: 353.1577 found: 353.1587.

Methyl (*Z*)-2-methylene-4-(methylthio)-3-phenylpent-3-enoate (S-1o)

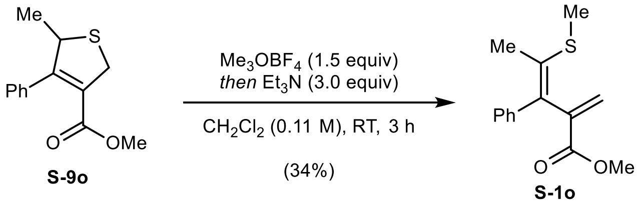

Following GP3, DHT **S-9o** (158 mg, 0.674 mmol, 1.00 equiv) and Me_3OBF_4 (150 mg, 1.01 mmol, 1.50 equiv) were converted into 1,3-diene **S-1o** (70 minutes for *S*-methylation, 3 hours in total). Purification by flash-column chromatography on silica gel (2% of MTBE in PE + 1% Et_3N) furnished **S-1o** (57.1 mg, 0.230 mmol, 34%) as a colorless oil.

TLC (20% MTBE in PE): $R_f = 0.43$ (UV, $KMnO_4$, CAM).

^{1}H NMR (500 MHz, $CDCl_3$): δ_H [ppm] = 7.31 (t, J = 7.3 Hz, 2H), 7.26 – 7.18 (m, 3H), 6.28 (d, J = 1.4 Hz, 1H), 5.53 (d, J = 1.4 Hz, 1H), 3.75 (s, 3H), 2.26 (s, 3H), 2.03 (s, 3H).

^{13}C NMR (126 MHz, $CDCl_3$): δ_C [ppm] = 167.6, 142.4, 141.1, 135.6, 133.5, 129.3, 128.5, 128.2, 127.0, 52.2, 19.5, 15.2.

HRMS (ESI^+) calc. for $C_{14}H_{16}NaO_2S$ $[M+Na]^+$: 271.0763, found: 271.0773.

Methyl (*Z*)-4-((2-ethoxy-2-oxoethyl)thio)-2-methylene-3-phenylbut-3-enoate (S-1bb)

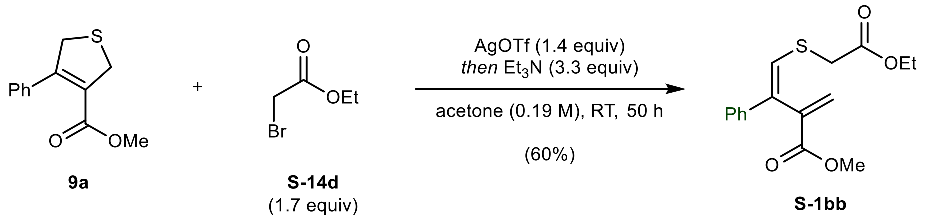

To a solution of DHT **9a** (300 mg, 1.36 mmol, 1.00 equiv) in acetone (7 mL, 0.19M), ethyl bromoacetate (**S-14d**, 0.25 mL, 2.3 mmol, 1.7 equiv) and AgOTf (471 mg, 1.85 mmol, 1.35 equiv) were added at RT. After 48 hours, Et_3N (0.62 mL, 4.5 mmol, 3.3 equiv) was added dropwise. After two hours, the solvent was removed under reduced pressure. Purification by flash-column chromatography on silica gel (10%→25% MTBE in PE) furnished 1,3-diene **S-1bb** (248 mg, 0.810 mmol, 60%) as a pale-yellow solid.

TLC (20% MTBE in PE): $R_f = 0.31$ (UV, CAM, $KMnO_4$).

^{1}H NMR (500 MHz, $CDCl_3$): δ_H [ppm] = 7.31 – 7.18 (m, 5H), 6.71 (s, 1H), 6.64 (d, J = 1.4 Hz, 1H), 5.83 (d, J = 1.5 Hz, 1H), 4.20 (q, J = 7.1 Hz, 2H), 3.65 (s, 3H), 3.42 (s, 2H), 1.27 (t, J = 7.1 Hz, 3H).

^{13}C NMR (126 MHz, $CDCl_3$): δ_C [ppm] = 169.7, 166.4, 139.3, 138.2, 136.4, 131.5, 128.6, 127.5, 125.8, 125.7, 61.8, 52.3, 35.7, 14.3.

HRMS (ESI$^+$) calc. for $C_{16}H_{18}NaO_4S$ $[M+Na]^+$: 329.0818, found: 329.0826.

Methyl (*Z*)-4-(benzylthio)-2-methylene-3-phenylbut-3-enoate (S-1bc)

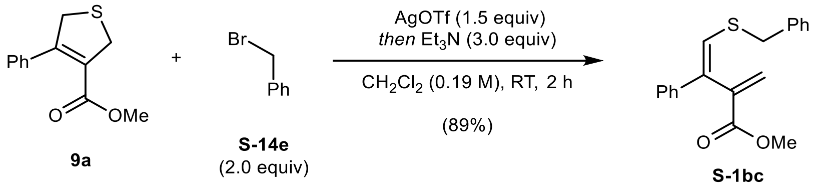

To a solution of DHT **9a** (220 mg, 1.00 mmol, 1.00 equiv) in CH_2Cl_2 (4.8 mL, 0.19 M), benzyl bromide **S-14e** (0.24 mL, 2.0 mmol, 2.0 equiv) and AgOTf (386 mg, 1.50 mmol, 1.50 equiv) were added at RT. After one hour, Et_3N (0.42 mL, 3.0 mmol, 3.0 equiv) was added dropwise at RT. After one hour, the reaction mixture was filtered, the solvent was removed under reduced pressure. Purification by flash-column chromatography on silica gel (7% MTBE in PE) furnished 1,3-diene **S-1bc** (277 mg, 0.892 mmol, 89%) as a pale-yellow oil.

TLC (20% MTBE in CyH): $R_f = 0.65$ (UV, CAM).

^{1}H NMR (500 MHz, $CDCl_3$): δ_H [ppm] = 7.36 – 7.18 (m, 10H), 6.62 – 6.60 (m, 2H), 5.78 (d, J = 1.4 Hz, 1H), 3.98 (s, 2H), 3.65 (s, 3H).

^{13}C NMR (126 MHz, $CDCl_3$): δ_C [ppm] = 166.7, 139.7, 138.7, 137.6, 135.4, 131.2, 129.0, 128.8, 128.6, 127.5, 127.2, 126.8, 125.7, 52.3, 38.6.

HRMS (ESI$^+$) calc. for $C_{19}H_{18}NaO_2S$ $[M+Na]^+$: 333.0920; found: 333.0933.

Methyl (*Z*)-4-(allylthio)-2-methylene-3-phenylbut-3-enoate (S-1be)

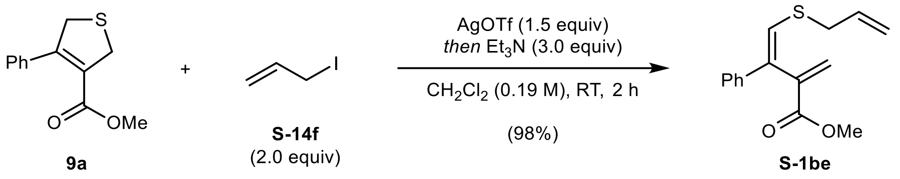

To a solution of DHT **9a** (100 mg, 0.454 mmol, 1.00 equiv) in CH_2Cl_2 (2.4 mL, 0.19 M), allyl iodide **S-14f** (86 μL, 0.91 mmol, 2.0 equiv, 97%) and AgOTf (175 mg, 0.681 mmol, 1.50 equiv) were added at RT. The reaction mixture was stirred for 1 hour. Et_3N (189 μL, 1.36 mmol, 3.00 equiv) was added dropwise at RT, and the mixture was stirred for another hour. Subsequently, the mixture was filtered, and the solvent was removed under reduced pressure. Purification by flash-column chromatography on silica gel (10% EtOAc in CyH) furnished 1,3-diene **S-1be** (115 mg, 0.443 mmol, 98%) as a colorless oil.

TLC (10% EtOAc in CyH): R_f = 0.42 (UV, CAM).

^{1}H NMR (500 MHz, $CDCl_3$): δ_H [ppm] = 7.36 – 7.19 (m, 5H), 6.68 (d, J = 1.2 Hz, 1H), 6.56 (s, 1H), 5.96 – 5.88 (m, 1H), 5.88 (d, J = 1.4 Hz, 1H), 5.29 (dd, J = 17.0, 1.4 Hz, 1H), 5.22 (dd, J = 9.9, 1.3 Hz, 1H), 3.73 (s, 3H), 3.43 (d, J = 7.0 Hz, 2H).

^{13}C NMR (126 MHz, $CDCl_3$): δ_C [ppm] = 166.7, 139.8, 138.8, 135.0, 134.1, 131.2, 128.6, 127.2, 126.7, 125.6, 118.1, 52.3, 37.1.

HRMS (ESI$^+$) calc. for $C_{15}H_{16}NaO_2S$ $[M+Na]^+$: 283.0763, found: 283.0777.

Methyl (*Z*)-4-(((diethoxyphosphoryl)methyl)thio)-2-methylene-3-phenylbut-3-enoate (S-1ca)

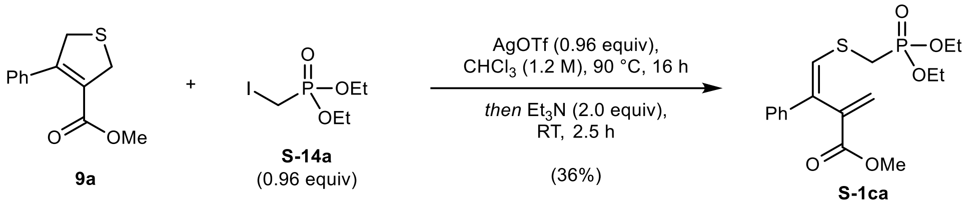

To a solution of DHT **9a** (104 mg, 0.472 mmol, 1.00 equiv) in $CHCl_3$ (4.0 mL, 1.2 M), α-iodophosphonate **S-14a** (76 μL, 0.45 mmol, 0.96 equiv) and AgOTf (117 mg, 0.455 mmol, 0.960 equiv) were added at RT. Upon addition, the reaction mixture was heated to 90 °C. After 16.5 hours, the reaction was allowed to cool to RT and Et_3N (0.13 mL, 0.94 mmol, 2.0 equiv) was added dropwise. After 2.5 hours, the solvent was removed under reduced pressure. Purification by flash-column chromatography on silica gel (first: 5%→66% EtOAc in PE; second: 0.5%→99% *i*-PrOH in PE + 1% Et_3N) furnished 1,3-diene **S-1ca** (62.0 mg, 0.167 mmol, 36%) as a colorless, crystalline solid.

TLC (7.5% *i*-PrOH in PE): R_f = 0.25 (UV, $KMnO_4$, CAM).

^{1}H NMR (500 MHz, $CDCl_3$): δ_H [ppm] = 7.31 – 7.17 (m, 5H), 6.81 (s, 1H), 6.64 (d, J = 1.5 Hz, 1H), 5.83 (d, J = 1.5 Hz, 1H), 4.22 – 4.13 (m, 4H), 3.64 (s, 3H), 2.96 (d, J = 12.8 Hz, 2H), 1.32 (t, J = 7.1 Hz, 6H).

^{13}C NMR (126 MHz, $CDCl_3$): δ_C [ppm] = 166.4, 139.2, 138.1, 135.8, 131.5, 128.6, 127.4, 126.6 (d, J = 2.8 Hz), 125.6, 63.0 (d, J = 6.7 Hz), 52.3, 27.5 (d, J = 150.3 Hz), 16.6 (d, J = 5.9 Hz).

^{31}P{^{1}H,^{13}C}-NMR (162 MHz, $CDCl_3$): δ_P [ppm] = 23.52 (s).

HRMS (ESI) calc. for $C_{17}H_{23}NaO_5PS$ $[M+Na]^+$: 393.0896; found: 393.0911.

Methyl (*Z*)-2-methylene-3-phenyl-4-((2,2,2-trifluoroethyl)thio)but-3-enoate (S-1cb)

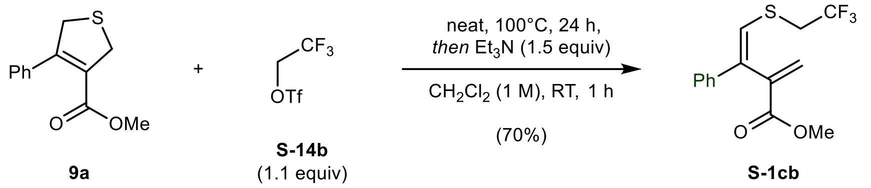

DHT **9a** (110 mg, 0.500 mmol, 1.00 equiv) and 2,2,2-trifluoroethyl triflate **S-14b** (128 mg, 0.550 mmol, 1.10 equiv) were added to a 1 mL vial at RT and heated to 100 °C (sand bath). After 24 hours, the reaction mixture was allowed to cool to RT. The reaction mixture was dissolved by the addition of CH_2Cl_2 (0.5 mL, 1 M) and Et_3N (0.10 mL, 0.75 mmol, 1.5 equiv) was added dropwise. Upon addition, the reaction mixture was stirred for another hour before the solvent was removed under reduced pressure. Purification by flash-column chromatography on silica gel (0%→5% EtOAc in PE) furnished 1,3-diene **S-1cb** (106 mg, 0.350 mmol, 70%) as a colorless oil.

TLC (15% EtOAc in PE): R_f = 0.57 (UV, $KMnO_4$).

^{1}H NMR (500 MHz, $CDCl_3$): δ_H [ppm] = 7.33 − 7.24 (m, 5H), 6.67 (d, J = 1.3 Hz, 1H), 6.56 (s, 1H), 5.85 (d, J = 1.2 Hz, 1H), 3.69 (s, 3H), 3.31 (q, J = 9.5 Hz, 2H).

^{13}C NMR (126 MHz, $CDCl_3$): δ_C [ppm] = 166.3, 139.1, 138.2, 137.7, 131.7, 128.7, 127.8, 125.9, 125.3 (q, J = 277.2 Hz), 52.4, 36.5 (q, J = 33.0 Hz), 29.8.

^{19}F NMR (376 MHz, $CDCl_3$): δ_F [ppm] = −66.52 (s).

HRMS (ESI^+) calc. for $C_{14}H_{13}F_3NaO_2S$ $[M+Na]^+$: 325.0484, found: 325.0484.

Methyl (*Z*)-4-((cyanomethyl)thio)-2-methylene-3-phenylbut-3-enoate (S-1cc)

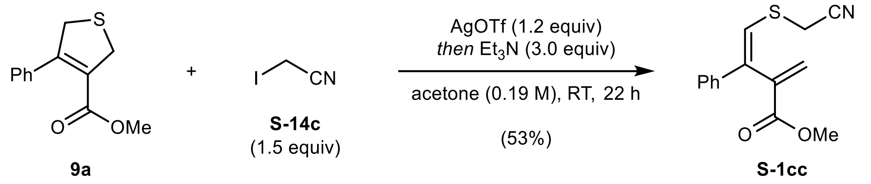

To a solution of DHT **9a** (150 mg, 0.681 mmol, 1.00 equiv) in acetone (3.5 mL, 0.19 M), iodoacetonitrile **S-14c** (78 µL, 1.0 mmol, 1.5 equiv) and AgOTf (216 mg, 0.841 mmol, 1.23 equiv) were added at RT. After 22 hours, Et_3N (0.28 mL, 2.0 mmol, 3.0 equiv) was added dropwise. After 15 minutes, the solvent

was removed under reduced pressure. Purification by flash-column chromatography on silica gel (25%→50% MTBE in PE) furnished 1,3-diene **S-1cc** (93 mg, 0.36 mmol, 53%) as an orange oil.

TLC (20% MTBE in PE): R_f = 0.14 (UV, $KMnO_4$).

^{1}H NMR (500 MHz, $CDCl_3$): δ_H [ppm] = 7.36 – 7.27 (m, 5H), 6.66 (d, *J* = 1.2 Hz, 1H), 6.62 (s, 1H), 5.83 (d, *J* = 1.3 Hz, 1H), 3.70 (s, 3H), 3.49 (s, 2H).

^{13}C NMR (126 MHz, $CDCl_3$): δ_C [ppm] = 166.1, 140.2, 138.7, 138.2, 131.7, 128.7, 128.2, 126.1, 122.0, 116.1, 52.5, 19.2.

HRMS (ESI^+) calc. for $C_{14}H_{13}NNaO_2S$ $[M+Na]^+$: 282.0559; found: 282.0563.

5. Synthesis of Vinyl Cyclopropanes

5.1. Substrate Scope

1-Ethyl 2-methyl 1-(methylthio)-2-(1-phenylvinyl)cyclopropane-1,2-dicarboxylate (3a)

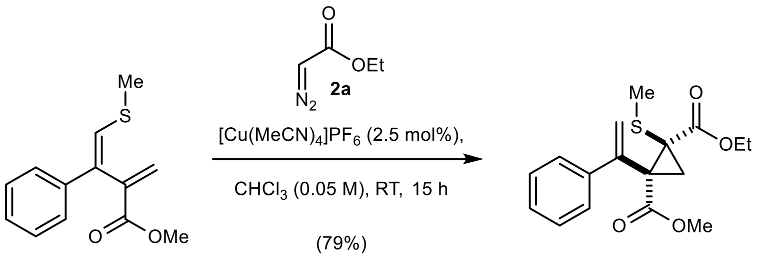

1a **3a**

Following GP1, 1,3-diene **1a** (46.9 mg, 0.200 mmol, 1.00 equiv) was converted into VCP **3a**. Purification by flash-column chromatography on silica gel (10%→20% MTBE in PE + 2% Et_3N) furnished VCP **3a** (53.3 mg, 0.159 mmol, 79%) as a pale-yellow oil.

TLC (20% MTBE in PE): R_f = 0.27 (UV, CAM).

^{1}H NMR (500 MHz, $CDCl_3$): δ_H [ppm] = 7.67 – 7.63 (m, 2H), 7.38 – 7.27 (m, 4H), 5.93 (s, 1H), 5.36 (s, 1H), 4.35 – 4.20 (m, 2H), 3.61 (s, 3H), 2.42 (d, J = 5.2 Hz, 1H), 2.07 (s, 3H), 1.60 (d, J = 5.3 Hz, 1H), 1.35 (t, J = 7.1 Hz, 3H).

^{13}C NMR (126 MHz, $CDCl_3$): δ_C [ppm] =171.5, 169.1, 142.2, 138.6, 128.4, 128.0, 126.3, 118.3, 61.8, 52.9, 42.7, 41.7, 28.4, 15.8, 14.2.

HRMS (ESI$^+$) calc. for $C_{17}H_{20}NaO_6S$ [M+Na]$^+$: 343.0975, found: 343.0973.

Scale-Up Experiment: Following GP1, 1,3-diene **1a** (1.20 g, 5.12 mmol, 1.00 equiv) was converted into VCP **3a**. Purification by flash-column chromatography on silica gel (20% MTBE in PE + 1% Et_3N) furnished VCP **3a** (1.13 g, 3.53 mmol, 69%) as a pale-yellow oil which solidified to a colorless solid upon storage at –20 °C. The obtained analytical data were in full agreement with those obtained from the previous experiment on 0.2 mmol scale.

1-Ethyl 2-methyl 2-(1-(4-methoxyphenyl)vinyl)-1-(methylthio)cyclopropane-1,2-dicarboxylate (3b)

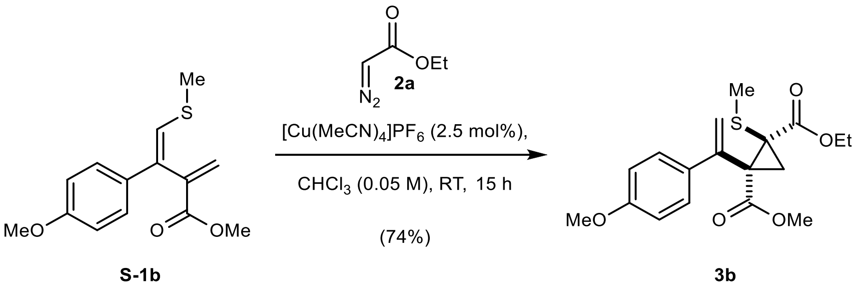

S-1b **3b**

Following GP1, 1,3-diene **S-1b** (52.9 mg, 0.200 mmol, 1.00 equiv) was converted into VCP **3b**. Purification by flash-column chromatography on silica gel (10%→20% MTBE in PE + 2% Et_3N) furnished VCP **3b** (52.0 mg, 0.148 mmol, 74%) as a yellow oil.

TLC (20% MTBE in PE): R_f = 0.23 (UV, CAM).

^{1}H NMR (400 MHz, $CDCl_3$): δ_H [ppm] = 7.64 – 7.56 (m, 2H), 6.93 – 6.85 (m, 2H), 5.82 (s, 1H), 5.24 (s, 1H), 4.36 – 4.19 (m, 2H), 3.82 (s, 3H), 3.61 (s, 3H), 2.40 (d, *J* = 5.2 Hz, 1H), 2.08 (s, 3H), 1.62 (d, *J* = 5.2 Hz, 1H), 1.35 (t, *J* = 7.1 Hz, 3H).

^{13}C NMR (126 MHz, $CDCl_3$): δ_C [ppm] = 171.6, 169.2, 159.5, 141.6, 131.1, 127.5, 116.1, 113.7, 61.8, 55.3, 52.9, 42.8, 41.7, 28.5, 15.9, 14.3.

HRMS (ESI^+) calc. for $C_{18}H_{22}NaO_5S$ $[M+Na]^+$: 373.1080, found: 373.1096.

1-Ethyl 2-methyl 1-(methylthio)-2-(1-(4-(trifluoromethyl)phenyl)vinyl)cyclopropane-1,2-dicarboxylate (3c)

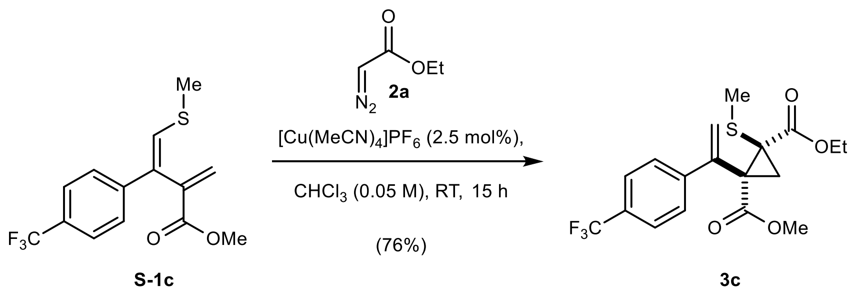

Following GP1, 1,3-diene **S-1c** (60.5 mg, 0.200 mmol, 1.00 equiv) was converted into VCP **3c**. Purification by flash-column chromatography on silica gel (10%→20% MTBE in PE + 2% Et_3N) furnished VCP **3c** (58.7 mg, 0.151 mmol, 76%) as a colorless oil.

TLC (20% MTBE in PE): R_f = 0.26 (UV, CAM).

^{1}H NMR (500 MHz, $CDCl_3$): δ_H [ppm] = 7.79 – 7.75 (m, 2H), 7.60 (d, *J* = 8.2 Hz, 2H), 6.00 (s, 1H), 5.45 (s, 1H), 4.33 – 4.21 (m, 2H), 3.60 (s, 3H), 2.45 (d, *J* = 5.3 Hz, 1H), 2.09 (s, 3H), 1.62 (d, *J* = 5.3 Hz, 1H), 1.35 (t, *J* = 7.1 Hz, 3H).

^{13}C NMR (126 MHz, $CDCl_3$): δ_C [ppm] = 171.1, 168.9, 142.1, 141.3, 129.8 (q, *J* = 32.4 Hz), 126.5, 125.3 (q, *J* = 3.5 Hz), 124.3 (q, *J* = 271.9 Hz),120.3, 62.0, 53.0, 42.4, 41.8, 28.3, 15.9, 14.2 (q, *J* = 2.4 Hz).

^{19}F NMR (376 MHz, $CDCl_3$): δ_F [ppm] = −62.43 (s).

HRMS (ESI^+) calc. for $C_{18}H_{19}F_3NaO_4S$ $[M+Na]^+$: 411.0848, found: 411.0849.

2-Ethyl 1-methyl 1-(1-(4-bromophenyl)vinyl)-2-(methylthio)cyclopropane-1,2-dicarboxylate (3d)

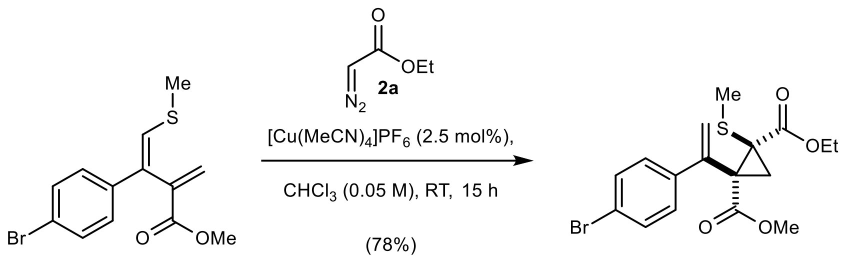

Following GP1, 1,3-diene **S-1d** (62.6 mg, 0.200 mmol, 1.00 equiv) was converted into VCP **3d**. Purification by flash-column chromatography on silica gel (10%→20% MTBE in PE + 2% Et_3N) furnished VCP **3d** (62.4 mg, 0.156 mmol, 78%) as a pale-yellow oil.

TLC (20% MTBE in PE): R_f = 0.27 (UV, CAM).

^{1}H NMR (500 MHz, $CDCl_3$): δ_H [ppm] = 7.56 – 7.51 (m, 2H), 7.49 – 7.43 (m, 2H), 5.90 (s, 1H), 5.35 (s, 1H), 4.37 – 4.17 (m, 2H), 3.59 (s, 3H), 2.41 (d, J = 5.3 Hz, 1H), 2.08 (s, 3H), 1.61 (d, J = 5.3 Hz, 1H), 1.34 (t, J = 7.1 Hz, 3H).

^{13}C NMR (126 MHz, $CDCl_3$): δ_C [ppm] = 171.3, 169.0, 141.3, 137.6, 131.5, 127.9, 122.1, 118.6, 61.9, 53.0, 42.4, 41.8, 28.3, 15.9, 14.2.

HRMS (ESI^+) calc. for $C_{17}H_{19}BrNaO_4S$ $[M+Na]^+$: 421.0080, found: 421.0092.

1-Ethyl 2-methyl 2-(1-(4-formylphenyl)vinyl)-1-(methylthio)cyclopropane-1,2-dicarboxylate (3e)

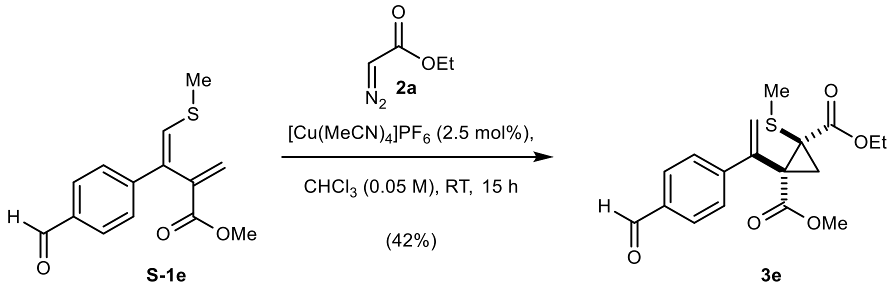

Following GP1, 1,3-diene **S-1e** (52.5 mg, 0.200 mmol, 1.00 equiv) was converted into VCP **3e**. Purification by flash-column chromatography on silica gel (20% MTBE in PE + 2% Et_3N) furnished VCP **3e** (28.9 mg, 82.9 µmol, 42%) as a colorless oil.

TLC (20% MTBE in PE): R_f = 0.31 (UV, CAM).

^{1}H NMR (500 MHz, $CDCl_3$): δ_H [ppm] = 10.01 (s, 1H), 7.91 – 7.82 (m, 4H), 6.07 (s, 1H), 5.50 (s, 1H), 4.34 – 4.22 (m, 2H), 3.60 (s, 3H), 2.46 (d, J = 5.3 Hz, 1H), 2.09 (s, 3H), 1.64 (d, J = 5.3 Hz, 1H), 1.36 (t, J = 7.1 Hz, 3H).

^{13}C NMR (126 MHz, $CDCl_3$): δ_C [ppm] = 192.0, 171.1, 168.9, 144.7, 141.6, 135.7, 129.9, 126.9, 121.1, 62.0, 53.0, 42.4, 41.9, 28.4, 16.0, 14.3.

HRMS (ESI^+) calc. for $C_{18}H_{20}NaO_5S$ $[M+Na]^+$: 371.0924, found: 371.0908.

2-Ethyl 1-methyl 1-(1-(4-(ethoxycarbonyl)phenyl)vinyl)-2-(methylthio)cyclopropane-1,2-dicarboxylate (3f)

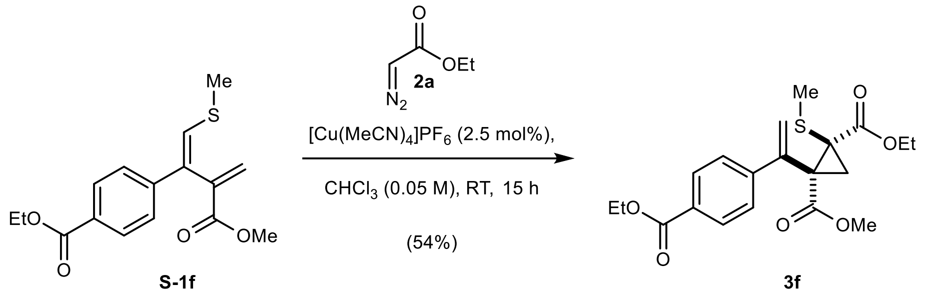

Following GP1, 1,3-diene **S-1f** (61.3 mg, 0.200 mmol, 1.00 equiv) was converted into VCP **3f**. Purification by flash-column chromatography on silica gel (10%→20% MTBE in PE + 2% Et_3N) furnished VCP **3f** (42.0 mg, 0.107 mmol, 54%) as a pale-yellow solid.

TLC (20% MTBE in PE): R_f = 0.21 (UV, CAM).

^{1}H NMR (500 MHz, $CDCl_3$): δ_H [ppm] = 8.08 – 7.98 (m, 2H), 7.78 – 7.70 (m, 2H), 6.02 (s, 1H), 5.45 (s, 1H), 4.37 (q, J = 7.1 Hz, 2H), 4.34 – 4.21 (m, 2H), 3.59 (s, 3H), 2.44 (d, J = 5.3 Hz, 1H), 2.07 (s, 3H), 1.62 (d, J = 5.3 Hz, 1H), 1.39 (t, J = 7.1 Hz, 3H), 1.36 (t, J = 7.2 Hz, 3H).

^{13}C NMR (126 MHz, $CDCl_3$): δ_C [ppm] = 171.3, 168.9, 166.5, 143.0, 141.7, 129.8, 129.7, 126.2, 120.2, 62.0, 61.1, 53.0, 42.5, 41.9, 28.4, 16.0, 14.5, 14.3.

HRMS (ESI$^+$) calc. for $C_{20}H_{24}NaO_6S$ $[M+Na]^+$: 415.1186, found 415.1191.

2-Ethyl 1-methyl 1-(1-(4-(dimethylcarbamoyl)phenyl)vinyl)-2-(methylthio)cyclopropane-1,2-dicarboxylate (3g)

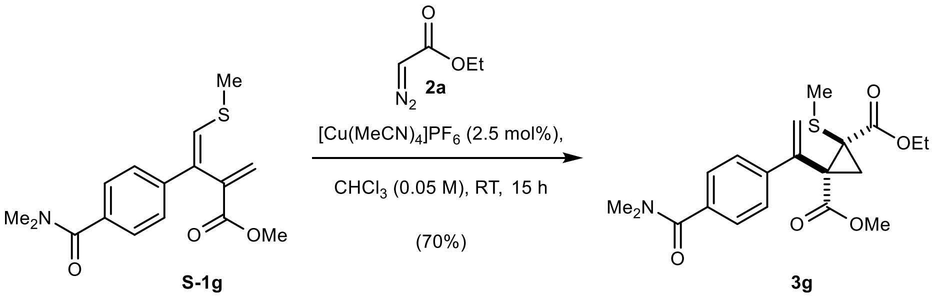

Following GP1, 1,3-diene **S-1g** (61.1 mg, 0.200 mmol, 1.00 equiv) was converted into VCP **3g**. Purification by flash-column chromatography on silica gel (10%→20% MTBE in PE + 2% Et_3N) furnished VCP **3g** (58.0 mg, 0.140 mmol, 70%) as a pale-yellow oil.

TLC (20% EtOAc in PE): R_f = 0.09 (UV, CAM).

^{1}H NMR (500 MHz, $CDCl_3$): δ_H [ppm] = 7.67 (d, J = 8.1 Hz, 2H), 7.40 (d, J = 8.0 Hz, 2H), 5.95 (s, 1H), 5.39 (s, 1H), 4.33 – 4.18 (m, 2H), 3.58 (s, 3H), 3.10 (s, 3H), 2.99 (s, 3H), 2.40 (d, J = 5.3 Hz, 1H), 2.07 (s, 3H), 1.60 (d, J = 5.3 Hz, 1H), 1.33 (t, J = 7.1 Hz, 3H).

^{13}C NMR (126 MHz, $CDCl_3$): δ_C [ppm] = 171.5, 171.3, 169.0, 141.6, 139.9, 135.6, 127.3, 126.2, 119.2, 61.9, 53.0, 42.5, 41.8, 39.8, 35.5, 28.2, 15.9, 14.2.

HRMS (ESI$^+$) calc. for $C_{20}H_{25}NNaO_5S$ $[M+Na]^+$: 414.1346, found: 414.1359.

2-Ethyl 1-methyl 1-(1-(2-chlorophenyl)vinyl)-2-(methylthio)cyclopropane-1,2-dicarboxylate (3h)

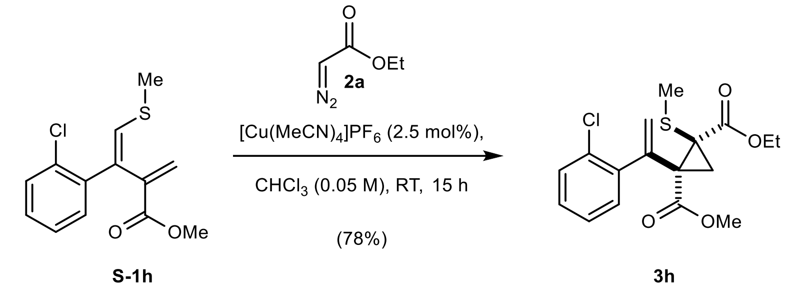

Following GP1, 1,3-diene **S-1h** (53.8 mg, 0.200 mmol, 1.00 equiv) was converted into VCP **3h**. Purification by flash-column chromatography on silica gel (10%→20% MTBE in PE + 2% Et_3N) furnished VCP **3h** (55.4 mg, 0.156 mmol, 78%) as a colorless oil.

TLC (20% MTBE in PE): R_f = 0.62 (UV, CAM).

^{1}H NMR (500 MHz, $CDCl_3$): δ_H [ppm] = 7.38 – 7.32 (m, 2H), 7.25 – 7.17 (m, 2H), 5.88 (s, 1H), 5.79 (s, 1H), 4.23 – 4.15 (m, 2H), 3.71 (s, 3H), 2.39 (d, J = 5.8 Hz, 1H), 2.16 (s, 3H), 1.29 (t, J = 7.1 Hz, 3H), 1.09 (d, J = 5.8 Hz, 1H).

^{13}C NMR (126 MHz, $CDCl_3$): δ_C [ppm] = 170.6, 168.6, 139.8, 139.8, 132.6, 132.0, 129.7, 128.8, 126.8, 126.2, 61.9, 52.9, 43.3, 42.6, 27.2, 14.9, 14.3.

HRMS (ESI$^+$) calc. for $C_{17}H_{19}ClNaO_4S$ $[M+Na]^+$: 377.0585, found: 377.0597.

1-Ethyl 2-methyl 1-(methylthio)-2-(1-(3-nitrophenyl)vinyl)cyclopropane-1,2-dicarboxylate (3i)

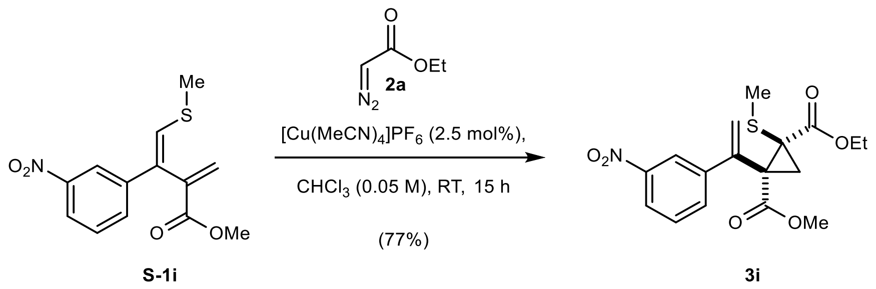

Following GP1, 1,3-diene **S-1i** (55.9 mg, 0.200 mmol, 1.00 equiv) was converted into VCP **3i**. Purification by flash-column chromatography on silica gel (20% EtOAc in PE + 2% Et_3N) furnished VCP **3i** (56.3 mg, 0.154 mmol, 77%) as a pale-yellow solid.

TLC (20% MTBE in PE): R_f = 0.62 (UV, CAM).

^{1}H NMR (500 MHz, $CDCl_3$): δ_H [ppm] 8.57 (d, J = 1.9 Hz, 1H), 8.18 – 8.12 (m, 1H), 8.04 – 7.98 (m, 1H), 7.53 (t, J = 8.0 Hz, 1H), 6.05 (s, 1H), 5.49 (s, 1H), 4.36 – 4.24 (m, 3H), 3.60 (s, 3H), 2.49 (d, J = 5.4 Hz, 1H), 2.10 (s, 3H), 1.66 (d, J = 4.4 Hz, 1H), 1.36 (t, J = 6.4 Hz, 3H).

^{13}C NMR (126 MHz, $CDCl_3$): δ_C [ppm] = 170.8, 168.6, 148.6, 140.8, 140.5, 132.2, 129.3, 122.7, 121.3, 120.6, 62.2, 53.0, 42.2, 42.0, 28.3, 16.1, 14.2.

HRMS (ESI$^+$) calc. for $C_{17}H_{19}NNaO_6S$ $[M+Na]^+$: 388.0825, found: 388.0825.

1-Ethyl 2-methyl 1-(methylthio)-2-(1-(thiophen-2-yl)vinyl)cyclopropane-1,2-dicarboxylate (3j)

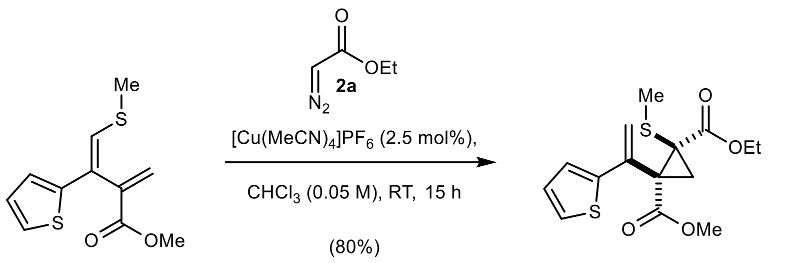

Following GP1, 1,3-diene **S-1j** (48.1 mg, 0.200 mmol, 1.00 equiv) was converted into VCP **3j**. Purification by flash-column chromatography on silica gel (10%→20% MTBE in PE + 2% Et_3N) furnished VCP **3j** (52.0 mg, 0.160 mmol, 80%) as an orange oil.

TLC (20% MTBE in PE): R_f = 0.62 (UV, CAM).

^{1}H NMR (500 MHz, $CDCl_3$): δ_H [ppm] = 7.33 (dd, J = 3.7, 1.2 Hz, 1H), 7.19 (dd, J = 5.2, 1.2 Hz, 1H), 6.99 (dd, J = 5.1, 3.6 Hz, 1H), 5.77 (s, 1H), 5.15 (s, 1H), 4.37 – 4.19 (m, 2H), 3.62 (s, 3H), 2.40 (d, J = 5.2 Hz, 1H), 2.10 (s, 3H), 1.67 (d, J = 5.2 Hz, 1H), 1.34 (t, J = 7.1 Hz, 3H).

^{13}C NMR (126 MHz, $CDCl_3$): δ_C [ppm] = 171.1, 169.0, 143.5, 137.2, 127.7, 126.3, 124.9, 116.4, 61.9, 53.0, 42.9, 41.5, 28.3, 16.0, 14.3.

HRMS (ESI^+) calc. for $C_{15}H_{18}NaO_4S_2$ $[M+Na]^+$: 349.0539 found: 349.0564.

2-Ethyl 1-methyl 1-(1-(1-(*tert*-butoxycarbonyl)-1H-indol-5-yl)vinyl)-2-(methylthio)cyclopropane-1,2-dicarboxylate (3k)

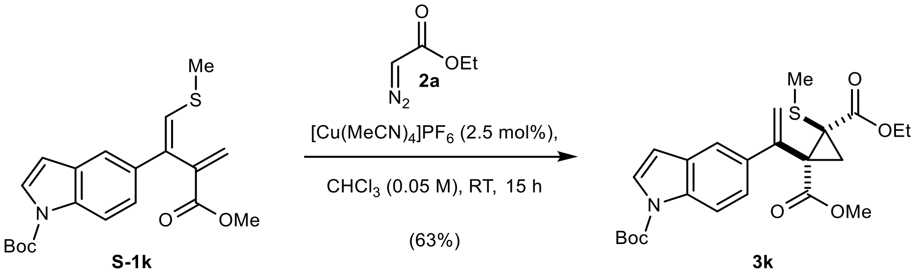

Following GP1, 1,3-diene **S-1k** (83.0 mg, 90%, 0.200 mmol, 1.00 equiv) was converted into VCP **3k**. Purification by flash-column chromatography on silica gel (15% EtOAc in PE + 1% Et_3N) furnished VCP **3k** (58.1 mg, 0.126 mmol, 63%) as a yellow oil.

TLC (20% MTBE in PE): R_f = 0.23 (UV, CAM).

^{1}H NMR (500 MHz, $CDCl_3$): δ_H [ppm] = 8.08 (d, J = 7.5 Hz, 1H), 7.87 (d, J = 1.9 Hz, 1H), 7.63 (dd, J = 8.8, 2.0 Hz, 1H), 7.58 (d, J = 3.7 Hz, 1H), 6.57 (d, J = 3.7 Hz, 1H), 5.95 (s, 1H), 5.34 (s, 1H), 4.36 – 4.22 (m, 2H), 3.60 (s, 3H), 2.42 (d, J = 5.2 Hz, 1H), 2.07 (s, 3H), 1.67 (s, 9H), 1.64 (d, J = 5.2 Hz, 1H), 1.38 (t, J = 7.2 Hz, 3H).

^{13}C NMR (126 MHz, $CDCl_3$): δ_C [ppm] 171.7, 169.2, 142.3, 133.4, 130.7, 126.4, 122.8, 119.0, 117.3, 114.9, 107.9, 83.8, 77.4, 61.8, 52.9, 43.0, 41.8, 28.5, 28.3, 15.9, 14.3, 14.3.

HRMS (ESI^+) calc. for $C_{24}H_{29}NNaO_6S$ $[M+Na]^+$: 482.1608, found: 482.1595.

1-Ethyl 2-methyl -1-(methylthio)-2-(prop-1-en-2-yl)cyclopropane-1,2-dicarboxylate (3l)

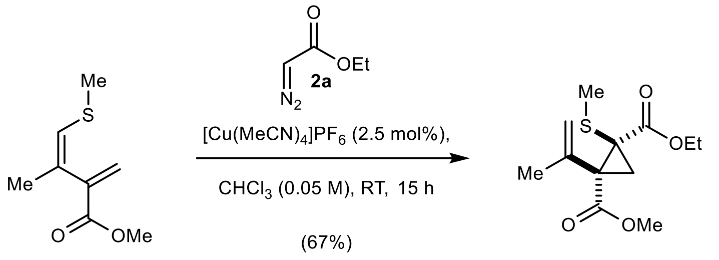

Following GP1, 1,3-diene **S-1l** (37.0 mg, 0.200 mmol, 1.00 equiv) was converted into VCP **3l**. Purification by flash-column chromatography on silica gel (10%→20% MTBE in PE + 2% Et_3N) furnished VCP **3l** (37.2 mg, 0.144 mmol, 67%) as a pale-yellow oil.

TLC (20% MTBE in PE): R_f = 0.62 (CAM).

^{1}H NMR (500 MHz, $CDCl_3$): δ_H [ppm] = 5.17 (s, 1H), 5.02 (s, 1H), 4.22 – 4.13 (m, 2H), 3.66 (s, 3H), 2.27 (d, J = 5.2 Hz, 2H), 2.19 (s, 3H), 1.97 (t, J = 1.1 Hz, 3H), 1.53 (d, J = 5.2 Hz, 2H), 1.27 (t, J = 7.1 Hz, 11H).

^{13}C NMR (126 MHz, $CDCl_3$): δ_C [ppm] = 170.6, 169.3, 140.1, 118.2, 61.8, 52.7, 45.2, 40.5, 27.4, 27.0, 22.4, 16.0.

HRMS (ESI$^+$) calc. for $C_{12}H_{19}O_4S$ $[M+H]^+$: 259.0999, found: 259.1010.

1-Ethyl 2-methyl 1-(methylthio)-2-(1-((triisopropylsilyl)oxy)vinyl)cyclopropane-1,2-dicarboxylate (3m)

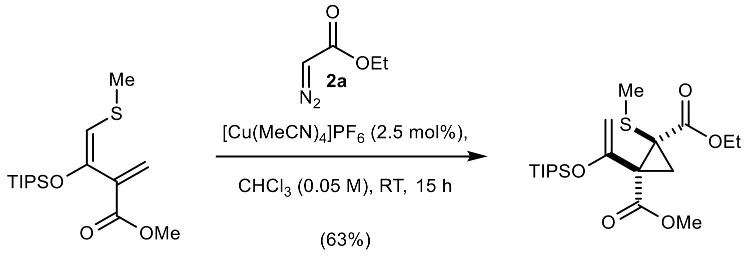

Following GP1, 1,3-diene **S-1m** (66.1 mg, 0.200 mmol, 1.00 equiv) was converted into VCP **3m**. Purification by double flash-column chromatography on silica gel (first: 3% EtOAc in PE + 1% Et_3N, second: 3% MTBE in PE + 1% Et_3N) furnished VCP **3m** (52.5 mg, 0.126 mmol, 63%) as a pale-yellow oil.

TLC (5% MTBE in PE): R_f = 0.58 (UV, CAM).

^{1}H NMR (500 MHz, $CDCl_3$): δ_H [ppm] =4.35 (d, J = 1.9 Hz, 1H), 4.29 (d, J = 1.8 Hz, 1H), 4.22 – 4.09 (m, 2H), 3.65 (s, 3H), 2.22 (s, 3H), 2.20 (d, J = 5.3 Hz, 1H), 1.56 (d, J = 5.3 Hz, 1H), 1.30 – 1.20 (m, 6H), 1.11 (dd, J = 7.4, 3.1 Hz, 18H).

^{13}C NMR (126 MHz, $CDCl_3$): δ_C [ppm] = 170.4, 169.1, 153.3, 93.9, 61.7, 52.4, 44.6, 40.6, 27.2, 18.2, 18.2, 15.9, 14.2, 12.7.

HRMS (ESI$^+$) calc. for $C_{20}H_{36}NaO_5SSi$ $[M+Na]^+$: 439.1945, found: 439.1947.

2-Ethyl 1-methyl 1-acetyl-2-(methylthio)cyclopropane-1,2-dicarboxylate (3n)

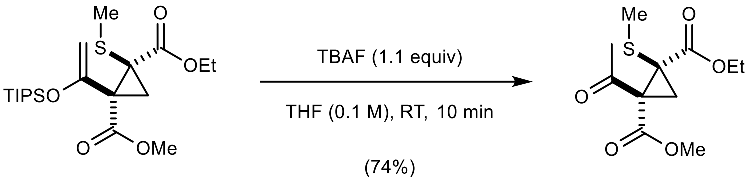

3m **3n**

To a solution of VCP **3m** (34.5 mg, 82.8 µmol, 1.00 equiv) in THF (0.8 mL, 0.1 M), tetra-*n*-butylammonium fluoride (TBAF, 27 µL, 0.091, 1.1 equiv, 1 M in THF) was added at RT leading to an orange reaction mixture. After 10 minutes, saturated aqueous $NaHCO_3$ (1 mL) solution and EtOAc (1 mL) were added. The layers were separated and the aqueous layer was extracted using EtOAc (3 ×1.5 mL). The combined organic layers were dried over Na_2SO_4. The dried solution was filtered, and the filtrate was concentrated under reduced pressure. Purification by double flash-column chromatography on silica gel (first: 10% EtOAc in PE + 1% Et_3N, second: 5% EtOAc in PE + 1% Et_3N) furnished cyclopropane **3n** (15.9 mg, 61.1 µmol, 74%) as a colorless oil.

TLC (5% MTBE in PE): R_f = 0.17 (UV, CAM).

^{1}H NMR (500 MHz, $CDCl_3$): δ_H [ppm] = 4.21 (qd, *J* = 7.1, 2.1 Hz, 2H), 3.72 (s, 3H), 2.45 (s, 3H), 2.20 (s, 3H), 2.16 (d, *J* = 5.7 Hz, 1H), 1.91 (d, *J* = 5.7 Hz, 1H), 1.29 (t, *J* = 7.2 Hz, 3H).

^{13}C NMR (126 MHz, $CDCl_3$): δ_C [ppm] = 197.9, 168.2, 167.8, 62.2, 53.0, 48.4, 40.3, 30.5, 24.4, 16.1, 14.2.

HRMS (ESI$^+$) calc. for $C_{11}H_{16}NaO_5S$ $[M+Na]^+$: 283.0611, found: 283.0618.

1-Ethyl 2-methyl 1-(methylthio)-2-((*E*)-1-phenylprop-1-en-1-yl)cyclopropane-1,2-dicarboxylate (3o)

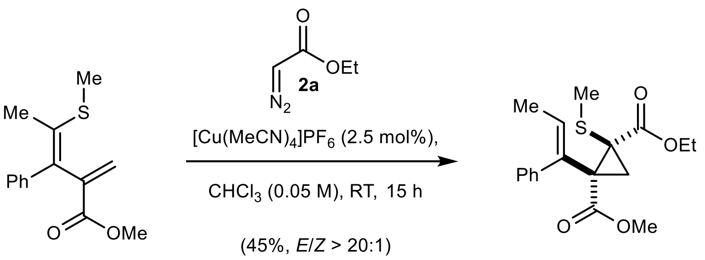

S-1o **3o**

Following GP1, 1,3-diene **S-1o** (55.2 mg, 90%, 0.200 mmol, 1.00 equiv) was converted into VCP **3o**. Purification by flash-column chromatography on silica gel (first: 10%→20% MTBE in PE + 1% Et_3N, second: 20% PhMe in PE + 1% Et_3N) furnished VCP **3o** (30.0 mg, 89.8 µmol, 45%) as a yellow oil and single diastereomer.

TLC (20% MTBE in PE): R_f = 0.21 (UV, CAM).

^{1}H NMR (500 MHz, $CDCl_3$): δ_H [ppm] = 7.37 – 7.21 (m, 5H), 6.09 (q, *J* = 7.1 Hz, 1H), 4.28 – 4.12 (m, 2H), 3.68 (s, 3H), 2.24 (d, *J* = 5.3 Hz, 1H), 2.09 (s, 3H), 1.78 (d, *J* = 7.1 Hz, 3H), 1.29 (t, *J* = 7.1 Hz, 3H), 1.23 (d, *J* = 5.4 Hz, 1H).

^{13}C NMR (126 MHz, $CDCl_3$): δ_C [ppm] = 171.7, 169.4, 138.9, 134.0, 131.4, 129.6, 128.0, 126.9, 61.9, 52.8, 45.4, 42.1, 28.3, 15.6, 15.3, 14.3.

HRMS (ESI^+) calc. for $C_{18}H_{22}NaO_4S$ $[M+Na]^+$: 357.1131, found: 357.1117.

Ethyl 2-acetyl-1-(methylthio)-2-(1-phenylvinyl)cyclopropane-1-carboxylate (4a)

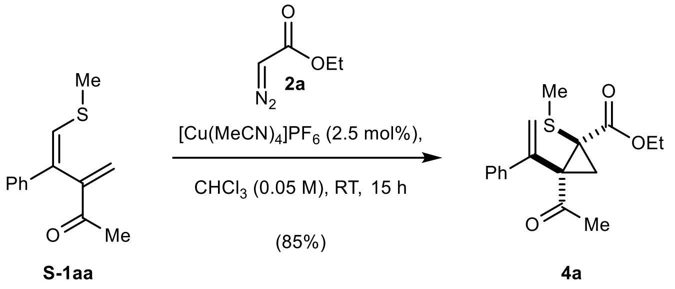

Following GP1, 1,3-diene **S-1aa** (43.7 mg, 0.200 mmol, 1.00 equiv) was converted into VCP **4a**. Purification by flash-column chromatography on silica gel (first: 10%→20% MTBE in PE, second: 20% PhMe in PE + 1% Et_3N) furnished VCP **4a** (51.8 mg, 0.170 mmol, 85%) as a yellow oil.

TLC (20% MTBE in PE): R_f = 0.30 (UV, CAM).

^{1}H NMR (500 MHz, $CDCl_3$): δ_H [ppm] = 7.71 – 7.66 (m, 2H), 7.42 – 7.36 (m, 2H), 7.35 – 7.29 (m, 1H), 6.08 (s, 1H), 5.42 (s, 1H), 4.35 – 4.19 (m, 2H), 2.27 (d, J = 4.9 Hz, 1H), 2.15 (s, 3H), 2.08 (s, 3H), 1.60 (d, J = 4.8 Hz, 1H), 1.35 (t, J = 7.1 Hz, 3H).

^{13}C NMR (126 MHz, $CDCl_3$): δ_C [ppm] = 205.8, 169.5, 143.1, 138.1, 128.8, 128.5, 126.2, 118.6, 61.7, 49.7, 43.0, 30.2, 29.1, 15.7, 14.2.

HRMS (ESI^+) calc. for $C_{17}H_{20}NaO_3S$ $[M+Na]^+$: 327.1025, found: 327.1023.

Ethyl 2-(methoxy(methyl)carbamoyl)-1-(methylthio)-2-(1-phenylvinyl)cyclopropane-1-carboxylate (4b)

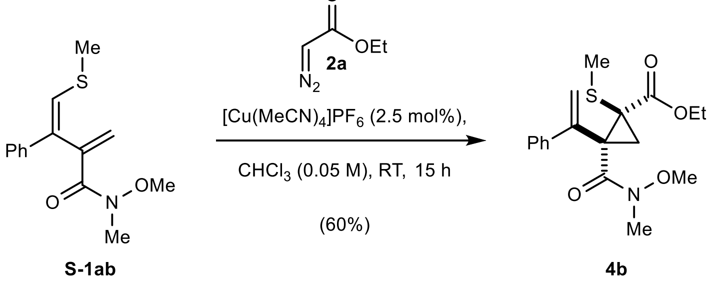

Following GP1, 1,3-diene **S-1ab** (52.7 mg, 0.200 mmol, 1.00 equiv) was converted into VCP **4b**. Purification by flash-column chromatography on silica gel (20% EtOAc + 20% PhMe in *n*-pentane + 2% Et_3N) furnished VCP **4b** (41.6 mg, 0.119 mmol, 60%) as a pale-yellow oil.

TLC (20% EtOAc, 20% PhMe in *n*-pentane): R_f = 0.18 (UV, CAM).

^{1}H NMR (500 MHz, $CDCl_3$): δ_H [ppm] = 7.64 – 7.62 (m, 1H), 7.40 – 7.10 (m, 4H), 5.79 (s, 1H), 5.42 (s, 1H), 4.37 – 4.22 (m, 2H), 3.58 (s, 3H), 3.08 (s, 3H), 2.43 (d, *J* = 5.6 Hz, 1H), 2.13 (s, 3H), 1.74 (d, *J* = 5.6 Hz, 1H), 1.36 (t, *J* = 7.1 Hz, 3H).

^{13}C NMR (126 MHz, $CDCl_3$): δ_C [ppm] = 170.9, 142.8, 138.6, 129.1, 128.3, 128.2, 128.1, 127.7, 125.4, 118.3, 62.3, 60.6, 45.9, 29.8, 15.3, 14.3.

HRMS (ESI^+) calc. for $C_{18}H_{23}NNaO_4S$ $[M+Na]^+$: 372.1240, found: 372.1238.

1-(Methylthio)-5-phenyl-3-azabicyclo[3.1.0]hexane-2,4-dione (4d)

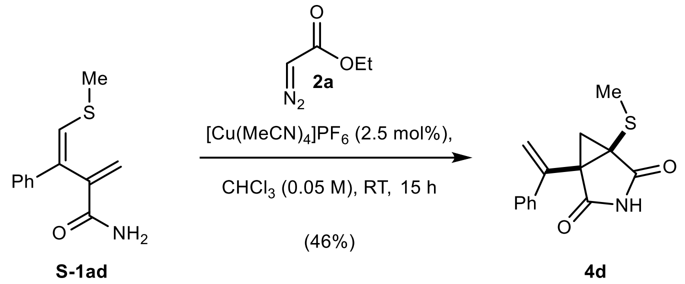

Following GP1, 1,3-diene **S-1ad** (43.9 mg, 0.200 mmol, 1.00 equiv) was converted into VCP **4d**. Purification by double flash-column chromatography on silica gel (first: 5% MTBE in CH_2Cl_2 + 1% Et_3N; second: 2% EtOAc in CH_2Cl_2 + 1% Et_3N) furnished VCP **4d** (23.9 mg, 92.2 µmol, 46%) as an orange-brown oil.

TLC (50% EtOAc in PE): R_f = 0.28 (UV, CAM).

^{1}H NMR (700 MHz, $CDCl_3$): δ_H [ppm] = 7.40 – 7.30 (m, 5H), 5.92 (s, 1H), 5.45 (s, 1H), 2.25 (d, *J* = 4.8 Hz, 1H), 2.16 (s, 3H), 1.99 (d, *J* = 4.9 Hz, 1H).

^{13}C NMR (176 MHz, $CDCl_3$): δ_C [ppm] = 173.8, 138.6, 138.2, 128.9, 128.6, 126.3, 121.9, 45.6, 40.4, 33.2, 15.9.

HRMS (ESI^+) calc. for $C_{14}H_{13}NNaO_2S$ $[M+Na]^+$: 282.0559, found: 282.0559.

3-Methyl-1-(methylthio)-5-phenyl-3-azabicyclo[3.1.0]hexane-2,4-dione (4e)

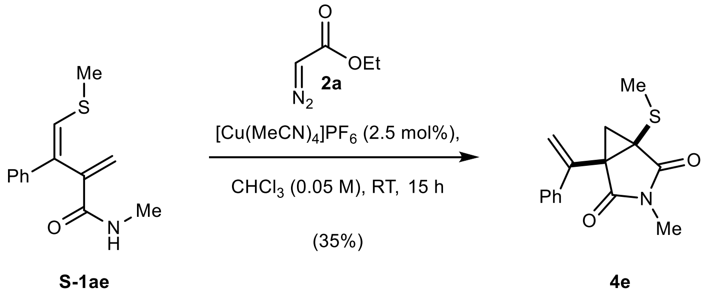

Following GP1, 1,3-diene **S-1ae** (47.0 mg, 0.200 mmol, 1.00 equiv) was converted into VCP **4e**. Purification by flash-column chromatography on silica gel (first: 40% EtOAc in PE + 1% Et_3N, second: 10% EtOAc + 10% CH_2Cl_2 in PE + 1% Et_3N) furnished VCP **4e** (19.3 mg, 70.2 µmol, 35%) as an orange solid.

TLC (50% EtOAc in PE): $R_f = 0.57$ (UV, CAM).

^{1}H NMR (500 MHz, $CDCl_3$): δ_H [ppm] = 7.36 – 7.29 (m, 5H), 5.89 (s, 1H), 5.37 (s, 1H), 3.95 (s, 3H), 2.20 (d, J = 4.3 Hz, 1H), 2.19 (s, 3H), 1.96 (d, J = 4.4 Hz, 1H).

^{13}C NMR (126 MHz, $CDCl_3$): δ_C [ppm] = 193.4, 186.3, 138.5, 138.0, 128.9, 128.6, 126.0, 120.3, 58.8, 46.4, 44.5, 38.2, 15.6.

HRMS (ESI$^+$) calc. for $C_{15}H_{15}NNaO_2S$ $[M+Na]^+$: 296.0716, found: 296.0712.

1-(Methylthio)-5-phenyl-3-oxabicyclo[3.1.0]hexan-2-one (4g)

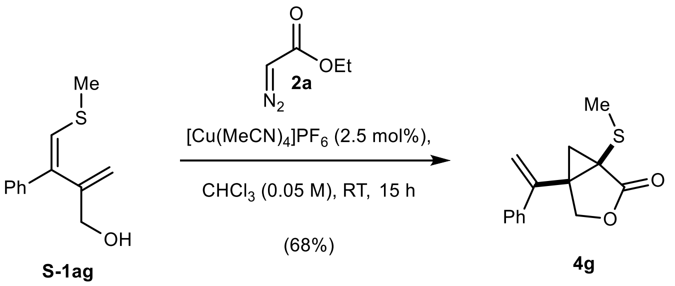

Following GP1, 1,3-diene **S-1ag** (41.3 mg, 0.200 mmol, 1.0 equiv) was converted into VCP **4g**. Purification by flash-column chromatography on silica gel (10% MTBE in PE + 2% Et_3N) furnished VCP **4g** (33.7 mg, 0.137 mmol, 68%) as a colorless oil.

TLC (20% EtOAc in CyH): $R_f = 0.44$ (UV, CAM).

^{1}H NMR (500 MHz, $CDCl_3$): δ_H [ppm] = 7.40 – 7.30 (m, 5H), 5.75 (s, 1H), 5.41 (s, 1H), 4.26 – 4.22 (m, 2H), 2.35 (s, 3H), 1.84 (d, J = 4.9 Hz, 1H), 1.66 (d, J = 4.9 Hz, 1H).

^{13}C NMR (126 MHz, $CDCl_3$): δ_C [ppm] = 175.1, 141.9, 137.8, 128.9, 128.6, 126.7, 119.4, 71.8, 42.6, 37.2, 25.5, 15.4.

HRMS (ESI$^+$) calc. for $C_{14}H_{14}NaO_2S$ $[M+Na]^+$: 269.0607, found: 269.0605.

Ethyl 2-(methoxymethyl)-1-(methylthio)-2-(1-phenylvinyl)cyclopropane-1-carboxylate (4h)

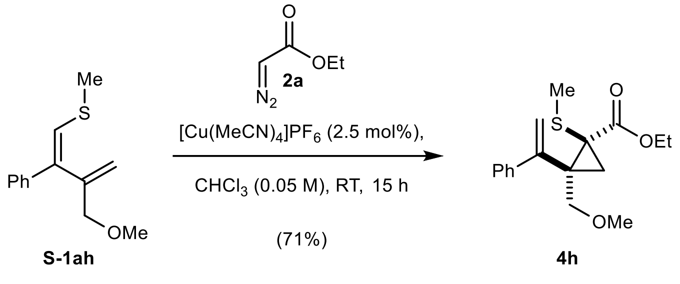

Following GP1, 1,3-diene **S-1ah** (44.1 mg, 0.200 mmol, 1.00 equiv) was converted into VCP **4h**. Purification by flash-column chromatography on silica gel (5% PhMe + 10% EtOAc in *n*-pentane + 2% Et_3N) furnished VCP **4h** (43.4 mg, 0.142 mmol, 71%) as a colorless oil.

TLC (20% EtOAc in CyH): $R_f = 0.44$ (UV, CAM).

^{1}H NMR (500 MHz, $CDCl_3$): δ_H [ppm] = 7.65 – 7.59 (m, 2H), 7.39 – 7.33 (m, 2H), 7.31 – 7.26 (m, 1H), 5.72 (s, 1H), 5.30 (s, 1H), 4.34 – 4.21 (m, 2H), 3.66 (d, J = 9.8 Hz, 1H), 3.22 (d, J = 9.9 Hz, 1H), 3.18 (s, 3H), 2.12 (s, 3H), 1.98 (d, J = 5.5 Hz, 1H), 1.38 (d, J = 5.5 Hz, 1H), 1.36 (t, J = 7.1 Hz, 3H) .

^{13}C NMR (126 MHz, $CDCl_3$): δ_C [ppm] = 170.4, 145.1, 138.7, 128.4, 127.8, 127.1, 117.4, 74.8, 61.7, 58.9, 39.9, 37.9, 23.5, 15.6, 14.4.

HRMS (ESI^+) calc. for $C_{17}H_{22}NaO_3S$ $[M+Na]^+$: 329.1182, found: 329.1182.

Ethyl 7-(methylthio)-2-oxo-1-vinylbicyclo[4.1.0]heptane-7-carboxylate (4i)

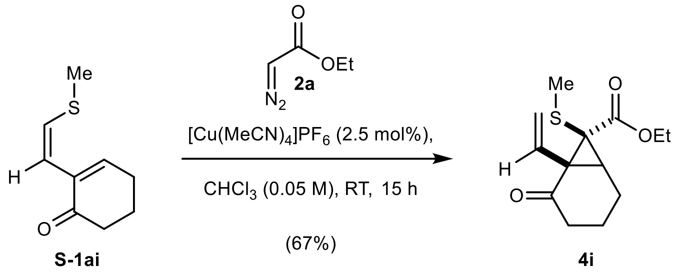

Following GP1, 1,3-diene **S-1ai** (33.7 mg, 0.200 mmol, 1.00 equiv) was converted into VCP **4i**. Purification by flash-column chromatography on silica gel (10%→20% MTBE in PE + 1% Et_3N) furnished VCP **4i** (34.2 mg, 0.134 mmol, 67%) as a colorless oil.

TLC (20% MTBE in PE): R_f = 0.36 (CAM).

^{1}H NMR (700 MHz, $CDCl_3$): δ_H [ppm] = 6.40 (ddd, J = 17.3, 10.6, 0.6 Hz, 1H), 5.31 (dd, J = 10.5, 1.0 Hz, 1H), 5.17 (dd, J = 17.3, 1.0 Hz, 1H), 4.21 (q, J = 7.1 Hz, 2H), 2.23 –2.20 (m, 2H), 2.14 – 2.11 (m, 2H), 2.10 (s, 3H), 2.06 – 2.01 (m, 1H), 1.77 – 1.68 (m, 1H), 1.65 – 1.58 (m, 1H), 1.30 (t, J = 7.1 Hz, 3H).

^{13}C NMR (176 MHz, $CDCl_3$): δ_C [ppm] = 204.5, 168.6, 133.3, 118.4, 62.2, 44.5, 43.2, 38.6, 33.6, 20.9, 19.7, 15.8, 14.2.

HRMS (ESI^+) calc. for $C_{13}H_{18}NaO_3S$ $[M+Na]^+$: 277.0869, found: 277.0866.

Ethyl 7-(methylthio)-2-oxo-1-(prop-1-en-2-yl)bicyclo[4.1.0]heptane-7-carboxylate (4j)

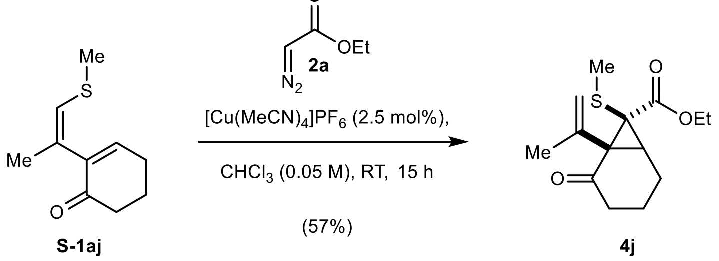

Following GP1, 1,3-diene **S-1aj** (36.5 mg, 0.200 mmol, 1.00 equiv) was converted into VCP **4j**. Purification by flash-column chromatography on silica gel (10%→20% MTBE in PE + 1% Et_3N) furnished VCP **4j** (30.8 mg, 0.115 mmol, 57%) as a pale-yellow oil.

TLC (20% MTBE in PE): R_f = 0.30 (UV, CAM).

^{1}H NMR (500 MHz, $CDCl_3$): δ_H [ppm] = 5.10 – 5.07 (m, 1H), 4.92 – 4.87 (m, 1H), 4.20 (q, J = 7.1 Hz, 2H), 2.26 (s, 3H), 2.25 – 2.20 (m, 1H), 2.20 – 2.16 (m, 1H), 2.16 – 2.12 (m, 1H), 2.10 (dd, J = 7.8, 3.2 Hz, 1H), 2.07 – 2.05 (m, 3H), 2.01 – 1.94 (m, 1H), 1.78 – 1.63 (m, 2H), 1.29 (t, J = 7.1 Hz, 3H).

^{13}C NMR (126 MHz, $CDCl_3$): δ_C [ppm] = 203.9, 168.8, 143.8, 116.8, 62.1, 48.1, 42.2, 38.6, 37.2, 23.1, 21.8, 20.1, 16.7, 14.1.

HRMS (ESI$^+$) calc. for $C_{14}H_{20}NaO_3S$ $[M+Na]^+$: 291.1025, found: 291.1024.

1-(*Tert*-butyl) 2-methyl 1-(methylthio)-2-(1-phenylvinyl)cyclopropane-1,2-dicarboxylate (5a)

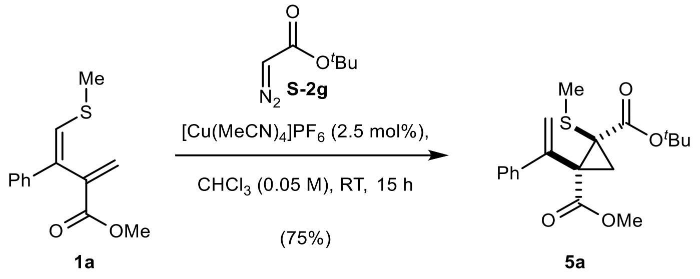

Following GP1, 1,3-diene **1a** (46.9 mg, 0.200 mmol, 1.00 equiv) was converted into VCP **5a** using *tert*-butyl diazo acetate (**S-2g**, 84 µL, 0.60 mmol, 3.0 equiv). Purification by flash-column chromatography on silica gel (10%→20% MTBE in PE + 1% Et_3N) furnished VCP **5a** (52.3 mg, 0.150 mmol, 75%) as a pale-yellow oil.

TLC (20% MTBE in PE): R_f = 0.49 (UV, CAM).

^{1}H NMR (500 MHz, $CDCl_3$): δ_H [ppm] = 7.71 – 7.66 (m, 2H), 7.36 – 7.31 (m, 2H), 7.31 – 7.25 (m, 1H), 5.92 (s, 1H), 5.33 (s, 1H), 3.61 (s, 3H), 2.38 (d, J = 5.0 Hz, 1H), 2.07 (s, 3H), 1.57 (d, J = 5.2 Hz, 1H), 1.54 (s, 9H).

^{13}C NMR (126 MHz, $CDCl_3$): δ_C [ppm] = 171.5, 167.7, 142.5, 138.6, 128.2, 127.9, 126.3, 117.8, 81.7, 52.8, 42.9, 42.5, 28.3, 27.9, 15.9.

HRMS (ESI$^+$) calc. for $C_{19}H_{25}O_4S$ $[M+H]^+$: 349.1468, found: 349.1472.

1-Benzyl 2-methyl 1-(methylthio)-2-(1-phenylvinyl)cyclopropane-1,2-dicarboxylate (5b)

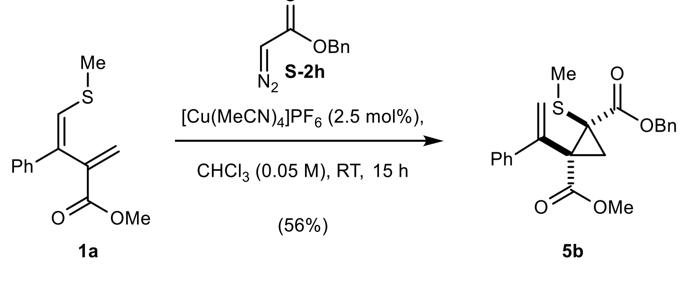

Following GP1, 1,3-diene **1a** (46.9 mg, 0.200 mmol, 1.00 equiv) was converted into VCP **5b** using benzyl diazo acetate (**S-2h**, 1.18 mL, 0.600 mmol, 3.00 equiv, 10% in PhMe). Purification by flash-column chromatography on silica gel (first: PhMe + 1% Et_3N; second: 10%→ 20% EtOAc in CyH +

1% Et_3N) furnished VCP **5b** (42.8 mg, 0.112 mmol, 56%) as a colorless oil. Recrystallization from *n*-pentane and CH_2Cl_2 gave crystals suitable for X-ray analysis (CCDC-2496555).

TLC (20% MTBE in PE): R_f = 0.49 (UV, CAM).

^{1}H NMR (500 MHz, $CDCl_3$): δ_H [ppm] = 7.65 – 7.61 (dd, *J* = 7.7, 2.0 Hz, 2H), 7.51 – 7.34 (m, 5H), 7.34 – 7.21 (m, 3H), 5.94 (s, 1H), 5.35 (s, 1H), 5.31 – 5.15 (m, 2H), 3.50 (s, 3H), 2.44 (d, *J* = 5.3 Hz, 1H), 2.05 (s, 3H), 1.65 (d, *J* = 5.3 Hz, 1H).

^{13}C NMR (126 MHz, $CDCl_3$): δ_C [ppm] = 171.5, 169.0, 142.2, 138.6, 135.9, 128.7, 128.4, 128.3, 128.0, 126.3, 118.2, 67.6, 52.9, 42.8, 41.8, 28.5, 15.9.

HRMS (ESI$^+$) calc. for $C_{22}H_{22}NaO_4S$ $[M+Na]^+$: 405.1131, found: 405.1127.

Methyl 2-acetyl-2-(methylthio)-1-(1-phenylvinyl)cyclopropane-1-carboxylate (5c)

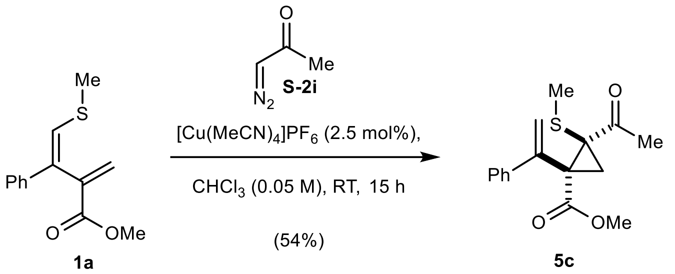

Following GP1, 1,3-diene **1a** (46.9 mg, 0.200 mmol, 1.00 equiv) was converted into VCP **5c** using diazo acetone (**S-2i**, 5.2 mL, 0.60 mmol, 3.0 equiv, 0.17 M in $CHCl_3$,). Purification by flash-column chromatography on silica gel (20% MTBE in PE + 1% Et_3N) furnished VCP **5c** (31.4 mg, 0.108 mmol, 54%) as a pale-yellow oil.

TLC (20% MTBE in PE): R_f = 0.35 (UV, CAM).

^{1}H NMR (500 MHz, $CDCl_3$): δ_H [ppm] = 7.53 – 7.50 (m, 2H), 7.37 – 7.34 (m, 2H), 7.31 – 7.28 (m, 1H), 5.86 (s, 1H), 5.41 (s, 1H), 3.65 (s, 3H), 2.47 (s, 3H), 2.38 (d, *J* = 5.2 Hz, 1H), 1.89 (s, 3H), 1.48 (d, *J* = 5.2 Hz, 1H).

^{13}C NMR (126 MHz, $CDCl_3$): δ_C [ppm] = 200.6, 171.6, 142.6, 139.4, 128.4, 128.0, 126.6, 119.5, 53.1, 48.0, 44.3, 27.6, 27.5, 15.3.

HRMS (ESI$^+$) calc. for $C_{16}H_{18}NaO_3S$ $[M+Na]^+$: 313.0869, found: 313.0861.

Methyl 2-benzoyl-2-(methylthio)-1-(1-phenylvinyl)cyclopropane-1-carboxylate (5d)

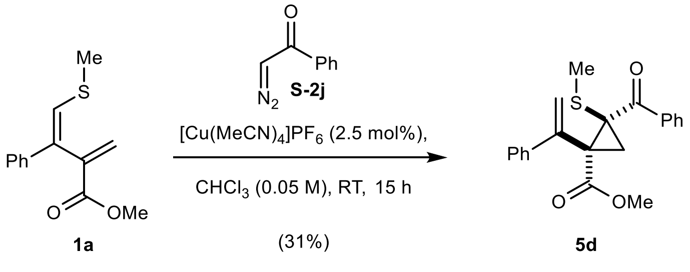

Following GP1, 1,3-diene **1a** (47.0 mg, 0.200 mmol, 1.00 equiv) was converted into VCP **5d** using 2-diazo-1-phenylethan-1-one (**S-2j**, 88 mg, 0.60 mmol, 3.0 equiv). Purification by flash-column chromatography on silica gel (first: 5% MTBE + 20% PhMe in PE; second: 0%→1.5% EtOAc in PE+ 1% Et_3N) furnished VCP **5d** (21.9 mg, 62.1 µmol, 31%) as a yellow oil.

TLC (20% MTBE in PE): $R_f = 0.23$ (UV, CAM).

^{1}H NMR (500 MHz, $CDCl_3$): δ_H [ppm] = 8.12 – 8.07 (m, 2H), 7.65 – 7.61 (m, 2H), 7.60 – 7.52 (m, 1H), 7.49 – 7.42 (m, 2H), 7.40 – 7.35 (m, 2H), 7.34 – 7.28 (m, 1H), 5.96 (s, 1H), 5.54 (s, 1H), 3.50 (s, 3H), 2.54 (d, J = 5.4 Hz, 1H), 1.78 (s, 3H), 1.67 (d, J = 5.3 Hz, 1H).

^{13}C NMR (126 MHz, $CDCl_3$): δ_C [ppm] = 191.3, 171.8, 142.9, 140.0, 135.3, 133.0, 129.4, 128.5, 128.3, 127.9, 127.3, 120.0, 52.8, 45.3, 42.5, 28.9, 15.4.

HRMS (ESI^+) calc. for $C_{21}H_{20}NaO_3S$ $[M+Na]^+$: 375.1025, found: 375.1028.

Note: Due to thermal instability of VCP **5a**, concentration under reduced pressure was performed at ambient temperature (bath temperature: 22 °C).

Methyl 2-(methylthio)-1-(1-phenylvinyl)cyclopropane-1-carboxylate (5e)

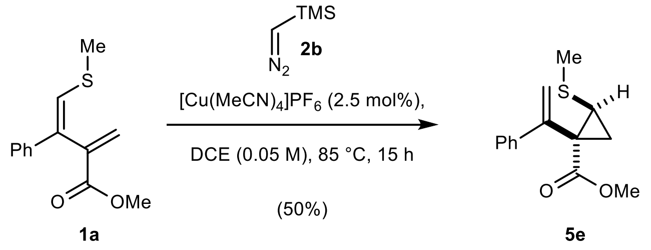

In a 20 mL reaction vial equipped with a magnetic stir bar and a red rubber septum PTFE screw cap, 1,3-diene **1a** (46.9 mg, 0.200 mmol, 1.00 equiv) $[Cu(MeCN)_4]PF_6$ (1.86 mg, 5.00 µmol, 2.5 mol%) were placed, the atmosphere was changed to argon (three cycles) and dry DCE (1.0 mL, 0.2 M) was added. A solution of the tetramethylsilyl diazomethane (**2b**, 0.30 mL, 0.60 mmol, 3.0 equiv, 2 M in hexanes) in DCE (3 mL) was added at RT. The mixture was heated to 85 °C. After 15 hours, the reaction mixture was allowed to cool to RT and was filtered over a plug of deactivated silica eluted with MTBE containing 10% of Et_3N. The filtrate was concentrated under reduced pressure and purification by flash-column chromatography on silica gel (0%→3% MTBE in PE + 2% Et_3N) furnished VCP **5e** (24.6 mg, 98.9 µmol, 50%) as a pale-yellow oil.

TLC (20% MTBE in PE): $R_f = 0.35$ (UV, CAM).

^{1}H NMR (500 MHz, $CDCl_3$): δ_H [ppm] = 7.49 – 7.45 (m, 2H), 7.35 – 7.30 (m, 2H), 7.29 – 7.26 (m, 1H), 5.90 (s, 1H), 5.32 (s, 1H), 3.63 (s, 3H), 3.08 (dd, J = 8.5, 6.2 Hz, 1H), 2.00 (s, 3H), 1.85 (dd, J = 8.5, 4.7 Hz, 1H), 1.28 (dd, J = 6.2, 4.7 Hz, 1H).

^{13}C NMR (126 MHz, $CDCl_3$): δ_C [ppm] = 173.9, 142.4, 139.6, 128.4, 127.8, 126.2, 118.4, 52.8, 36.6, 31.4, 24.7, 16.7.

HRMS (ESI^+) calc. for $C_{14}H_{16}NaO_2S$ $[M+Na]^+$: 271.0763, found: 271.0767.

1-Ethyl 2-methyl 1-(ethylthio)-2-(1-phenylvinyl)cyclopropane-1,2-dicarboxylate (6a)

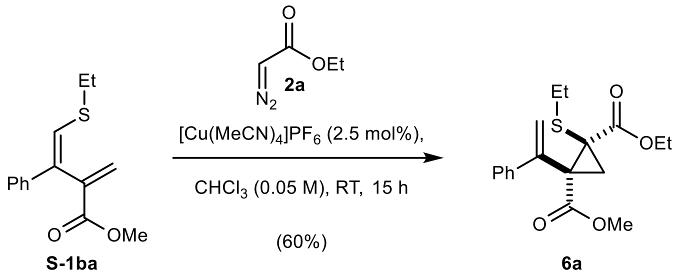

Following GP1, 1,3-diene **S-1ba** (49.7 mg, 0.200 mmol, 1.00 equiv) was converted into VCP **6a**. Purification by flash-column chromatography on silica gel (10% MTBE in PE + 1% Et_3N) furnished VCP **6a** (40.0 mg, 0.120 mmol, 60%) as a colorless oil.

TLC (20% MTBE in PE): R_f = 0.32 (UV, CAM).

^{1}H NMR (500 MHz, $CDCl_3$): δ_H [ppm] = 7.68 – 7.62 (m, 2H), 7.38 – 7.33 (m, 2H), 7.31 – 7.26 (m, 1H), 5.92 (s, 1H), 5.34 (s, 1H), 4.34 – 4.19 (m, 2H), 3.59 (s, 3H), 2.58 (q, J = 7.5 Hz, 2H), 2.46 (d, J = 5.3 Hz, 1H), 1.65 (d, J = 5.2 Hz, 1H), 1.35 (t, J = 7.1 Hz, 3H), 1.15 (t, J = 7.5 Hz, 3H).

^{13}C NMR (126 MHz, $CDCl_3$): δ_C [ppm] = 171.6, 169.7, 142.1, 138.5, 128.4, 128.0, 126.3, 118.2, 61.9, 52.9, 42.0, 41.1, 29.8, 28.7, 27.3, 14.2.

HRMS (ESI^+) calc. for $C_{18}H_{22}NaO_4S$ $[M+Na]^+$: 357.1131, found: 357.1147.

1-Ethyl 2-methyl 1-((2-ethoxy-2-oxoethyl)thio)-2-(1-phenylvinyl)cyclopropane-1,2-dicarboxylate (6b)

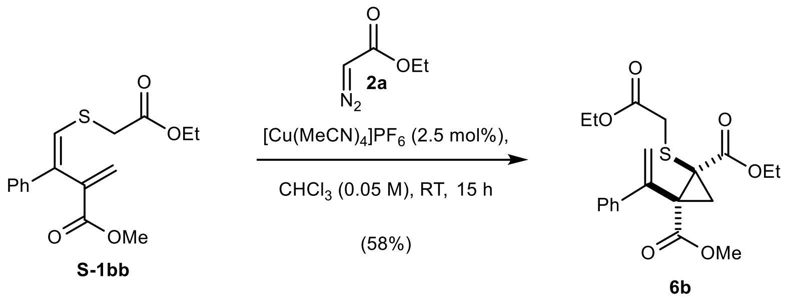

Following GP1, 1,3-diene **S-1bb** (61.3 mg, 0.200 mmol, 1.00 equiv) was converted into VCP **6b**. Purification by flash-column chromatography on silica gel (15% EtOAc in PE + 1% Et_3N) furnished VCP **6b** (45.5 mg, 0.116 mmol, 58%) as a colorless oil.

TLC (10% EtOAc in PE): R_f = 0.18 (UV, CAM).

^{1}H NMR (500 MHz, $CDCl_3$): δ_H [ppm] = 7.64 – 7.59 (m, 2H), 7.37 – 7.32 (m, 2H), 7.31 – 7.27 (m, 1H), 5.91 (s, 1H), 5.40 (s, 1H), 4.32 – 4.18 (m, 2H), 4.11 (q, J = 7.2 Hz, 2H), 3.61 (s, 3H), 3.37 (d, J = 15.3 Hz, 1H), 3.24 (d, J = 15.4 Hz, 1H), 2.45 (d, J = 5.4 Hz, 1H), 1.72 (d, J = 5.5 Hz, 1H), 1.34 (t, J = 7.2 Hz, 3H), 1.22 (t, J = 7.1 Hz, 3H).

^{13}C NMR (126 MHz, $CDCl_3$): δ_C [ppm] = 171.1, 169.5, 169.0, 141.8, 138.6, 128.5, 128.1, 126.4, 118.9, 77.4, 62.1, 61.6, 53.0, 42.9, 40.8, 35.0, 28.5, 14.2.

HRMS (ESI^+) calc. for $C_{20}H_{24}NaO_6S$ $[M+Na]^+$: 415.1186, found: 415.1180.

1-Ethyl 2-methyl 1-(benzylthio)-2-(1-phenylvinyl)cyclopropane-1,2-dicarboxylate (6c)

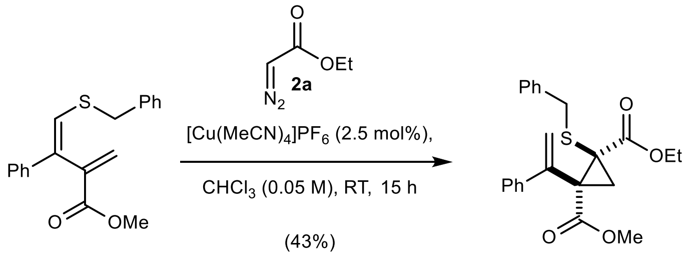

Following GP1, *S*-benzyl-diene **S-1bc** (62.1 mg, 0.200 mmol, 1.00 equiv) was converted into VCP **6c**. Purification by flash-column chromatography on silica gel (20% MTBE in PE + 2% Et_3N) furnished VCP **6c** (33.7 mg, 85.0 µmol, 43%) as a pale-yellow oil.

TLC (20% EtOAc in CyH): R_f = 0.36 (UV, CAM).

^{1}H NMR (500 MHz, $CDCl_3$): δ_H [ppm] = 7.69 – 7.61 (m, 2H), 7.39 – 7.33 (m, 2H), 7.30 – 7.28 (m, 1H), 7.25 – 7.17 (m, 5H), 5.90 (s, 1H), 5.30 (s, 1H), 4.32 – 4.14 (m, 2H), 3.81 (d, *J* = 12.8 Hz, 1H), 3.72 (d, *J* = 12.8 Hz, 1H), 3.59 (s, 3H), 2.36 (d, *J* = 5.4 Hz, 1H), 1.43 (d, *J* = 5.4 Hz, 1H), 1.34 (t, *J* = 7.2 Hz, 3H).

^{13}C NMR (126 MHz, $CDCl_3$): δ_C [ppm] = 171.5, 169.3, 142.0, 138.6, 137.1, 129.3, 128.5, 128.4, 128.0, 127.3, 126.4, 118.4, 77.4, 61.9, 52.9, 41.9, 41.0, 37.5, 28.5, 14.2.

HRMS (ESI^+) calc. for $C_{23}H_{24}NaO_4S$ $[M+Na]^+$: 419.1288, found: 419.1304.

Note: Due to thermal instability of VCP **5a**, concentration under reduced pressure was performed at ambient temperature (bath temperature: 22 °C).

1-Ethyl 2-methyl (phenylthio)-2-(1-phenylvinyl)cyclopropane-1,2-dicarboxylate (6d)

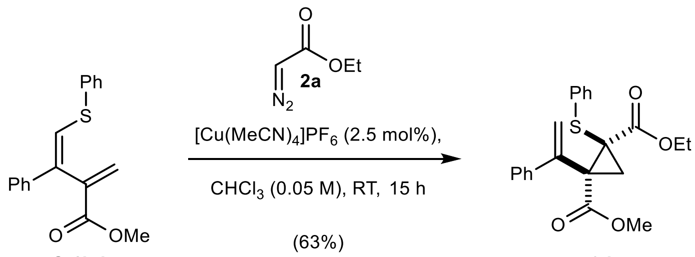

Following GP1, 1,3-diene **S-1bd** (59.3 mg, 0.200 mmol, 1.00 equiv) was converted into VCP **6d**. Purification by flash-column chromatography on silica gel (10%→20% MTBE in PE + 1% Et_3N) furnished VCP **6d** (48.2 mg, 0.126 mmol, 63%) as a pale-yellow solid.

TLC (20% MTBE in PE): R_f = 0.32 (UV, CAM).

^{1}H NMR (500 MHz, $CDCl_3$): δ_H [ppm] = 7.72 – 7.68 (m, 2H), 7.41 – 7.19 (m, 8H), 5.95 (s, 1H), 5.34 (s, 1H), 4.33 – 4.19 (m, 2H), 3.59 (s, 3H), 2.54 (d, *J* = 5.7 Hz, 1H), 1.66 (d, *J* = 5.6 Hz, 1H), 1.30 (t, *J* = 7.1 Hz, 3H).

^{13}C NMR (126 MHz, $CDCl_3$): δ_C [ppm] = 171.0, 169.7, 141.8, 138.5, 134.7, 130.6, 129.1, 128.5, 128.1, 127.2, 126.3, 118.5, 62.1, 52.9, 43.6, 41.9, 28.3, 14.1.

HRMS (ESI^+) calc. for $C_{22}H_{22}NaO_4S$ $[M+Na]^+$: 405.1131, found: 405.1111.

5.2. Limitations

The 1,3-dienes and diazo reagents depicted in Figure S2 did not allow for the successful synthesis and isolation of the corresponding VCPs. Subjecting **S-1p** to the optimized reaction conditions gave a complex mixture, likely due to competing succinic anhydride formation and decomposition, which hindered purification. Almost full conversion was observed for **S-1q,** and a promising signal set (14% NMR yield) appeared in the crude NMR, but we were unable to isolate the product. Aldehyde **S-1ak** also showed productive reactivity giving 34% NMR yield of a VCP intermediate, which, however, rapidly decomposed during attempted purification. This likely reflects the high intrinsic reactivity of the resulting donor–acceptor cyclopropane. Nitrile **S-1al** showed full conversion but no product in the crude ^{1}H NMR, likely due to competing reactivity of the CN-group, aligning with the unsuccessful reaction in MeCN (Table S1, entry 10). Diazo reagents lacking carbonyl groups (**2c**, **2d**, **S-2k**) or α-protons (**2e**) typically pose challenges and often give complex mixtures alongside dimerization products analogous to **S-20** as (*E*/*Z*)-mixtures. However, these limitations can be partially addressed by preinstalling the EWG during the DHT ring-opening step (see Section 7).

A.1,3-Diene Limitations

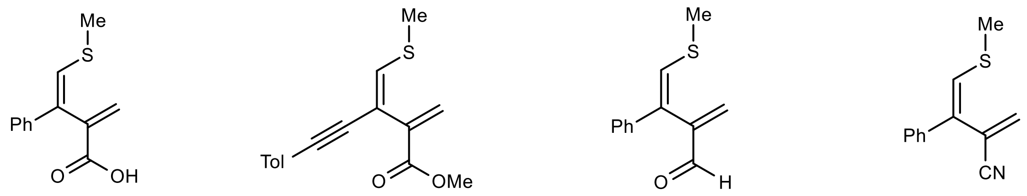

S-1p (complex mixture) | **S-1q** (low product formation) | **S-1ak** (corresponding VCP rapidly decomposes) | **S-1al** (no VCP fromed)

B. Diazo Reagent Limitations

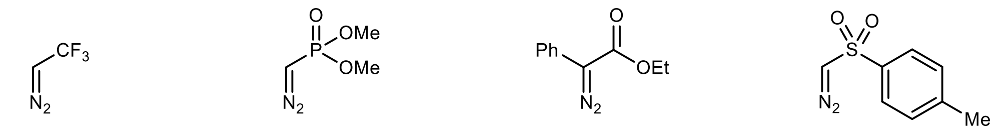

2c (no VCP formed) | **2d** (complex mixture) | **2e** (no VCP formed) | **S-2k** (complex mixture)

Figure S2. Limitations of the developed VCP synthesis.

6. Mechanistic Studies

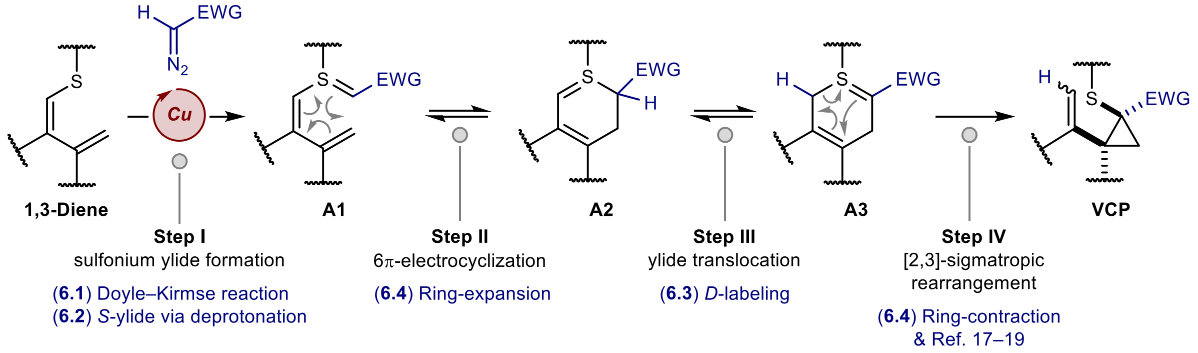

Figure S3. Mechanistic hypothesis and supporting experiments for the elemental steps I–IV.

6.1. Doyle–Kirmse Reaction

Ethyl (Z)-2-((3-(methoxycarbonyl)-2-phenylbuta-1,3-dien-1-yl)thio)pent-4-enoate (7)

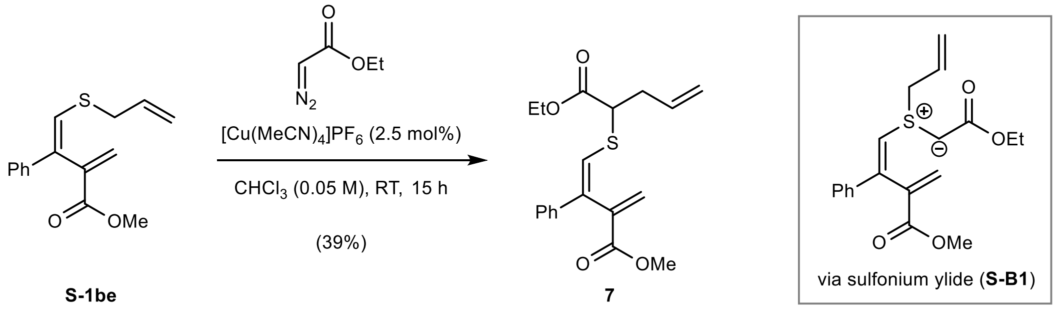

Following GP1, 1,3-diene **S-1be** (52.1 mg, 0.200 mmol, 1.00 equiv) was converted into Doyle–Kirmse product **7**. Purification by flash-column chromatography on silica gel (1%→2% EtOAc in CyH) furnished **7** (26.7 mg, 77.1 µmol, 39%) as a colorless oil.

TLC (33% EtOAc in CyH): $R_f = 0.42$ (UV, CAM).

^{1}H NMR (500 MHz, $CDCl_3$): δ_H [ppm] = 7.34 – 7.24 (m, 5H), 6.83 (s, 1H), 6.66 (d, J = 1.5 Hz, 1H), 5.87 – 5.76 (m, 2H), 5.20 – 5.10 (m, 2H), 4.30 – 4.19 (m, 2H), 3.68 (s, 3H), 3.55 (dd, J = 8.7, 2.1 Hz, 1H), 2.80 – 2.68 (m, 1H), 2.59 – 2.49 (m, 1H), 1.30 (t, J = 7.1 Hz, 3H).

^{13}C NMR (126 MHz, $CDCl_3$): δ_C [ppm] = 171.3, 166.4, 139.4, 138.3, 136.8, 133.7, 131.4, 131.4, 128.6, 127.5, 125.8, 123.6, 118.4, 61.6, 52.3, 47.9, 35.8, 14.3.

HRMS (ESI$^+$) calc. for $C_{19}H_{22}NaO_4S$ $[M+Na]^+$: 369.1131, found: 369.1158.

Conclusion: Under the optimized reaction conditions, no VCP but exclusive formation of the Doyle–Kirmse product **7** was observed. Since the product presumably arises from a [2,3]-sigmatropic rearrangement of sulfonium ylide (**S-B1**),[14,15] this experiment supports the hypothesized carbene transfer reaction catalyzed by Cu (Figure S3, 1,3-diene→**A1**).

6.2. Base-promoted Cyclopropanation from Sulfonium Salt

(*Z*)-(2-Ethoxy-2-oxoethyl)(3-(methoxycarbonyl)-2-phenylbuta-1,3-dien-1-yl)(methyl)sulfonium triflate salt (8)

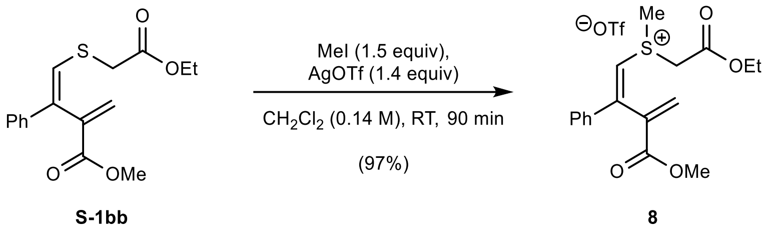

To a solution of 1,3-diene **S-1bb** (21.5 mg, 70.1 µmol, 1.00 equiv) in CH_2Cl_2 (0.50 mL, 0.14 M), AgOTf (25.0 mg, 97.3 µmol, 1.39 equiv) and MeI (6.5 µL, 0.11 mmol, 1.7 equiv) were added at RT. The formation of a yellow precipitate was immediately observed. After 90 minutes, the reaction mixture was filtered over a plug of Celite® and the filtrate was concentrated under reduced pressure to furnish crude triflate salt **8** (32.1 mg, 68.2 µmol, 97%). Recrystallization from MTBE gave crystals suitable for X-ray analysis (CCDC-2496551).

TLC (10% MeOH in CH_2Cl_2): R_f = 0.70 (UV, CAM).

^{1}H NMR (500 MHz, $CDCl_3$): δ_H [ppm] = 7.56 – 7.51 (m, 2H), 7.47 – 7.45 (m, 1H), 7.44 – 7.40 (m, 2H), 7.23 (s, 1H), 6.86 (s, 1H), 6.08 (s, 1H), 4.90 (d, *J* = 16.7 Hz, 1H), 4.70 (d, *J* = 16.8 Hz, 1H), 4.27 (q, *J* = 7.1 Hz, 2H), 3.84 (s, 3H), 3.27 (s, 3H), 1.30 (t, *J* = 7.2 Hz, 3H).

^{13}C NMR (126 MHz, $CDCl_3$): δ_C [ppm] = 165.4, 164.4, 157.7, 136.1, 135.5, 134.5, 132.0, 129.4, 128.3, 111.1, 64.0, 53.5, 46.6, 26.8, 14.0.

^{19}F NMR (376 MHz, $CDCl_3$): δ_F [ppm] = −79.39.

HRMS (ESI^+) calc. for $C_{17}H_{21}O_4S$ $[M{-}OTf]^+$: 321.1155, found: 321.1166.

HRMS (ESI^-) calc. for CF_3O_3S $[OTf]^-$: 148.9526, found: 148.9532.

Methyl 2-benzoyl-2-(methylthio)-1-(1-phenylvinyl)cyclopropane-1-carboxylate (3a)

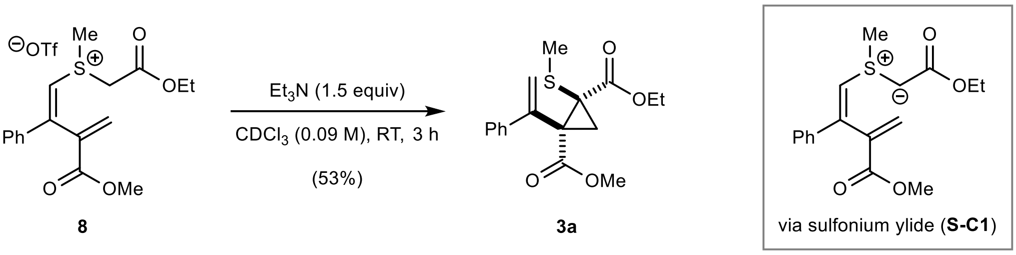

To a solution of sulfonium salt **8** (6.4 mg, 14 µmol, 1.0 equiv) in $CDCl_3$ (0.10 mL, 0.14 M), Et_3N (3.0 µL, 20 µmol, 1.5 equiv) in $CDCl_3$ (50 µL) was added at RT. After three hours, CH_2Br_2 (2.4 µL, 34 µmol, 2.5 equiv) was added as internal standard to determine the yield of VCP **3a** (53%) through ^{1}H NMR

spectroscopic analysis. The data obtained were in full agreement with those reported in the cyclopropanation of 1,3-diene **1a** with diazo reagent **2a**.

Conclusion: Alternative generation of the intermediary sulfonium ylide **S-A1** through deprotonation of sulfonium salt **7** led to efficient formation of VCP **3a**. This observation supports the hypothesis that *S*-ylide **S-A1** functions as a reactive intermediate initially formed via Cu-catalyzed carbene transfer reaction (Figure S3, 1,3-diene→**A1**).

6.3. Isotope Labeling Studies

Ethyl 2-diazoacetate-*d* (*D*-2a)

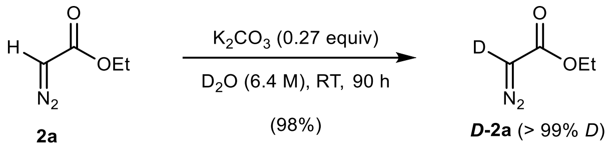

Ethyl 2-diazoacetate-*d* (***D*-2a**) was prepared according to a literature-known procedure.[16] To a solution of ethyl diazoacetate (**2a**, 0.50 mL, 87% in CH_2Cl_2, 4.8 mmol, 1.0 equiv) in D_2O (0.75 mL, 6.4 M), K_2CO_3 (175 mg, 1.26 mmol, 0.270 equiv) was added in one portion at RT. After 72 hours, 95% *D*-incorporation was identified through ^{1}H-NMR spectroscopy, the solvent was removed under reduced pressure followed by the addition of D_2O (0.75 mL, 6.4 M) and K_2CO_3 (175 mg, 1.26 mmol, 26.6 mol%). After 18 hours, decantation of the product and filtration over Na_2SO_4 furnished ***D*-2a** as an orange oil (532 mg, 4.66 mmol, 98% yield, > 99% D), which was used in the next step without further purification.

TLC (10% EtOAc in PE): R_f = 0.50 (UV, CAM).

1-Ethyl 2-methyl 1-(methylthio)-2-(1-phenylvinyl-2-*d*)cyclopropane-1,2-dicarboxylate (*D*-3a)

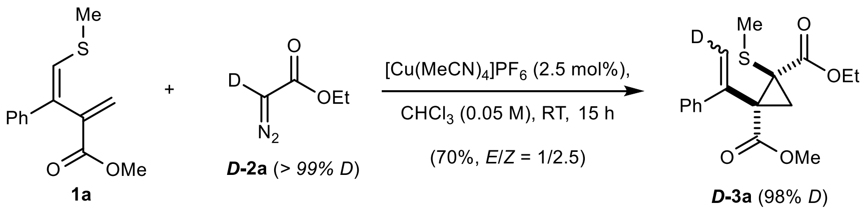

Following GP1, deuterated VCP ***D*-3a** was synthesized employing 1,3-diene **1a** (23.4 mg, 0.100 mmol, 1.00 equiv) and ethyl 2-diazoacetate-*d* (***D*-2a**, 36 µL, 0.30 mmol, 3.0 equiv, > 99% *D*). After removal of $CHCl_3$ under reduced pressure, CH_2Br_2 (3.5 µL, 50 µmol, 0.50 equiv) was added as internal standard to determine the yield of VCP ***D*-3a** (70%, *E*/*Z* = 1/2.5, 98% *D*) through ^{1}H NMR spectroscopic analysis (see Figure S4).

HRMS (ESI$^+$) calc. for $C_{17}H_{19}DNaO_4S$ $[M+Na]^+$: 344.1037, found: 344.1030.

Note: The reaction and NMR analysis were performed using dry and acid-free $CHCl_3$ and $CDCl_3$ (stored over 4 Å molecular sieves and K_2CO_3) to maintain high isotopic purity in ***D*-2a**.

Conclusion: Highly efficient *D*-incorporation into VCP **3a** underpins the hypothesized mechanistic scenario of ylide translocation through a protonation/deprotonation process (Step III, **A2→A3**).

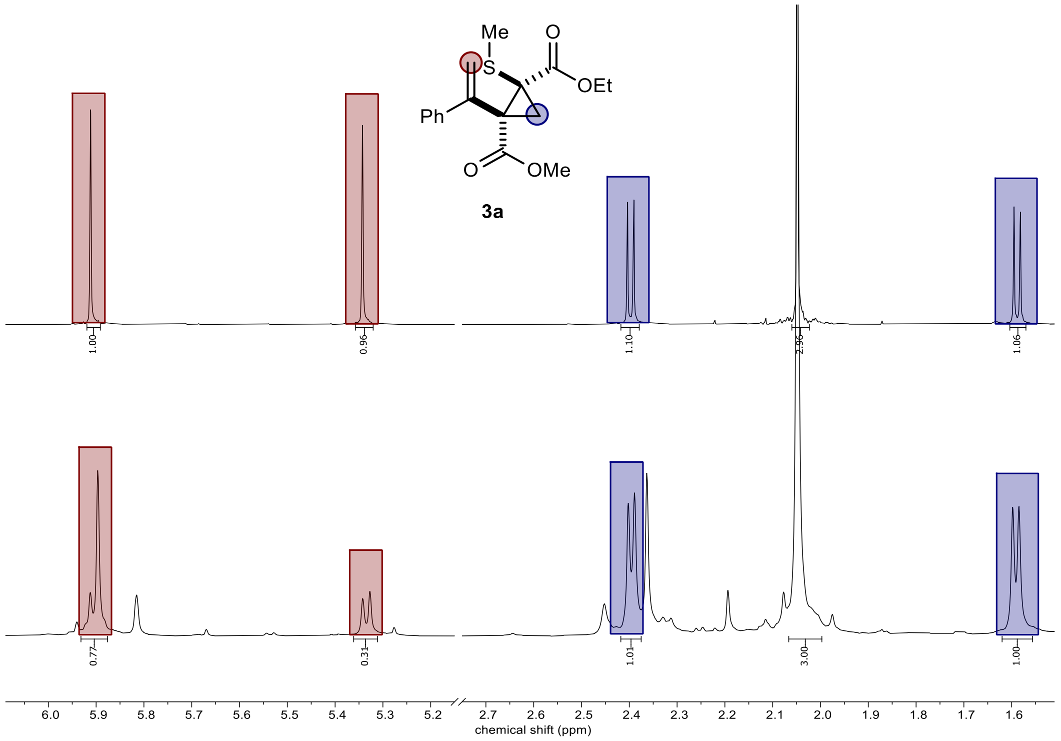

Figure S4. ^{1}H NMR spectra of isolated VCP **3a** (top) and deuterated VCP ***D*-3a** (bottom).

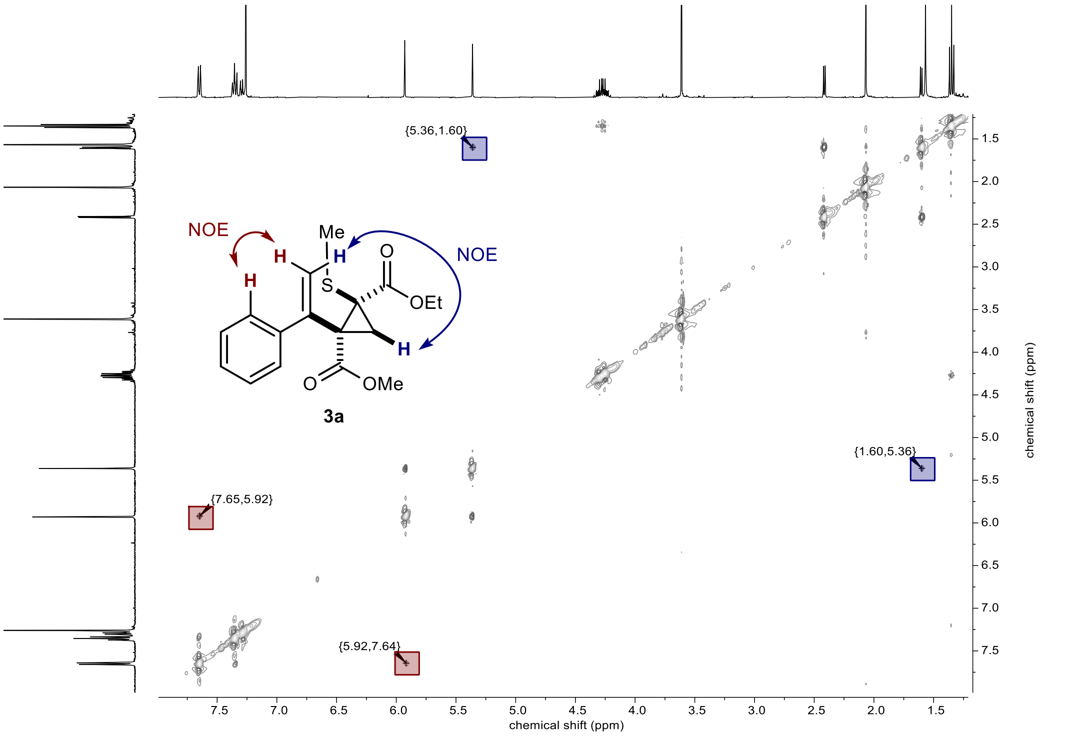

Figure S5. 2D (^{1}H–^{1}H) NOESY NMR spectrum of VCP **3a**.

6.4. Synthesis and Ring-Contraction of Thiopyrane Species

Methyl 2-benzoyl-5-phenyl-3,4-dihydro-2*H*-thiopyran-4-carboxylate (10c)

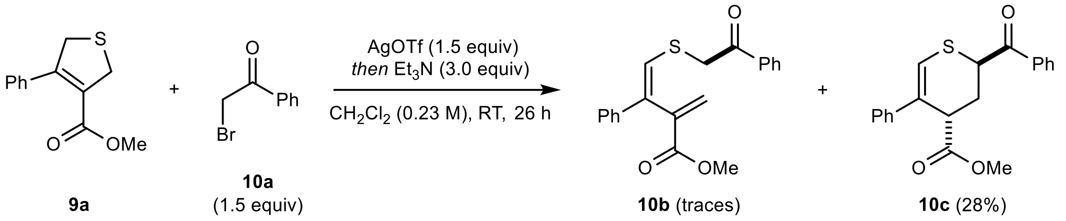

To a solution of DHT **9a** (100 mg, 0.454 mmol, 1.00 equiv) in CH_2Cl_2 (2 mL, 0.23 M), ω-bromo acetophenone **10a** (136 mg, 0.681mmol, 1.50 equiv) and AgOTf (175 mg, 0.681 mmol, 1.50 equiv) were sequentially added at RT. After 27 hours, Et_3N (0.19 mL, 1.4 mmol, 3.0 equiv) was added and after one additional hour, the solvent was removed under reduced pressure. Purification by flash-column chromatography (1% → 7.5% MTBE in PE) furnished 3,4-dihydro-2*H*-thiopyrane **10c** (42.0 mg, 0.125 mmol, 28%) as a yellow crystalline solid. Recrystallization from PE and MeOH gave crystals suitable for X-ray analysis (CCDC-2496552).

TLC (10% MTBE in PE): R_f = 0.38 (UV, $KMnO_4$, CAM).

^{1}H NMR (500 MHz, $CDCl_3$): δ_H [ppm] = 8.09 – 8.05 (m, 2H), 7.65 – 7.59 (m, 1H), 7.54 – 7.48 (m, 2H), 7.35 – 7.31 (m, 4H), 7.28 – 7.23 (m, 1H), 6.56 (s, 1H), 5.11 (dd, *J* = 11.3, 3.0 Hz, 1H), 4.07 – 4.03 (m, 1H), 3.65 (s, 3H), 2.78 (dt, *J* = 14.2, 3.1 Hz, 1H), 2.39 (ddd, *J* = 14.2, 11.2, 4.9 Hz, 1H).

^{13}C NMR (126 MHz, $CDCl_3$): δ_C [ppm] = 196.1, 173.8, 140.8, 135.1, 134.0, 129.1, 129.0, 129.0, 128.7, 127.3, 125.6, 120.0, 52.5, 41.6, 41.1, 28.3.

HRMS (ESI^+) calc. for $C_{20}H_{18}NaO_3S$ $[M+Na]^+$: 361.0869; found: 361.0882.

Conclusion: The application of α-bromo acetophenone (**10a**) as electrophile only led to traces of the 1,3-diene **10b** but ring-expansion to six-membered *S*-heterocycle **10c**. Likely, this intermediate arises from an intramolecular Michael-addition of initially formed **10b** supporting the hypothesized C−C bond formation through the envisioned 6π-electrocyclization (Step II, **A1→A2**).

Methyl 2-benzoyl-2-(methylthio)-1-(1-phenylvinyl)cyclopropane-1-carboxylate (5d)

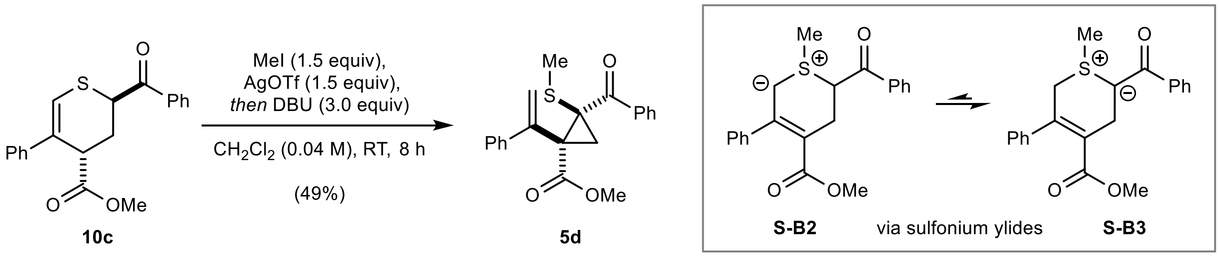

To a solution of DHTP **10c** (10.0 mg, 29.5 µmol, 1.00 equiv) in CH_2Cl_2 (0.75 mL, 0.040 M), MeI (2.8µL, 45 µmol, 1.5 equiv) and AgOTf (11.6 mg, 45 µmol, 1.52 equiv) were added at RT. After four hours, DBU (13.5 µL, 89.9 µmol, 3.04 equiv) was added. After additional four hours, the reaction mixture was filtered, and the filtrate was concentrated under reduced pressure at RT. Upon concentration, CH_2Br_2 (1.05 µL, 14.8 µmol, 0.500 equiv) was added as internal standard to determine the yield of VCP **5d** (49%) through ^{1}H NMR spectroscopic analysis. The analytical data obtained were in full agreement with those reported in the cyclopropanation of 1,3-diene **1a** with diazo reagent **S-2j**.

Conclusion: Application of DHTP **10c** to the one-pot procedure (*S*-methylation and base-promoted cyclopropane formation) led to VCP **5d** in moderate yield. From a mechanistic perspective, initial formation of sulfonium salt **S-B2** is envisioned to be followed by its equilibration to **S-B3**. Subsequent [2,3]-sigmatropic rearrangement is expected to yield VCP **5d** in accordance with the mechanistic hypothesis (Step IV, **A3**→VCP).[17–19]

6.5. Control Experiment: Non-Directed Cyclopropanation

Methyl 4-phenyl-2,5-dihydrothiophene-3-carboxylate 1,1-dioxide (S-15a)

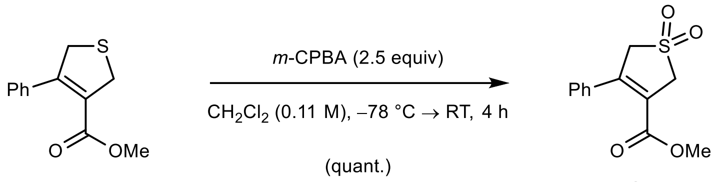

To a solution of DHT **9a** (525 mg, 2.38 mmol, 1 equiv) in CH_2Cl_2 (10 mL, 0.24 M), a solution of *meta*–chloroperoxybenzoic acid (*m*–CPBA, 70 %, 1.35 g, 5.88 mmol, 2.47 equiv) in CH_2Cl_2 (12 mL, 0.49 mmol) was added dropwise at −78 °C. Upon addition, the reaction mixture was allowed to warm to RT. After four hours, saturated aqueous $Na_2S_2O_3$ solution (50 mL) and $NaHCO_3$ solution (50 mL) were added, the layers were separated, and the aqueous layer was extracted with EtOAc (3 × 100 mL). The combined organic layers were washed with $NaHCO_3$ solution (3 × 100 mL), dried over Na_2SO_4, the dried solution was filtered, and the filtrate was concentrated under reduced pressure to furnish the title compound **S-15a** (601 mg, 2.38 mmol, 100%) as a pale-yellow solid, which could be used in the next step without further purification.

TLC (1% MeOH in CH_2Cl_2): $R_f = 0.41$ (UV, KMnO4).

^{1}H NMR (500 MHz, $CDCl_3$): δ_H [ppm] = 7.42 – 7.36 (m, 3H), 7.27 – 7.23 (m, 2H), 4.27 (t, J = 1.5 Hz, 2H), 4.23 (t, J = 1.5 Hz, 2H), 3.64 (s, 3H).

^{13}C NMR (126 MHz, $CDCl_3$): δ_C [ppm] = 163.1, 145.8, 134.1, 129.7, 128.6, 127.5, 122.7, 62.2, 58.1, 52.3.

HRMS (ESI$^+$) calc. for $C_{12}H_{12}NaO_4S$ $[M+Na]^+$: 275.0349; found: 275.0357.

Methyl 2-methylene-3-phenylbut-3-enoate (S-15b)

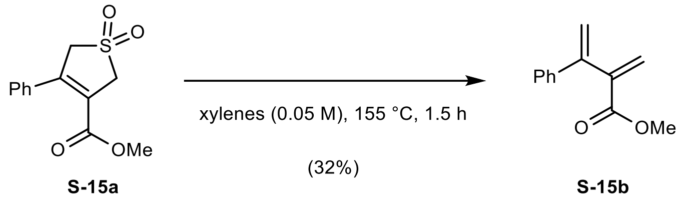

Sulfolene **S-15a** (103 mg, 0.408 mmol, 1.00 equiv) was added to a 50 mL flask equipped with a reflux condensor and dissolved in xylenes (8.0 mL, 0.05 M). The mixture was heated to 155 °C. After 90 minutes, the reaction mixture was allowed to cool to RT and slightly concentrated using the high vacuum. Purification by flash-column chromatography (0% → 2% MTBE in PE) furnished diene **S-15b** (24.8 mg, 0.132 mmol, 32%) as a colorless liquid.

TLC (5% MTBE in PE): R_f = 0.41 (UV, $KMnO_4$).

^{1}H NMR (500 MHz, $CDCl_3$): δ_H [ppm] = 7.37 – 7.27 (m, 5H), 6.34 (d, J = 1.7 Hz, 1H), 5.82 (d, J = 1.7 Hz, 1H), 5.53 (d, J = 1.4 Hz, 1H), 5.40 (d, J = 1.4 Hz, 1H), 3.66 (s, 3H).

^{13}C NMR (126 MHz, $CDCl_3$): δ_C [ppm] = 167.2, 146.1, 142.0, 139.7, 128.5, 128.1, 127.9, 126.7, 116.5, 52.2.

HRMS (ESI$^+$) calc. for $C_{12}H_{12}NaO_2$ $[M+Na]^+$: 211.0730; found: 211.0738.

Attempted Cyclopropanation of S-15b Lacking the Sulfur-Directing Group

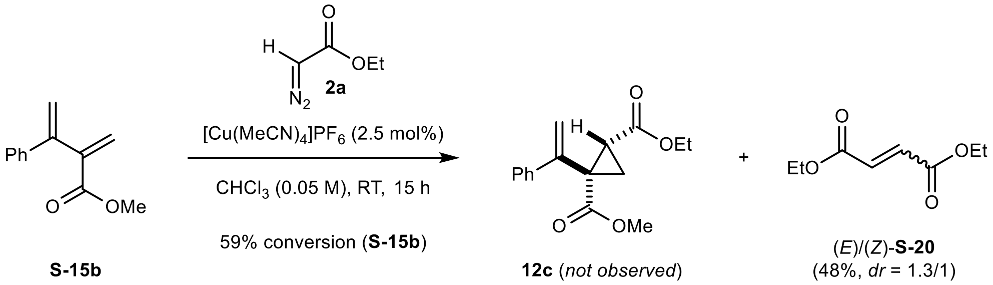

Following GP1, diene **S-15b** (24.5 mg, 0.130 mmol, 1.00 equiv) was subjected to cyclopropanation conditions using ethyl 2-diazoacetate (**2a**, 47 µL, 0.39 mmol, 3.0 equiv). After filtration over deactivated silica and removal of $CHCl_3$ under reduced pressure, CH_2Br_2 (9.0 µL, 0.13 mmol, 1.0 equiv) was added as internal standard. ^{1}H NMR spectroscopic analysis revealed remaining **S-15b** (41%) and **S-20** (48%, E/Z = 1.3/1) as the only detectable species.

Conclusion: No cyclopropanation products such as **12c** were formed under the optimized reaction conditions. Instead, the reaction predominantly furnished the dimerization products (E)- and (Z)-**S-20**. This outcome clearly underscores the advantage of our sulfur-directed approach for the stereoselective construction of VCPs.

7. Alternative Vinyl Cyclopropane Formation

Methyl 2-(dimethoxyphosphoryl)-2-(methylthio)-1-(1-phenylvinyl)cyclopropane-1-carboxylate (11a)

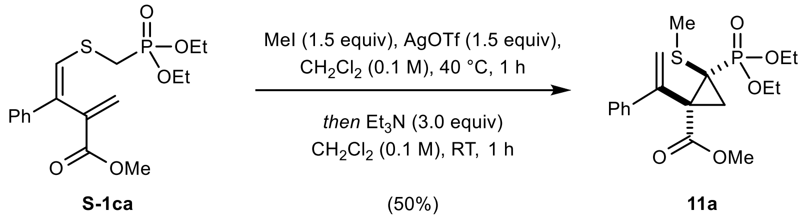

To a solution of 1,3-diene **S-1ca** (62.0 mg, 0.167 mmol, 1.00 equiv) in CH_2Cl_2 (1.7 mL, 0.098 M) were added MeI (16 µL, 0.26 mmol, 1.5 equiv) and AgOTf (66 mg, 0.252 mmol, 1.50 equiv) at RT. Upon addition, the reaction mixture was heated to 40 °C. After one hour, the reaction mixture was allowed to cool to RT and Et_3N (70 µL, 0.50 mmol, 3.0 equiv) was added dropwise. After one hour, the reaction mixture was filtered over a plug of Celite®, and the filtrate was concentrated under reduced pressure. Purification by flash-column chromatography on silica gel (0.5%→1% *i*-PrOH in PE + 1% Et_3N) furnished VCP **11a** (32.0 mg, 83.2 µmol, 50%) as a colorless, crystalline solid.

TLC (5% *i*-PrOH in PE): R_f = 0.24 (UV, $KMnO_4$, CAM).

^{1}H NMR (500 MHz, $CDCl_3$) δ_H [ppm] = 7.65 – 7.59 (m, 2H), 7.36 – 7.25 (m, 3H), 5.81 (s, 1H), 5.63 (s, 1H), 4.30 – 4.15 (m, 4H), 3.70 (s, 3H), 2.33 (dd, *J* = 15.7, *J* = 5.6 Hz, 1H), 2.14 (s, 3H), 1.43 – 1.33 (m, 7H).

^{13}C NMR (126 MHz, $CDCl_3$): δ_C [ppm] = 169.7 (d, *J* = 5.7 Hz), 143.0, 139.1, 128.2, 127.9, 127.4, 120.6, 63.5 (d, *J* = 6.7 Hz), 63.1 (d, *J* = 6.6 Hz), 52.9, 44.77, 31.1 (d, *J* = 196.8 Hz), 24.65, 16.65 (d, *J* = 6.8 Hz), 16.60 (d, *J* = 7.0 Hz), 16.34.

^{31}P{^{1}H,^{13}C} NMR (162 MHz, $CDCl_3$): δ_P [ppm] = 22.46.

HRMS (ESI$^+$) calc. for $C_{18}H_{25}NaO_5PS$ $[M+Na]^+$: 407.1053, found: 407.1058.

Methyl 2-(methylthio)-1-(1-phenylvinyl)-2-(trifluoromethyl)cyclopropane-1-carboxylate (11b)

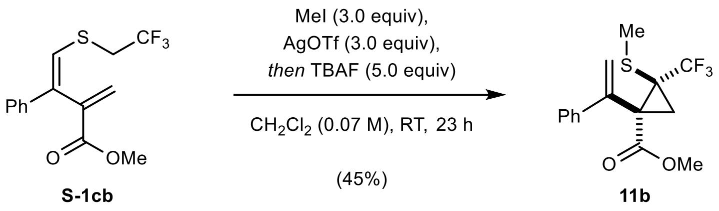

To a solution of 1,3-diene **S-1cb** (45.3 mg, 0.150 mmol, 1.00 equiv) in CH_2Cl_2 (2.25 mL, 0.0700 M), MeI (28 µL, 0.45 mmol, 3.0 equiv) and AgOTf (118 mg, 0.450 mmol, 3.00 equiv) were added at RT. Upon addition, the reaction mixture was heated to 40 °C. After 15 hours, the reaction mixture was cooled to RT and TBAF (0.75 mL, 0.75 mmol, 5.00 equiv, 1.0 M in hexanes) was added dropwise. After eight hours, the reaction mixture was filtered over a plug of Celite®, and the filtrate was concentrated under

reduced pressure. Purification by flash-column chromatography on silica gel (5% MTBE in PE + 1% Et_3N) furnished VCP **11b** (21.2 mg, 66.9 µmol, 45%) as a yellow oil.

TLC (5% MTBE in PE): $R_f = 0.41$ (UV, CAM, $KMnO_4$).

^{1}H NMR (500 MHz, $CDCl_3$): δ_H [ppm] = 7.60 – 7.54 (m, 2H), 7.40 – 7.32 (m, 2H), 7.32 – 7.28 (m, 1H), 5.82 (s, 1H), 5.65 (s, 1H), 3.72 (s, 3H), 2.25 (d, J = 6.3 Hz, 1H), 2.12 (d, J = 1.0 Hz, 3H), 1.40 – 1.34 (m, 1H).

^{13}C NMR (126 MHz, $CDCl_3$): δ_C [ppm] = 169.0, 142.3, 138.7, 128.4, 128.1, 127.1, 126.1 (q, J = 275.7 Hz), 120.9, 53.3, 43.7, 38.5 (q, J = 34.9 Hz), 23.3, 16.1.

^{19}F NMR (376 MHz, $CDCl_3$): δ_F [ppm] = –66.15.

HRMS (ESI^+) calc. for $C_{15}H_{15}F_3NaO_2S$ $[M+Na]^+$: 339.0637, found: 339.0648.

Methyl 2-cyano-2-(methylthio)-1-(1-phenylvinyl)cyclopropane-1-carboxylate (11c)

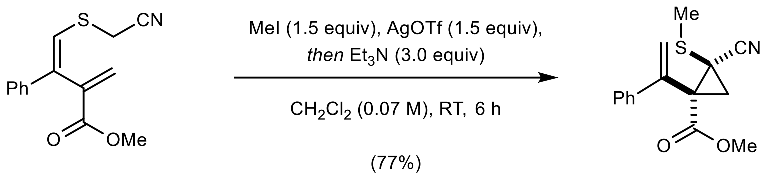

To a solution of 1,3-diene **S-1cc** (45.0 mg, 0.174 mmol, 1.00 equiv) in CH_2Cl_2 (2.5 mL, 0.070 M), MeI (17 µL, 0.26 mmol, 1.5 equiv) and AgOTf (66.8 mg, 0.260 mmol, 1.50 equiv) were added at RT. After six hours, Et_3N (72 µL, 0.52 mmol, 3.0 equiv) was added dropwise. After 15 minutes, the reaction mixture was filtered over a plug of Celite®, and the filtrate was concentrated under reduced pressure. Purification by flash-column chromatography on silica gel (20% MTBE in PE + 1% Et_3N) furnished VCP **11c** (36.9 mg, 0.135 mmol, 77%) as a yellow oil.

TLC (15% EtOAc in PE): $R_f = 0.28$ (UV, CAM).

^{1}H NMR (500 MHz, $CDCl_3$): δ_H [ppm] = 7.43 – 7.40 (m, 2H), 7.38 – 7.34 (m, 2H), 7.33 – 7.29 (m, 1H), 5.94 (s, 1H), 5.42 (s, 1H), 3.79 (s, 3H), 2.57 (d, J = 5.0 Hz, 1H), 2.15 (s, 3H), 1.62 (d, J = 5.5 Hz, 1H).

^{13}C NMR (126 MHz, $CDCl_3$): δ_C [ppm] = 169.5, 140.8, 138.3, 128.6, 128.4, 126.3, 120.4, 117.7, 53.6, 43.5, 30.3, 26.9, 15.8.

HRMS (ESI^+) calc. for $C_{15}H_{15}NNaO_2S$ $[M+Na]^+$: 296.0716, found: 296.0727.

8. Postmodifications

1-Ethyl 2-methyl 1-(methylsulfinyl)-2-(1-phenylvinyl)cyclopropane-1,2-dicarboxylate (12a & 12b)

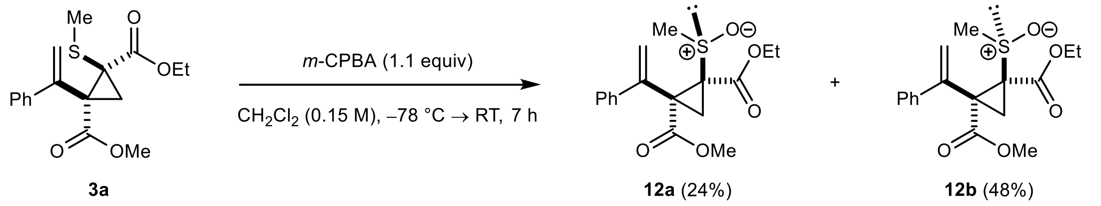

To a solution of VCP **3a** (48.5 mg, 0.150 mmol, 1.00 equiv) in CH_2Cl_2 (0.5 mL, 0.3 M), a solution of *meta*–chloroperoxybenzoic acid (*m*–CPBA, 70%, 39.0 mg, 0.158 mmol, 1.05 equiv) in CH_2Cl_2 (0.5 mL, 0.3 M) was added dropwise at –78 °C. After three hours, the reaction was allowed to slowly warm to RT. After four hours, saturated aqueous $NaHCO_3$ solution (2.0 mL) was added, the layers were separated, and the aqueous layer was extracted with MTBE (2 × 2.0 mL). The combined organic layers were dried over Na_2SO_4. The dried solution was filtered, and the filtrate was concentrated under reduced pressure. Purification by flash-column chromatography on silica gel (40% EtOAc in PE + 1% Et_3N) furnished sulfoxides **12a** (12.3 mg, 36.6 µmol, 24%) and **12b** (24.1 mg, 71.6 µmol, 48%) as colorless solids. Recrystallization of **12a** from MTBE and CH_2Cl_2 gave crystals suitable for X-ray analysis (CCDC-2496553).

Data for minor diastereomer (**12a**)

TLC (40% EtOAc in PE): R_f = 0.28 (UV).

^{1}H NMR (500 MHz, $CDCl_3$): δ_H [ppm] = 7.56 – 7.52 (m, 2H), 7.36 – 7.31 (m, 2H), 7.30 – 7.26 (m, 1H), 5.88 (s, 1H), 5.52 (s, 1H), 4.37 – 4.23 (m, 2H), 3.68 (s, 3H), 2.47 (s, 3H), 2.32 (d, *J* = 6.0 Hz, 1H), 2.14 (d, *J* = 6.0 Hz, 1H), 1.36 (t, *J* = 7.1 Hz, 3H).

^{13}C NMR (126 MHz, $CDCl_3$): δ_C = 170.6, 166.0, 139.9, 138.7, 128.5, 128.2, 126.6, 120.0, 62.6, 55.1, 53.3, 40.7, 37.9, 18.5, 14.2.

HRMS (ESI^+) calc. for $C_{17}H_{20}NaO_5S$ $[M+Na]^+$: 359.0924, found: 359.0928.

Data for major diastereomer (**12b**)

TLC (40% EtOAc in PE): R_f = 0.13 (UV, CAM).

^{1}H NMR (500 MHz, $CDCl_3$): δ_H [ppm] = 7.56 – 7.52 (m, 2H), 7.37 – 7.32 (m, 2H), 7.31 – 7.27 (m, 1H), 6.04 (s, 1H), 5.66 (s, 1H), 4.44 – 4.30 (m, 2H), 3.67 (s, 3H), 2.70 (s, 3H), 2.29 (d, *J* = 6.1 Hz, 1H), 1.37 (t, *J* = 7.2 Hz, 3H), 1.24 (d, *J* = 6.2 Hz, 1H).

^{13}C NMR (126 MHz, $CDCl_3$): δ_C [ppm] = 170.7, 164.2, 139.4, 138.6, 128.5, 128.2, 126.6, 121.3, 62.6, 55.2, 53.3, 42.0, 37.1, 20.8, 14.4.

HRMS (ESI^+) calc. for $C_{17}H_{20}NaO_5S$ $[M+Na]^+$: 359.0924, found: 359.0930.

2-Ethyl 1-methyl 1-(1-phenylvinyl)cyclopropane-1,2-dicarboxylate (12c)

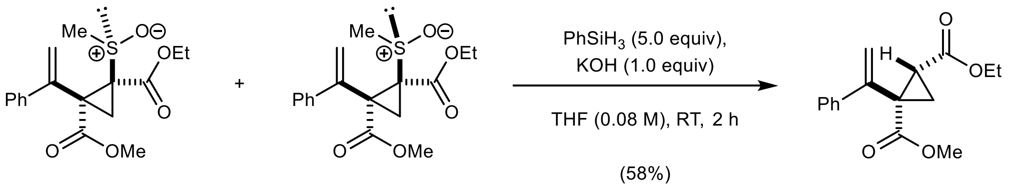

To a solution of sulfoxides **12a** and **12b** (*dr* = 1/1.5, 56.9 mg, 169 µmol, 1.00 equiv) in THF (2.0 mL, 0.08 M) sitting in a water bath, $PhSiH_3$ (0.10 mL, 0.85 mmol, 5.0 equiv) and KOH (9.5 mg, 0.17 mmol, 1.0 equiv) were added at RT. After two hours, saturated aqueous NH_4Cl solution (2 mL), water (2 mL) and CH_2Cl_2 (3 mL) were added, the layers were separated, and the aqueous layer was extracted with CH_2Cl_2 (2 × 3 mL). The combined organic layers were dried over Na_2SO_4, the dried solution was filtered, and the filtrate was concentrated under reduced pressure. Purification by flash-column chromatography on silica gel (20% MTBE in PE + 1% Et_3N) furnished the title compound **12c** (27.0 mg, 98.4 µmol, 58%) as a pale-yellow oil.

TLC (20% MTBE in PE): R_f = 0.44 (UV, CAM, $KMnO_4$).

^{1}H NMR (500 MHz, $CDCl_3$): δ_H [ppm] = 7.64 – 7.58 (m, 2H), 7.38 – 7.32 (m, 2H), 7.31 – 7.28 (m, 1H), 5.71 (s, 1H), 5.43 (s, 1H), 4.20 (qq, *J* = 11.0, 7.2 Hz, 2H), 3.60 (s, 3H), 2.26 (dd, *J* = 8.8, 6.7 Hz, 1H), 2.14 (dd, *J* = 6.7, 4.8 Hz, 1H), 1.41 (dd, *J* = 8.8, 4.8 Hz, 1H), 1.30 (t, *J* = 7.1 Hz, 4H).

^{13}C NMR (126 MHz, $CDCl_3$): δ_C [ppm] = 170.6, 170.1, 145.1, 138.4, 128.5, 128.2, 126.5, 116.8, 61.3, 52.7, 37.6, 28.9, 19.7, 14.3.

HRMS (ESI^+) calc. for $C_{16}H_{18}NaO_4$ $[M+Na]^+$: 297.1097, found: 297.1099.

1-Ethyl 2-methyl 1-(methylsulfonyl)-2-(1-phenylvinyl)cyclopropane-1,2-dicarboxylate (12d)

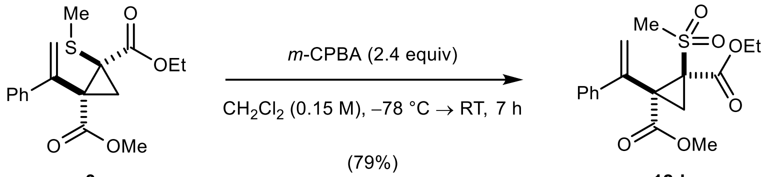

To a solution of VCP **3a** (48.5 mg, 0.150 mmol, 1.00 equiv) in CH_2Cl_2 (0.5 mL, 0.3 M), a solution of *meta*–chloroperoxybenzoic acid (*m*–CPBA, 70%, 88.0 mg, 0.357 mmol, 2.38 equiv) in CH_2Cl_2 (0.5 mL, 0.6 M) was added dropwise at –78 °C. After three hours, the reaction was allowed to slowly warm to RT. After four hours, saturated aqueous $NaHCO_3$ solution (2.0 mL) was added, the layers were separated, and the aqueous layer was extracted with MTBE (2 × 2.0 mL). The combined organic layers were dried over Na_2SO_4. The dried solution was filtered, and the filtrate was concentrated under reduced pressure. Purification by flash-column chromatography on silica gel (40% EtOAc in PE + 1% Et_3N) furnished sulfone **12d** (43.3 mg, 0.123 mmol, 79%) as a very pale-yellow oil.

TLC (40% EtOAc in PE): R_f = 0.64 (UV, CAM).

^{1}H NMR (500 MHz, $CDCl_3$): δ_H [ppm] = 7.54 – 7.49 (m, 2H), 7.38 – 7.33 (m, 2H), 7.32 – 7.27 (m, 1H), 5.87 (s, 1H), 5.62 (s, 1H), 4.39 – 4.27 (m, 2H), 3.70 (s, 3H), 2.81 (s, 3H), 2.41 (d, J = 6.1 Hz, 1H), 2.32 (d, J = 6.1 Hz, 1H), 1.36 (t, J = 7.1 Hz, 3H).

^{13}C NMR (126 MHz, $CDCl_3$): δ_C [ppm] = 169.7, 165.0, 139.4, 139.1, 128.5, 128.3, 126.9, 120.9, 63.1, 55.8, 53.6, 43.4, 41.1, 21.3, 13.9.

HRMS (ESI$^+$) calc. for $C_{17}H_{20}NaO_6S$ [M+Na]$^+$: 375.0873, found: 375.0877.

1-Ethyl 2-methyl 1-(*S*-methyl-*N*-tosylsulfinimidoyl)-2-(1-phenylvinyl)cyclopropane-1,2-dicarboxylate (12e)

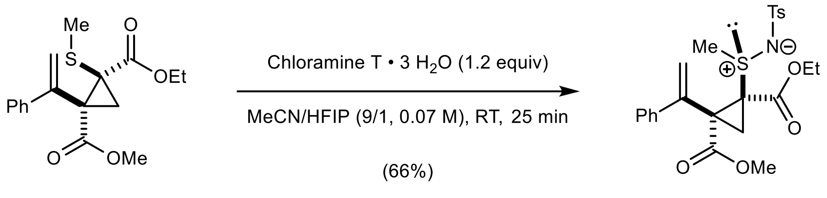

To a solution of VCP **3a** (40.0 mg, 0.125 mmol, 1.00 equiv) in MeCN/HFIP (2.0 mL, 9/1, 0.070 M), chloramine-T trihydrate (42.2 mg, 0.150 mmol, 1.20 equiv) was added in one portion at RT. After 25 minutes, saturated aqueous $Na_2S_2O_3$ solution (2.0 mL) was added, the layers were separated, and the aqueous layer was extracted with MTBE (2 × 2.0 mL). The combined organic layers were dried over Na_2SO_4. The dried solution was filtered, and the filtrate was concentrated under reduced pressure. Purification by flash-column chromatography on silica gel (10%→15% MTBE in CH_2Cl_2) furnished sulfilimine **12e** (40.3 mg, 82.3 µmol, 66%) as a pale-yellow oil. Recrystallization from *n*-pentane and CH_2Cl_2 gave crystals suitable for X-ray analysis (CCDC-2496554).

TLC (10% MTBE in CH_2Cl_2): $R_f = 0.41$ (UV, CAM, $KMnO_4$).

^{1}H NMR (500 MHz, $CDCl_3$): δ_H [ppm] = 7.69 – 7.65 (m, 2H), 7.45 – 7.40 (m, 2H), 7.39 – 7.31 (m, 3H), 7.18 – 7.14 (m, 2H), 5.91 (s, 1H), 5.46 (s, 1H), 4.38 – 4.27 (m, 2H), 3.65 (s, 3H), 2.43 (s, 3H), 2.37 (s, 3H), 2.28 (d, *J* = 7.0 Hz, 1H), 1.95 (d, *J* = 7.0 Hz, 1H), 1.36 (t, *J* = 7.1 Hz, 3H).

^{13}C NMR (126 MHz, $CDCl_3$): δ_C [ppm] = 169.5, 163.4, 141.9, 141.3, 140.2, 138.1, 129.2, 129.0, 129.0, 126.6, 120.8, 63.2, 53.6, 50.8, 43.2, 34.2, 25.9, 21.6, 14.1, 14.1.

HRMS (ESI$^+$) calc. for $C_{24}H_{27}NNaO_6S_2$ $[M+Na]^+$: 512.1172, found: 512.1153.

9. References

[1] O. V. Dolomanov, L. J. Bourhis, R. J. Gildea, J. A. K. Howard, H. Puschmann, “OLEX2: A Complete Structure Solution, Refinement and Analysis Program”, *J. Appl. Crystallogr.* **2009**, *42*, 339–341.

[2] G. M. Sheldrick, “Crystal Structure Refinement with SHELXL”, *Acta Crystallogr. A* **2015**, *71*, 3–8.

[3] G. M. Sheldrick, SHELXL, Version 2014/7: Program for Crystal Structure Solution and Refinement, Universität Göttingen, Göttingen (Germany) **2014**.

[4] G. M. Sheldrick, “A Short History of SHELX”, *Acta Crystallogr. A* **2008**, *64*, 112–122.

[5] K. Brandenburg, DIAMOND – Crystal and Molecular Structure Visualization, Crystal Impact, Bonn (Germany), http://www.crystalimpact.com/diamond.

[6] Persistence of Vision Pty. Ltd., Persistence of Vision Raytracer, Persistence of Vision Pty. Ltd., **2004**.

[7] F.-L. Haut, C. Habiger, L. A. Wein, K. Wurst, M. Podewitz, T. Magauer, “Rapid Assembly of Tetrasubstituted Furans via Pummerer-Type Rearrangement,” *J. Am. Chem. Soc.* **2021**, *143*, 1216–1223.

[8] F.-L. Haut, N. J. Feichtinger, I. Plangger, L. A. Wein, M. Müller, T.-N. Streit, K. Wurst, M. Podewitz, T. Magauer, “Synthesis of Pyrroles via Consecutive 6π-Electrocyclization/Ring-Contraction of Sulfilimines”, *J. Am. Chem. Soc.* **2021**, *143*, 9002–9008.

[9] M. Gausmann, “Electrosynthesis and Functionalization of Dehydroamino Acids and their Application in the Total Synthesis of Darobactin A”, Dissertation, Freie Universität Berlin, **2023**.

[10] L. Li, S. Müller, R. Petzold, T. Ritter, “Late-Stage Diazoester Installation via Arylthianthrenium Salts”, *Angew. Chem. Int. Ed.* **2025**, *64*, e202419931.

[11] B. Das, B. Sahana, D. P. Hari, “Photoinduced C–Cl Bond Activation of Polychloroalkanes with Triplet Carbenes: Synthetic Applications and Mechanistic Studies”, *JACS Au* **2025**, *5*, 291–301.

[12] W. Xie, C. Wang, J. Xu, “Reaction of 1,3,5-Triazinanes with Phosphoryl Diazomethanes: Access to 5-Phosphoryl-1,2,3,4-Tetrahydropyrimidines”, *Org. Lett.* **2024**, *26*, 3391–3396.

[13] M. Liras, O. García, I. Quijada-Garrido, R. París, “Transformation of the Bromine End Group into Thiol in (Meth)acrylic Polymers Synthesized by Atom Transfer Radical Polymerization”, *Macromolecules* **2011**, *44*, 1335–1339.

[14] W. Kirmse, M. Kapps, “Reaktionen des Diazomethans mit Diallylsulfid und Allylathern unter Kupfersalz-Katalyse”, *Chem. Ber.* **1968**, *101*, 994–1003.

[15] M. P. Doyle, W. H. Tamblyn, V. Bagheri, “Highly Effective Catalytic Methods for Ylide Generation from Diazo Compounds: Mechanism of the Rhodium- and Copper-Catalyzed Reactions with Allylic Compounds”, *J. Org. Chem.* **1981**, *46*, 5094–5102.

[16] K. L. Gutman, C. D. Quintanilla, L. Zhang, “Catalytic Enantioselective Protonation of Gold Enolates Enabled by Cooperative Gold(I) Catalysis”, *J. Am. Chem. Soc.* **2024**, *146*, 3598–3602.

[17] E. Vedejs, T. H. Eberlein, D. L. Varie, “Dienophilic Thioaldehydes”, *J. Am. Chem. Soc.* **1982**, *104*, 1445–1447.

[18] H. Ishibashi, Y. Kitano, H. Nakatani, M. Okada, M. Ikeda, M. Okura, Y. Tamura, “A [2^+ + 4] Polar Cycloaddition of α-Chlorosulfides with Conjugated Dienes: One-Pot Synthesis of 1-Acyl- and 1-Cyano-1-methylthio-2-vinylcyclopropanes.”, *Tetrahedron Lett.* **1984**, *25*, 4231–4232.

[19] H. Ishibashi, M. Okada, H. Nakatani, M. Ikeda, Y. Tamura, “A [2^+ + 4] Polar Cycloaddition of α-Thiocarbocations with 1,3-Dienes: Synthesis and Thermal Reaction of 1-Acyl-1-methylthio-2-vinylcyclopropanes”, *J. Chem. Soc., Perkin Trans. 1* **1986**, *1986*, 1763–1767.

[20] W. H. Midura, A. Rzewnicka, J. A. Krysiak, “Phenylsilane as an effective desulfinylation reagent“, *Beilstein J. Org. Chem*. **2017**, *13*, 1513–1517.

10. X-Ray Crystallographic Data

X-Ray diffraction of 5b (CCDC-2496555)

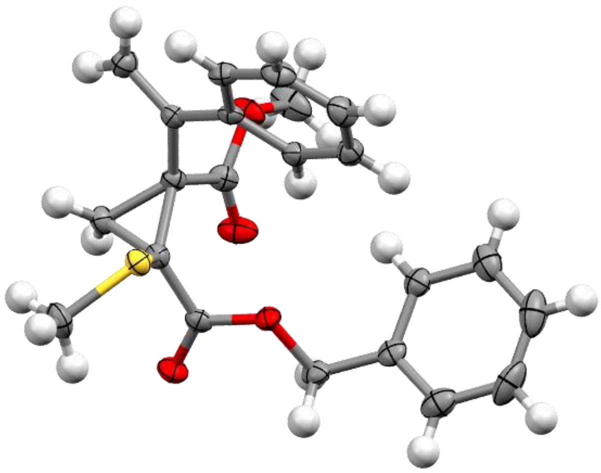

Figure S6. Molecular structure in solid state of $C_{22}H_{22}O_4S$. Ellipsoids are depicted with 50% probability level.

Table S4. Crystal data and structure refinement

Identification code	2496555	
Empirical formula	$C_{22}H_{22}O_4S$	
Formula weight	382.45	
Temperature	150.00 K	
Crystal system	orthorhombic	
Space group	$P2_12_12_1$	
Unit cell dimensions	a = 9.8277(8) Å	α = 90 °.
	b = 10.4363(7) Å	β = 90 °.
	c = 18.6790(13) Å	γ = 90 °.
Volume	1915.8(2) Å^3	
Z	4	
Density ρ (calculated)	1.326 g/cm^3	
Absorption coefficient μ	0.194 mm^{-1}	
F(000)	808.0	
Crystal size	0.555 × 0.488 × 0.356 mm^3	
Radiation	MoKα (λ = 0.71073 Å)	
2Θ range for data collection	4.47 to 52.756 °.	
Index ranges	−10 ≤ h ≤ 12, −13 ≤ k ≤ 13, −23 ≤ l ≤ 23	
Reflections collected	13183	
Independent reflections	3884 [R_{int} = 0.0497, R_{sigma} = 0.0474]	

Data / restraints / parameters	3884/ 0/ 252
Goodness-of-fit on F^2	1.032
Final indexes [I≥2σ (I)]	R_1 = 0.0334, wR_2 = 0.0727
Final R indices [all data]	R_1 = 0.0402, wR_2 = 0.0758
Largest diff. peak/hole	0.18/−0.20 e.$Å^{-3}$
Flack Parameter	−0.06(4)

X-Ray diffraction of 8 (CCDC-2496551)

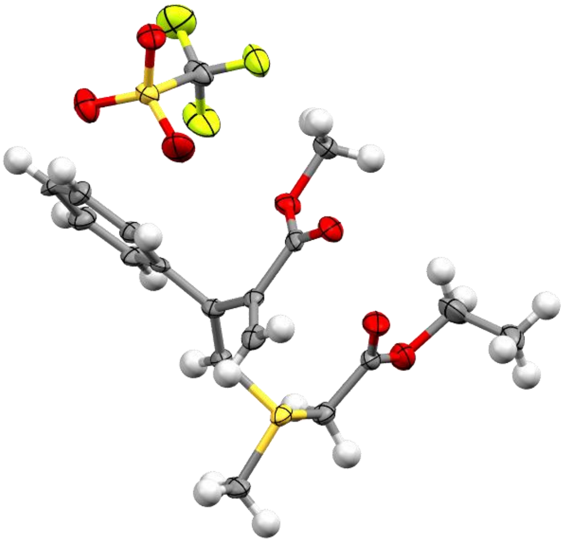

Figure S7. Molecular structure in solid state of $C_{18}H_{21}F_3O_7S_2$. Ellipsoids are depicted with 50% probability level.

Table S5. Crystal data and structure refinement

Identification code	2496551	
Empirical formula	$C_{18}H_{21}F_3O_7S_2$	
Formula weight	470.47	
Temperature	150.00 K	
Crystal system	monoclinic	
Space group	$P2_1$	
Unit cell dimensions	a = 9.7659(8) Å	α = 90 °.
	b = 9.8529(7) Å	β = 111.046(3) °.
	c = 11.6273(10) Å	γ = 90 °.
Volume	1044.17(15) $Å^3$	
Z	2	
Density (calculated)	1.496 g/cm^3	
Absorption coefficient μ	0.319 mm^{-1}	
F(000)	488	

Crystal size	0.142 × 0.378 × 0.754 mm^3
Radiation	MoKα (λ = 0.71073 Å)
2Θ range for data collection	4.47 to 50.74 °.
Index ranges	−11 ≤ h ≤ 10, 0 ≤ k ≤ 11, 0 ≤ l ≤ 14
Reflections collected	2034
Independent reflections	2034 [R_{int} = 0.0707, R_{sigma} = 0.0395]
Data / restraints / parameters	2034/ 1/ 275
Goodness-of-fit on F^2	1.072
Final indexes [I≥2σ (I)]	R_1 = 0.0597, wR_2 = 0.1483
Final R indices [all data]	R_1 = 0.0612, wR_2 = 0.1517
Largest diff. peak and hole	0.55 and -0.73 e.$Å^{-3}$
Flack Parameter	0.02(13)

X-Ray diffraction of 10c (CCDC-2496552)

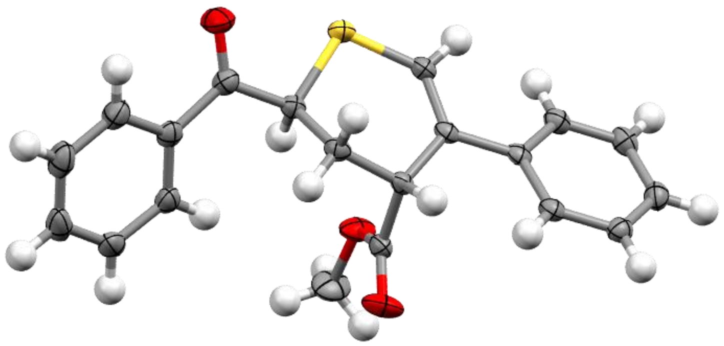

Figure S8. Molecular structure in solid state of $C_{20}H_{18}O_3S$. Ellipsoids are depicted with 50% probability level.

Table S6. Crystal data and structure refinement

Identification code	2496552	
Empirical formula	$C_{20}H_{18}O_3S$	
Formula weight	338.40	
Temperature	150.00 K	
Crystal system	monoclinic	
Space group	$P2_1/c$ (14)	
Unit cell dimensions	a = 13.3915(17) Å	α = 90 °.
	b = 7.7673(8) Å	β = 110.716 °.
	c = 16.9770(19) Å	γ = 90 °.
Volume	1651.7(3) $Å^3$	
Z	4	
Density (calculated)	1.361 g/cm^3	

Absorption coefficient μ	0.221 mm^{-1}
F(000)	712
Crystal size	0.16 × 0.411 × 0.719 mm^3
Radiation	MoKα (λ = 0.71073 Å)
2Θ range for data collection	5.01 to 50.84 °.
Index ranges	−16 ≤ h ≤ 16, −9 ≤ k ≤ 9, −20 ≤ l ≤ 20
Reflections collected	26090
Independent reflections	3042 [R_{int} = 0.0759, R_{sigma} = 0.0357]
Data / restraints / parameters	3042/ 0/ 218
Goodness-of-fit on F^2	1.059
Final indexes [I≥2σ (I)]	R_1 = 0.0361, wR_2 = 0.0858
Final R indices [all data]	R_1 = 0.0497, wR_2 = 0.0927
Largest diff. peak/hole	0.23/−0.24 e.$Å^{-3}$

X-Ray diffraction of 12a (CCDC-2496553)

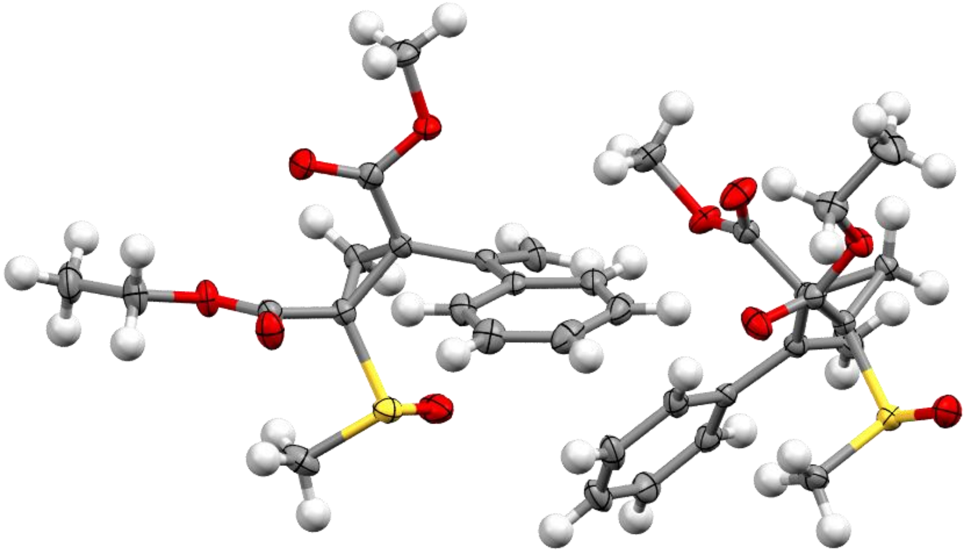

Figure S9. Molecular structure in solid state of $C_{34}H_{40}O_{10}S_2$. Ellipsoids are depicted with 50% probability level.

Table S7. Crystal data and structure refinement

Identification code	2496553
Empirical formula	$C_{34}H_{40}O_{10}S_2$ (2 × $C_{17}H_{20}O_5S$)
Formula weight	672.78
Temperature	150.00 K
Crystal system	monoclinic
Space group	$P2_1/c$

Unit cell dimensions	a = 7.7524(3) Å	α = 90 °.
	b = 8.6579(4) Å	β = 91.671 °.
	c = 49.025(2) Å	γ = 90 °.
Volume	3289.2(2) $Å^3$	
Z	4	
Density ρ (calculated)	1.359 g/cm^3	
Absorption coefficient μ	0.220 mm^{-1}	
F(000)	1424.0	
Crystal size	0.9 × 0.7 × 0.443 mm^3	
Radiation	MoKα (λ = 0.71073 Å)	
2Θ range for data collection	4.478 to 50.67°.	
Index ranges	$-8 \leq h \leq 9$, $-10 \leq k \leq 10$, $-58 \leq l \leq 58$	
Reflections collected	40169	
Independent reflections	5994 [R_{int} = 0.0579, R_{sigma} = 0.0346]	
Data / restraints / parameters	5994/ 0/ 421	
Goodness-of-fit on F^2	1.073	
Final indexes [I≥2σ (I)]	R_1 = 0.0349, wR_2 = 0.0873	
Final R indices [all data]	R_1 = 0.0411, wR_2 = 0.0932	
Largest diff. peak/hole	0.34/−0.32 e.$Å^{-3}$	

X-Ray diffraction of 12e (CCDC-2496554)

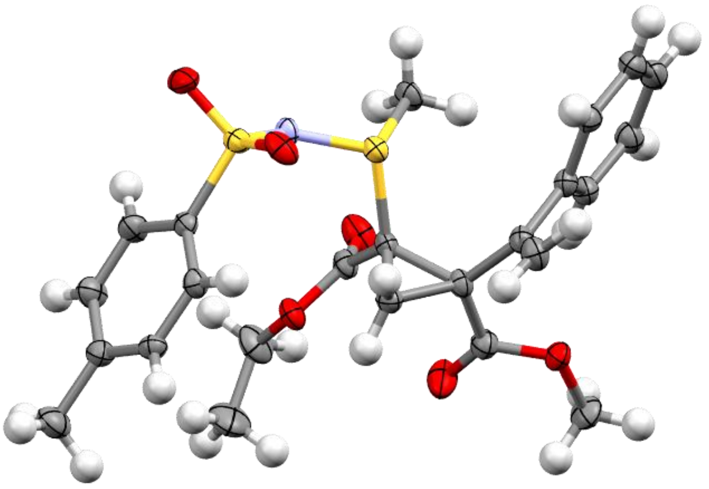

Figure S10. Molecular structure in solid state of $C_{24}H_{27}NO_6S_2$. Ellipsoids are depicted with 50% probability level.

Table S8. Crystal data and structure refinement

Identification code	2496554
Empirical formula	$C_{24}H_{28}NO_6S_2$

Formula weight	490.59	
Temperature	100.00 K	
Crystal system	orthorhombic	
Space group	$Pna2_1$	
Unit cell dimensions	a = 14.6321(5)Å	α = 90°.
	b = 20.9779(6)Å	β = 90°.
	c = 8.0134(2) Å	γ = 90°.
Volume	2459.72(13) $Å^3$	
Z	4	
Density ρ (calculated)	1.325 g/cm^3	
Absorption coefficient μ	0.256 mm^{-1}	
F(000)	1036.0	
Crystal size	0.628 × 0.304 × 0.304 mm^3	
Radiation	MoKα (λ = 0.71073 Å)	
2Θ range for data collection	4.4778 to 50.68°.	
Index ranges	$-17 \le h \le 17$, $-25 \le k \le 25$, $-9 \le l \le 9$	
Reflections collected	35179	
Independent reflections	4515 [R_{int} = 0.0556, R_{sigma} = 0.0267]	
Data / restraints / parameters	4515/ 1/ 309	
Goodness-of-fit on F^2	1.045	
Final indexes [I≥2σ (I)]	R_1 = 0.0303, wR_2 = 0.0753	
Final R indices [all data]	R_1 = 0.0358, wR_2 = 0.0799	
Largest diff. peak/hole	0.55/-0.37 e.$Å^{-3}$	
Flack Parameter	0.30(2)	

11. NMR Spectra

Methyl 4-(4-methoxyphenyl)-2,5-dihydrothiophene-3-carboxylate (S-9b)

^{1}H NMR (500 MHz, $CDCl_3$)

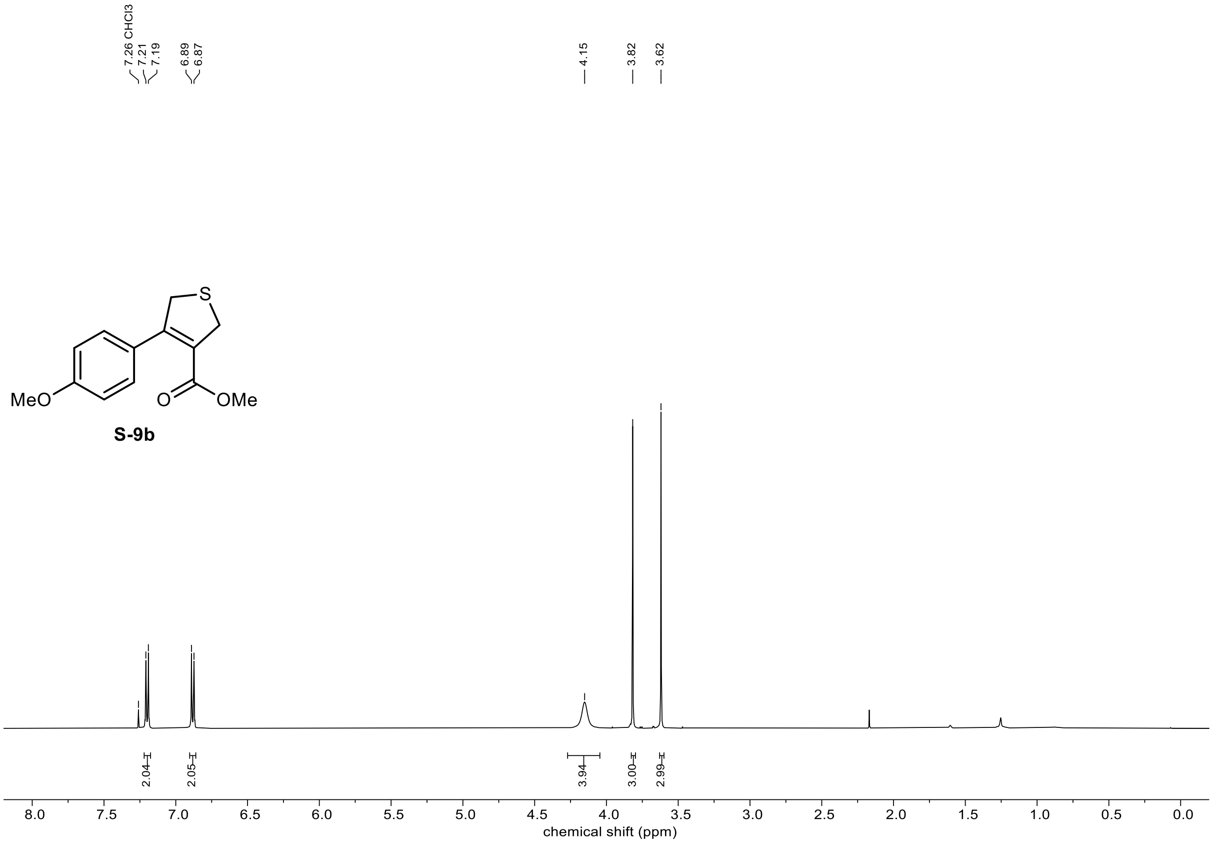

^{13}C NMR (126 MHz, $CDCl_3$)

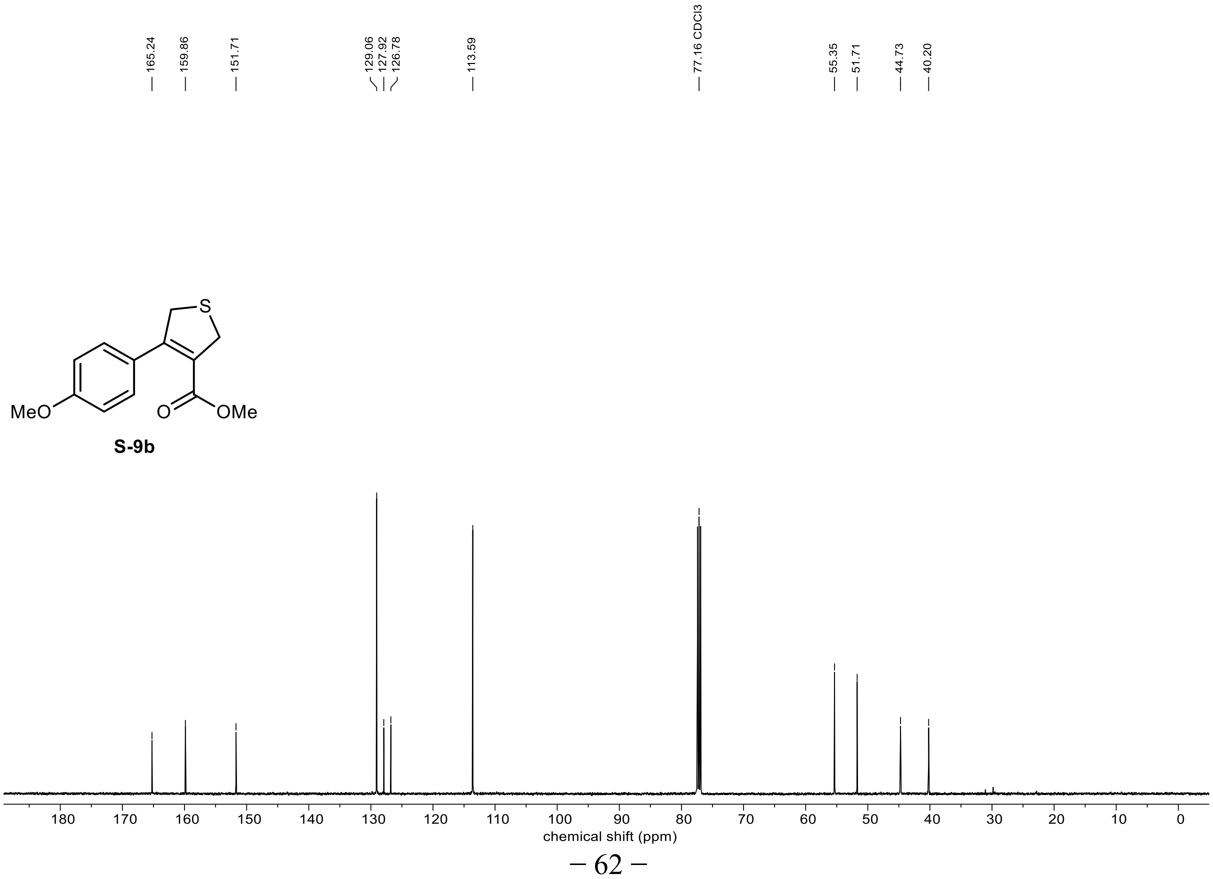

Methyl 4-(4-(trifluoromethyl)phenyl)-2,5-dihydrothiophene-3-carboxylate (S-9c)

^{1}H NMR (500 MHz, $CDCl_3$)

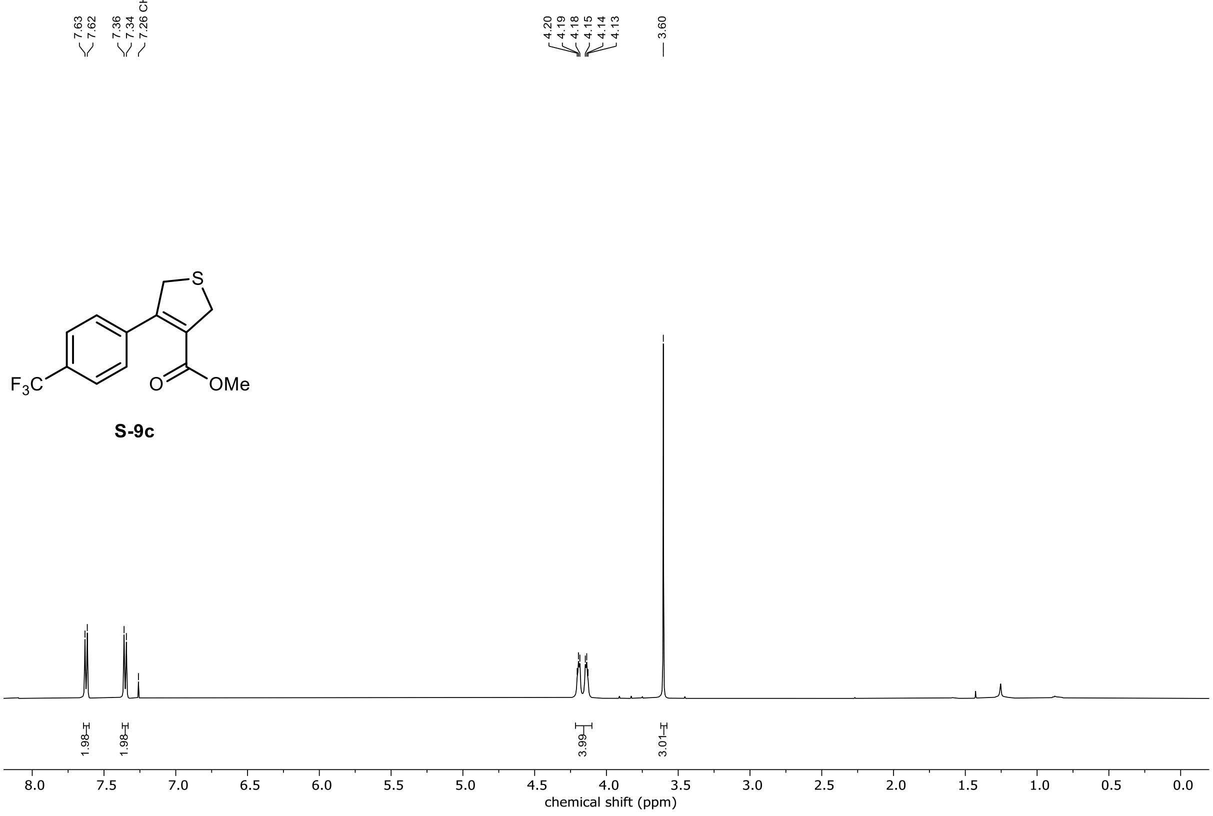

^{13}C NMR (126 MHz, $CDCl_3$)

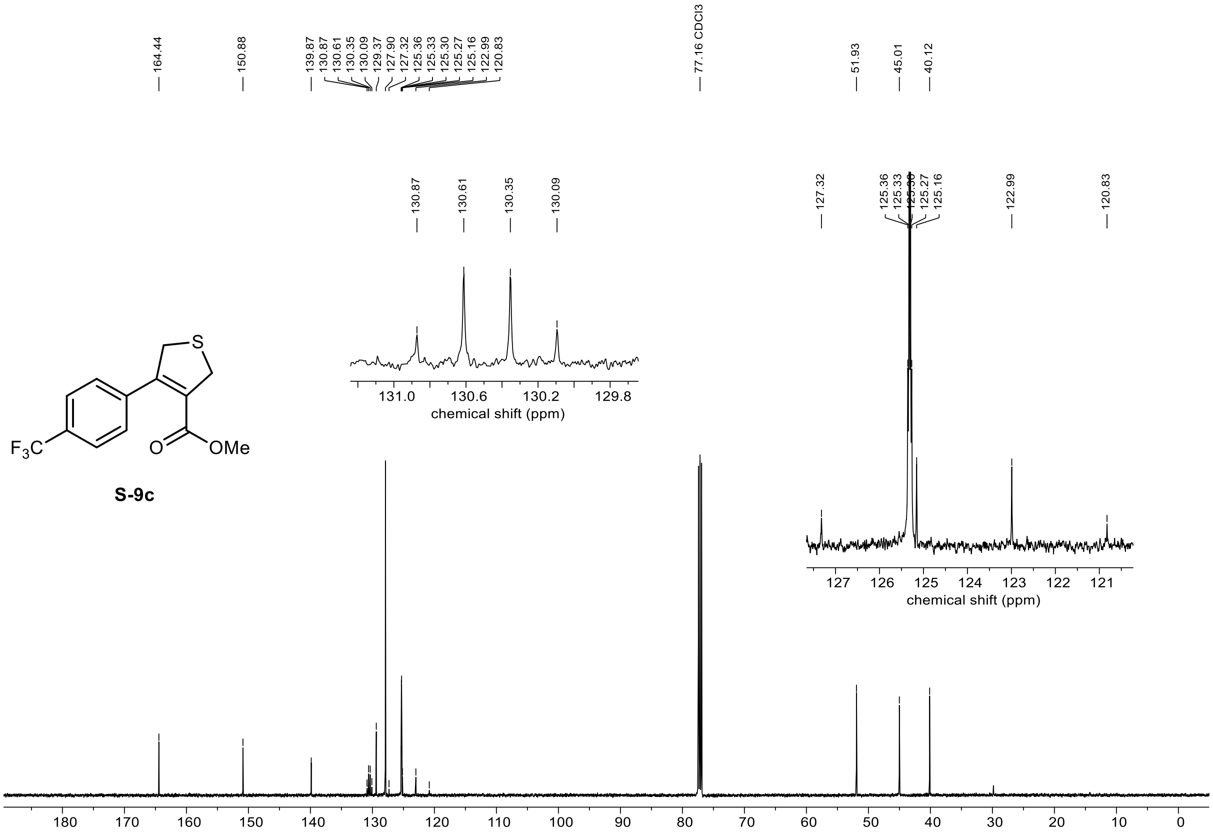

^{19}F NMR (376 MHz, $CDCl_3$)

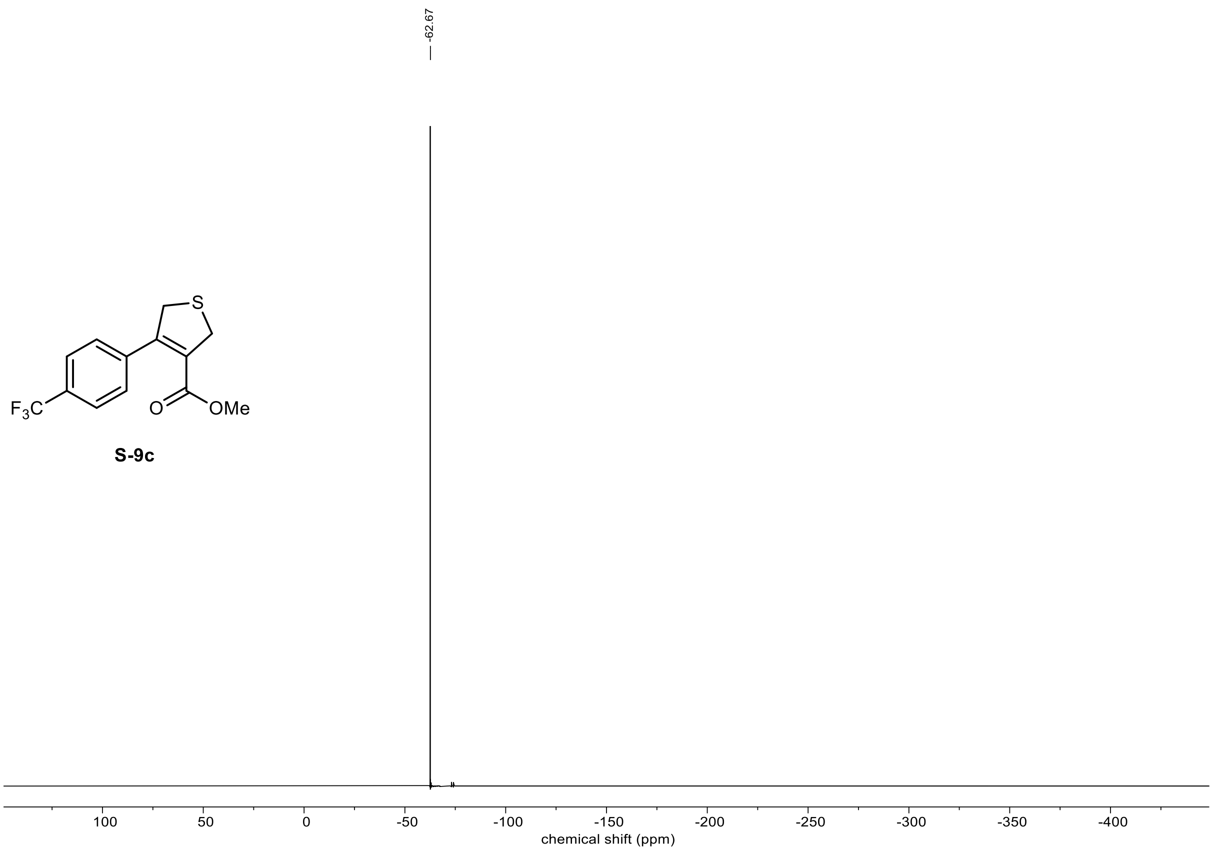

Methyl 4-(4-(ethoxycarbonyl)phenyl)-2,5-dihydrothiophene-3-carboxylate (S-9f)

^{1}H NMR (500 MHz, $CDCl_3$)

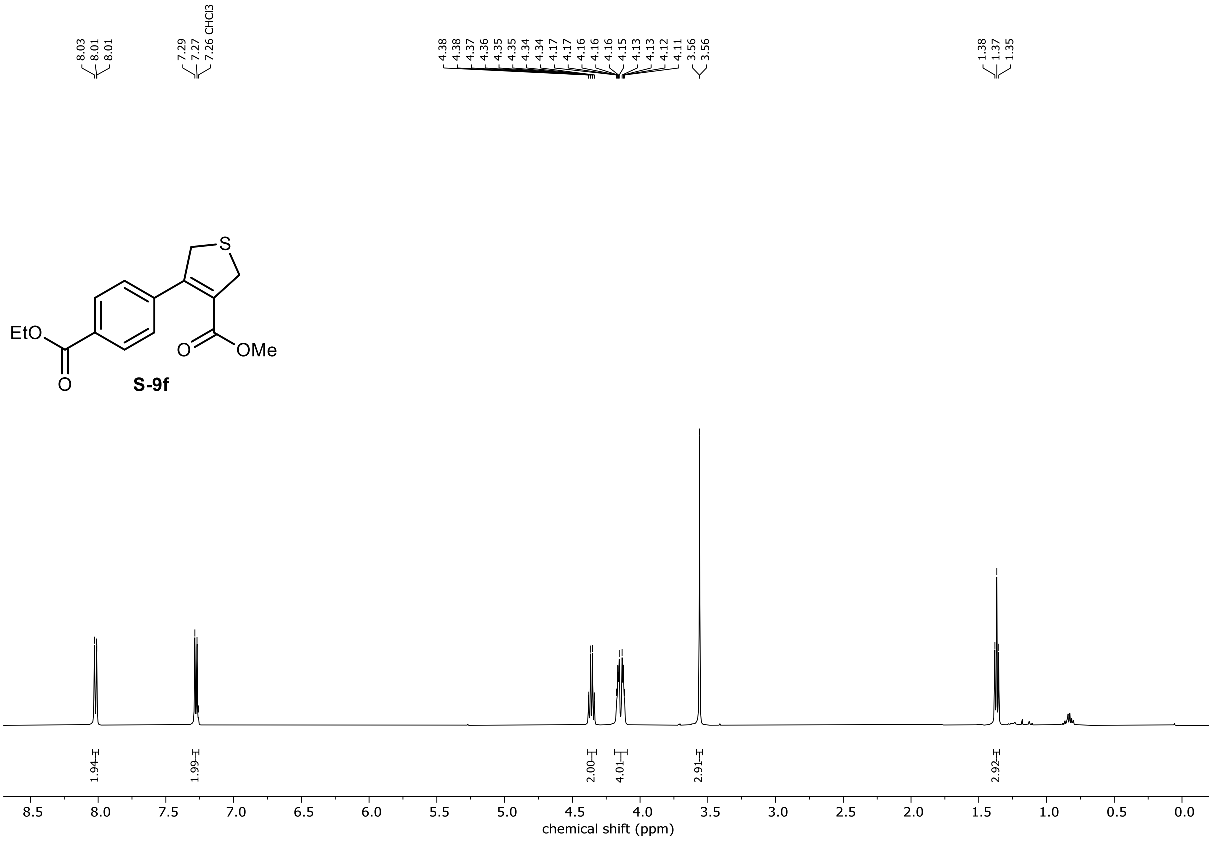

^{13}C NMR (126 MHz, $CDCl_3$)

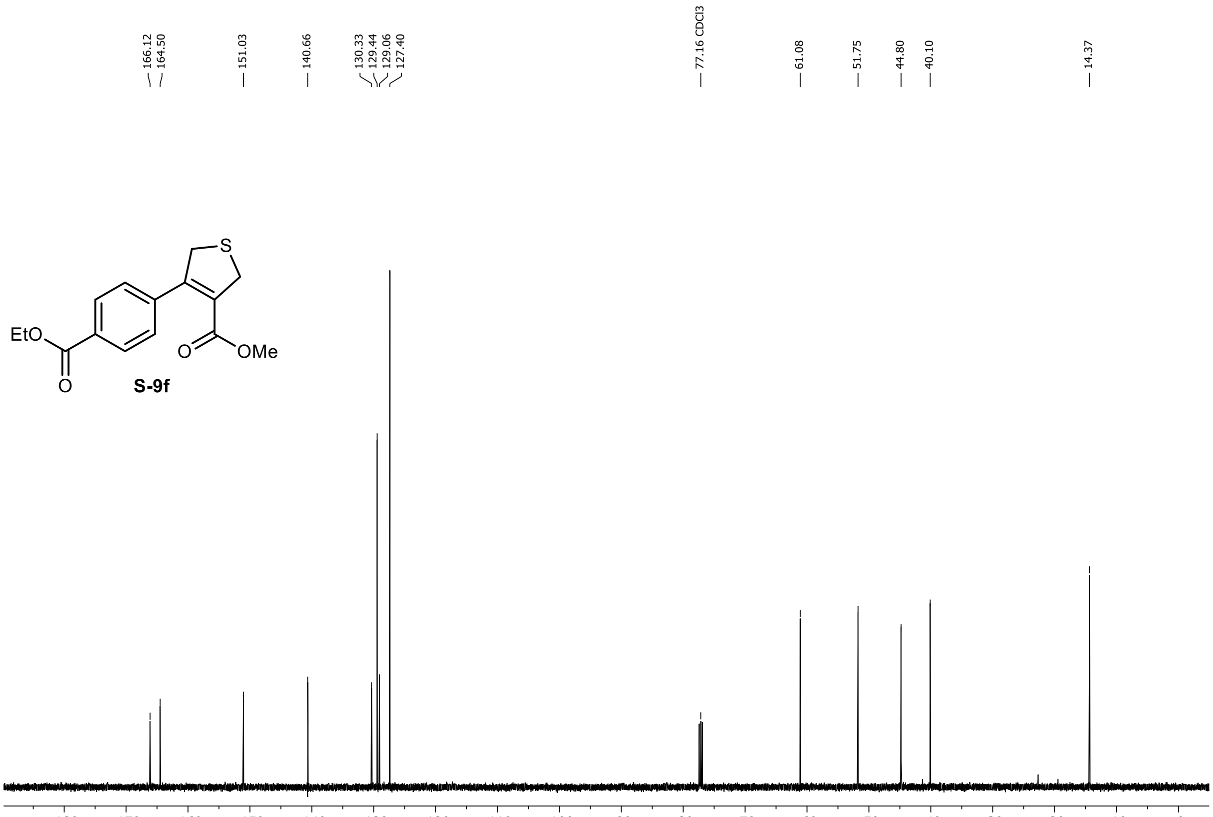

***Tert*-butyl 5-(4-(methoxycarbonyl)-2,5-dihydrothiophen-3-yl)-1*H*-indole-1-carboxylate (S-9k)**

^{1}H NMR (500 MHz, $CDCl_3$)

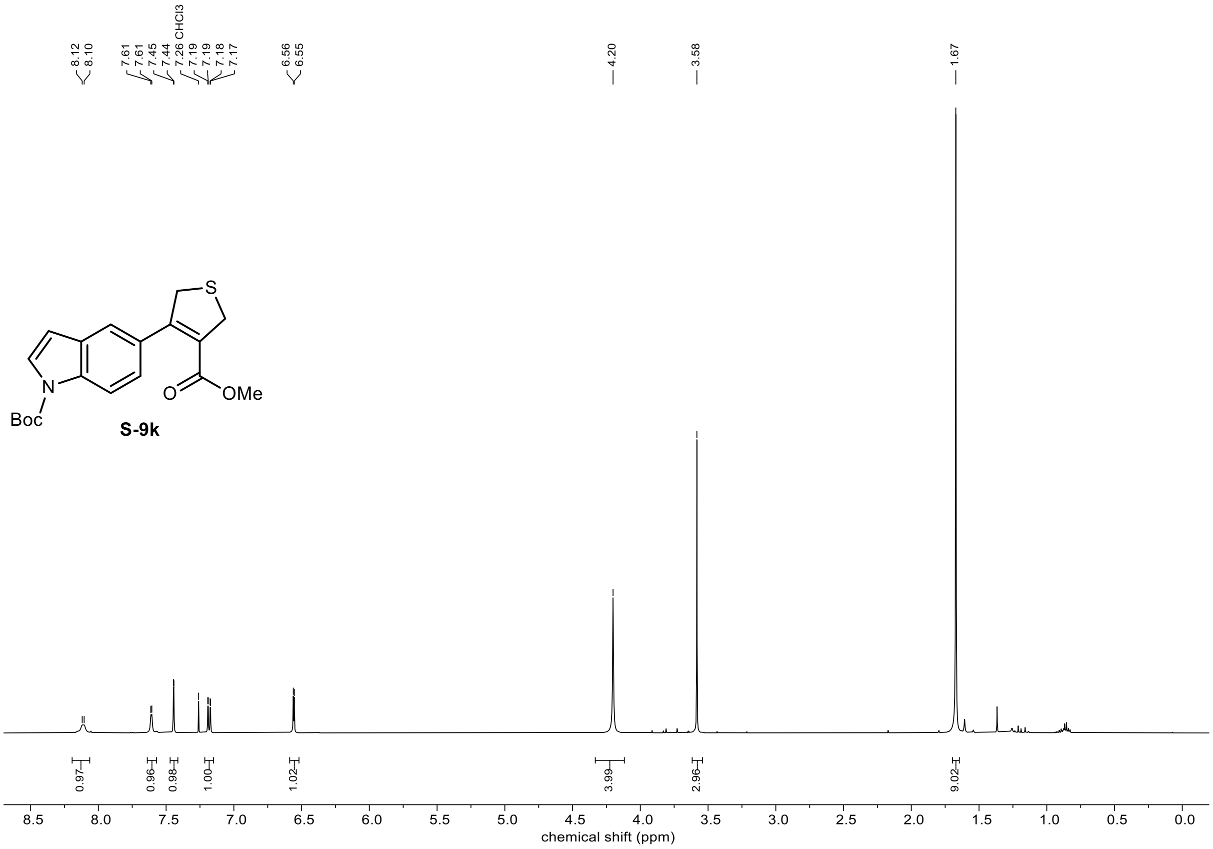

^{13}C NMR (126 MHz, $CDCl_3$)

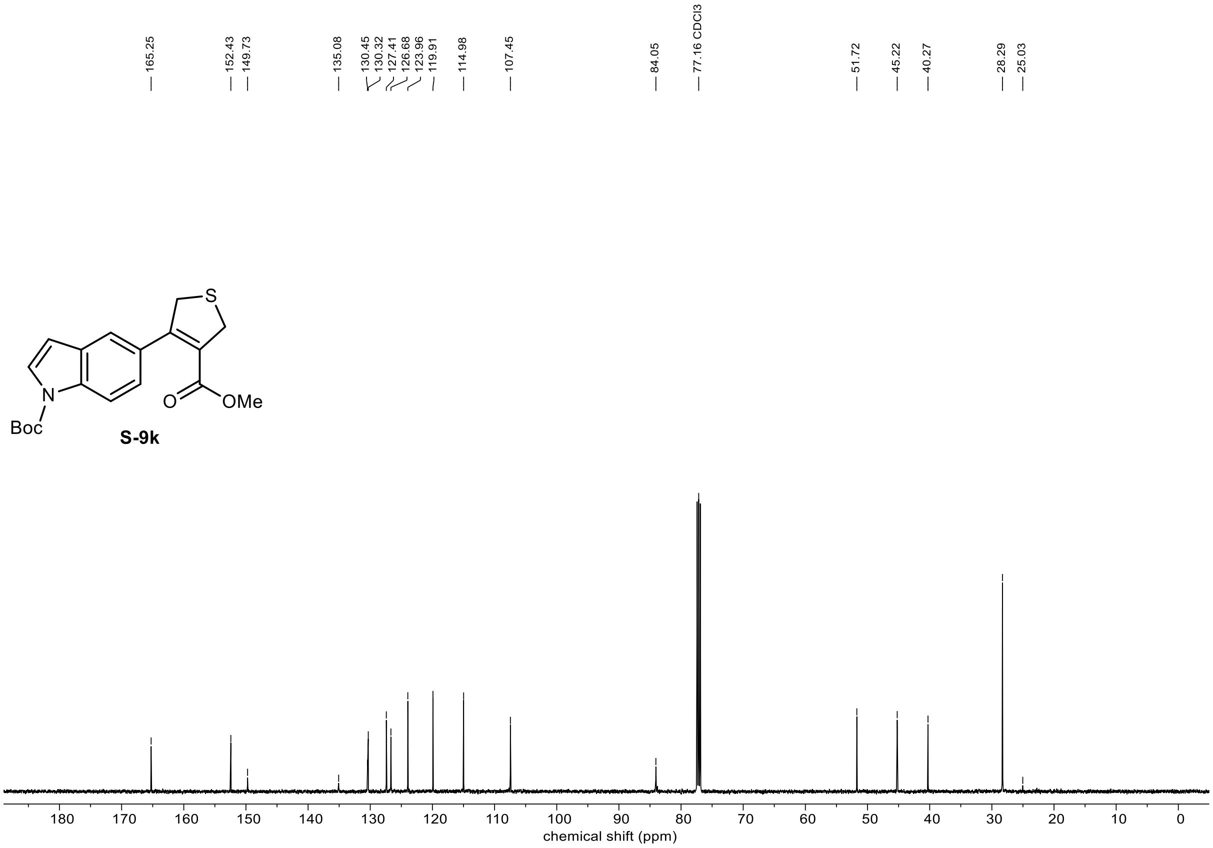

Methyl 4-((triisopropylsilyl)oxy)-2,5-dihydrothiophene-3-carboxylate (S-9m)

^{1}H NMR (500 MHz, $CDCl_3$)

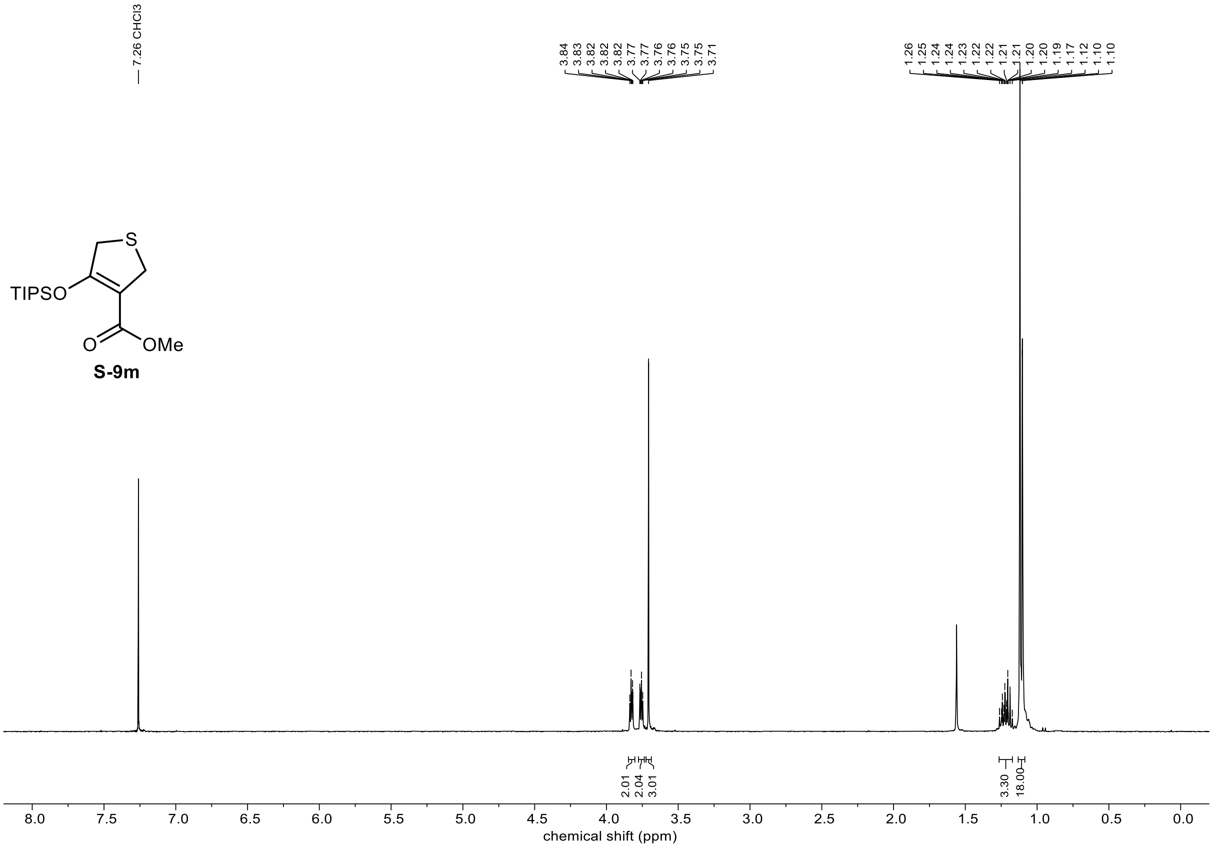

^{13}C NMR (126 MHz, $CDCl_3$)

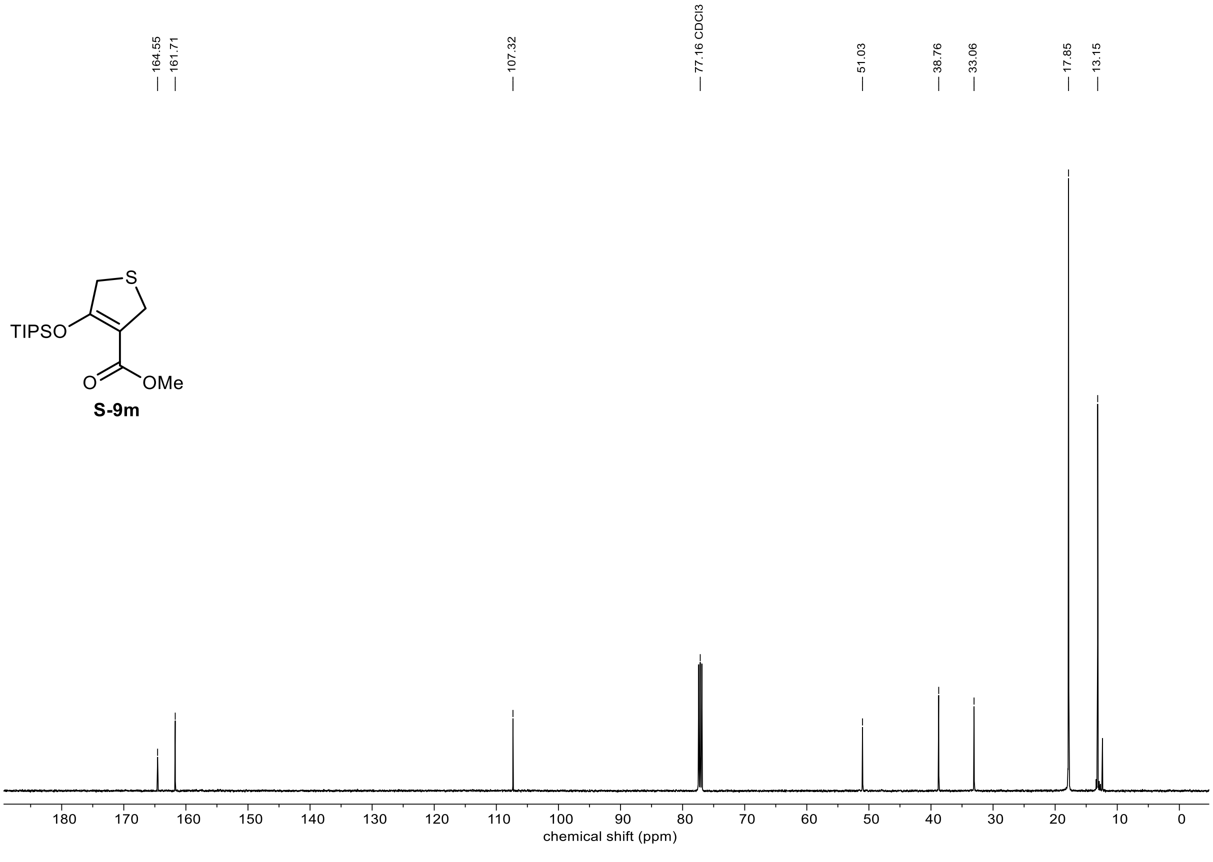

Methyl 4-hydroxy-5-methyl-2,5-dihydrothiophene-3-carboxylate (S-9q)

^{1}H NMR (500 MHz, $CDCl_3$)

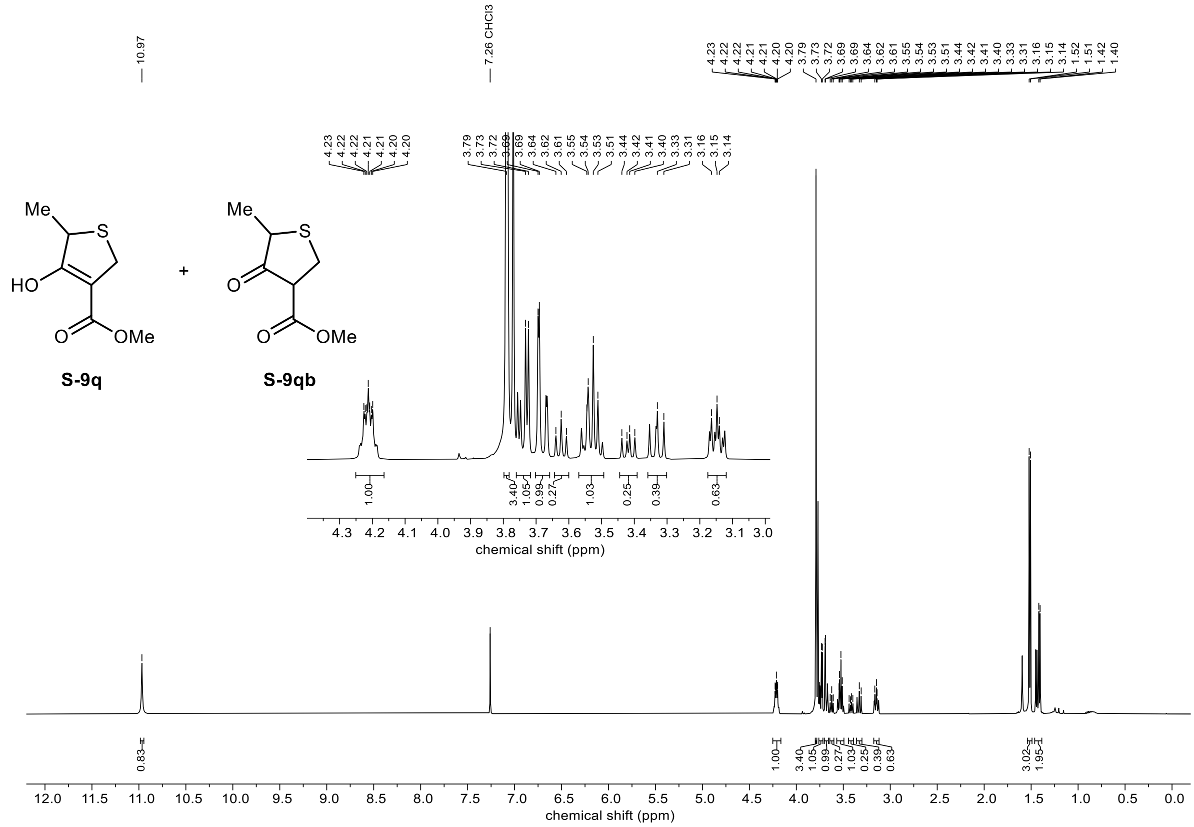

^{13}C NMR (126 MHz, $CDCl_3$)

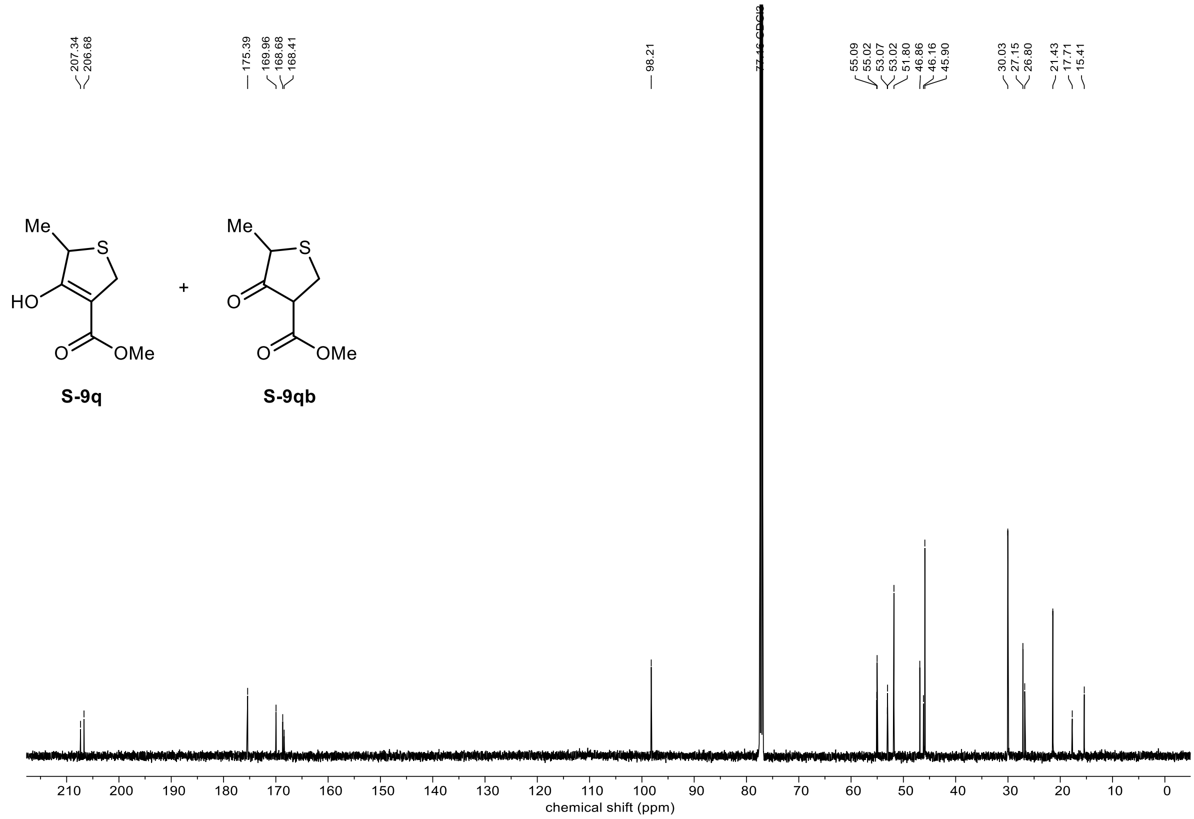

Methyl 5-methyl-4-(((trifluoromethyl)sulfonyl)oxy)-2,5-dihydrothiophene-3-carboxylate (S-9p)

^{1}H NMR (500 MHz, $CDCl_3$)

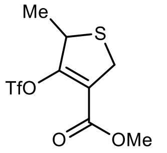

S-9p

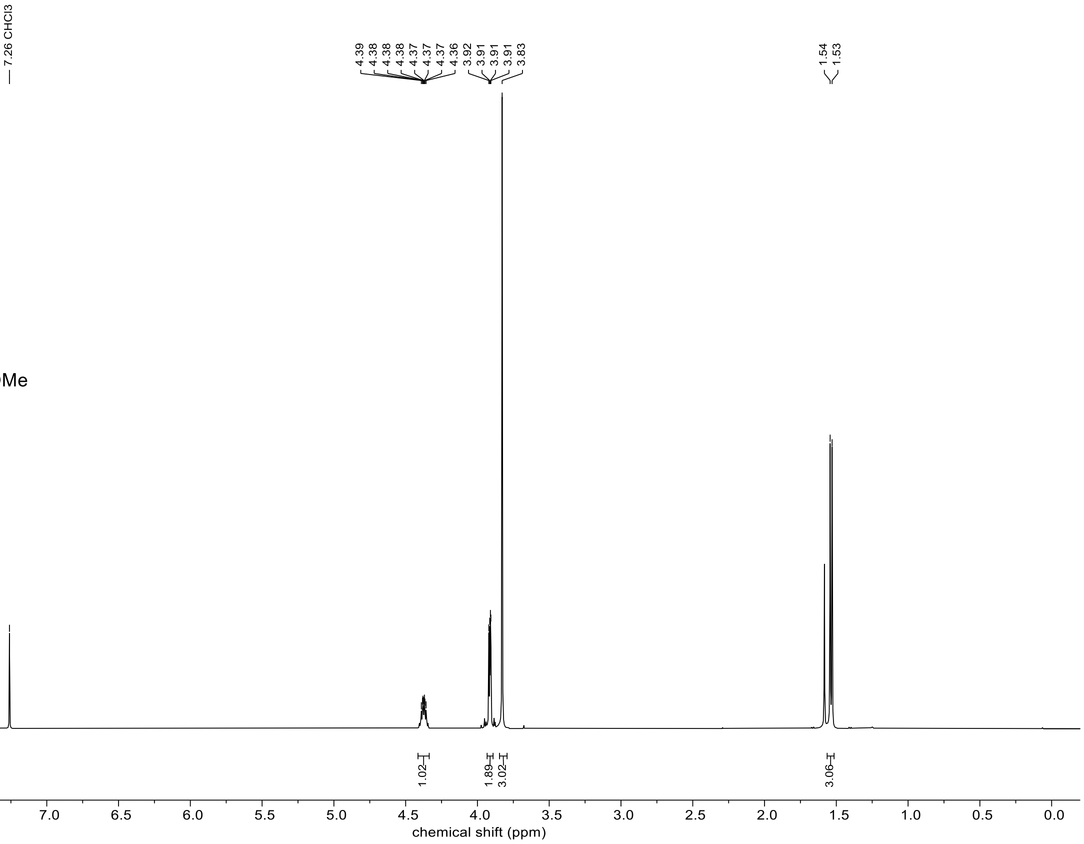

^{13}C NMR (126 MHz, $CDCl_3$)

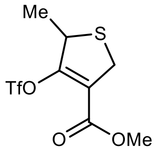

S-9p

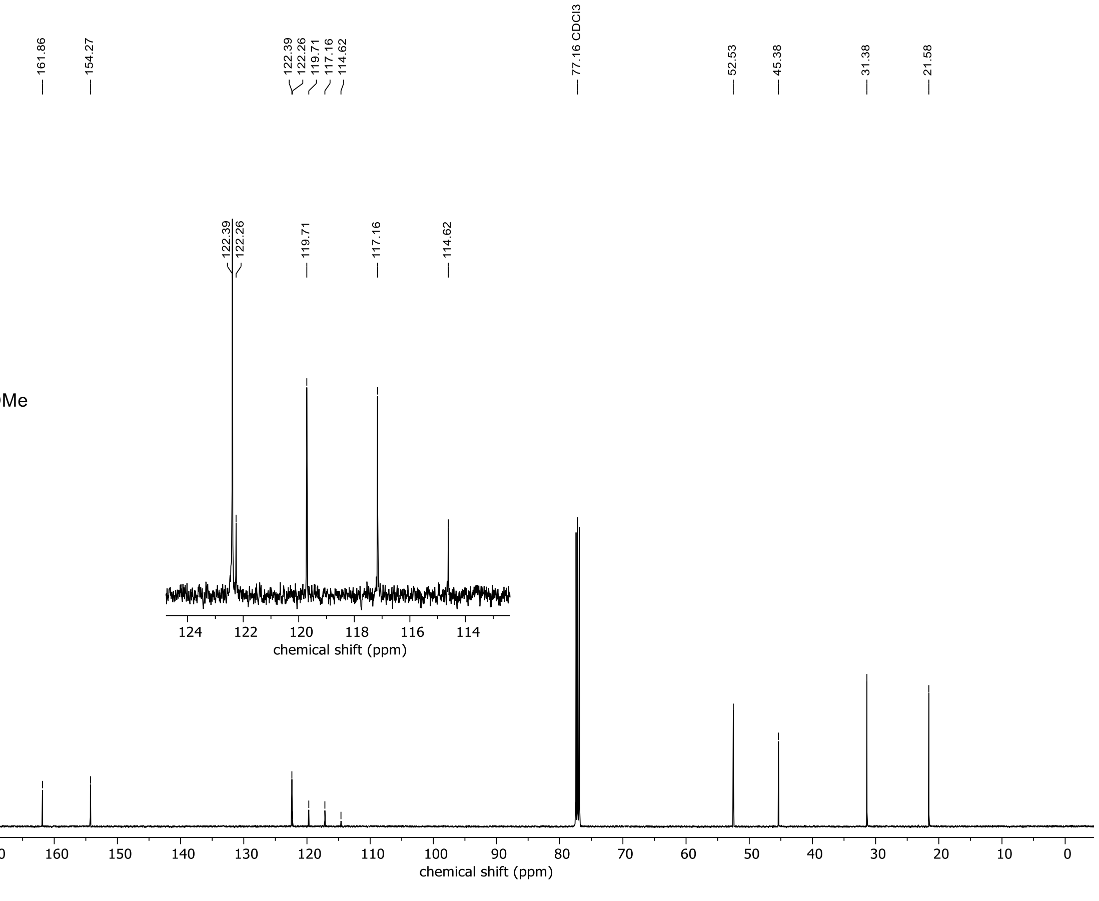

^{19}F NMR (376 MHz, $CDCl_3$)

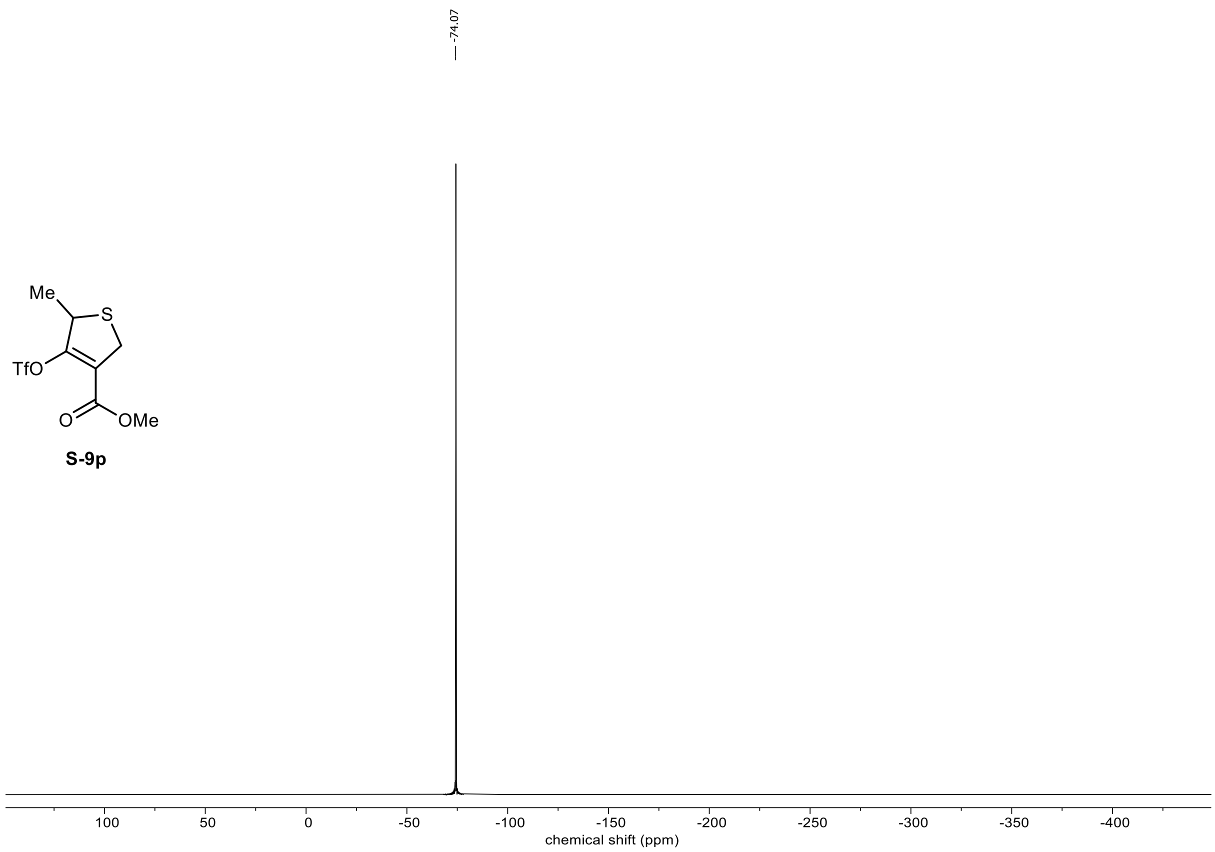

Methyl 5-methyl-4-phenyl-2,5-dihydrothiophene-3-carboxylate (S-9o)

^{1}H NMR (500 MHz, $CDCl_3$)

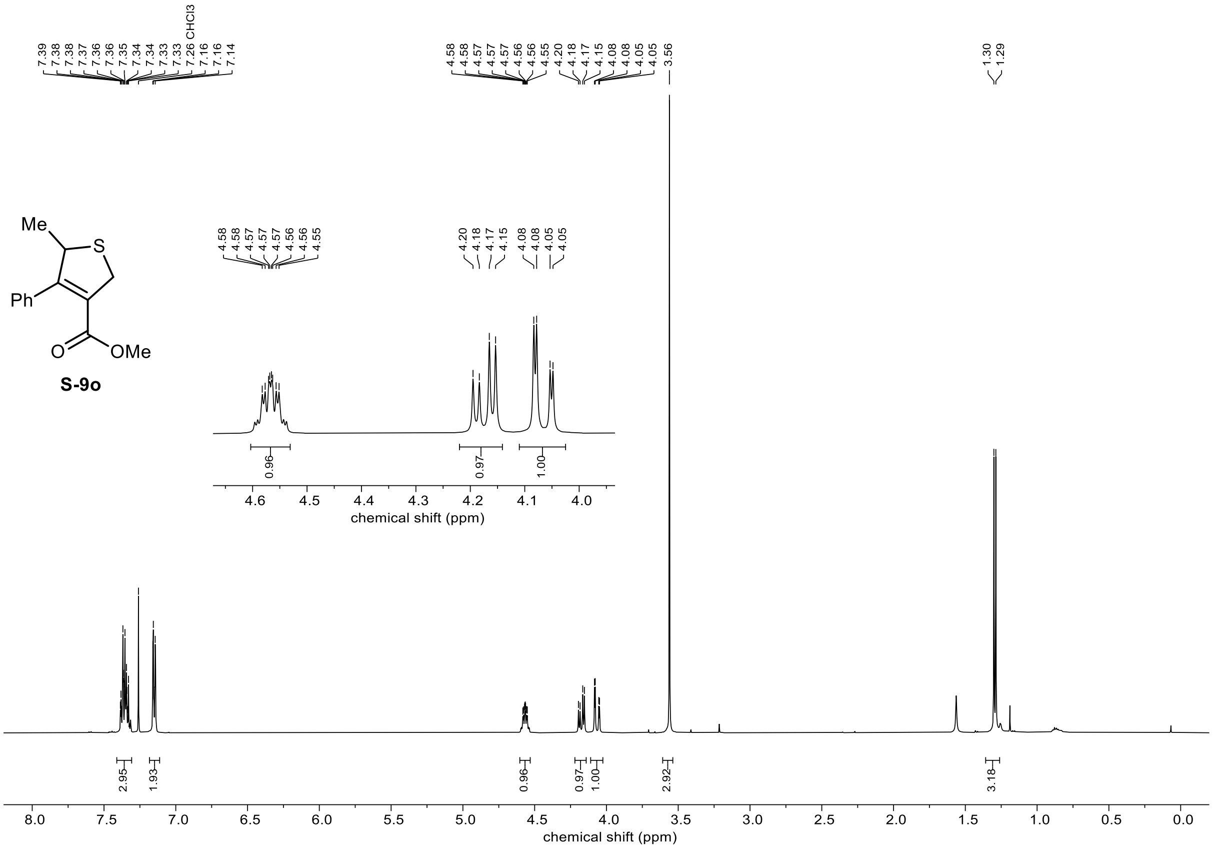

^{13}C NMR (126 MHz, $CDCl_3$)

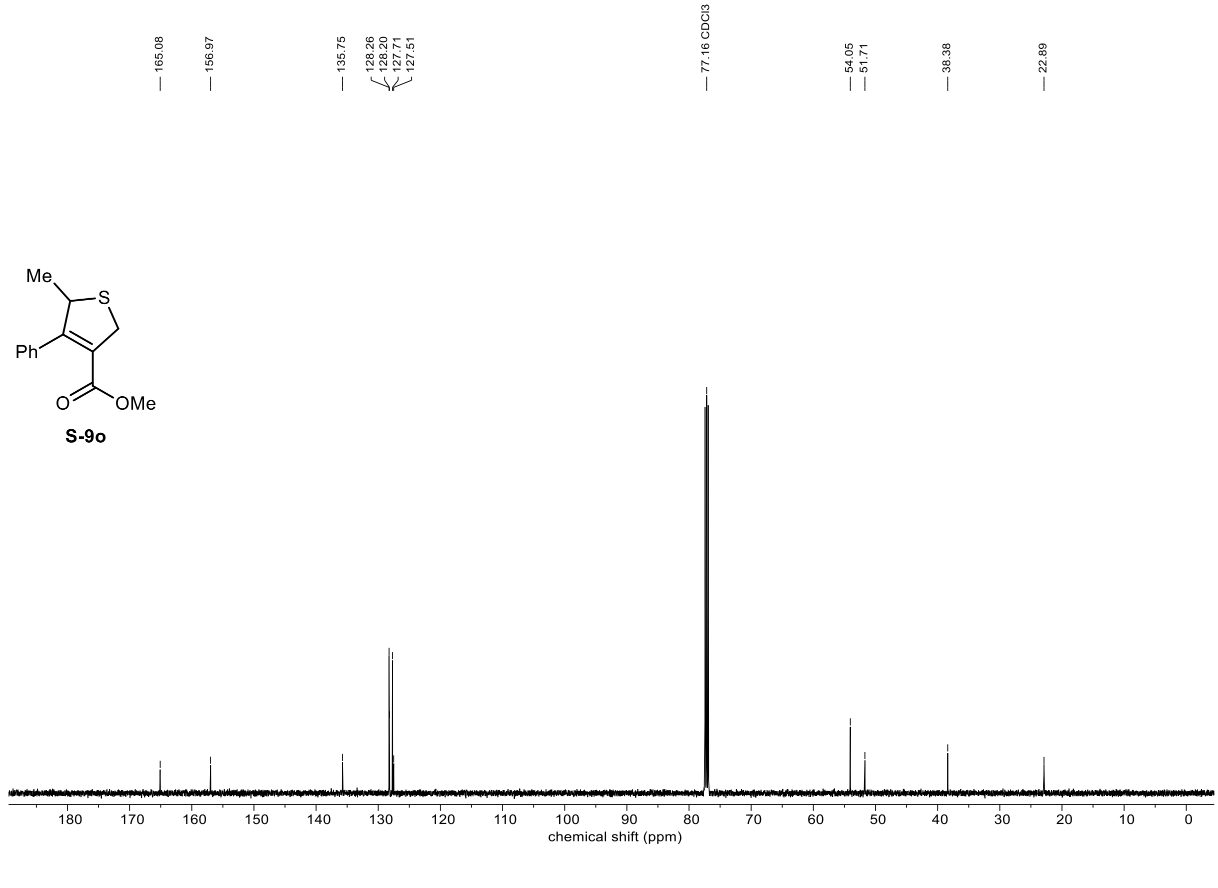

Methyl (*Z*)-3-(4-methoxyphenyl)-2-methylene-4-(methylthio)but-3-enoate (S-1b)

^{1}H NMR (500 MHz, $CDCl_3$)

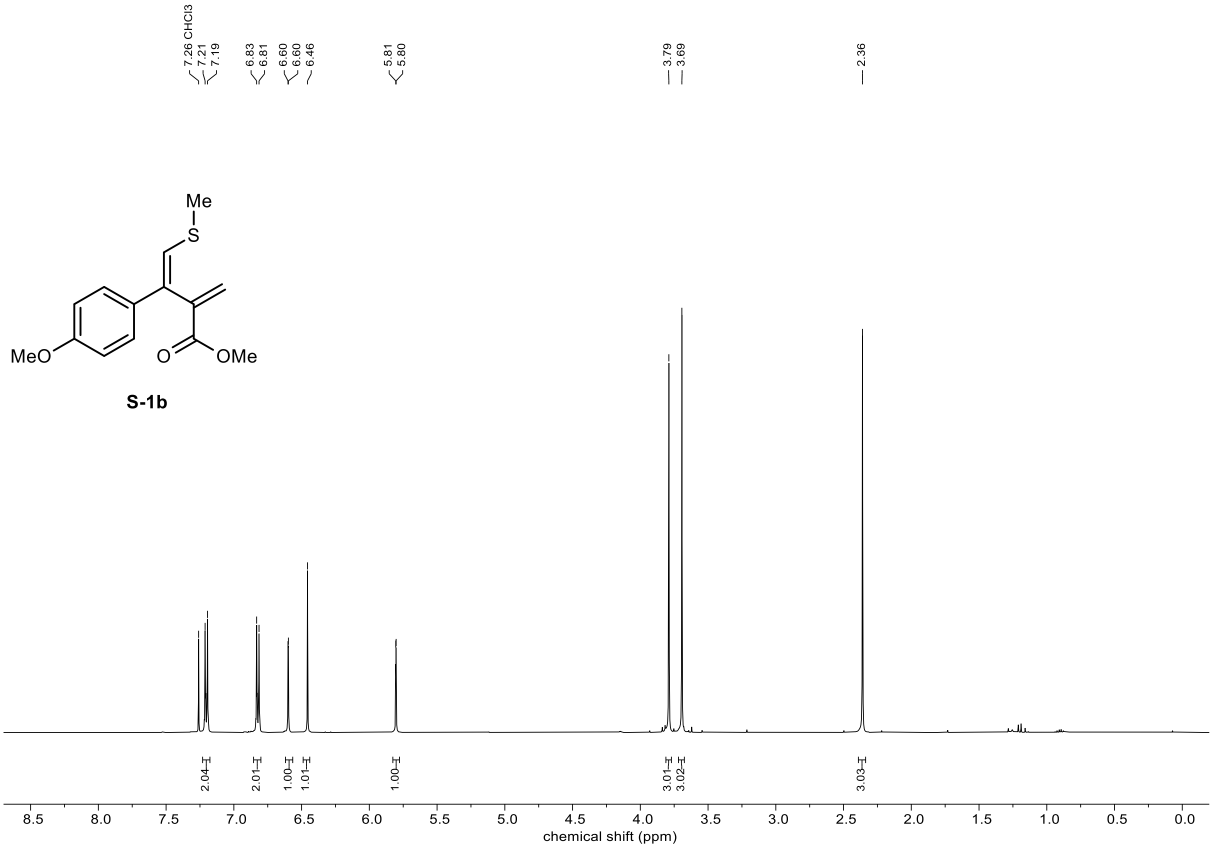

^{13}C NMR (126 MHz, $CDCl_3$)

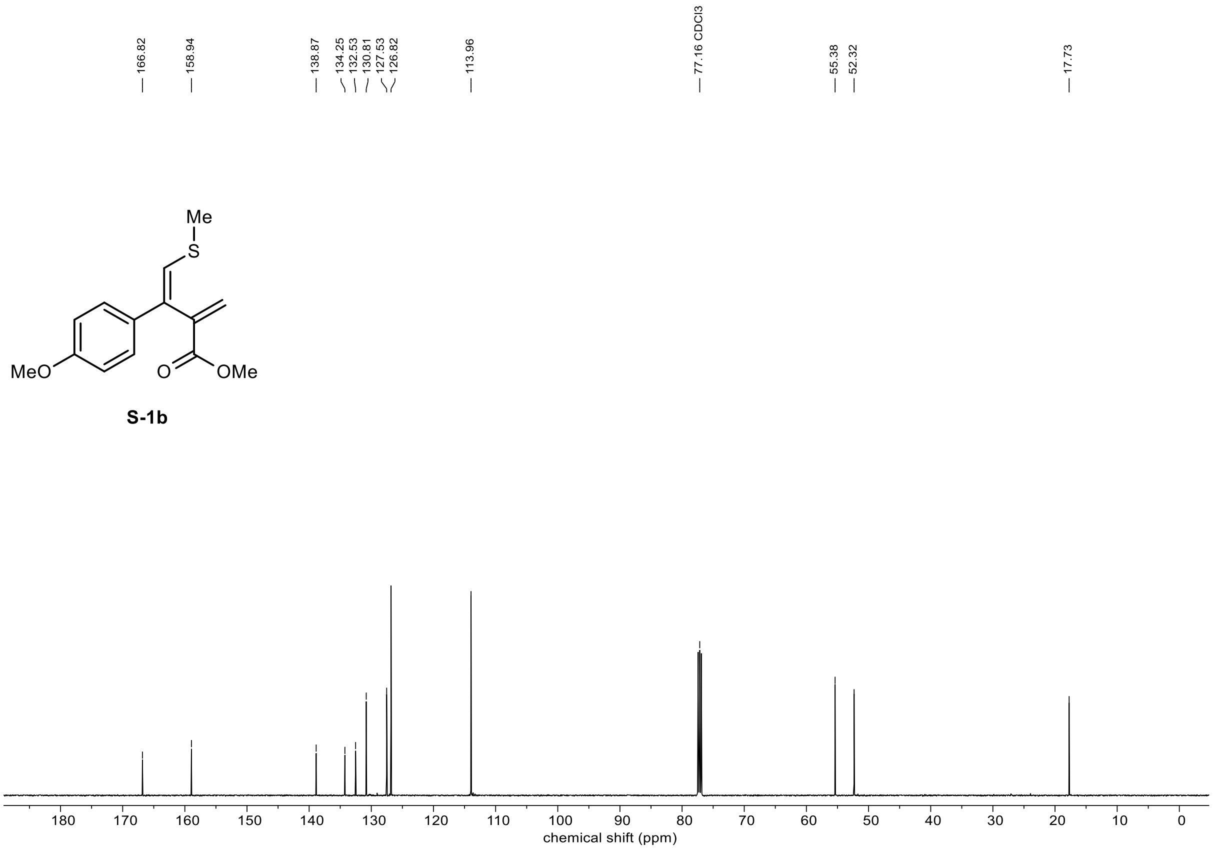

Methyl (*Z*)-2-methylene-4-(methylthio)-3-(4-(trifluoromethyl)phenyl)but-3-enoate (S-1c)

^{1}H NMR (500 MHz, $CDCl_3$)

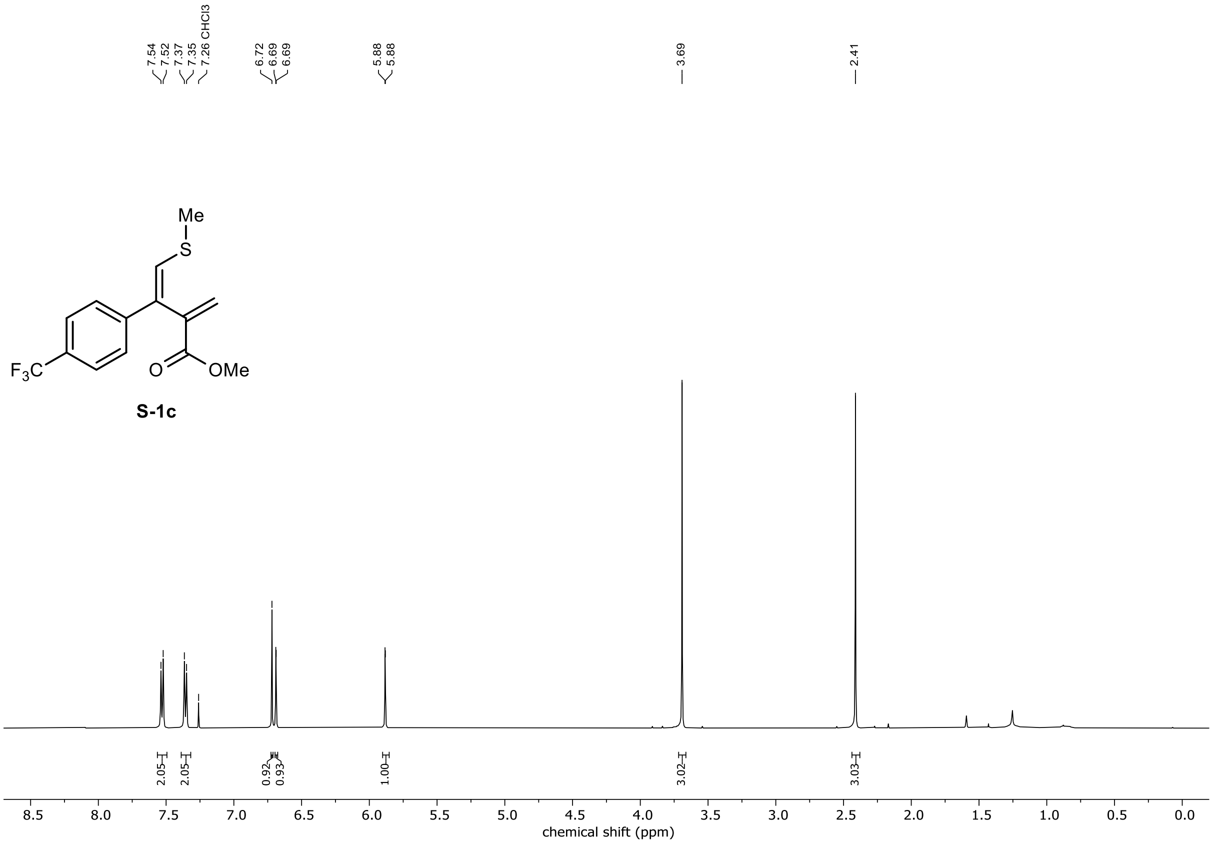

^{13}C NMR (126 MHz, $CDCl_3$)

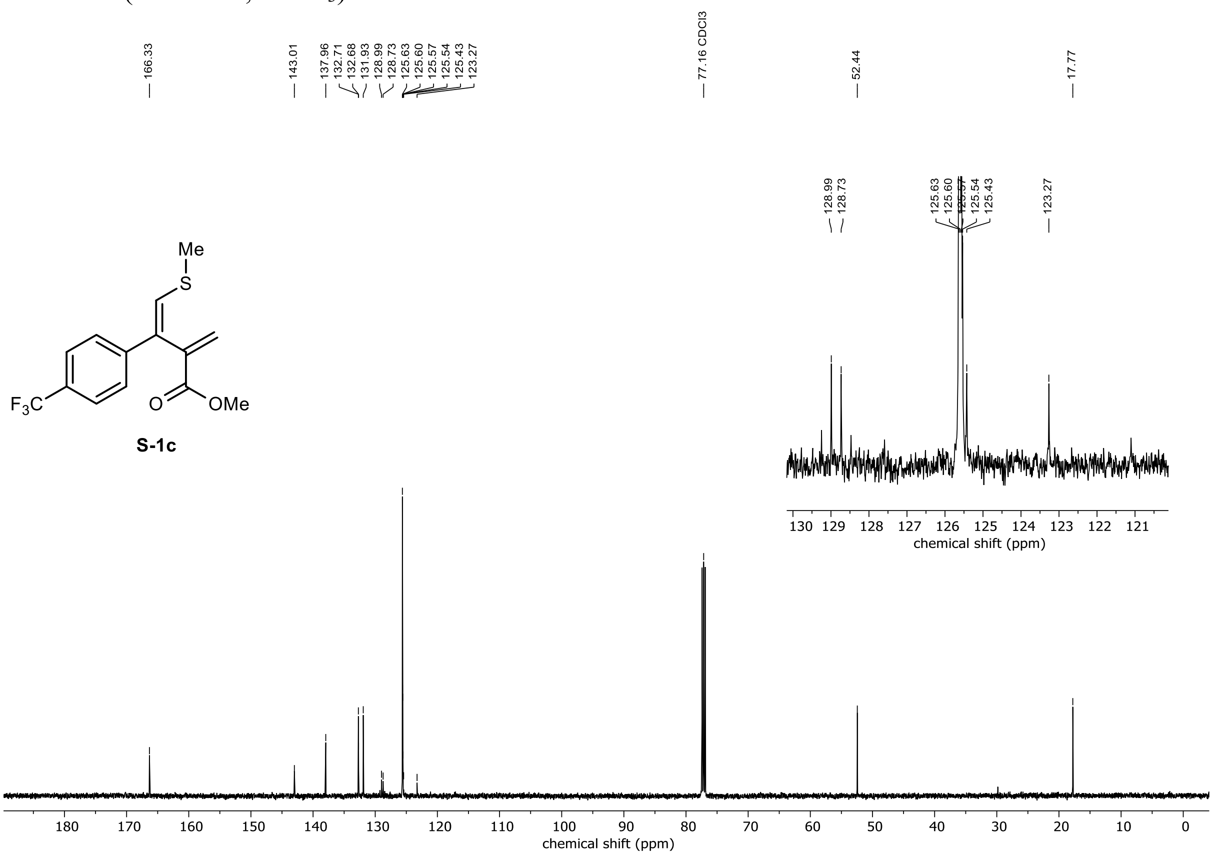

^{19}F NMR (376 MHz, $CDCl_3$)

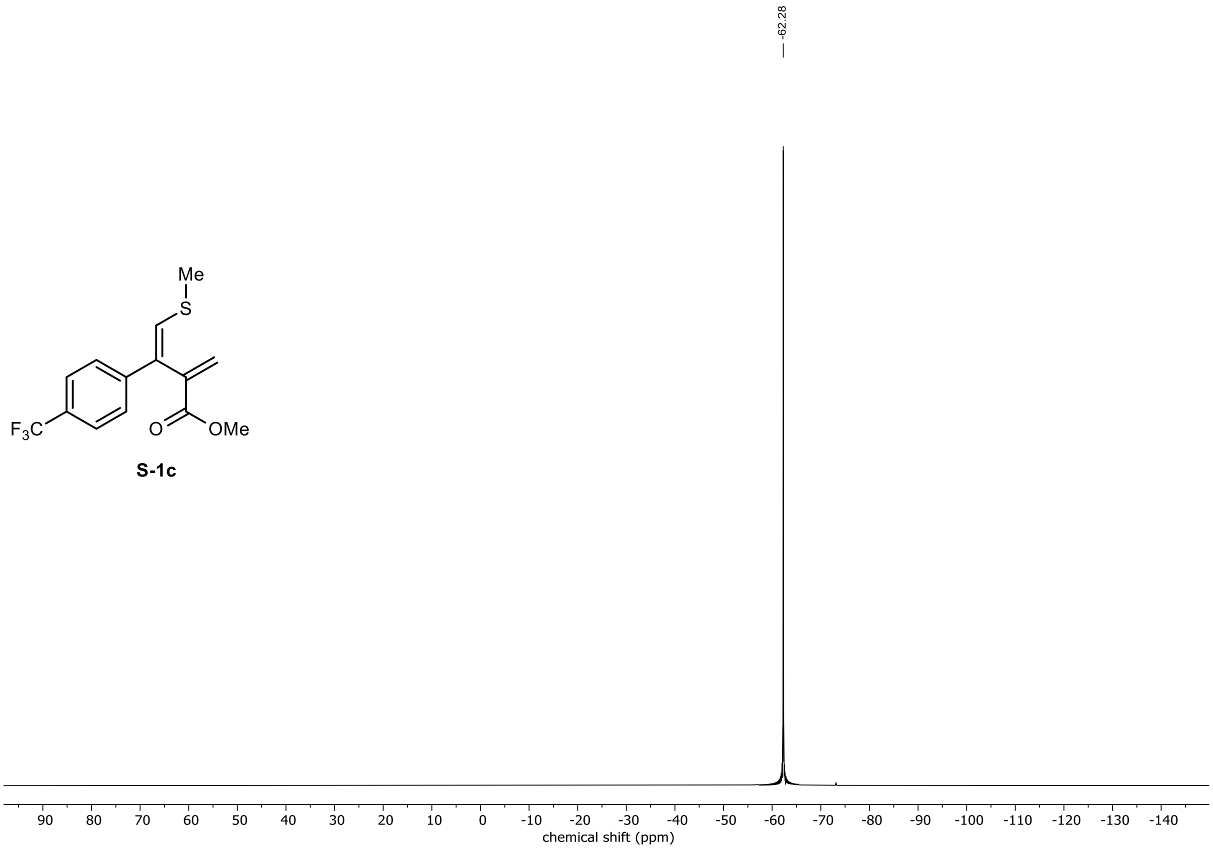

Ethyl (*Z*)-4-(3-(methoxycarbonyl)-1-(methylthio)buta-1,3-dien-2-yl)benzoate (S-1f)

^{1}H NMR (500 MHz, $CDCl_3$)

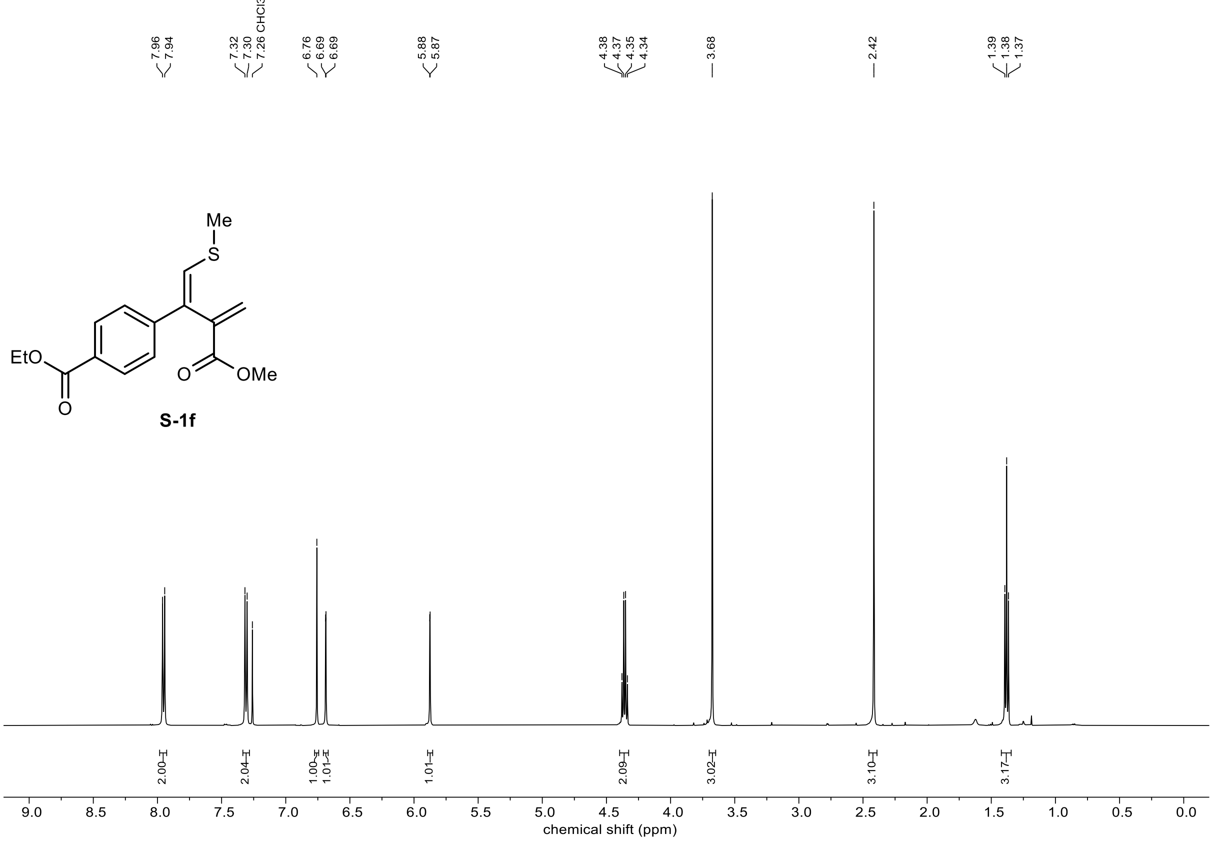

^{13}C NMR (126 MHz, $CDCl_3$)

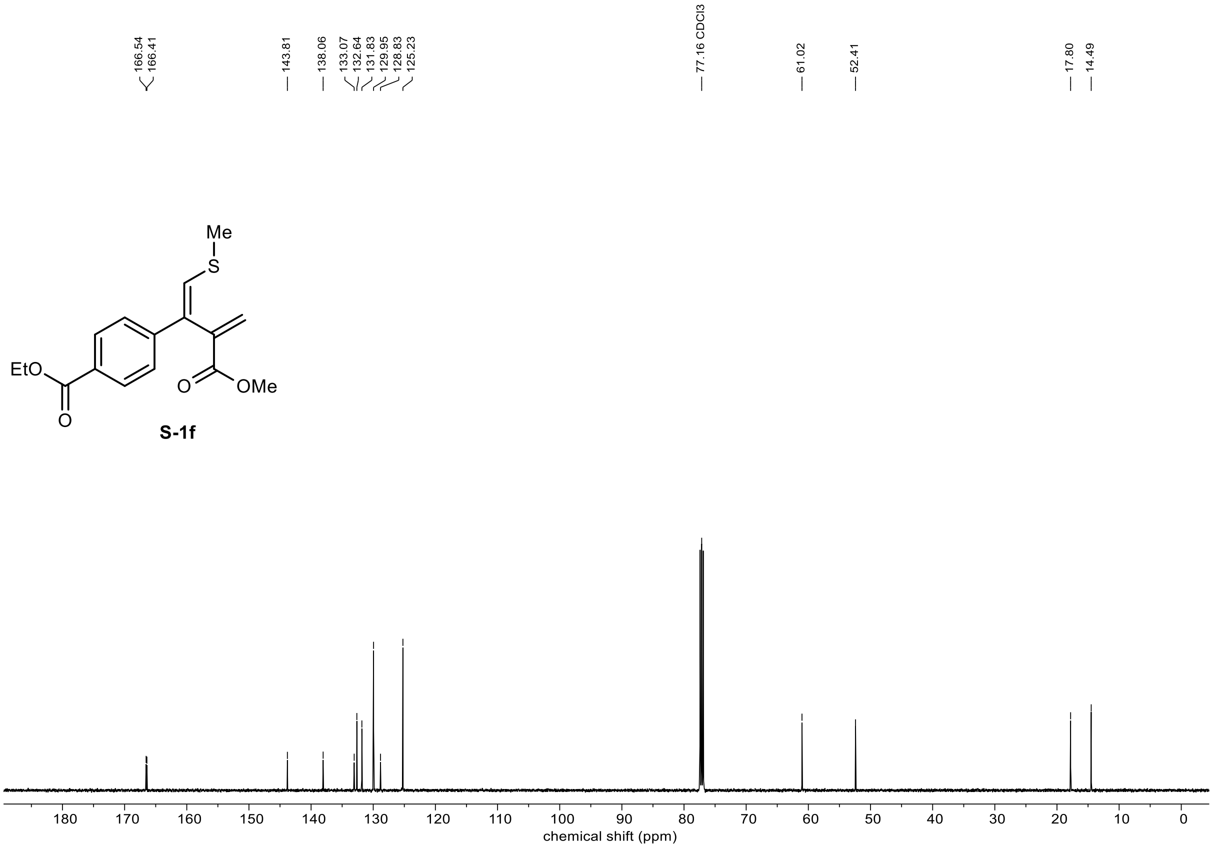

***Tert*-butyl (*Z*)-5-(3-(methoxycarbonyl)-1-(methylthio)buta-1,3-dien-2-yl)-1H-indole-1-carboxylate (S-1k)**

^{1}H NMR (500 MHz, $CDCl_3$)

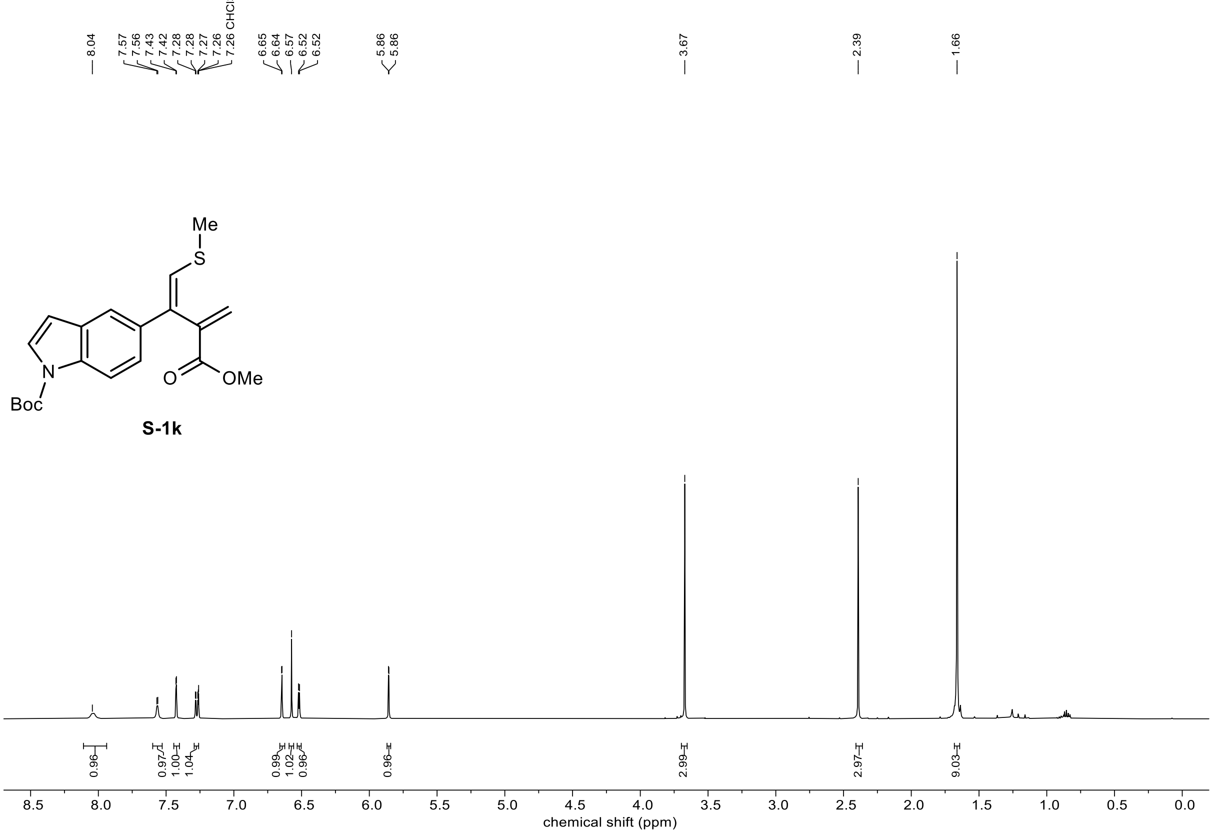

^{13}C NMR (126 MHz, $CDCl_3$)

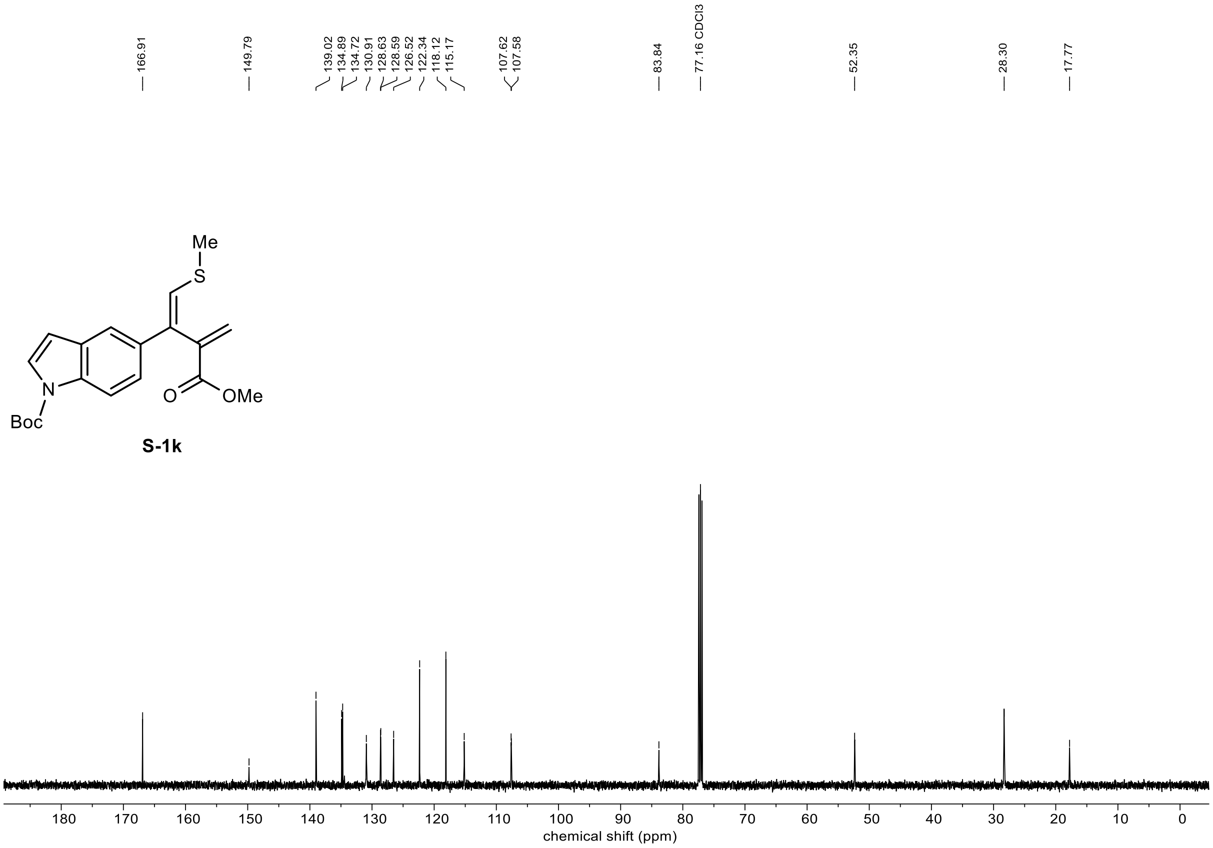

Methyl (*E*)-2-methylene-4-(methylthio)-3-((triisopropylsilyl)oxy)but-3-enoate (S-1m)

^{1}H NMR (500 MHz, $CDCl_3$)

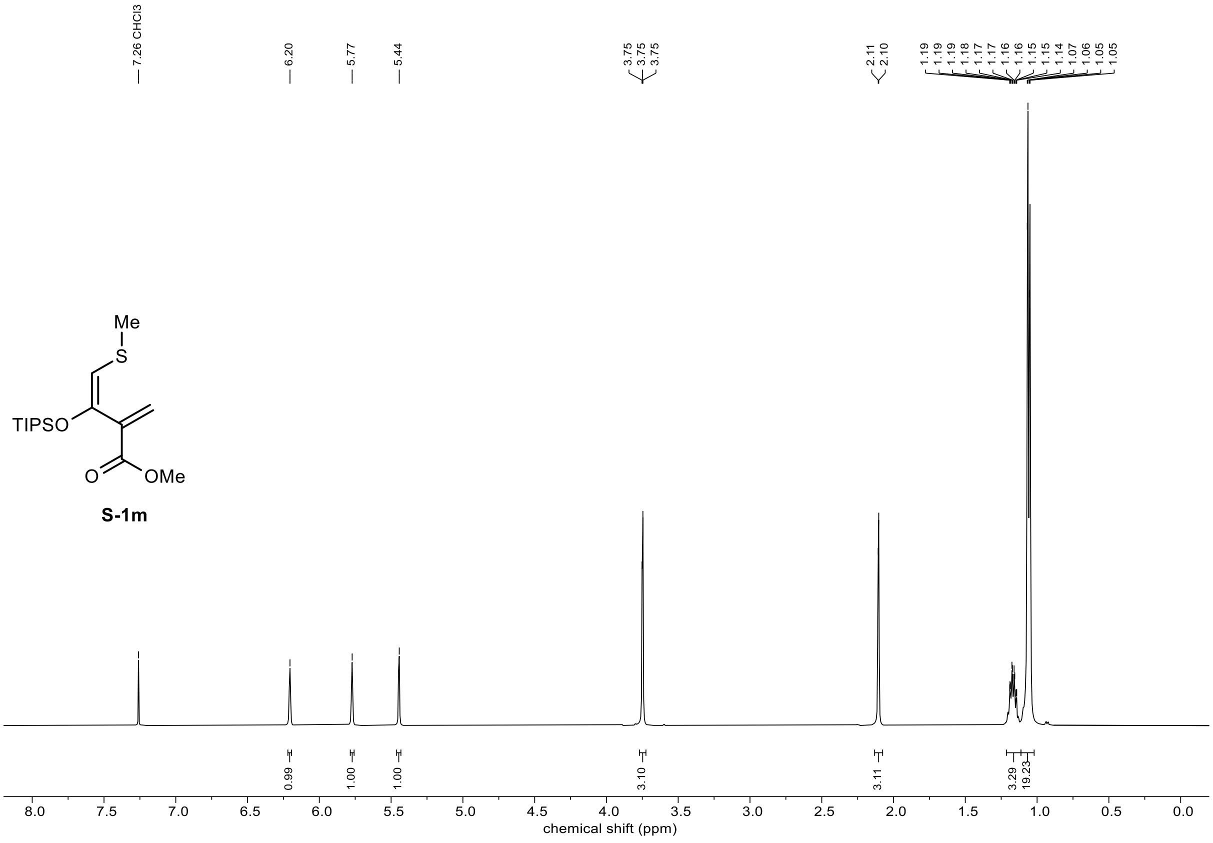

^{13}C NMR (126 MHz, $CDCl_3$)

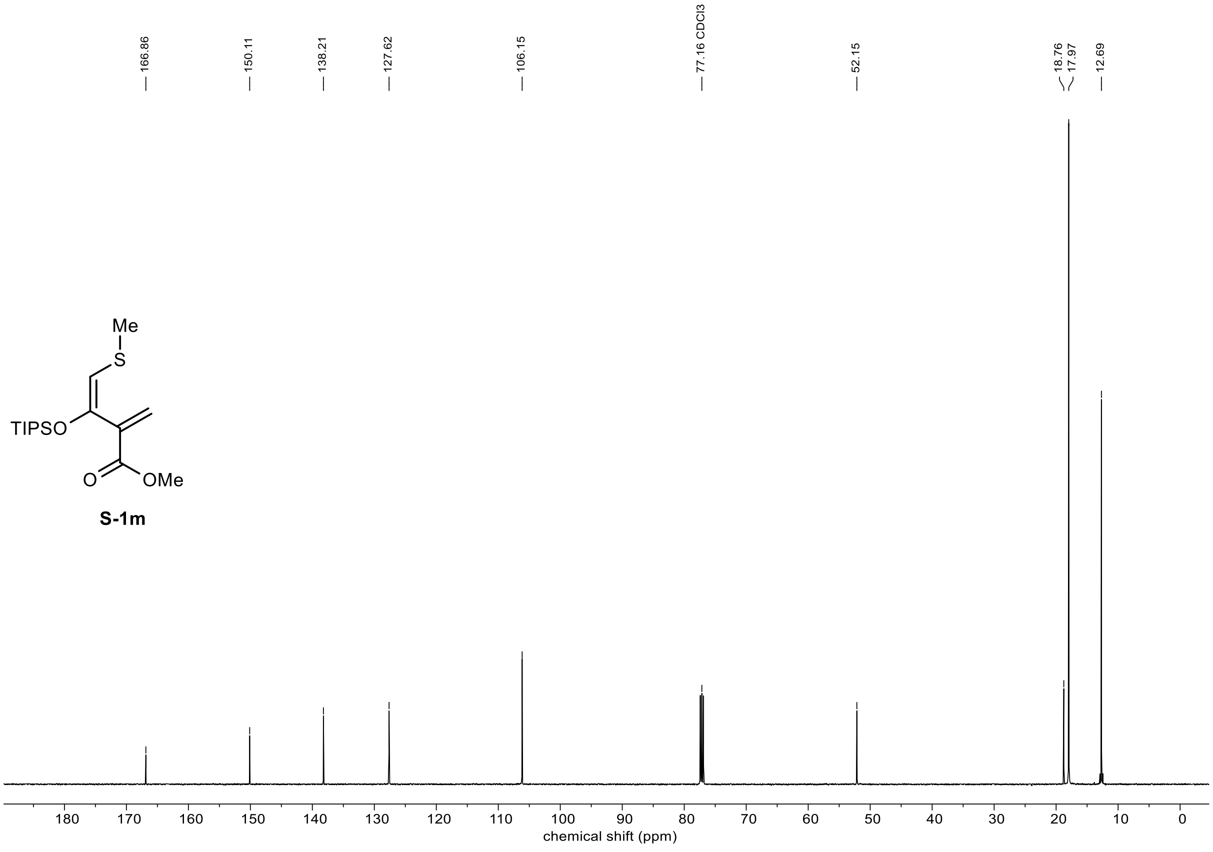

Methyl (*Z*)-2-methylene-4-(methylthio)-3-phenylpent-3-enoate (S-1o)

^{1}H NMR (500 MHz, $CDCl_3$)

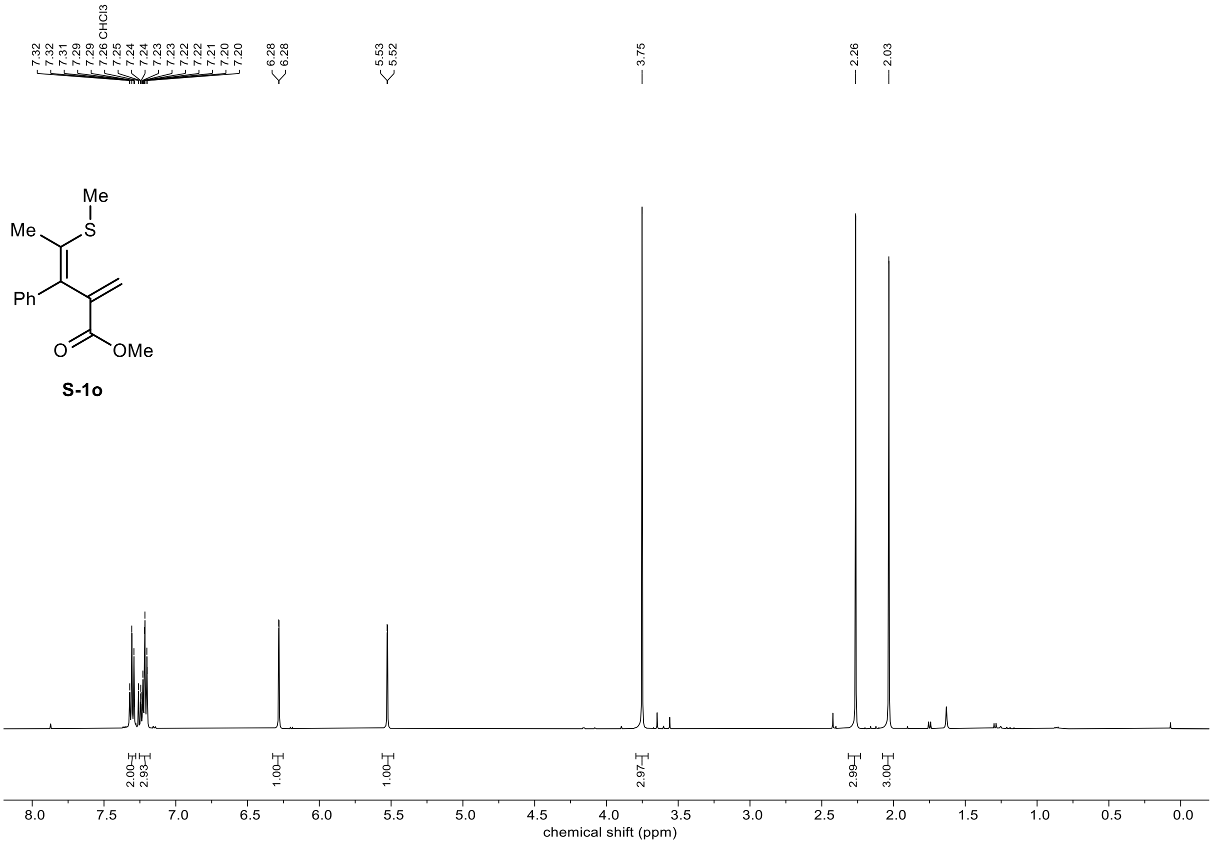

^{13}C NMR (126 MHz, $CDCl_3$)

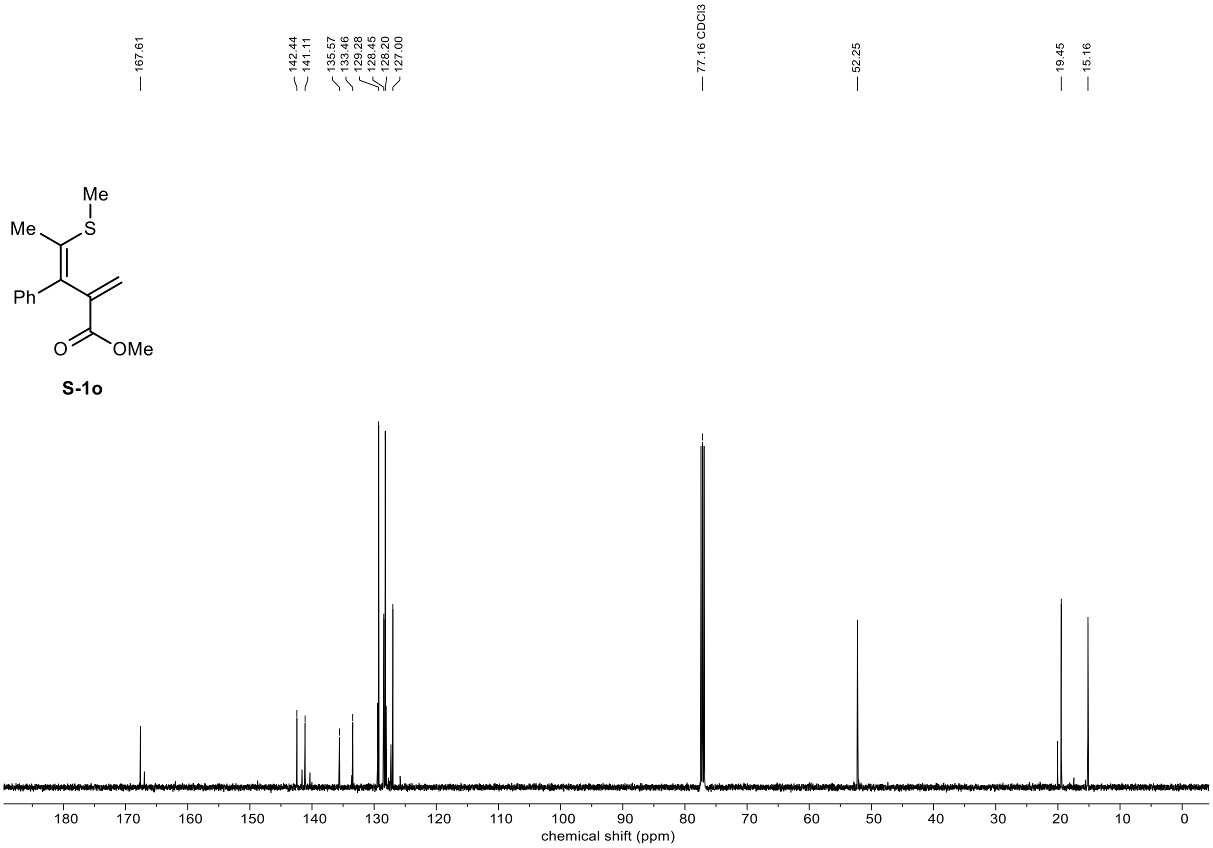

2D (^{1}H–^{1}H) NOESY (500 MHz, $CDCl_3$)

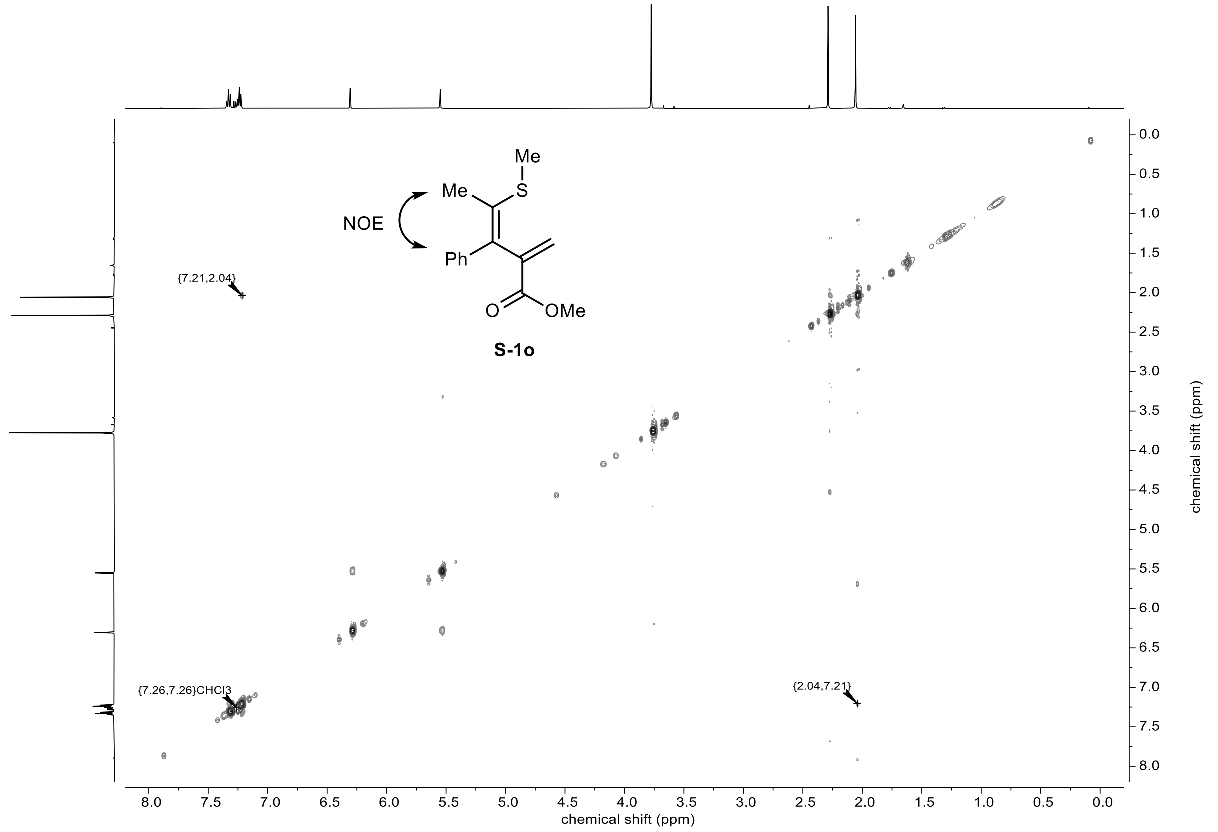

Methyl (*Z*)-4-((2-ethoxy-2-oxoethyl)thio)-2-methylene-3-phenylbut-3-enoate (S-1bb)

^{1}H NMR (500 MHz, $CDCl_3$)

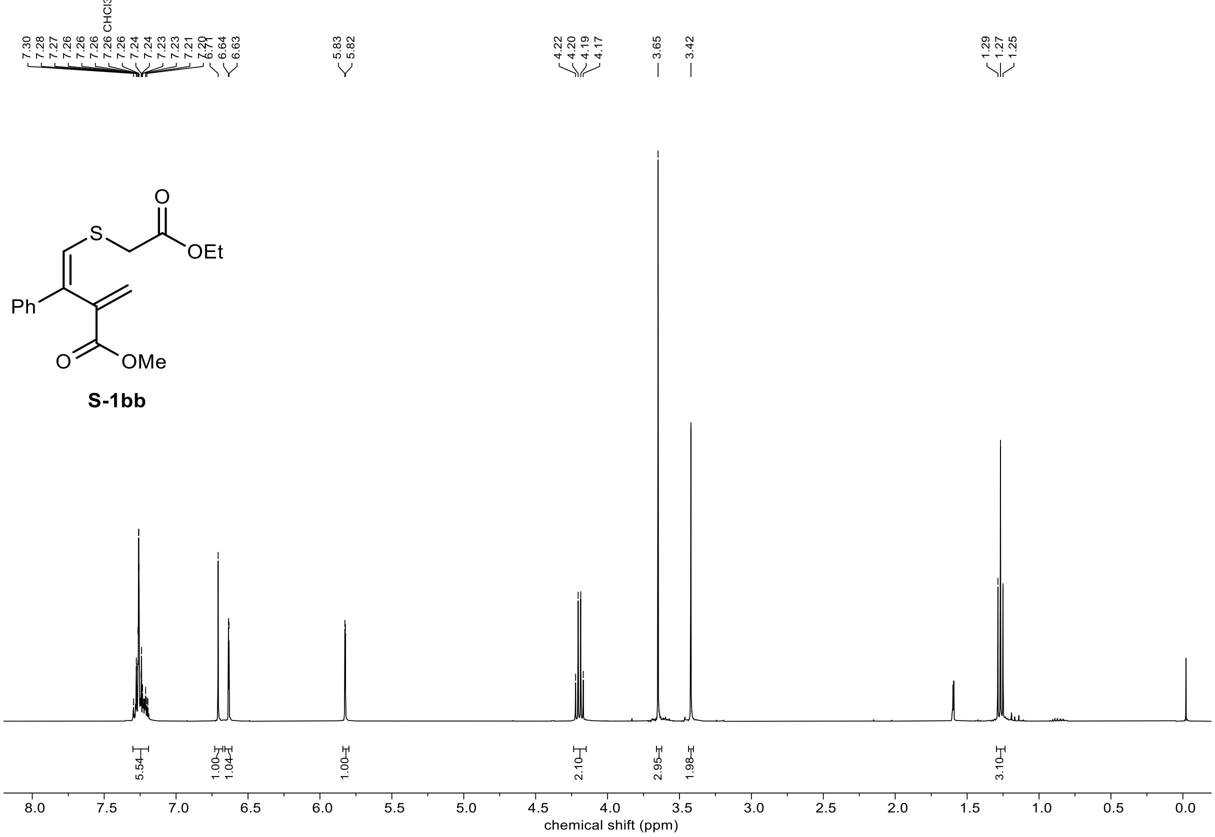

^{13}C NMR (126 MHz, $CDCl_3$)

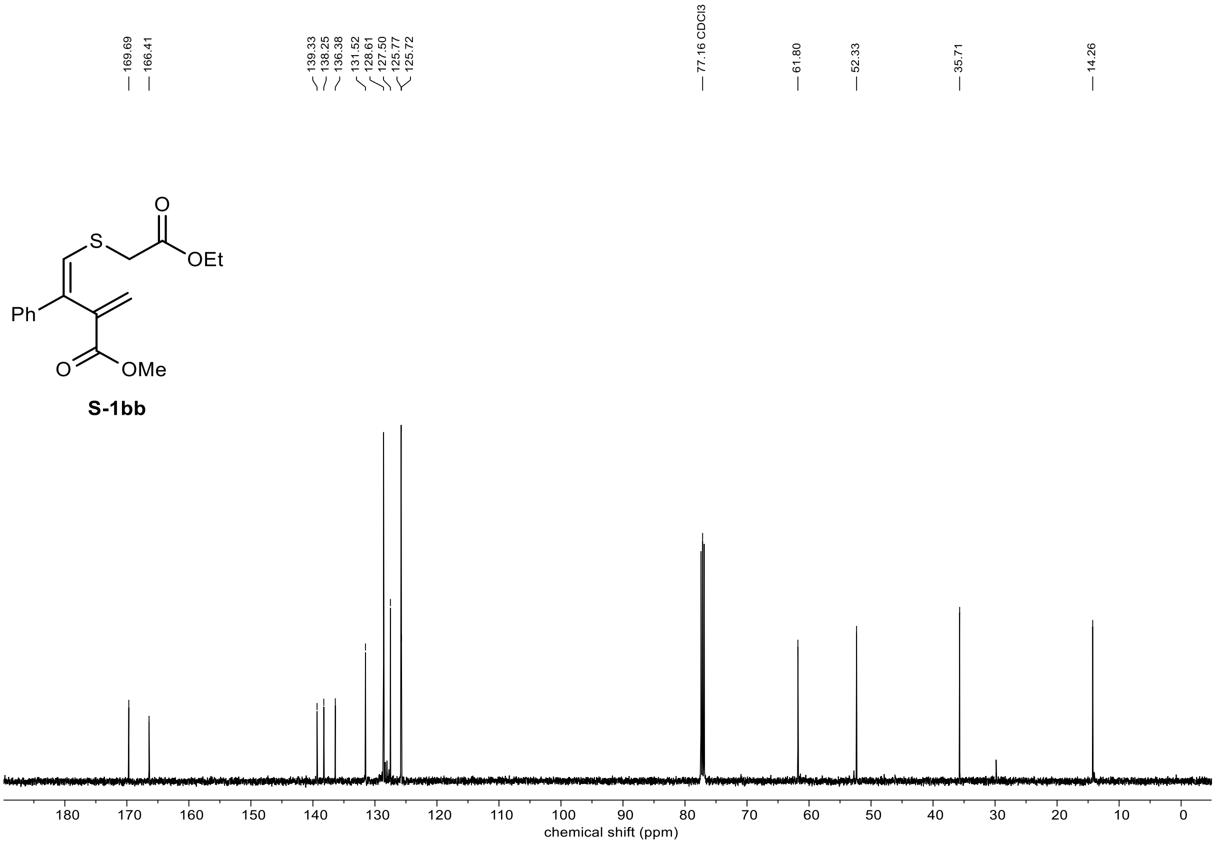

Methyl (*Z*)-4-(benzylthio)-2-methylene-3-phenylbut-3-enoate (S-1bc)

^{1}H NMR (500 MHz, $CDCl_3$)

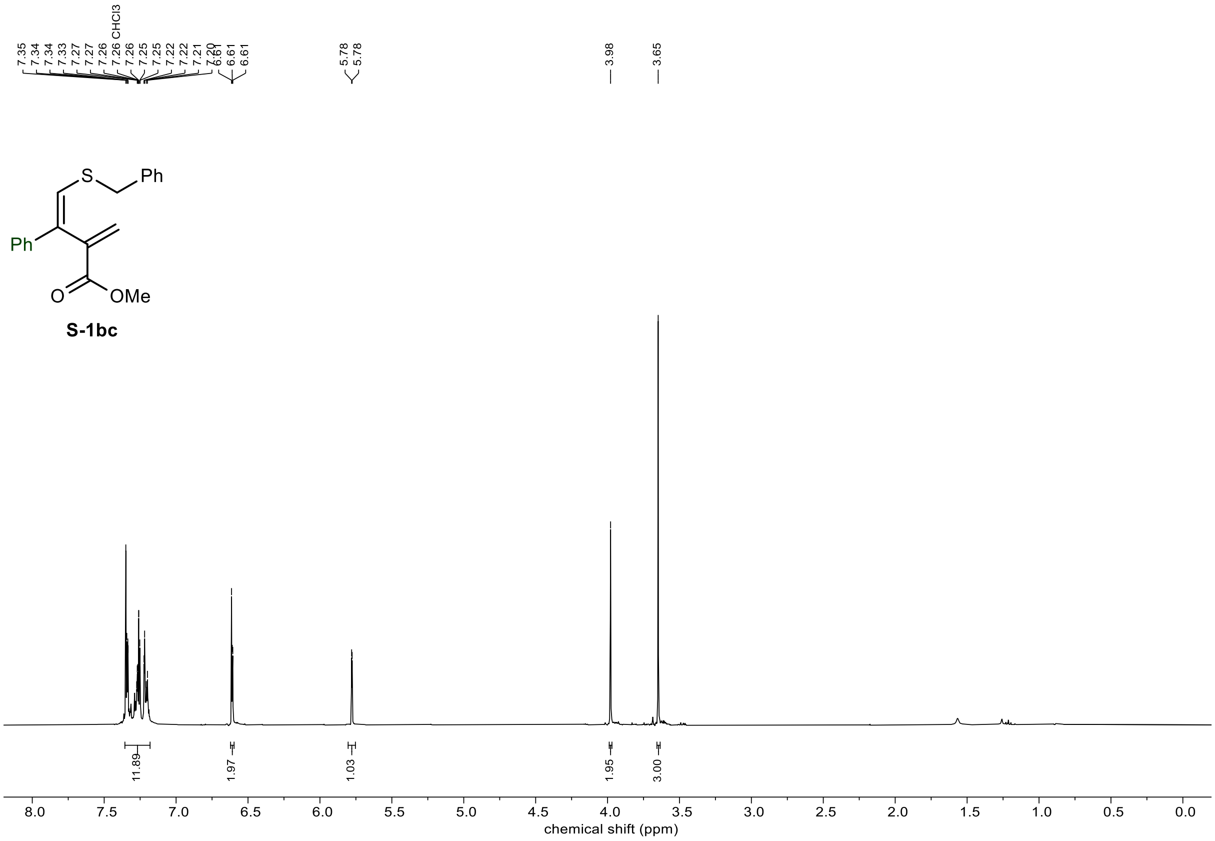

^{13}C NMR (126 MHz, $CDCl_3$)

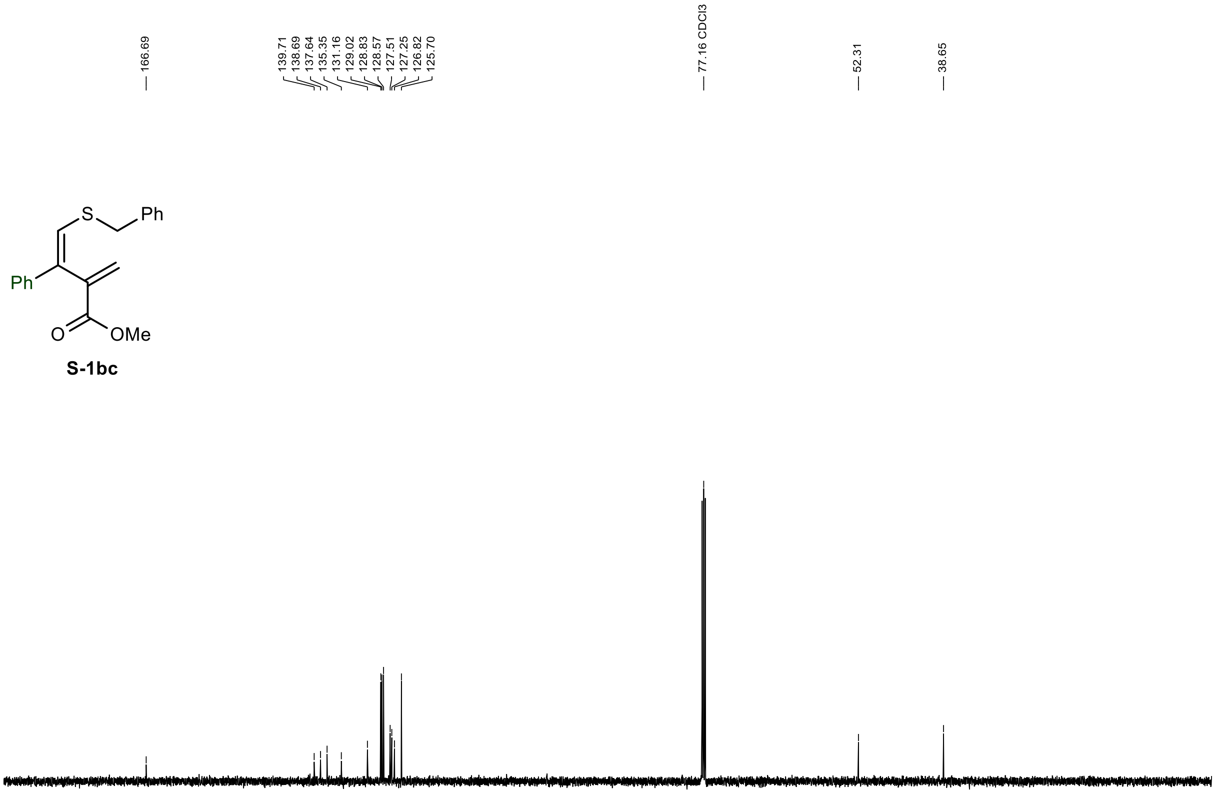

Methyl (*Z*)-4-(allylthio)-2-methylene-3-phenylbut-3-enoate (S-1be)

^{1}H NMR (500 MHz, $CDCl_3$)

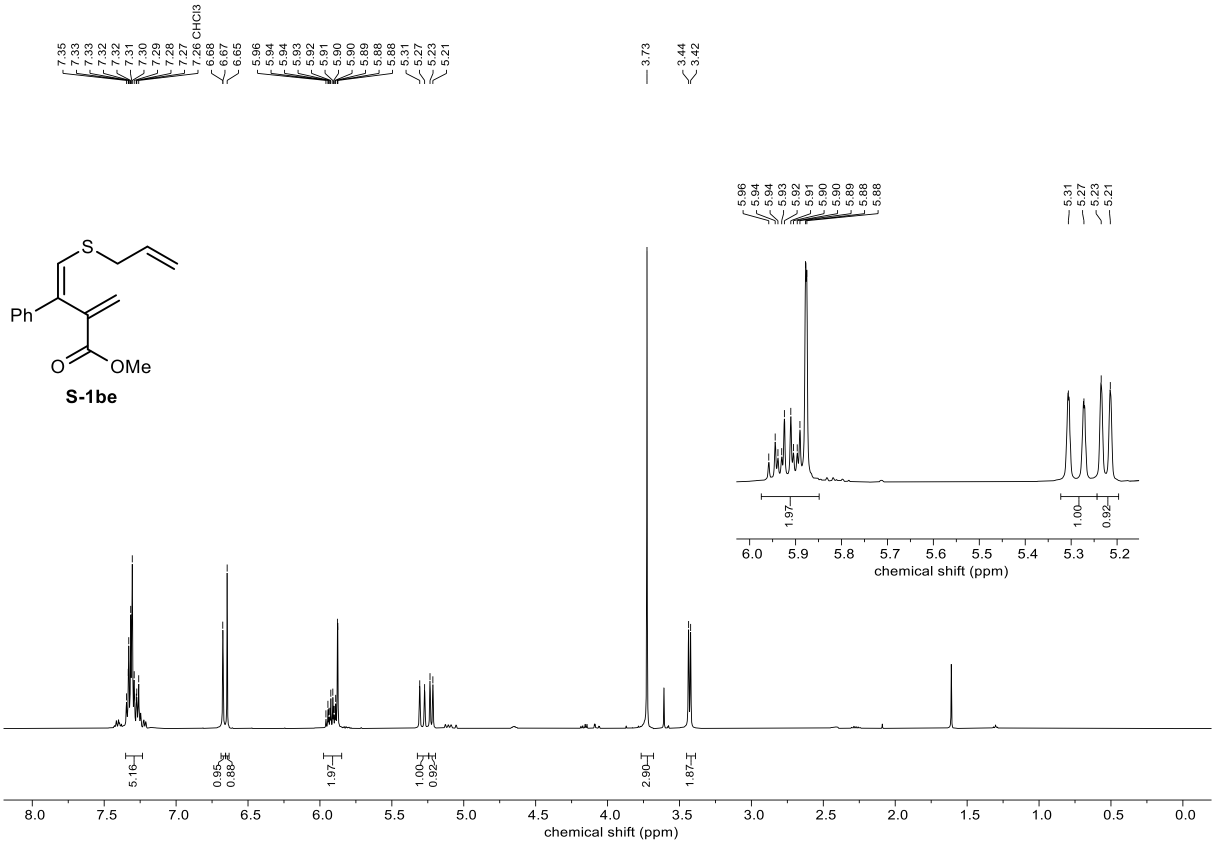

^{13}C NMR (126 MHz, $CDCl_3$)

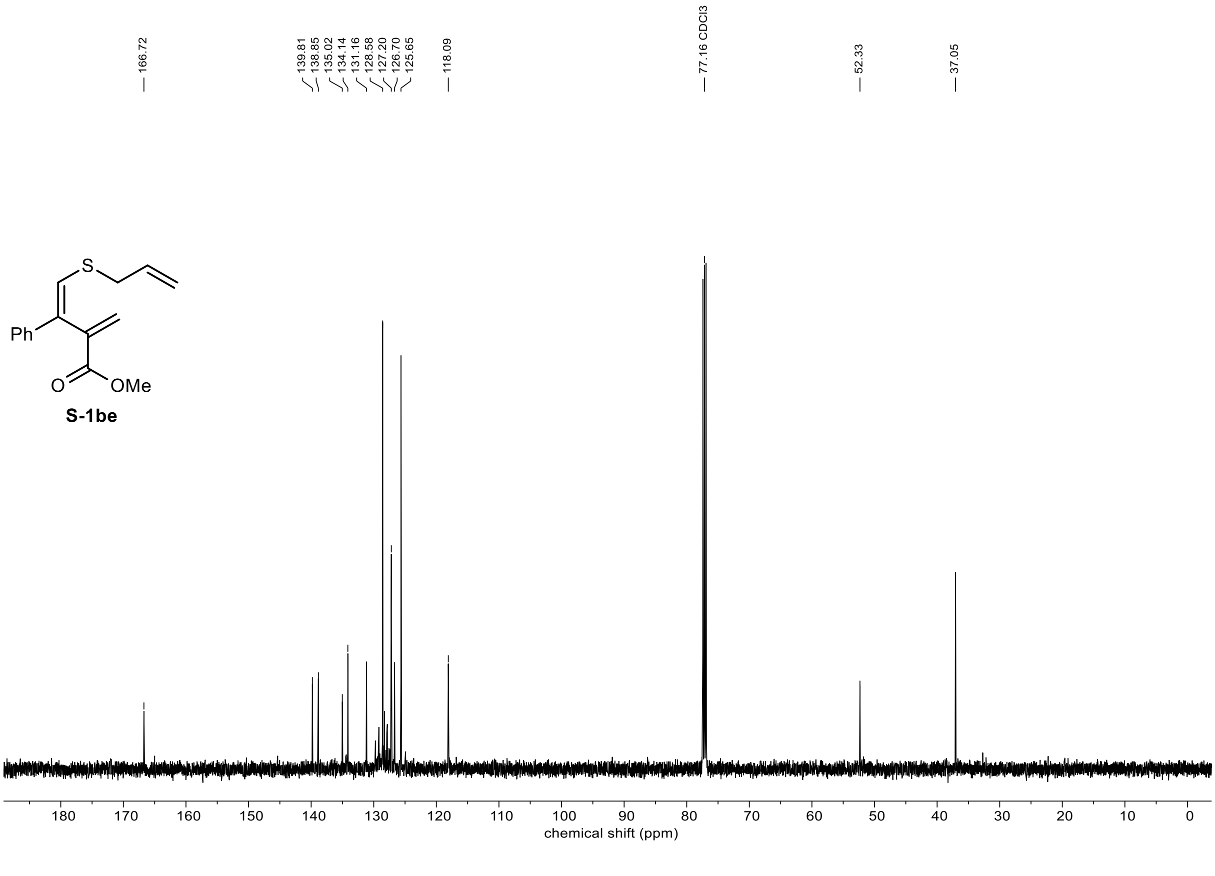

Methyl (*Z*)-4-(((diethoxyphosphoryl)methyl)thio)-2-methylene-3-phenylbut-3-enoate (S-1ca)

^{1}H NMR (500 MHz, $CDCl_3$)

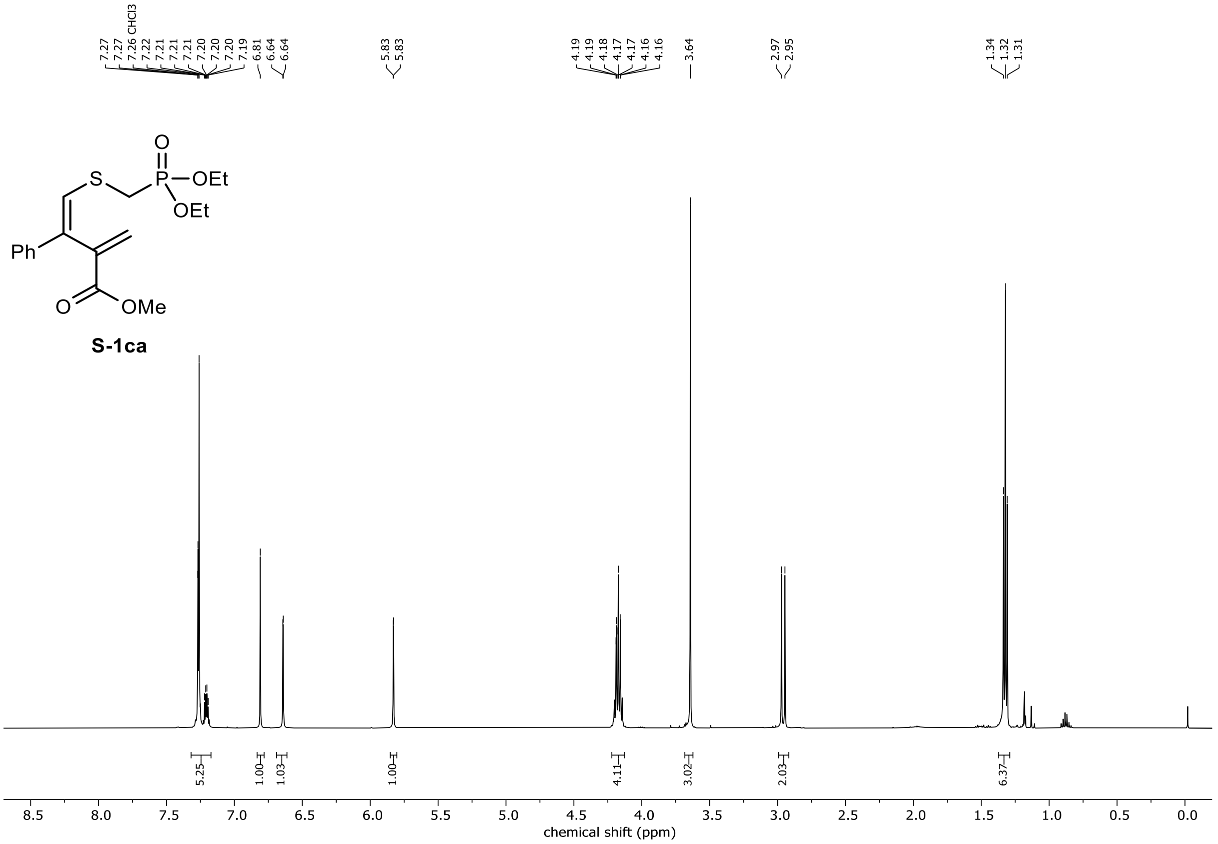

^{13}C NMR (126 MHz, $CDCl_3$)

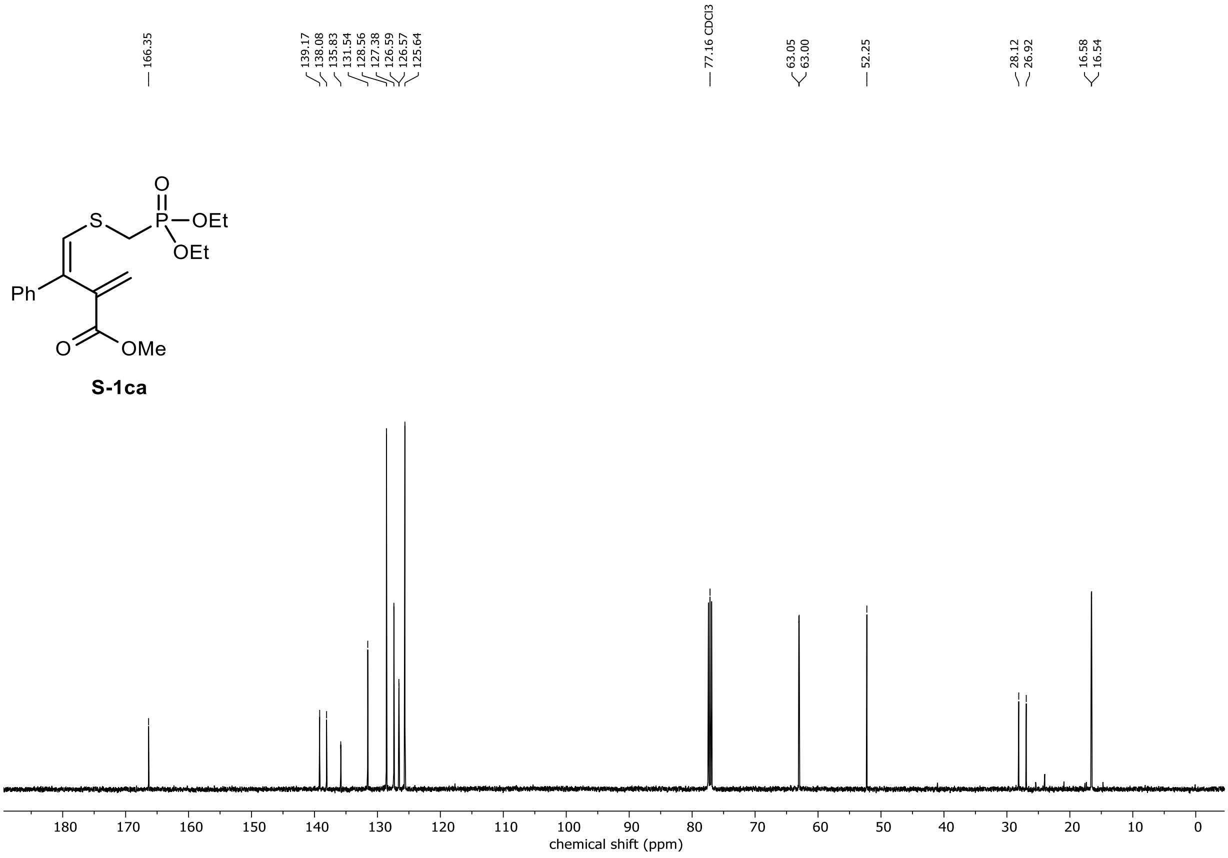

^{31}P{^{1}H,^{13}C}-NMR (162 MHz, $CDCl_3$)

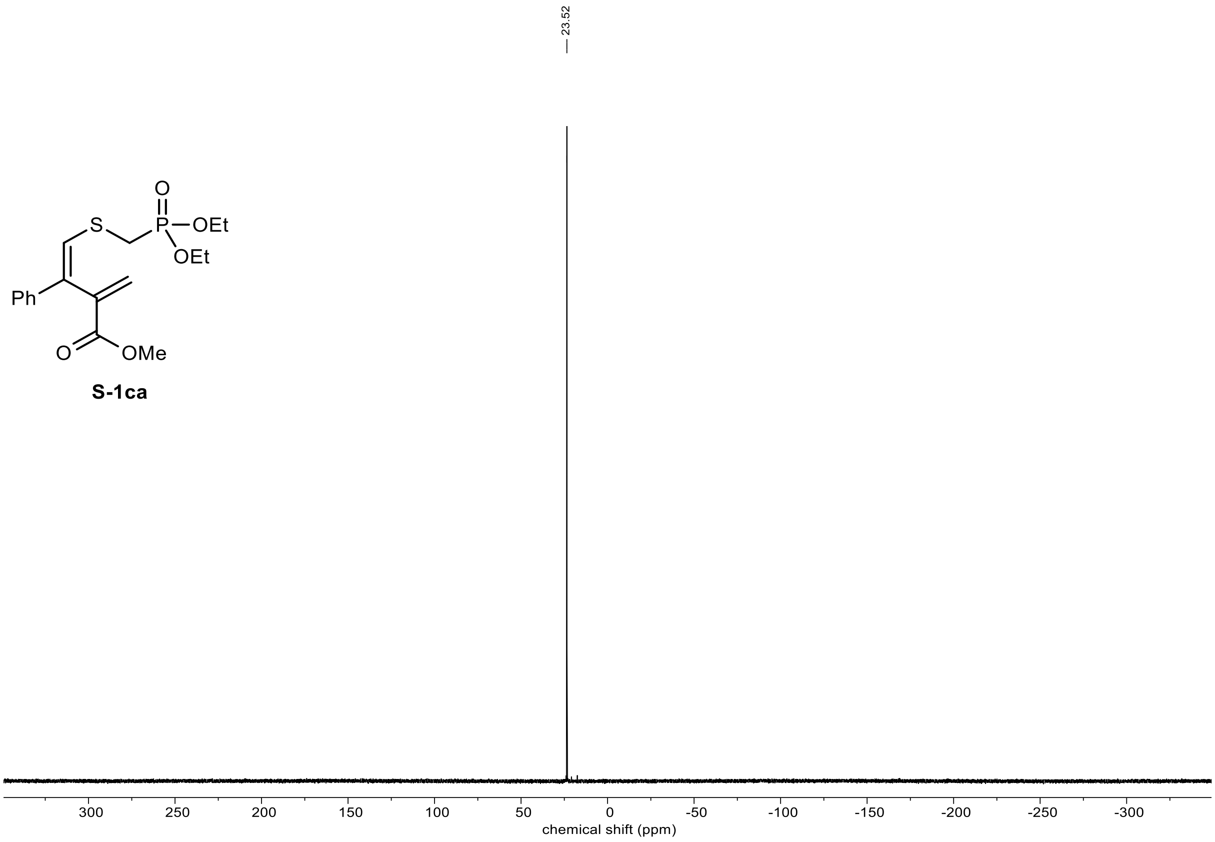

Methyl (*Z*)-2-methylene-3-phenyl-4-((2,2,2-trifluoroethyl)thio)but-3-enoate (S-1cb)

^{1}H NMR (500 MHz, $CDCl_3$)

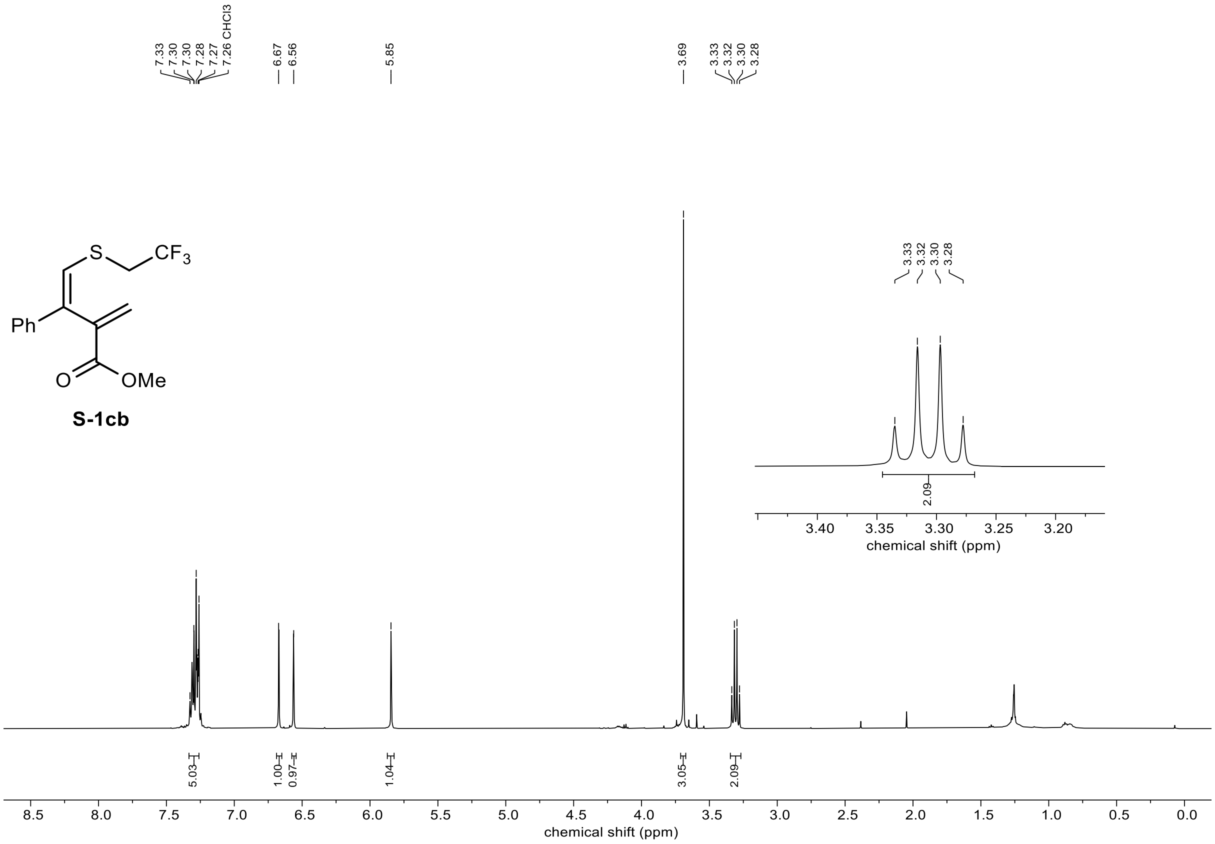

^{13}C NMR (126 MHz, $CDCl_3$)

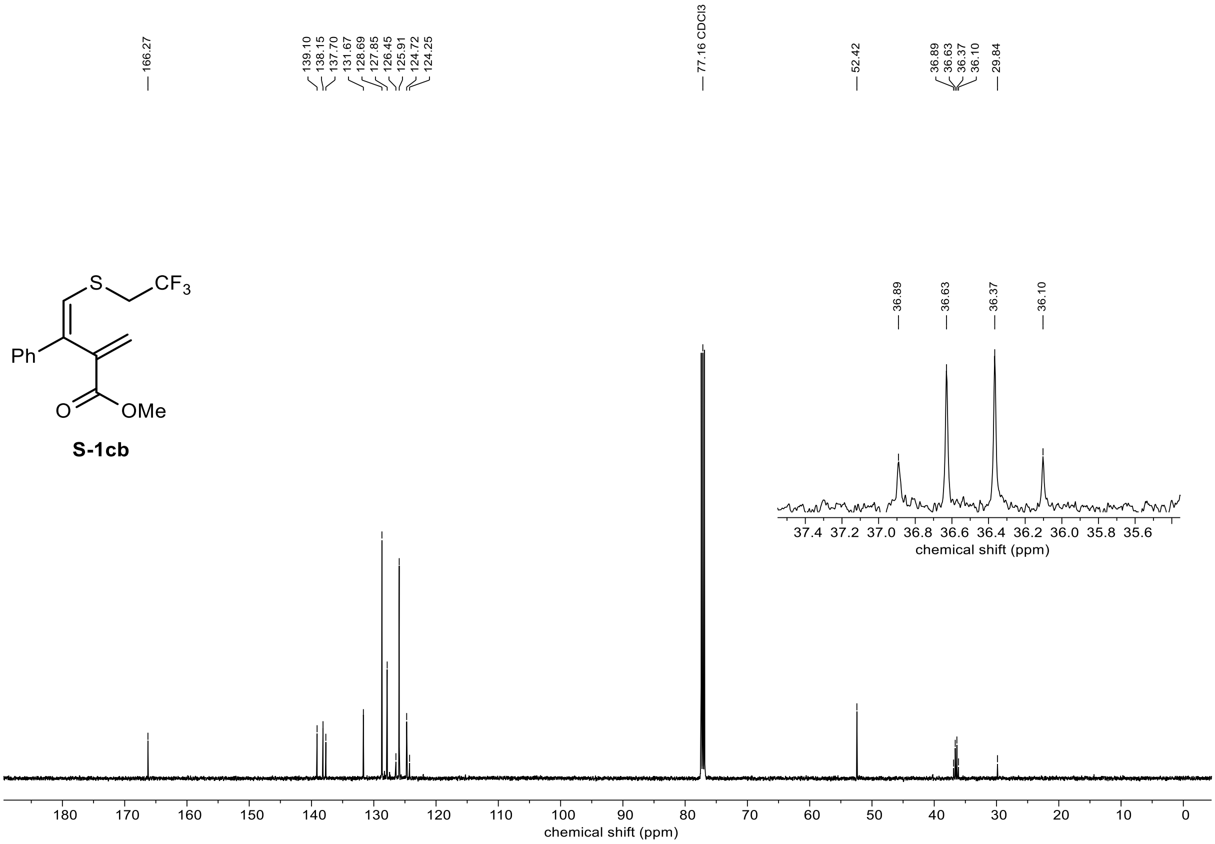

^{19}F NMR (376 MHz, $CDCl_3$)

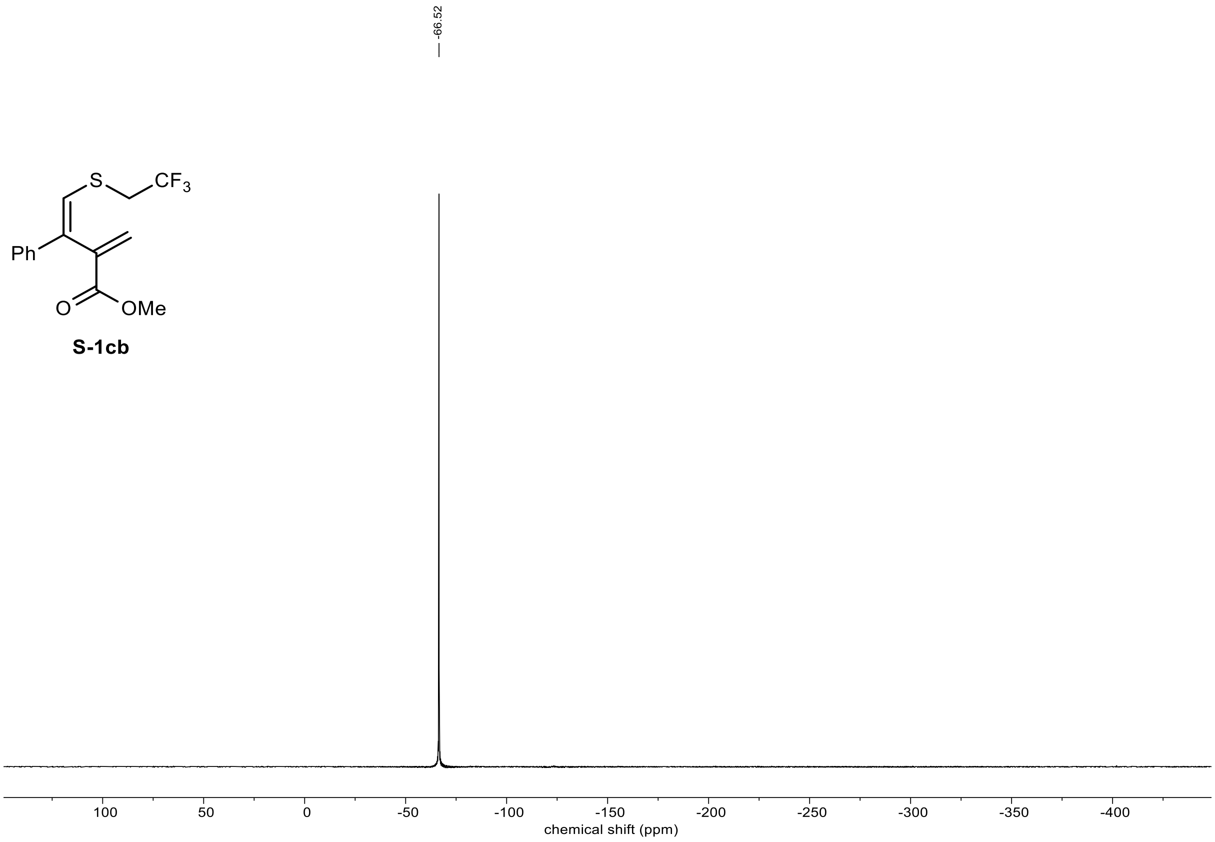

Methyl (*Z*)-4-((cyanomethyl)thio)-2-methylene-3-phenylbut-3-enoate (S-1cc)

^{1}H NMR (500 MHz, $CDCl_3$)

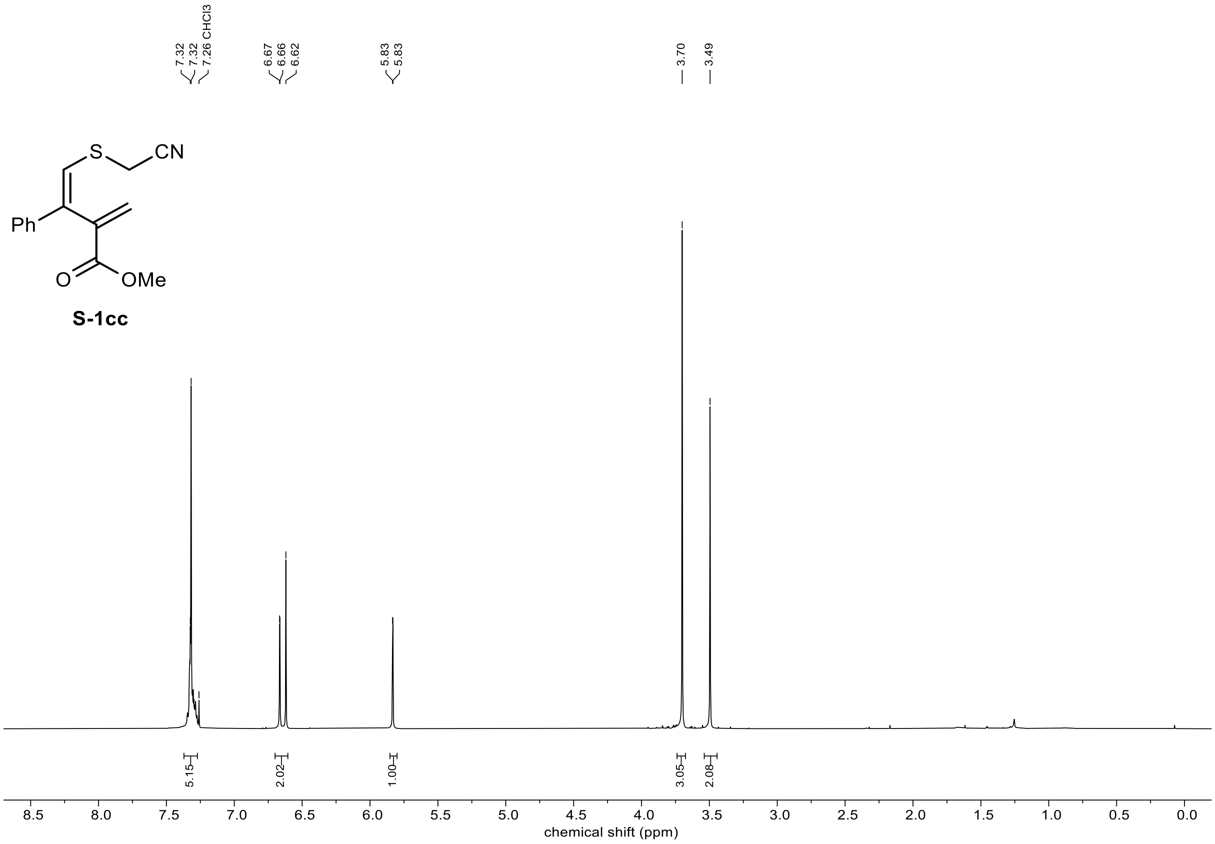

^{13}C NMR (126 MHz, $CDCl_3$)

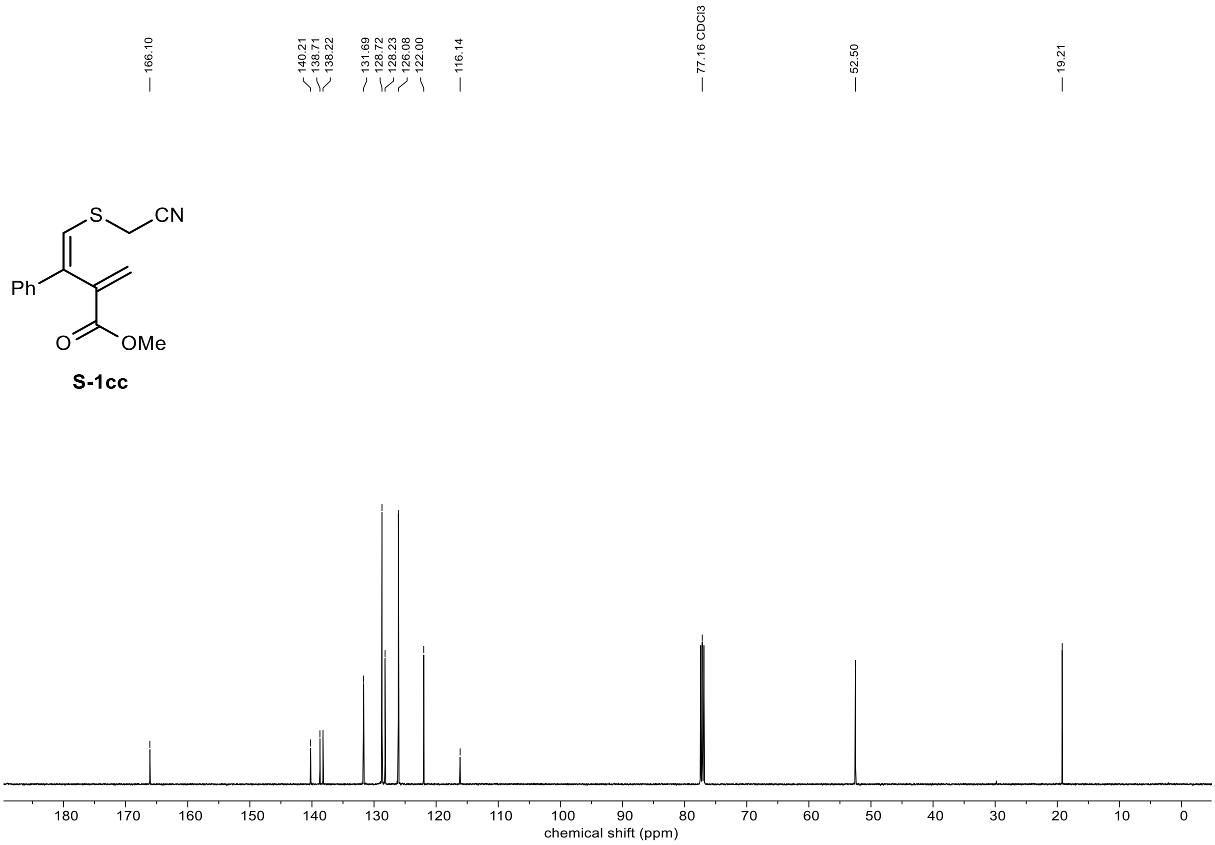

1-Ethyl 2-methyl 1-(methylthio)-2-(1-phenylvinyl)cyclopropane-1,2-dicarboxylate (3a)

^{1}H NMR (500 MHz, CDCl$_3$)

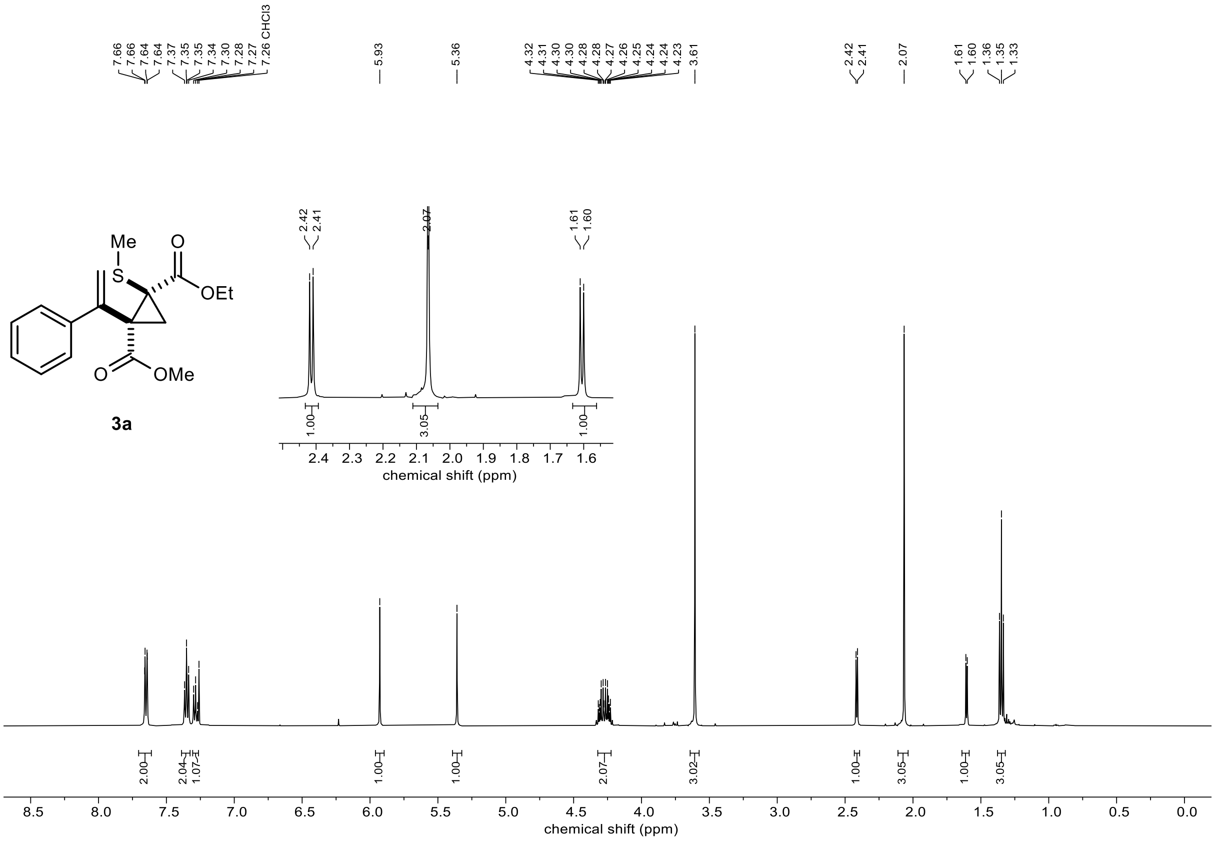

3a

^{13}C NMR (126 MHz, CDCl$_3$)

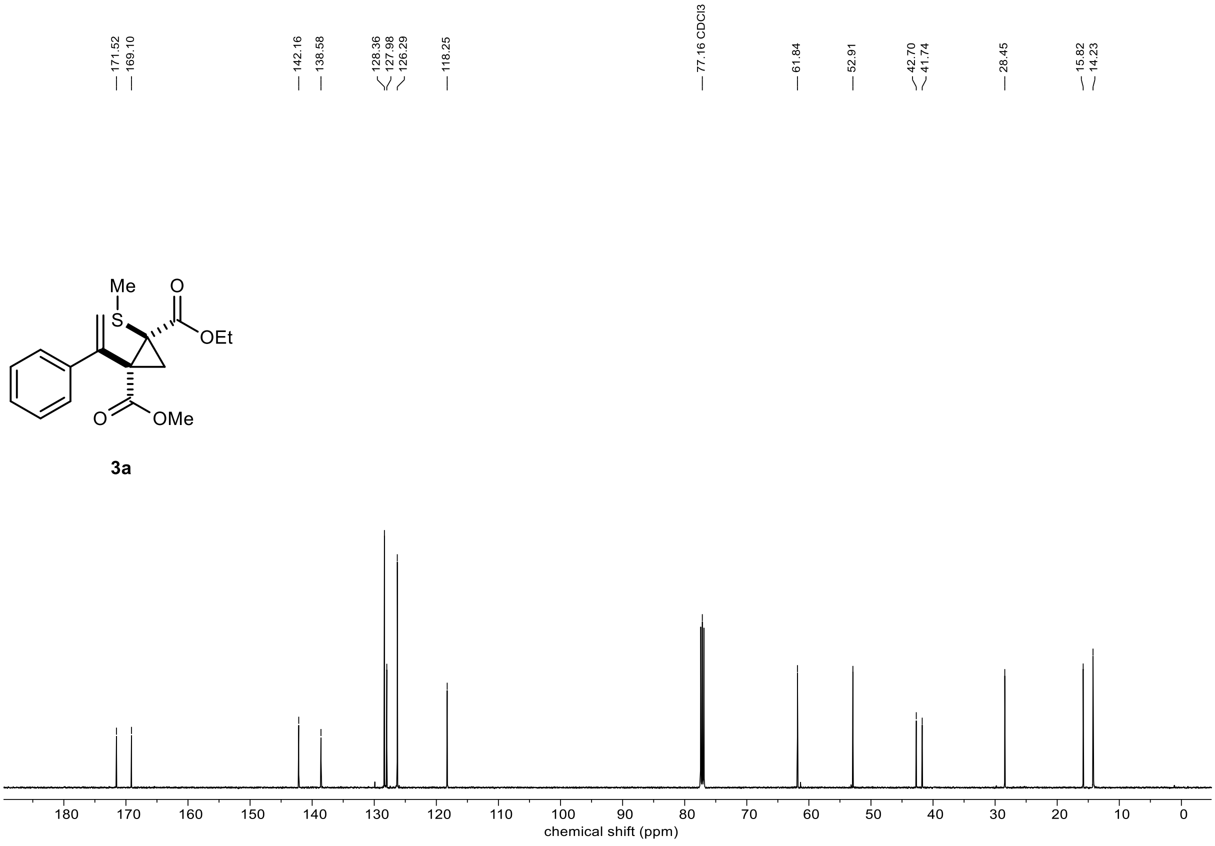

3a

1-Ethyl 2-methyl 2-(1-(4-methoxyphenyl)vinyl)-1-(methylthio)cyclopropane-1,2-dicarboxylate (3b)

^{1}H NMR (400 MHz, CDCl$_3$)

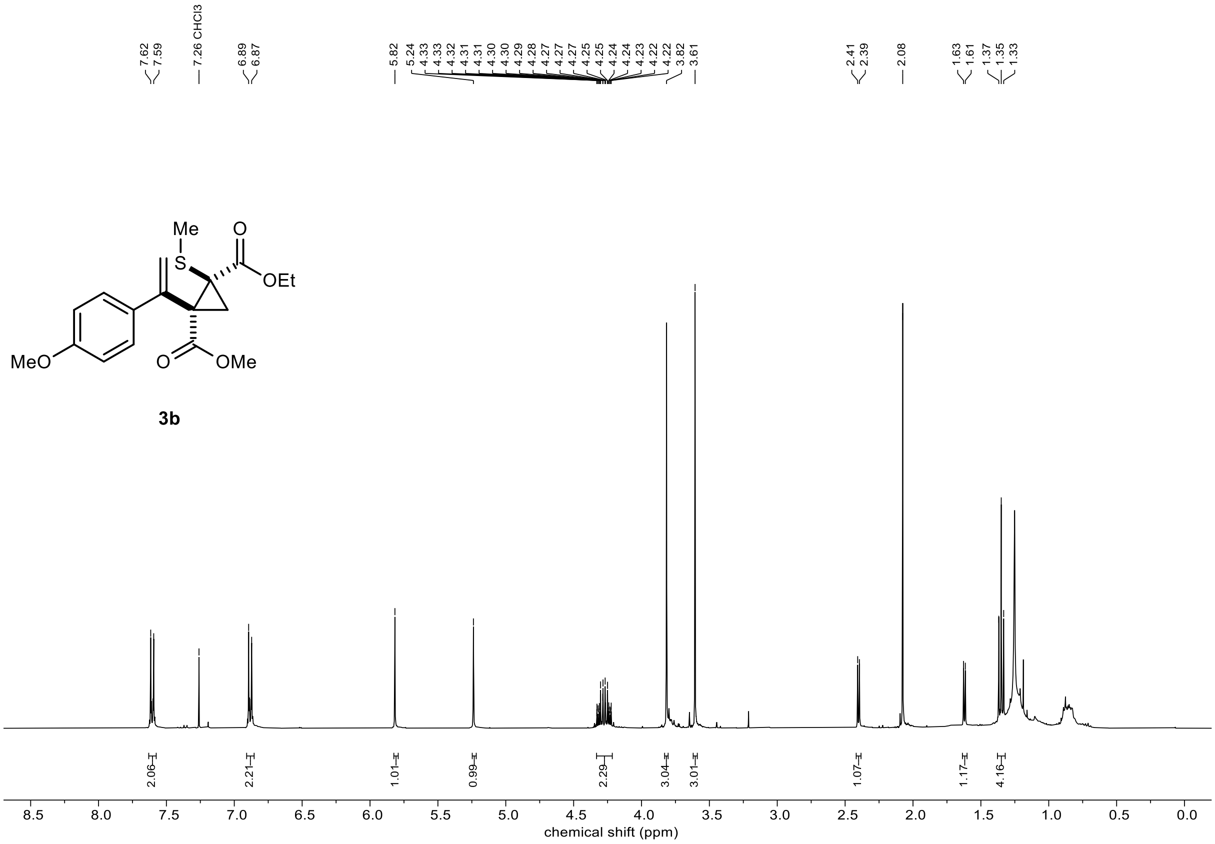

^{13}C NMR (126 MHz, CDCl$_3$)

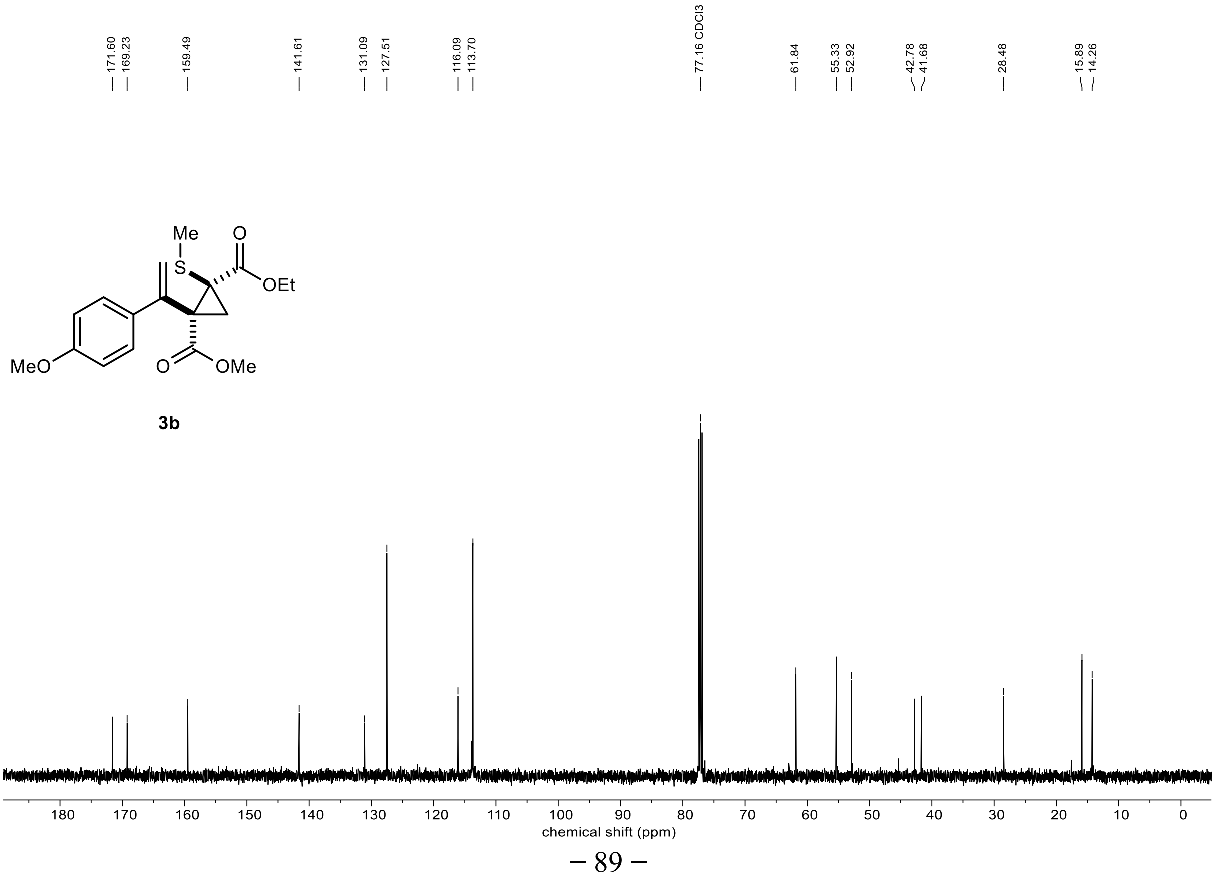

1-Ethyl 2-methyl 1-(methylthio)-2-(1-(4-(trifluoromethyl)phenyl)vinyl)cyclopropane-1,2-dicarboxylate (3c)

^{1}H NMR (500 MHz, $CDCl_3$)

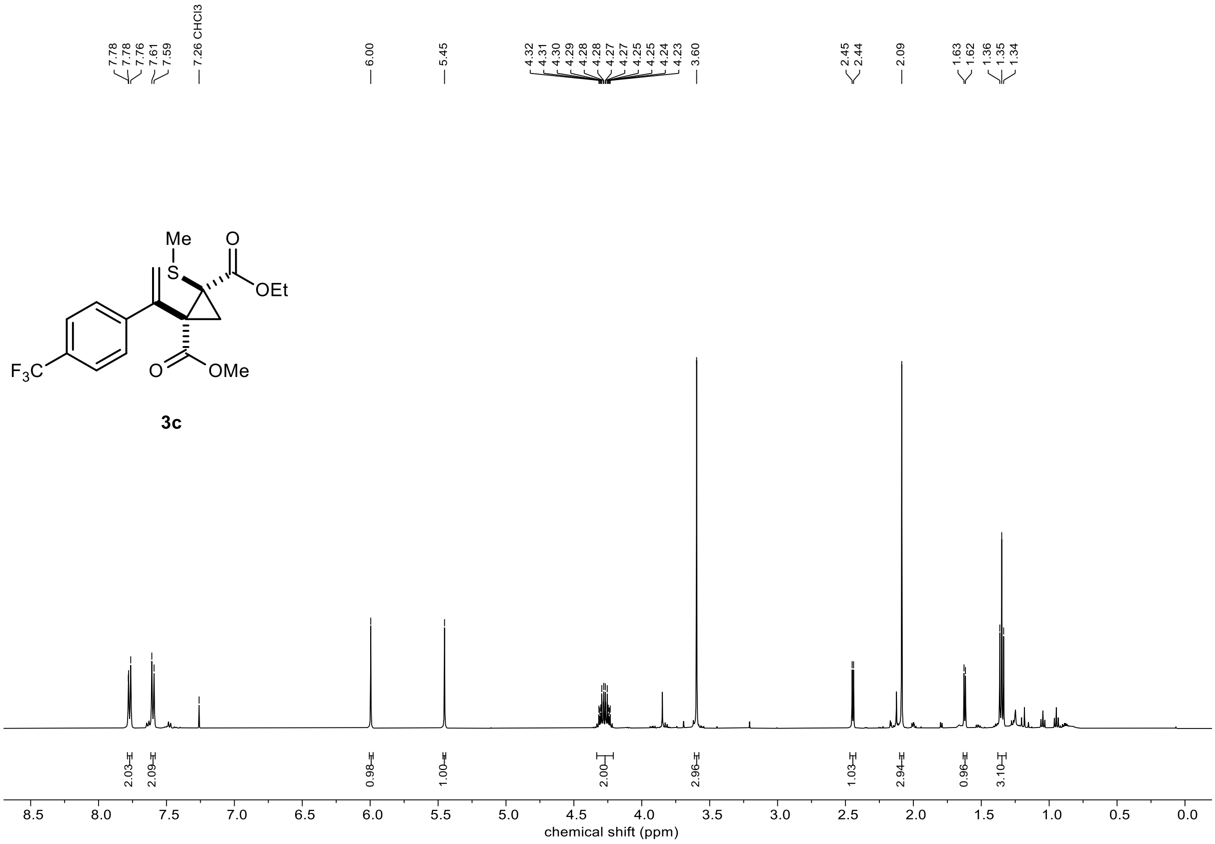

3c

^{13}C NMR (126 MHz, $CDCl_3$)

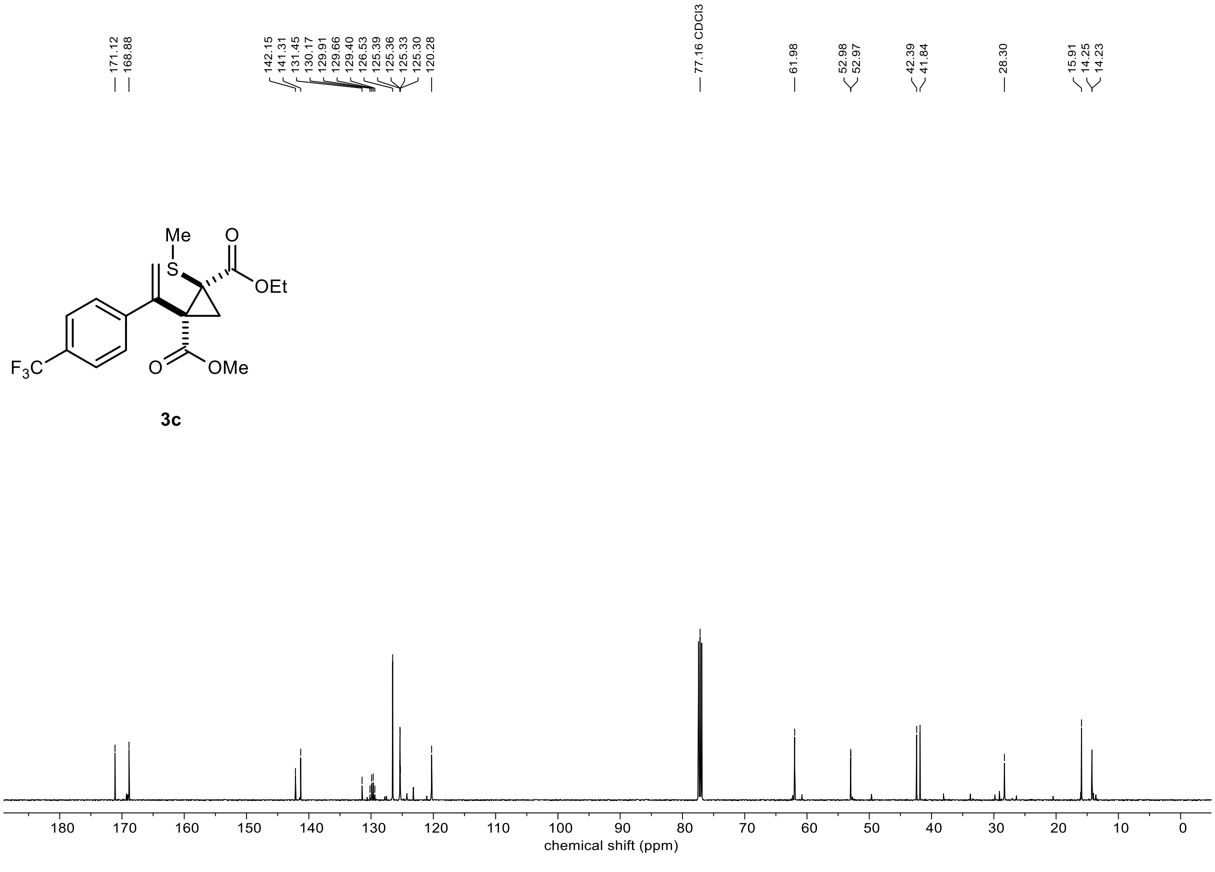

3c

^{19}F NMR (376 MHz, $CDCl_3$)

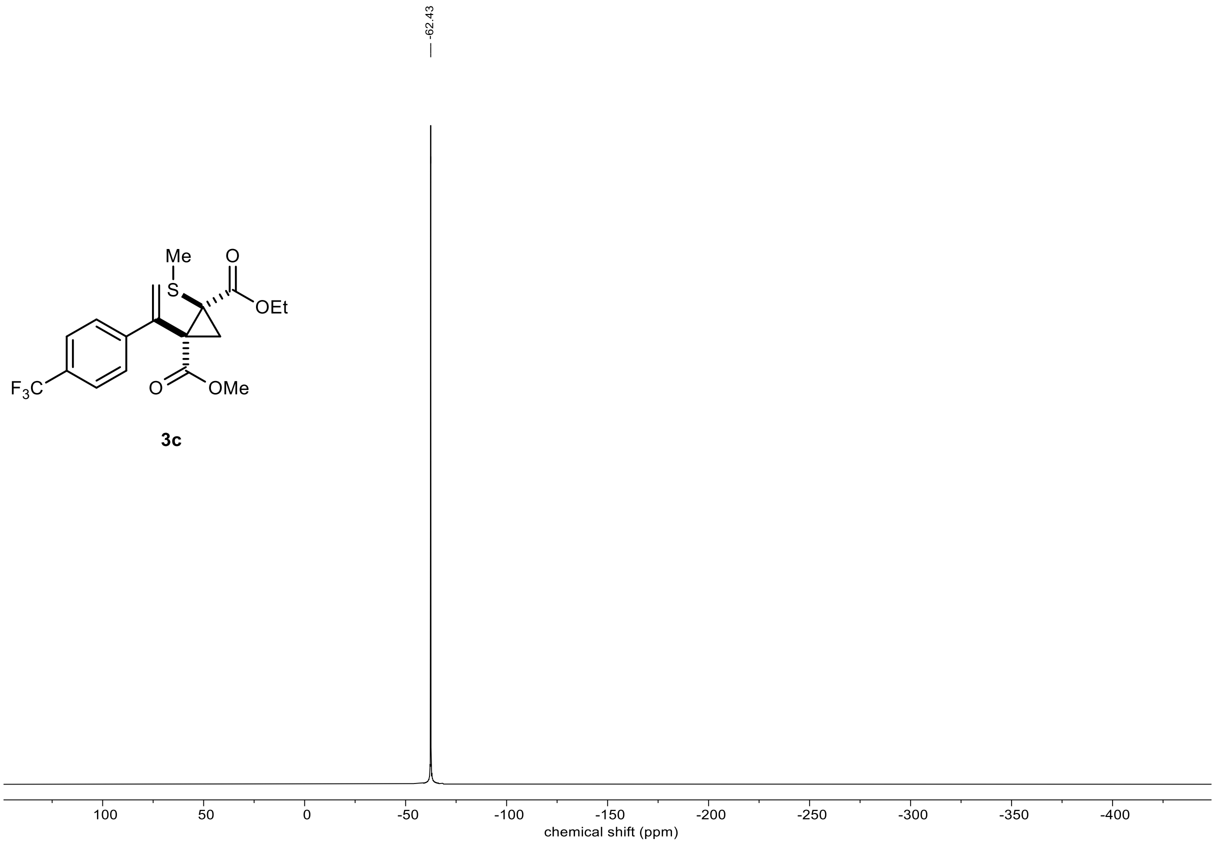

2-Ethyl 1-methyl 1-(1-(4-bromophenyl)vinyl)-2-(methylthio)cyclopropane-1,2-dicarboxylate (3d)

^{1}H NMR (500 MHz, $CDCl_3$)

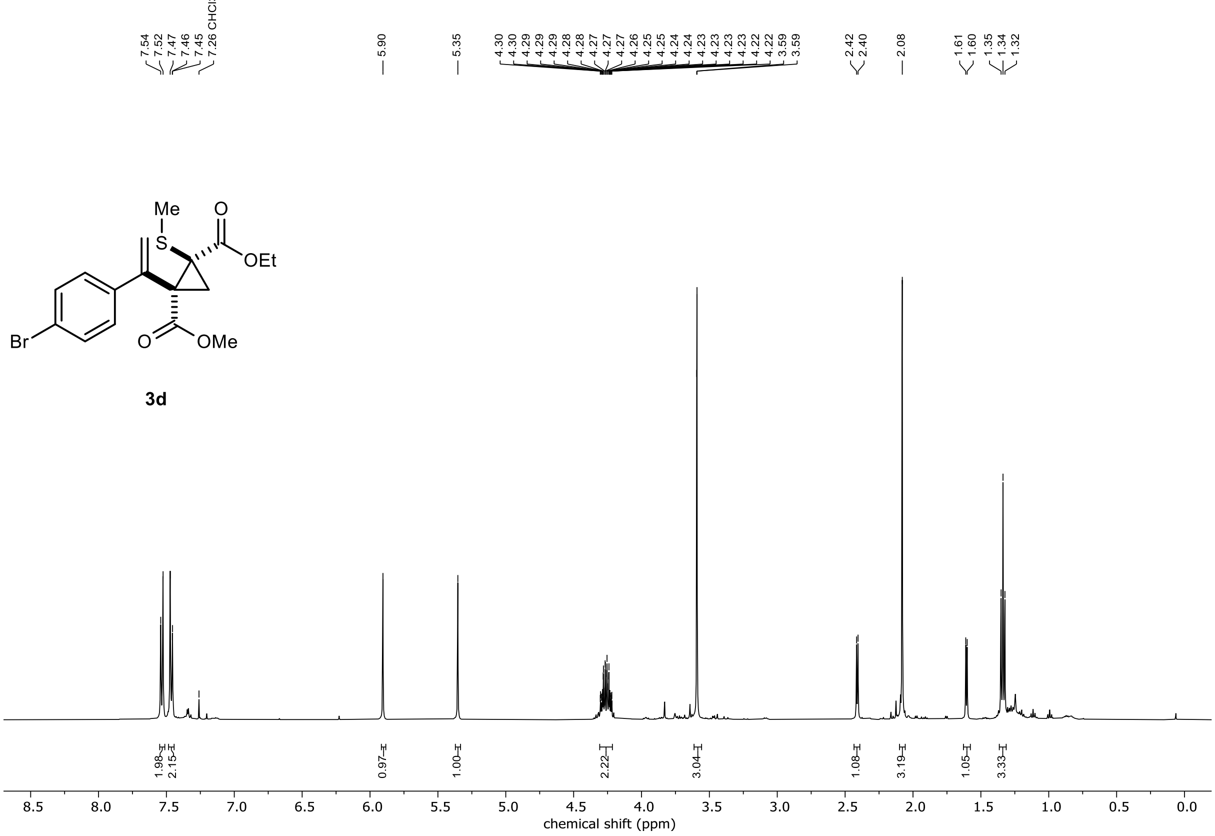

^{13}C NMR (126 MHz, $CDCl_3$)

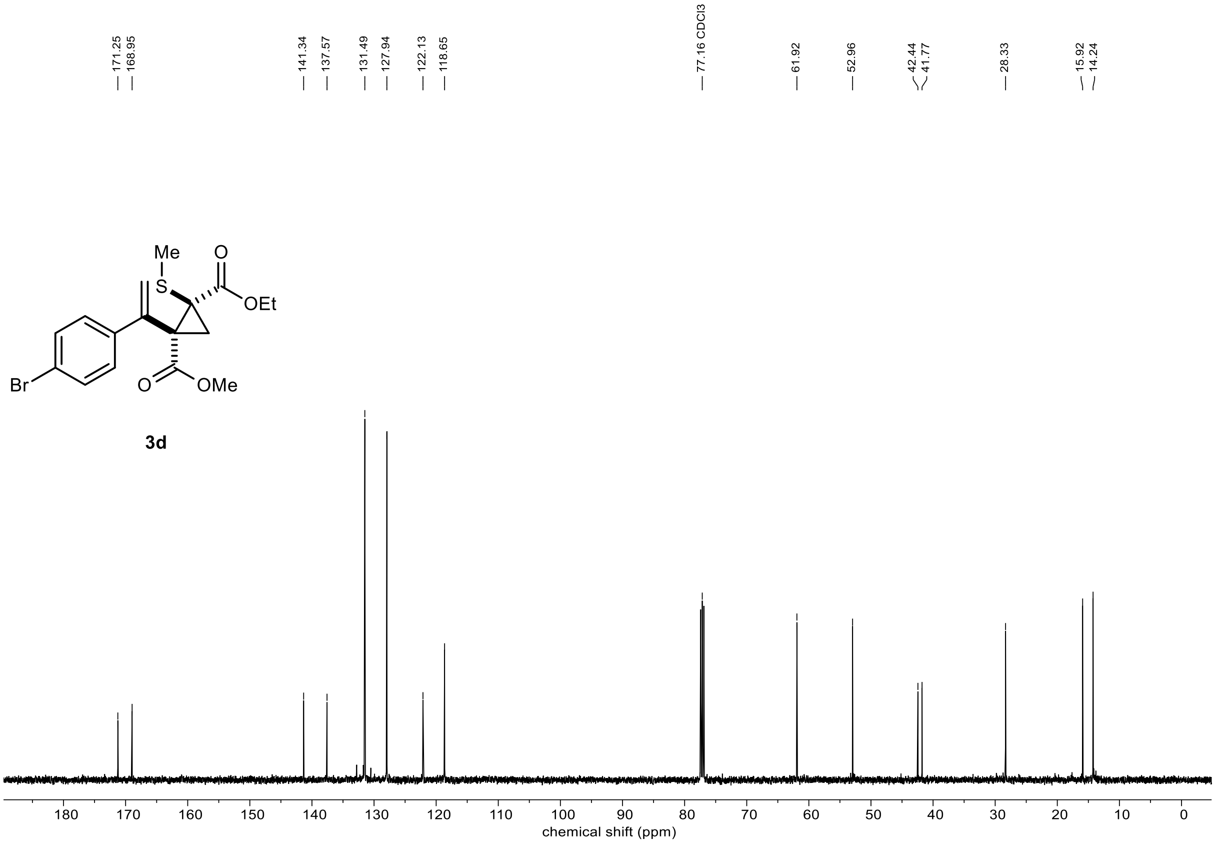

1-Ethyl 2-methyl 2-(1-(4-formylphenyl)vinyl)-1-(methylthio)cyclopropane-1,2-dicarboxylate (3e)

^{1}H NMR (500 MHz, $CDCl_3$)

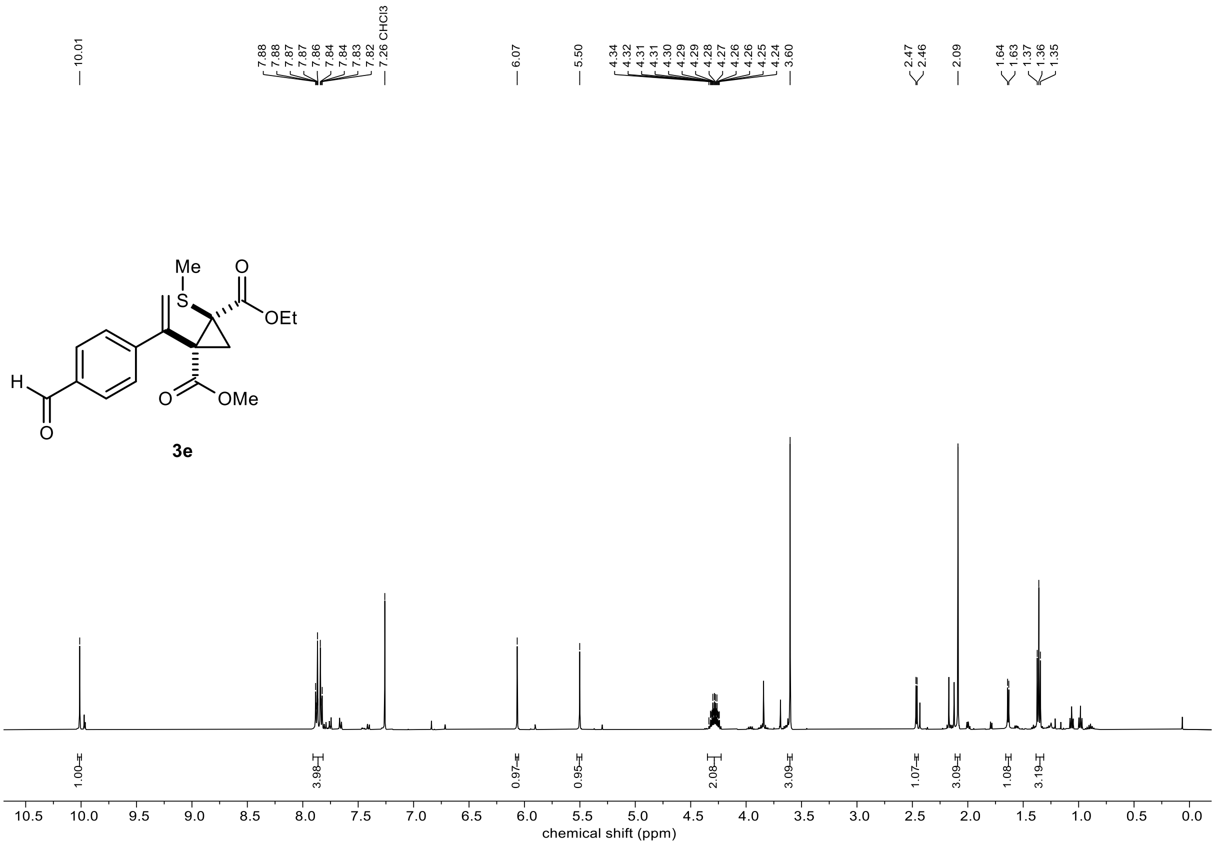

^{13}C NMR (126 MHz, $CDCl_3$)

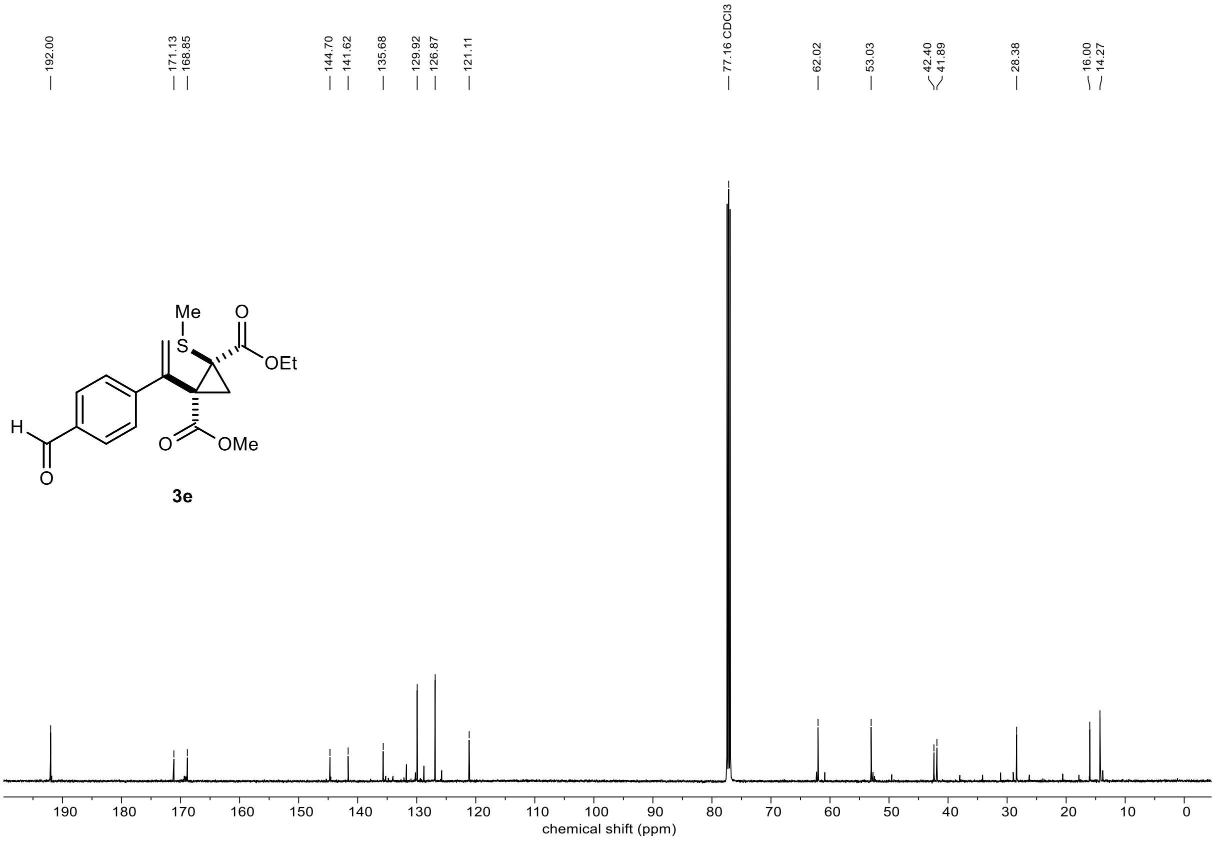

2-Ethyl 1-methyl 1-(1-(4-(ethoxycarbonyl)phenyl)vinyl)-2-(methylthio)cyclopropane-1,2-dicarboxylate (3f)

^{1}H NMR (500 MHz, $CDCl_3$)

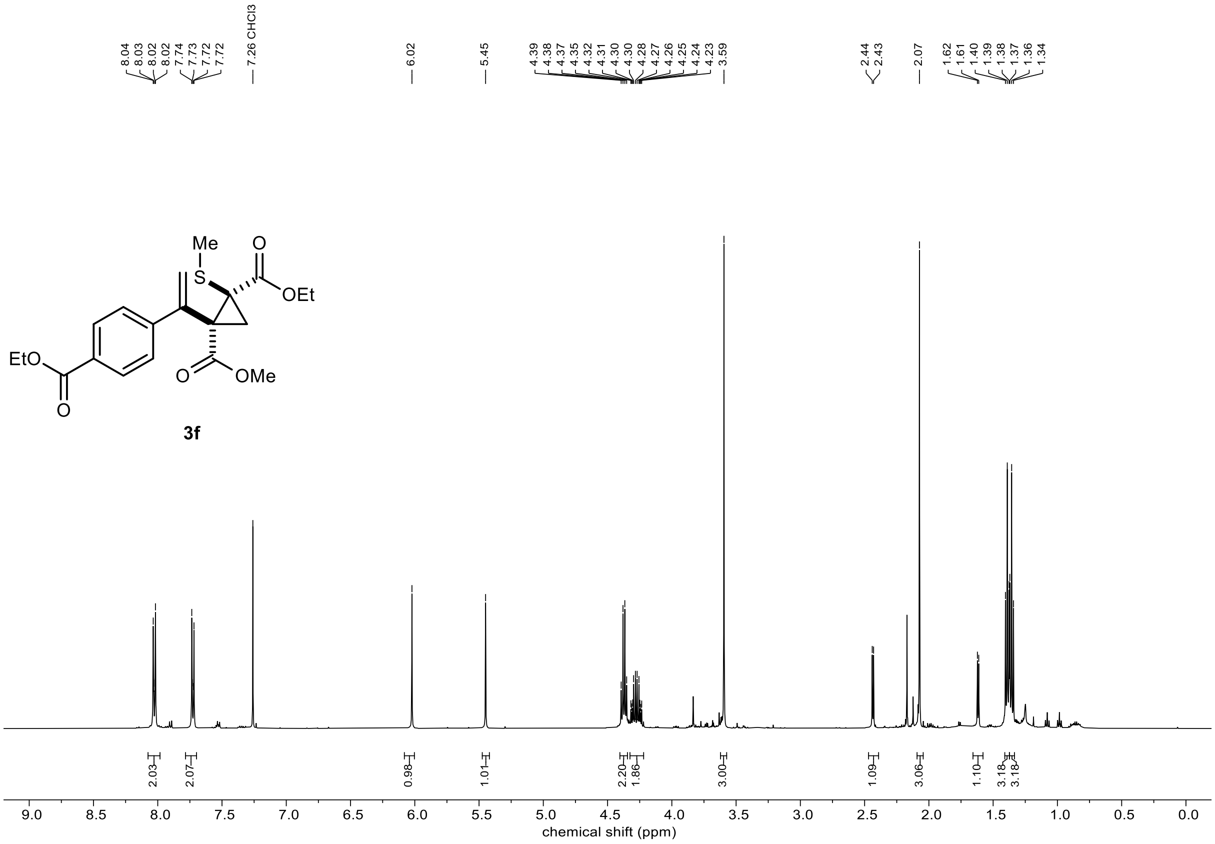

^{13}C NMR (126 MHz, $CDCl_3$)

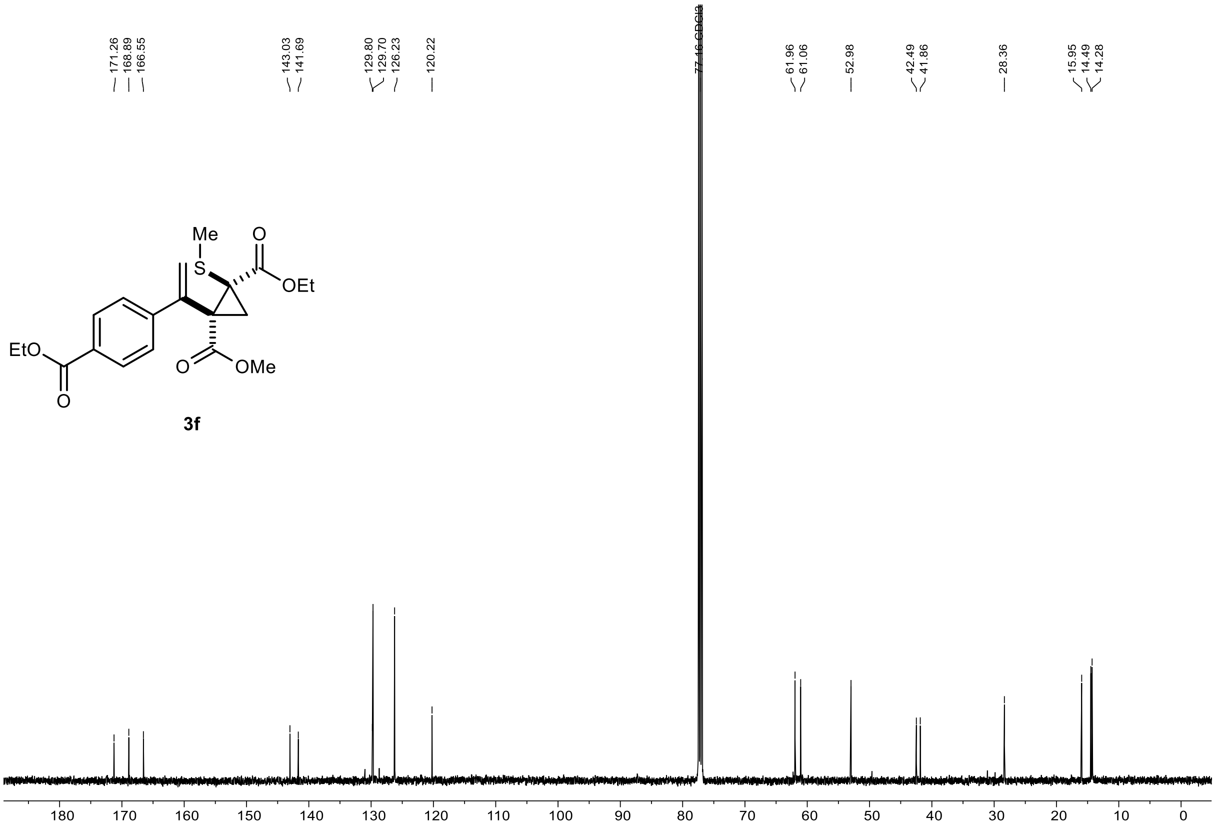

2-Ethyl 1-methyl 1-(1-(4-(dimethylcarbamoyl)phenyl)vinyl)-2-(methylthio)cyclopropane-1,2-dicarboxylate (3g)

^{1}H NMR (500 MHz, $CDCl_3$)

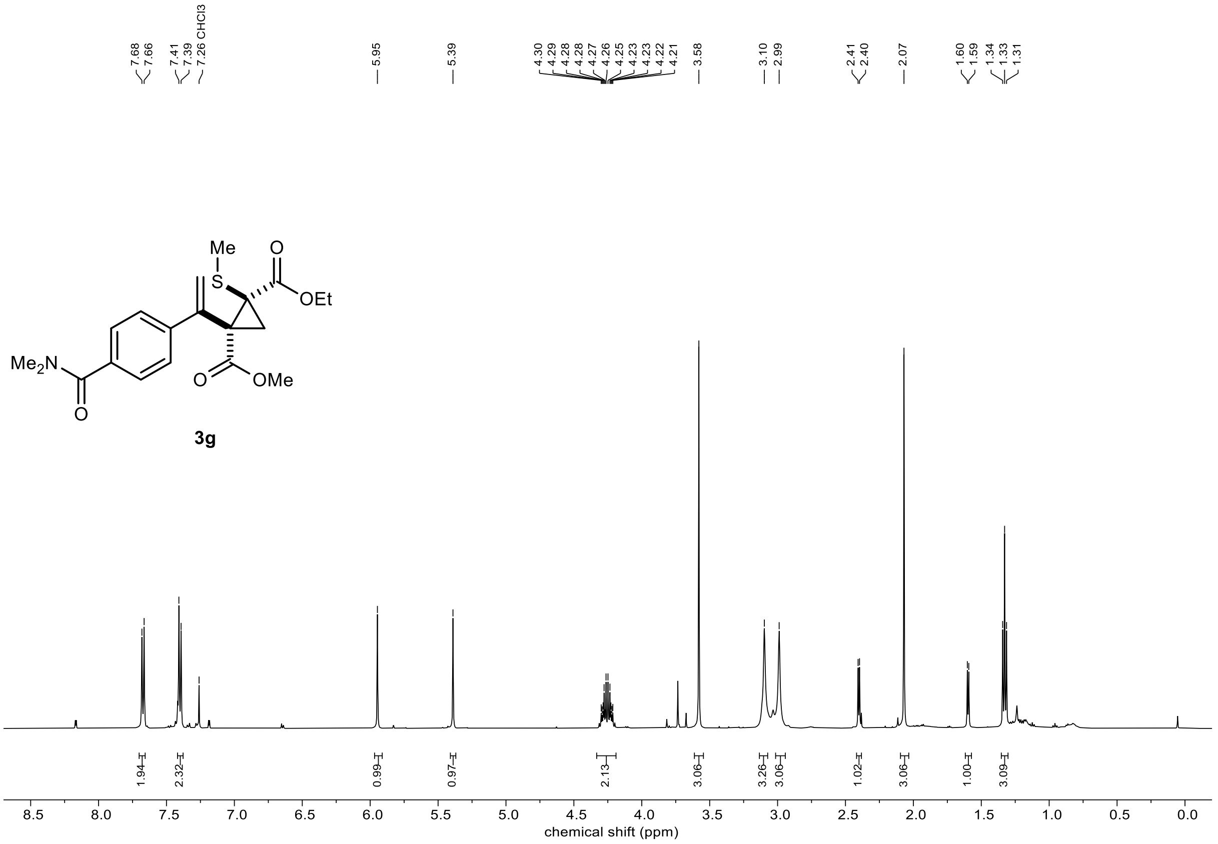

^{13}C NMR (126 MHz, $CDCl_3$)

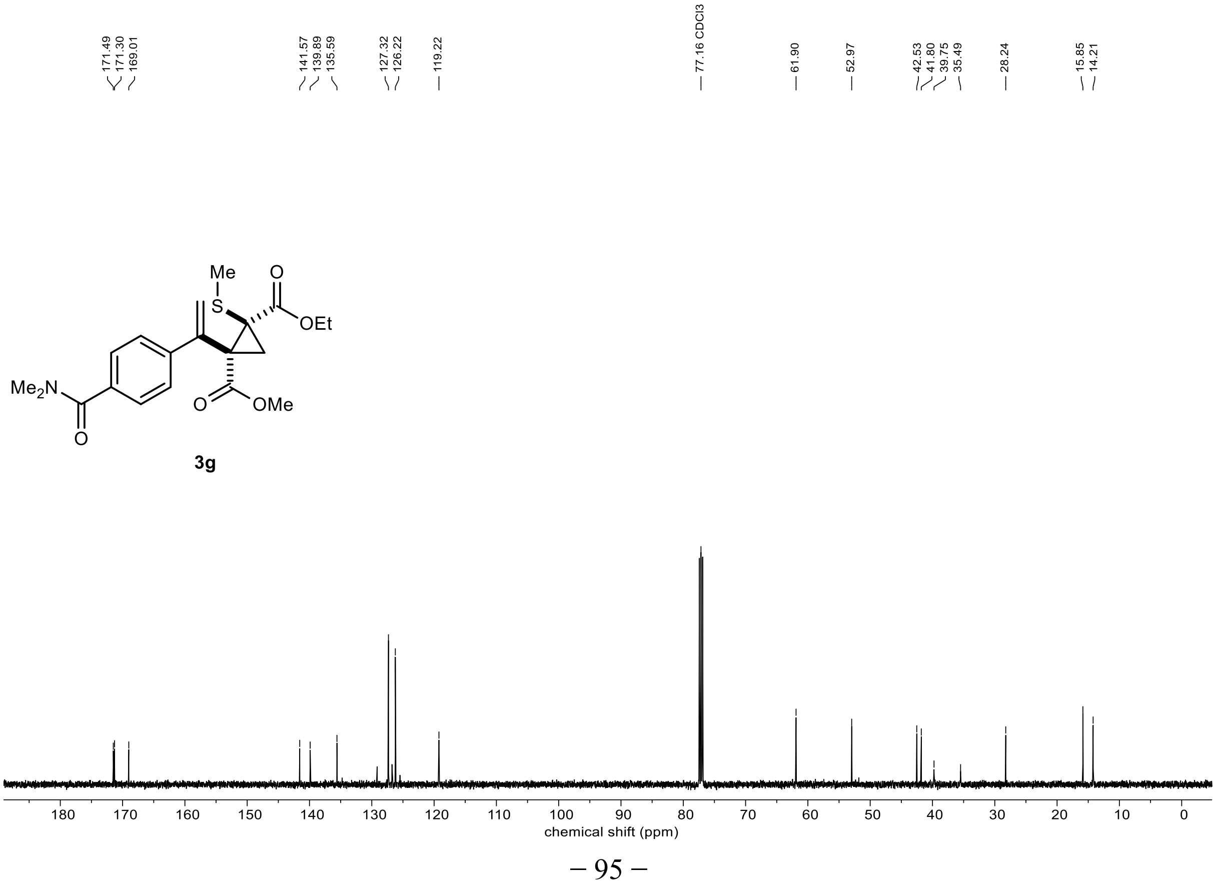

2-Ethyl 1-methyl 1-(1-(2-chlorophenyl)vinyl)-2-(methylthio)cyclopropane-1,2-dicarboxylate (3h)

^{1}H NMR (500 MHz, $CDCl_3$)

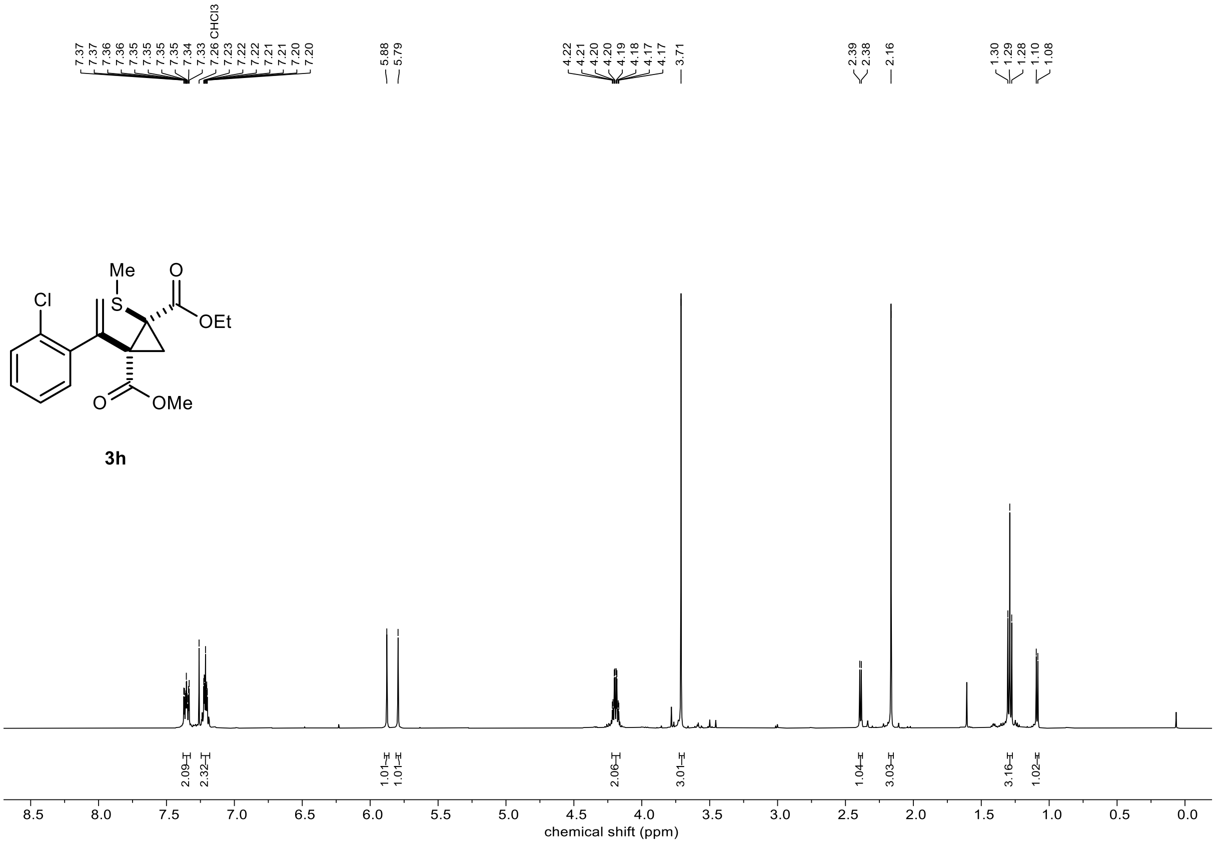

^{13}C NMR (126 MHz, $CDCl_3$)

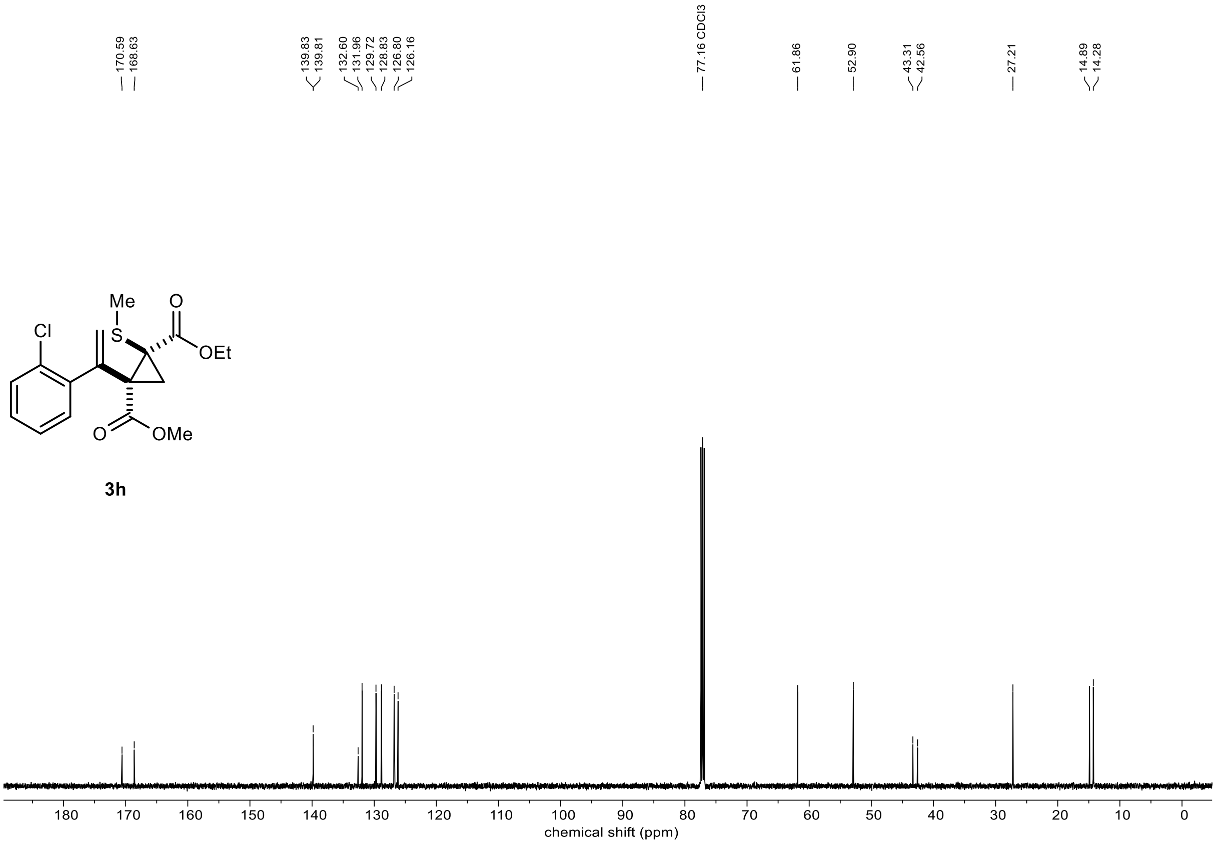

1-Ethyl 2-methyl 1-(methylthio)-2-(1-(3-nitrophenyl)vinyl)cyclopropane-1,2-dicarboxylate (3i)

^{1}H NMR (500 MHz, $CDCl_3$)

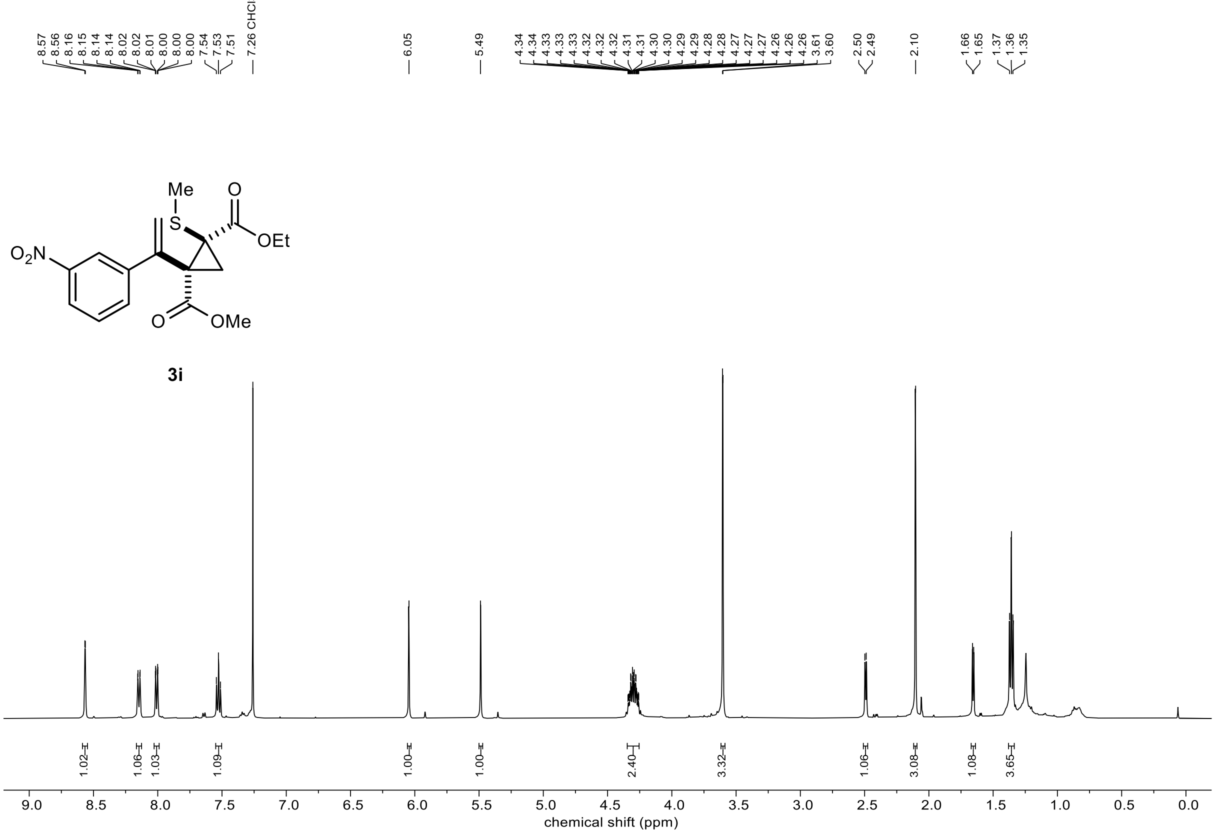

^{13}C NMR (126 MHz, $CDCl_3$)

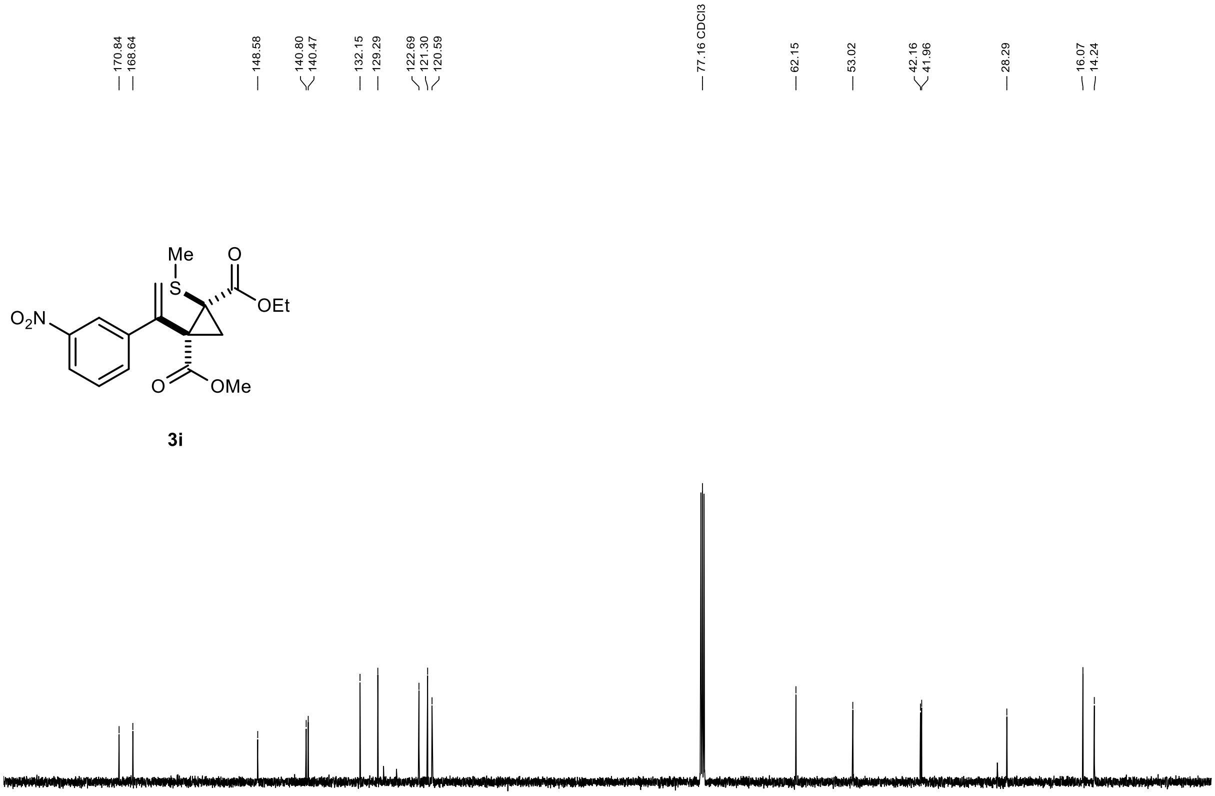

1-Ethyl 2-methyl 1-(methylthio)-2-(1-(thiophen-2-yl)vinyl)cyclopropane-1,2-dicarboxylate (3j)

^{1}H NMR (500 MHz, $CDCl_3$)

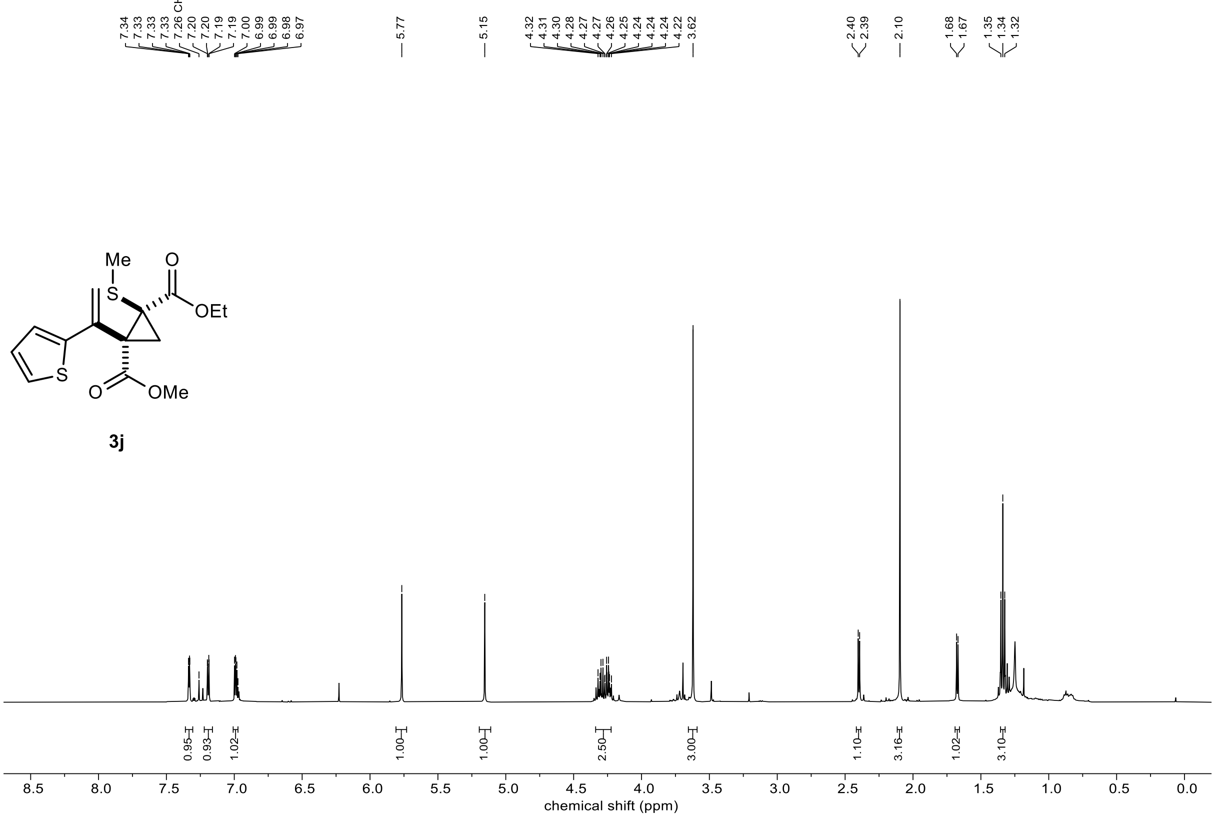

^{13}C NMR (126 MHz, $CDCl_3$)

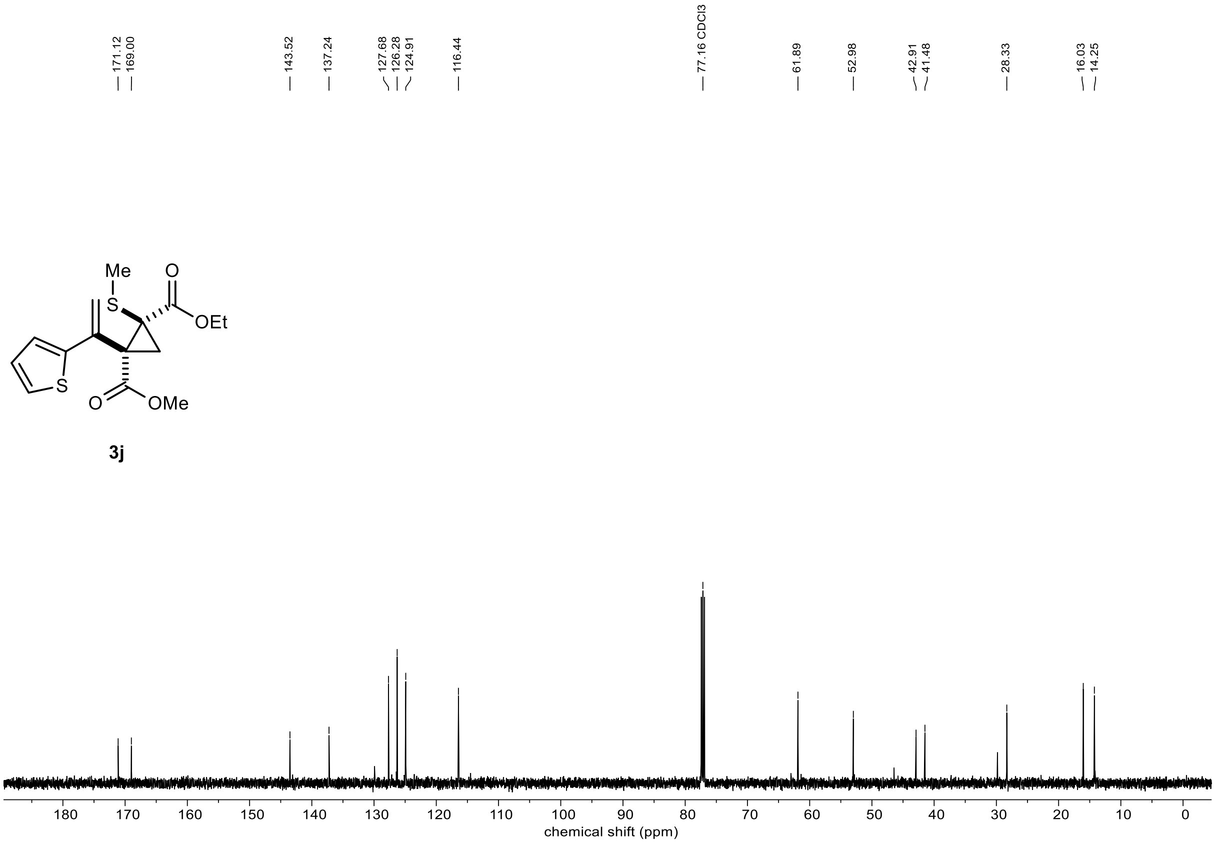

2-Ethyl 1-methyl 1-(1-(1-(*tert*-butoxycarbonyl)-1H-indol-5-yl)vinyl)-2-(methylthio)cyclopropane-1,2-dicarboxylate (3k)

^{1}H NMR (500 MHz, $CDCl_3$)

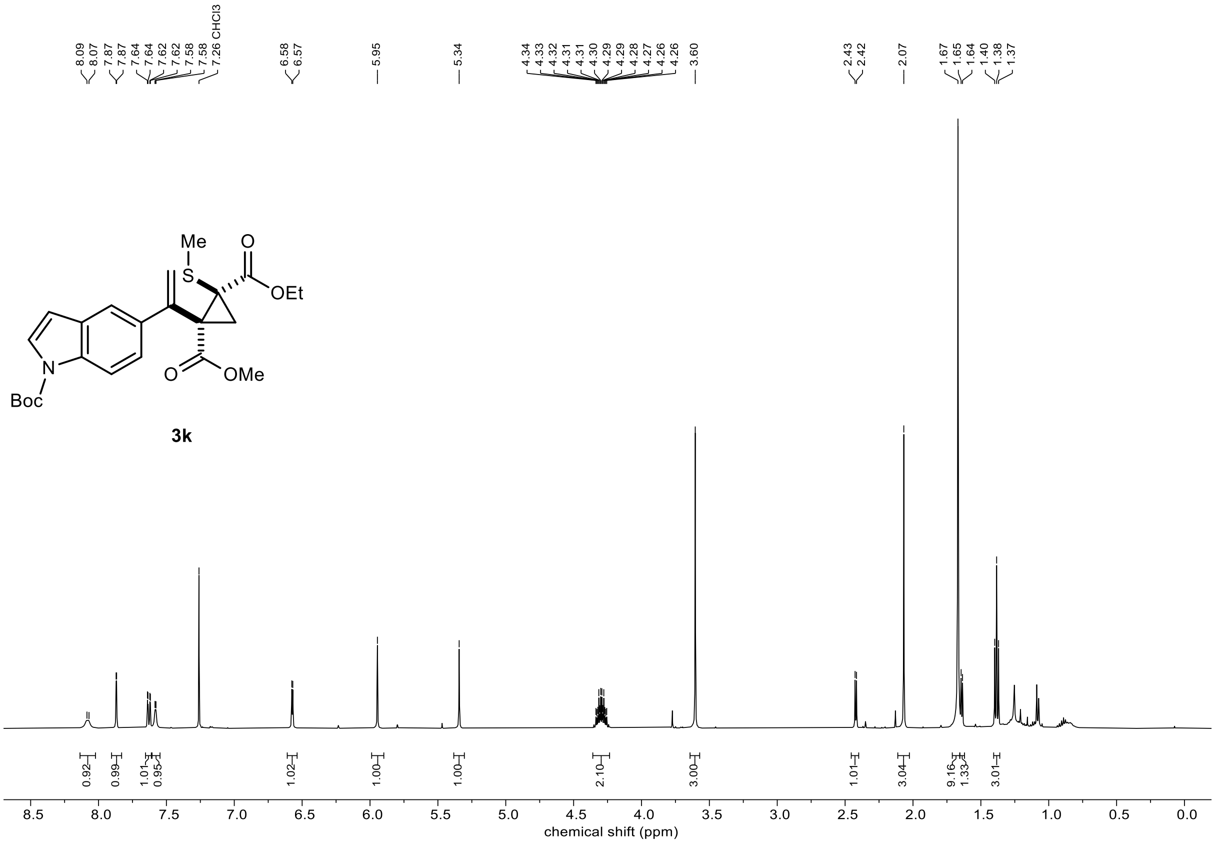

^{13}C NMR (126 MHz, $CDCl_3$)

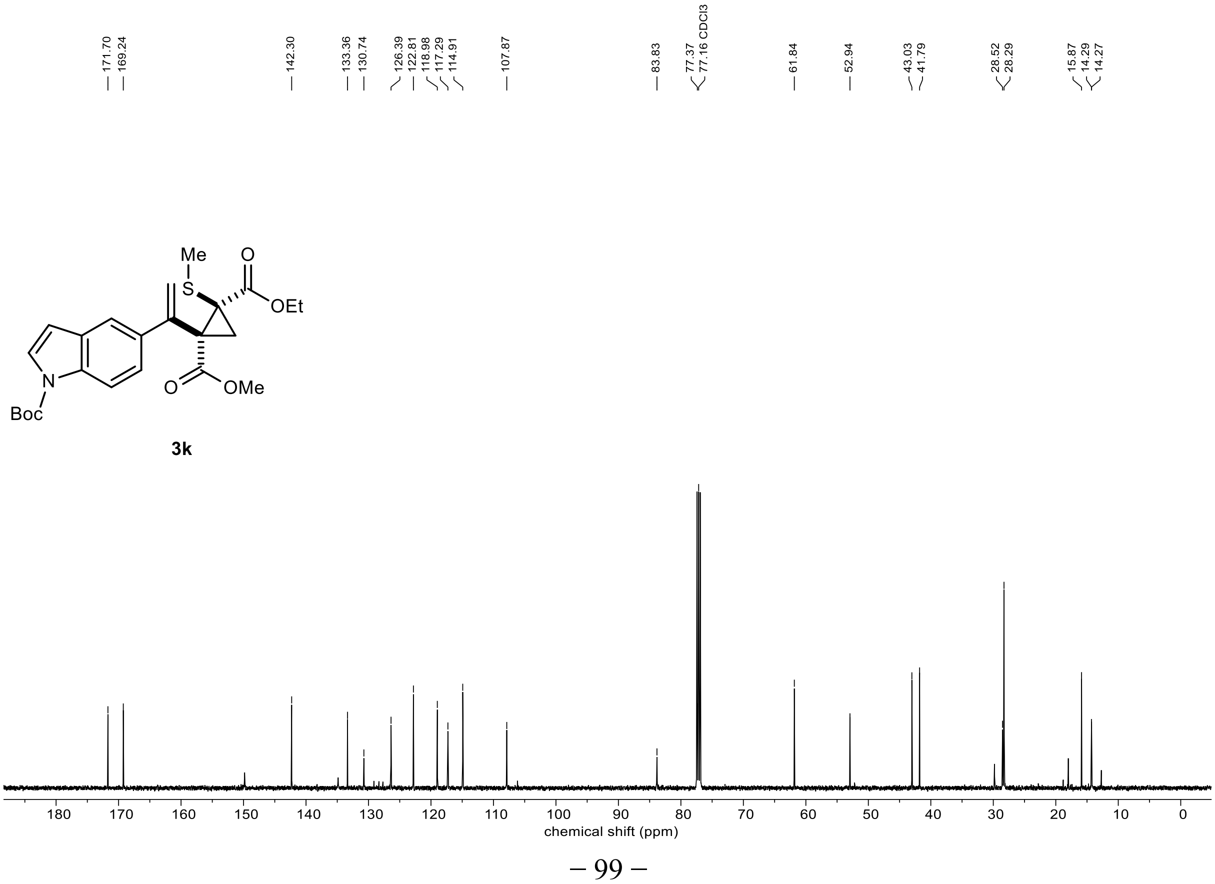

1-Ethyl 2-methyl -1-(methylthio)-2-(prop-1-en-2-yl)cyclopropane-1,2-dicarboxylate (3l)

^{1}H NMR (500 MHz, $CDCl_3$)

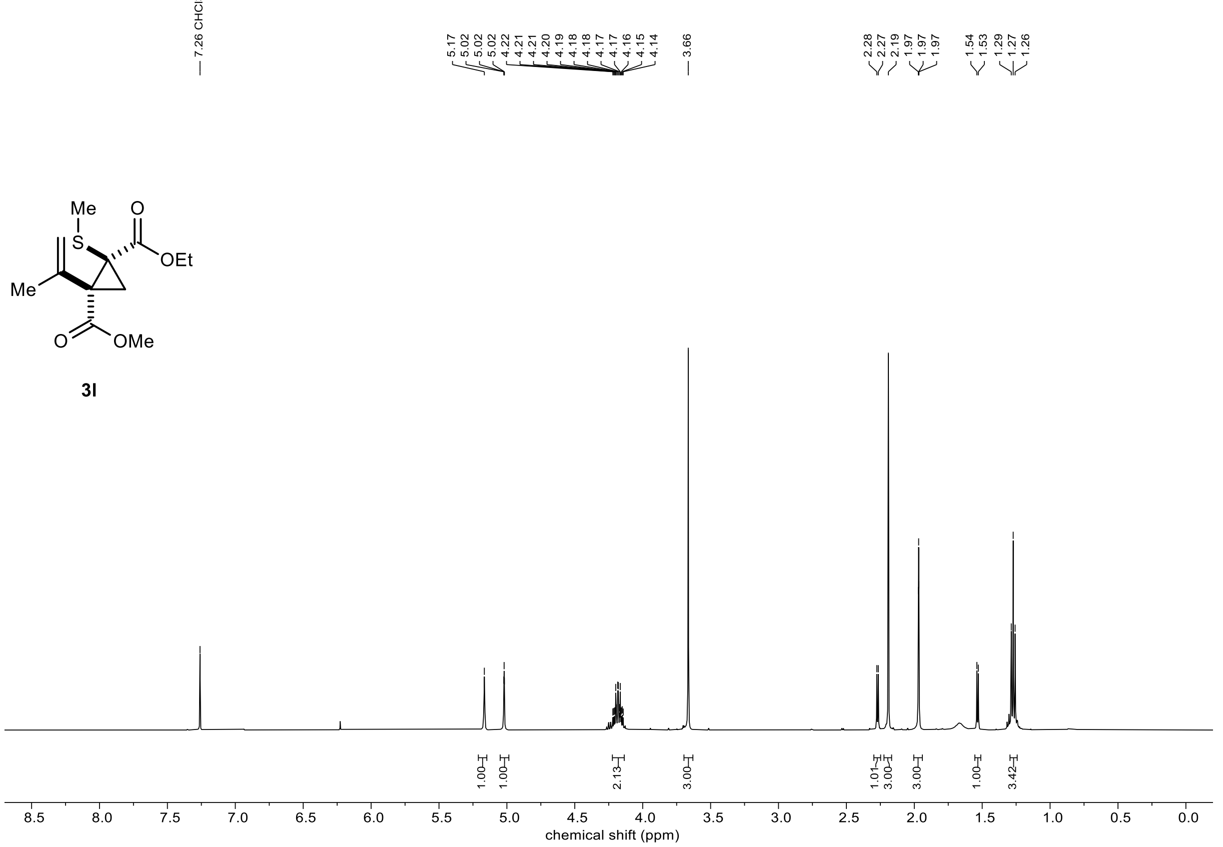

^{13}C NMR (126 MHz, $CDCl_3$)

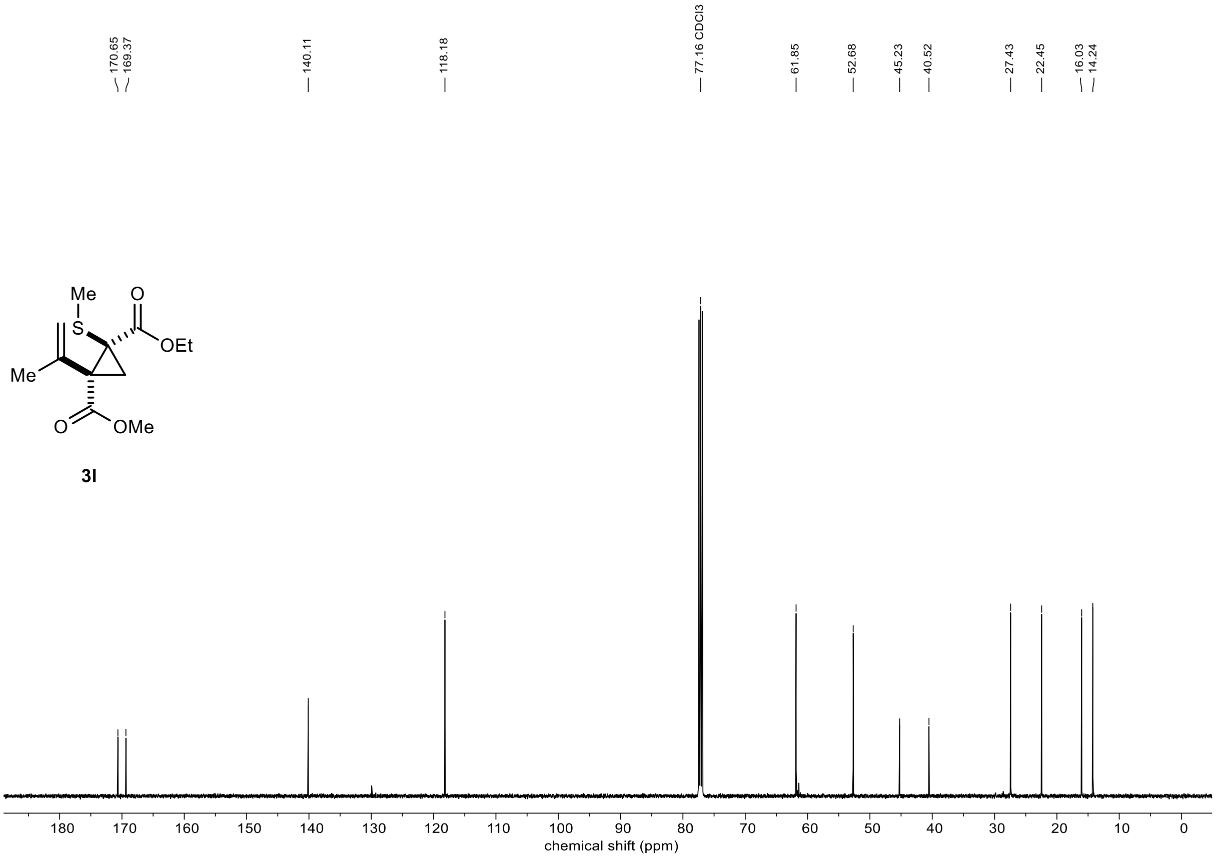

1-Ethyl 2-methyl 1-(methylthio)-2-(1-((triisopropylsilyl)oxy)vinyl)cyclopropane-1,2-dicarboxylate (3m)

^{1}H NMR (500 MHz, $CDCl_3$)

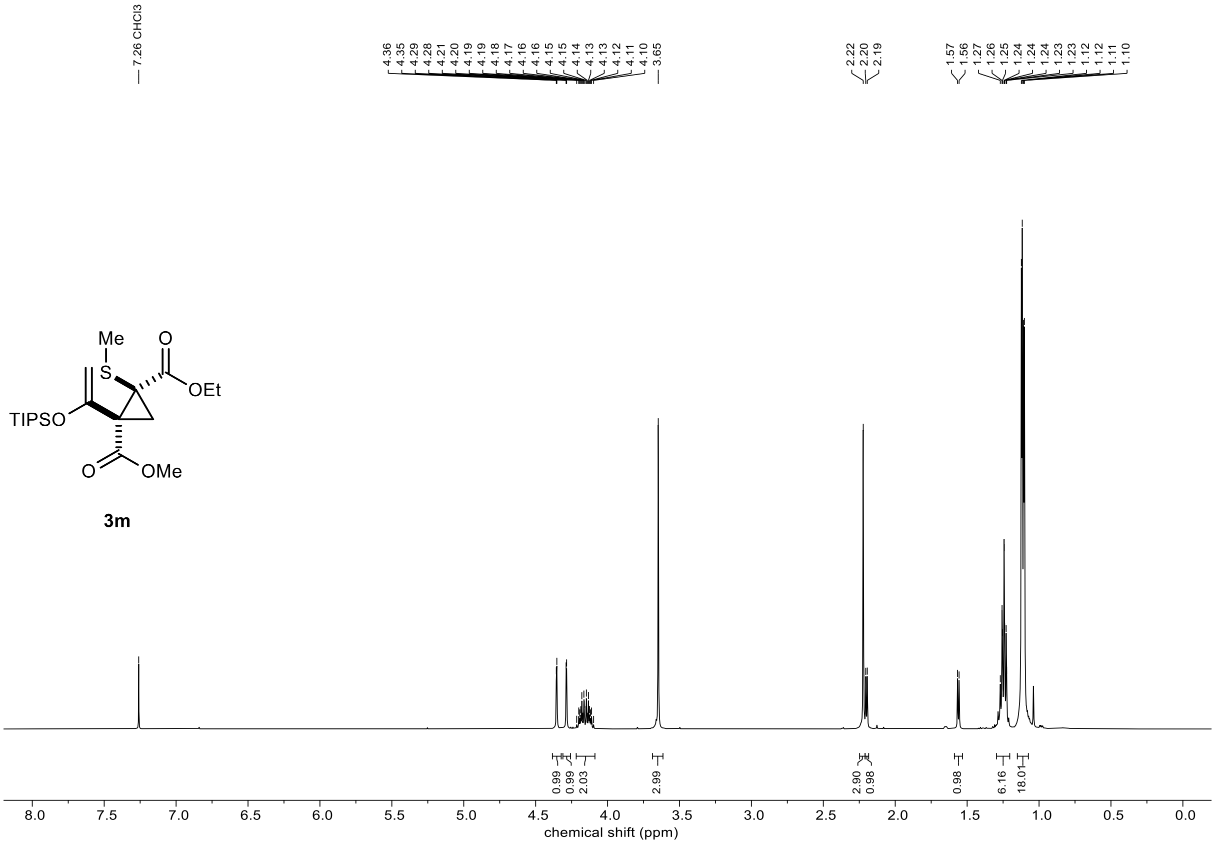

^{13}C NMR (126 MHz, $CDCl_3$)

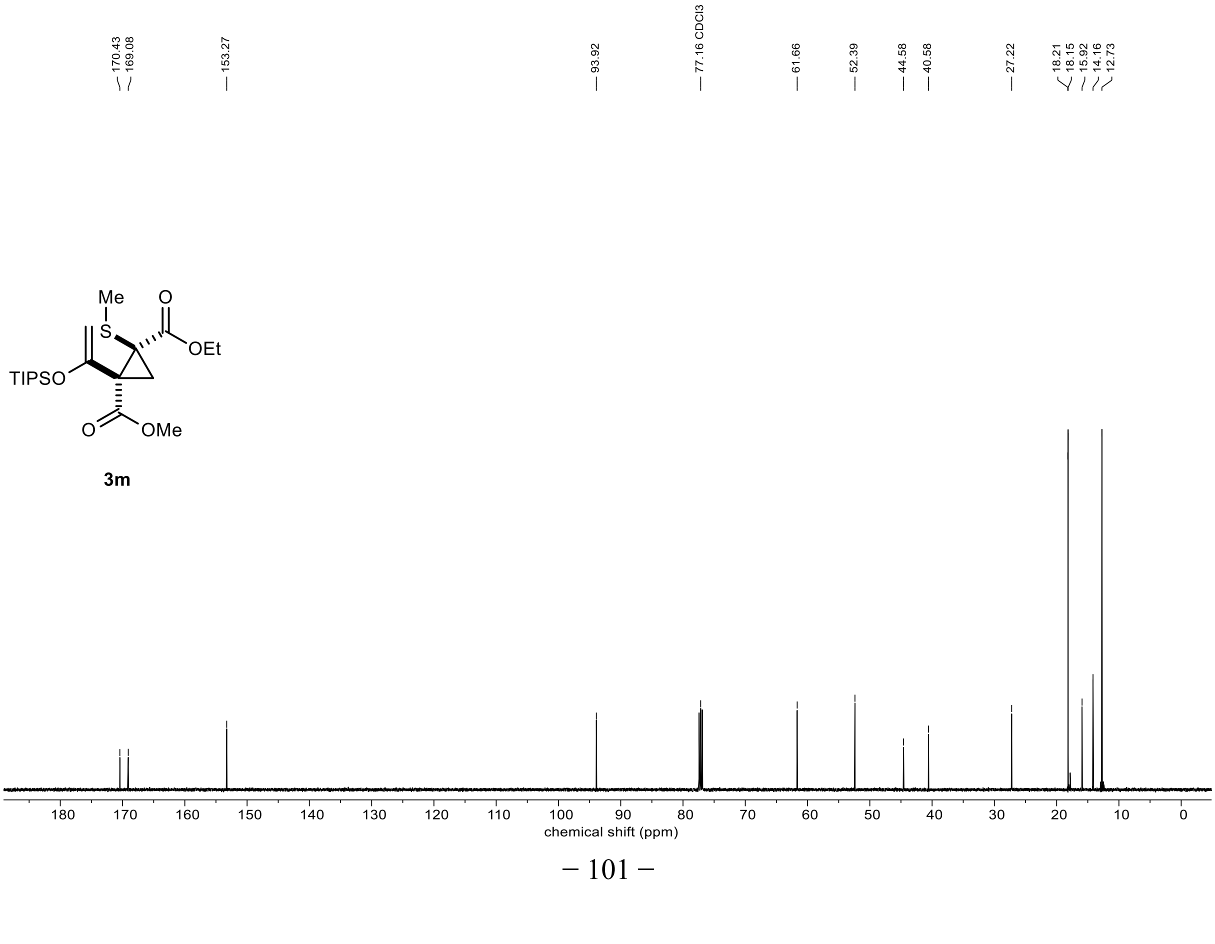

2-Ethyl 1-methyl 1-acetyl-2-(methylthio)cyclopropane-1,2-dicarboxylate (3n)

^{1}H NMR (500 MHz, $CDCl_3$)

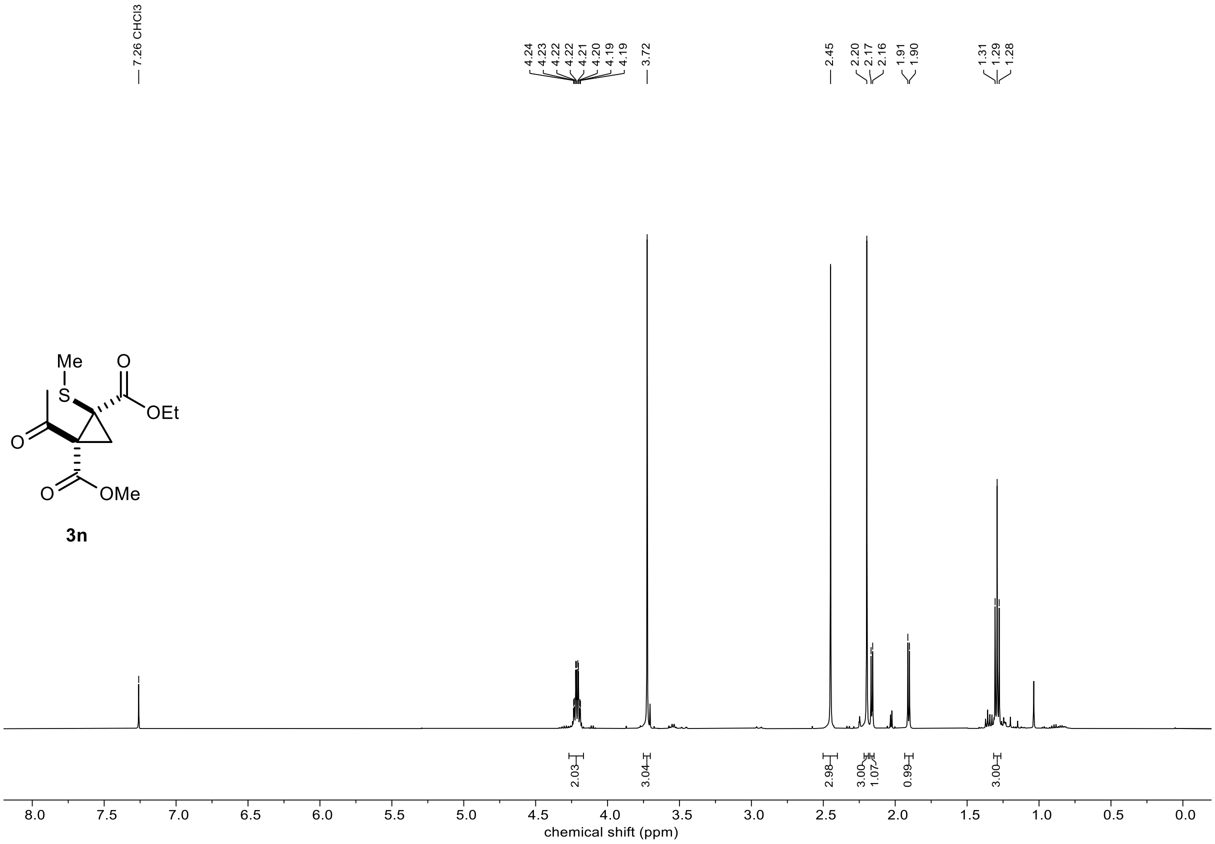

^{13}C NMR (126 MHz, $CDCl_3$)

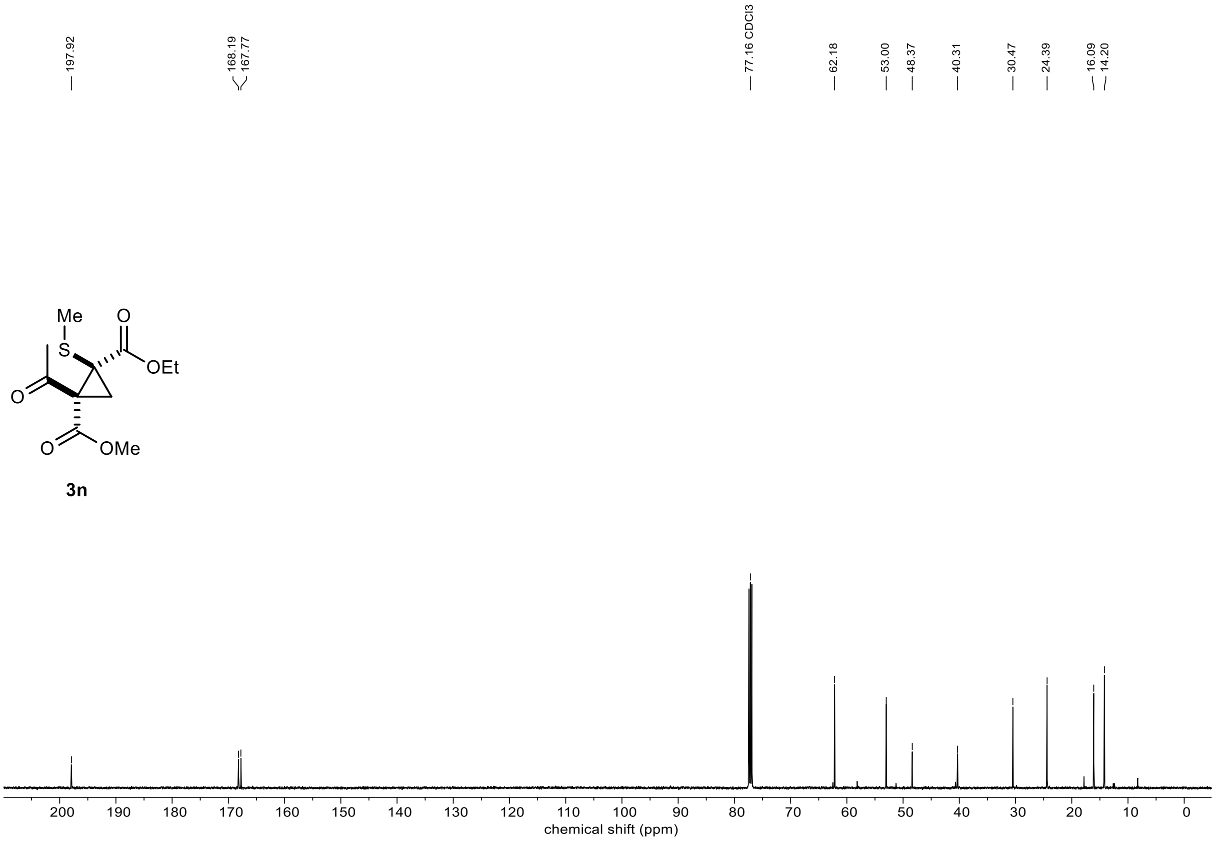

1-Ethyl 2-methyl 1-(methylthio)-2-((*E*)-1-phenylprop-1-en-1-yl)cyclopropane-1,2-dicarboxylate (3o)

^{1}H NMR (500 MHz, $CDCl_3$)

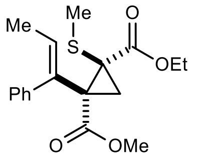

3o

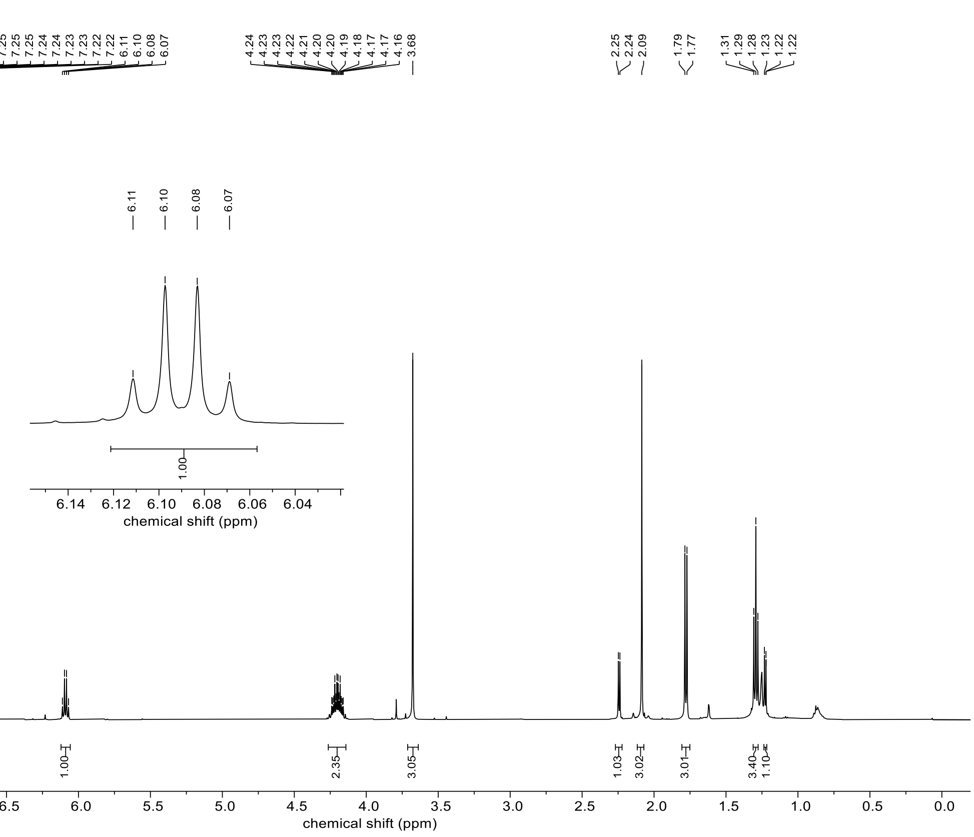

^{13}C NMR (126 MHz, $CDCl_3$)

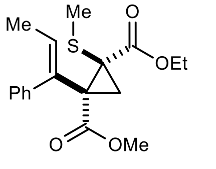

3o

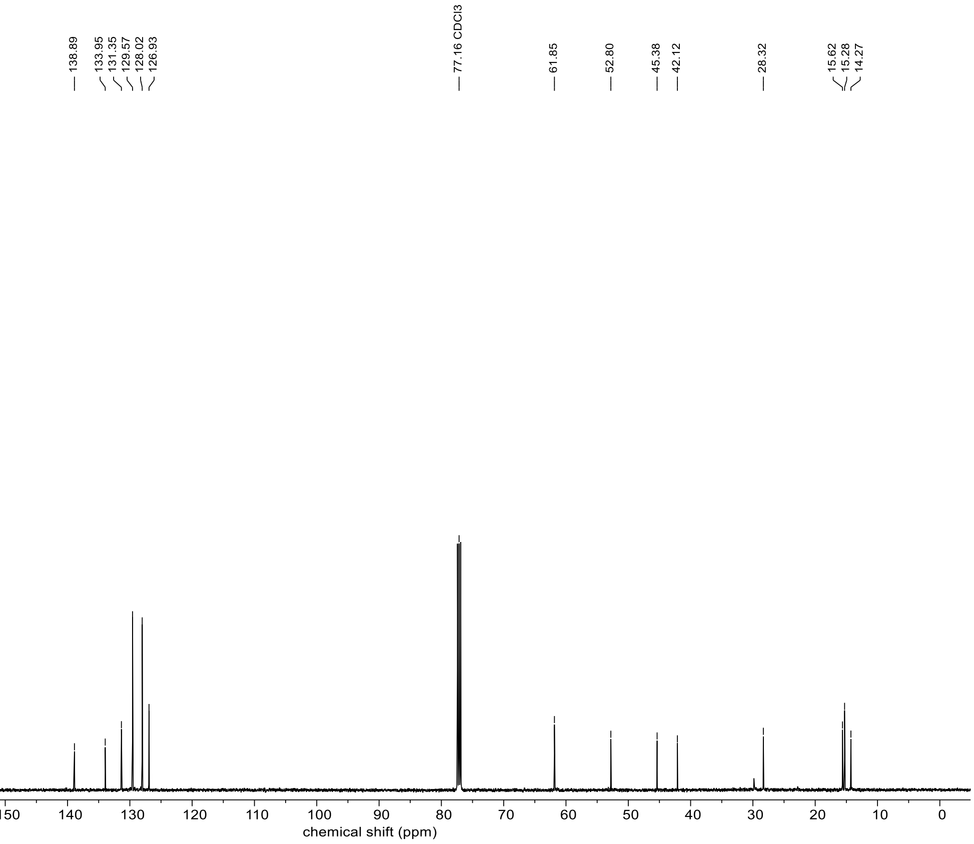

Ethyl 2-acetyl-1-(methylthio)-2-(1-phenylvinyl)cyclopropane-1-carboxylate (4a)

^{1}H NMR (500 MHz, $CDCl_3$)

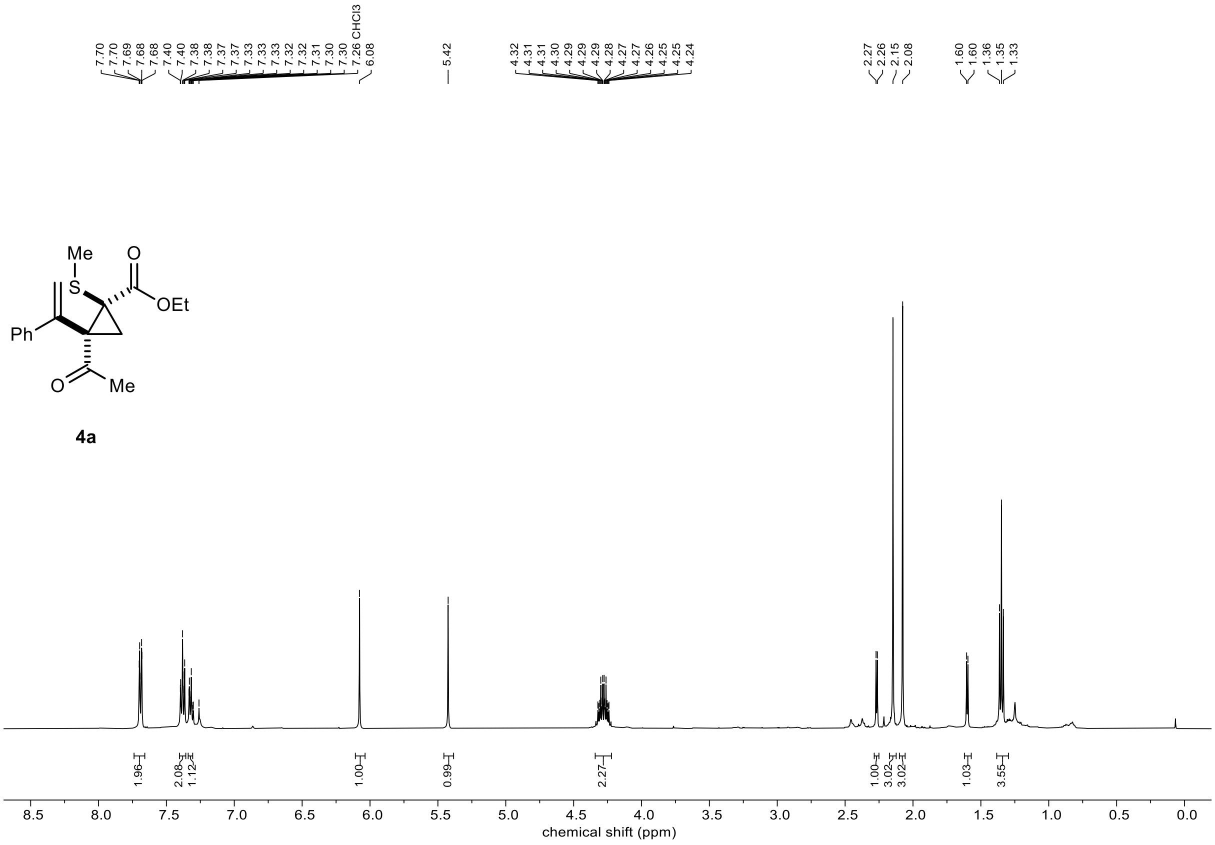

^{13}C NMR (126 MHz, $CDCl_3$)

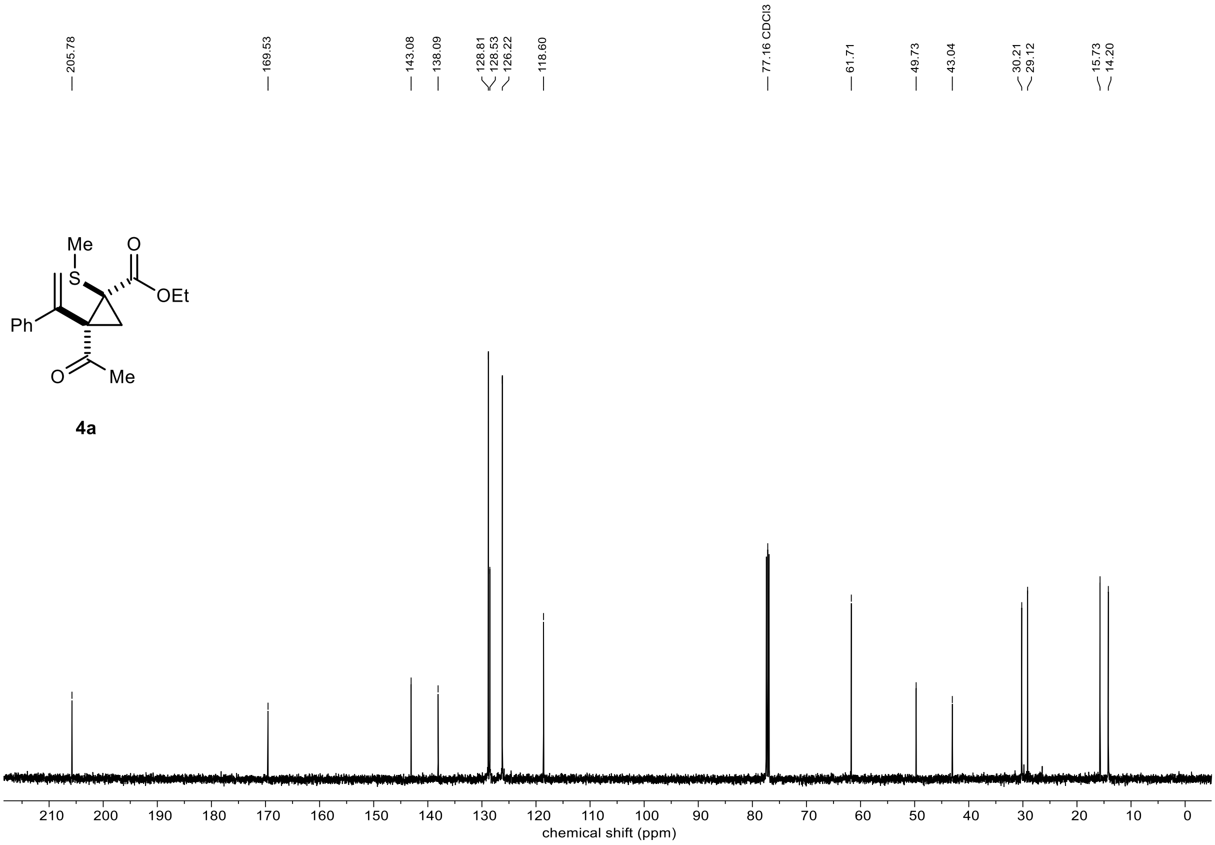

Ethyl 2-(methoxy(methyl)carbamoyl)-1-(methylthio)-2-(1-phenylvinyl)cyclopropane-1-carboxylate (4b)

^{1}H NMR (500 MHz, $CDCl_3$)

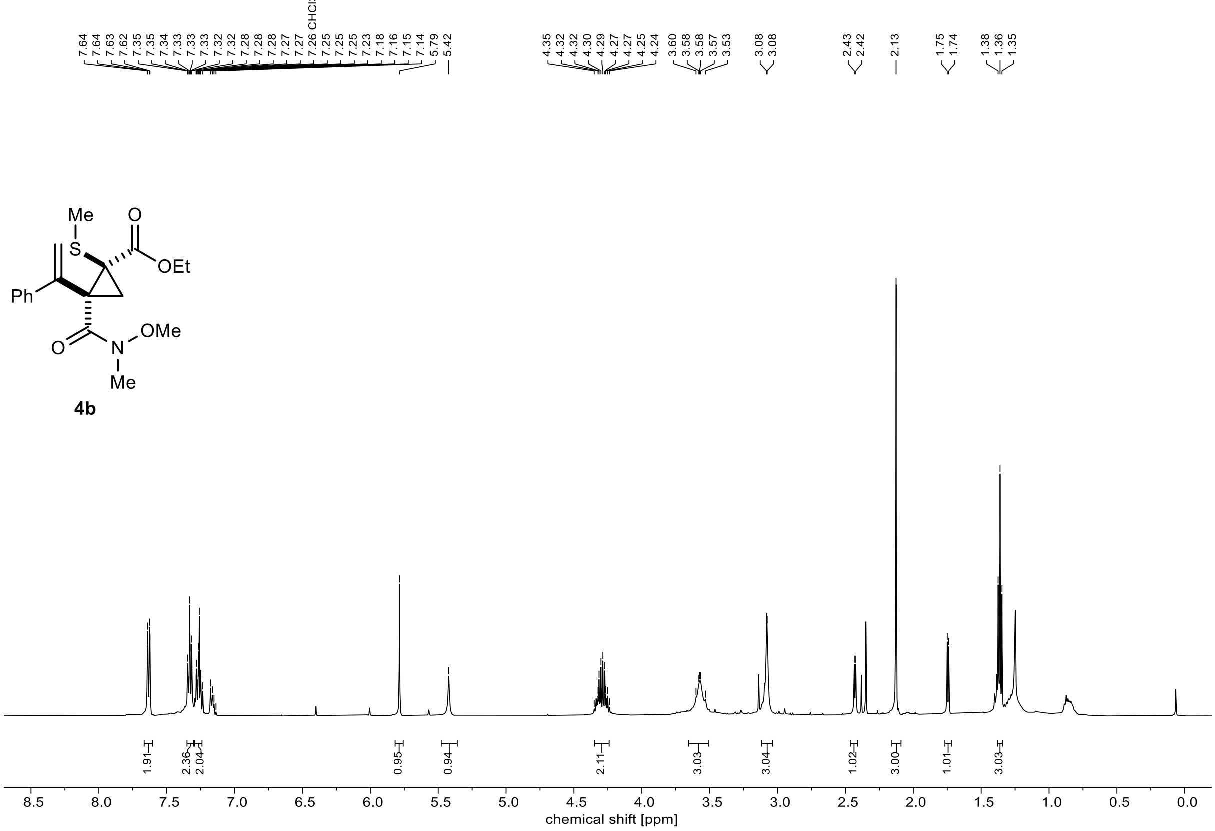

^{13}C NMR (126 MHz, $CDCl_3$)

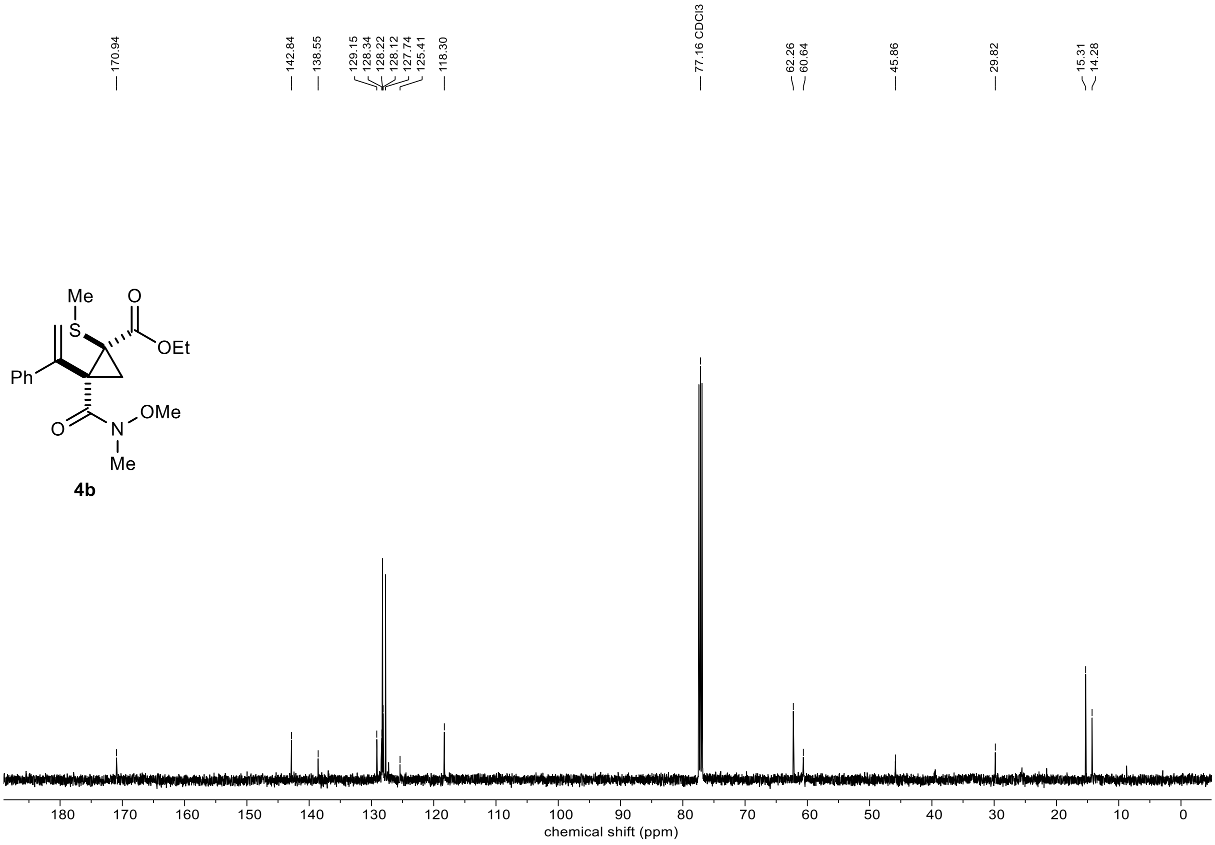

1-(Methylthio)-5-phenyl-3-azabicyclo[3.1.0]hexane-2,4-dione (4d)

^{1}H NMR (700 MHz, $CDCl_3$)

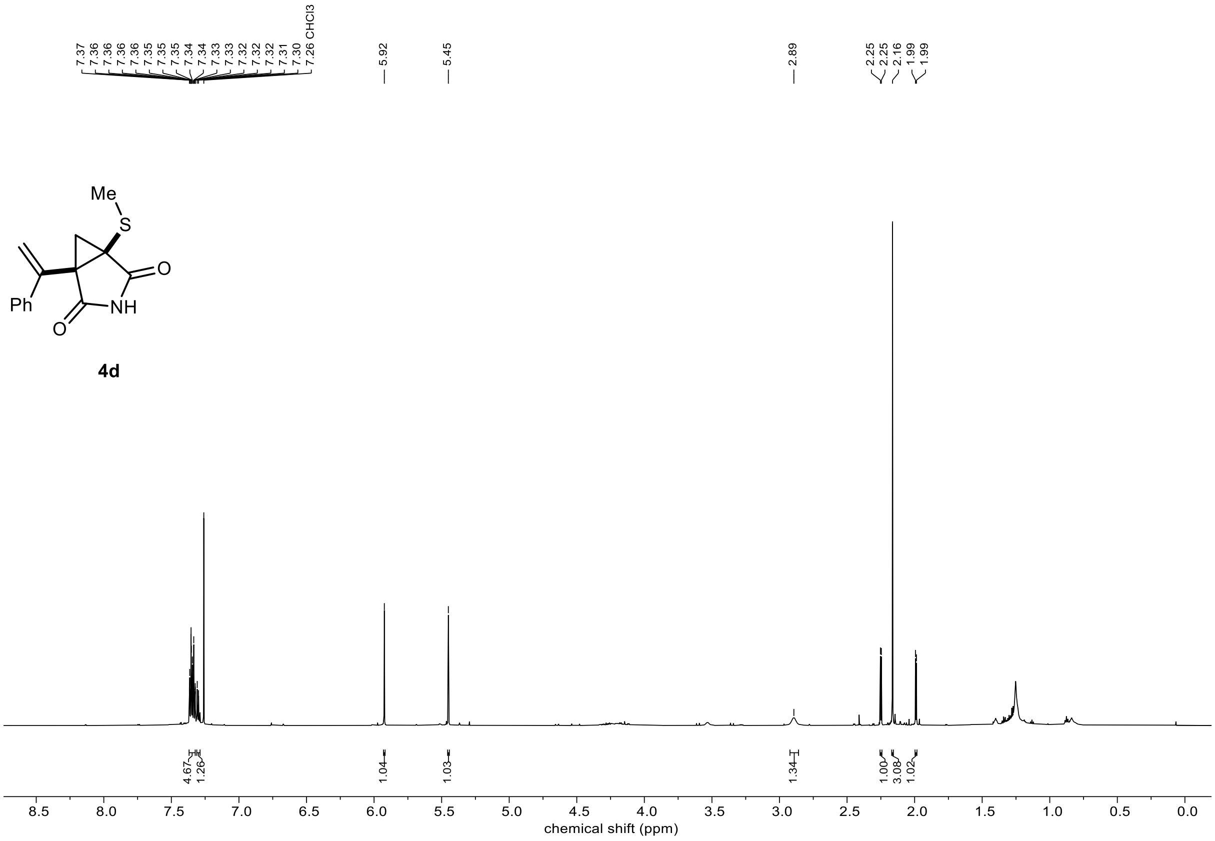

^{13}C NMR (176 MHz, $CDCl_3$)

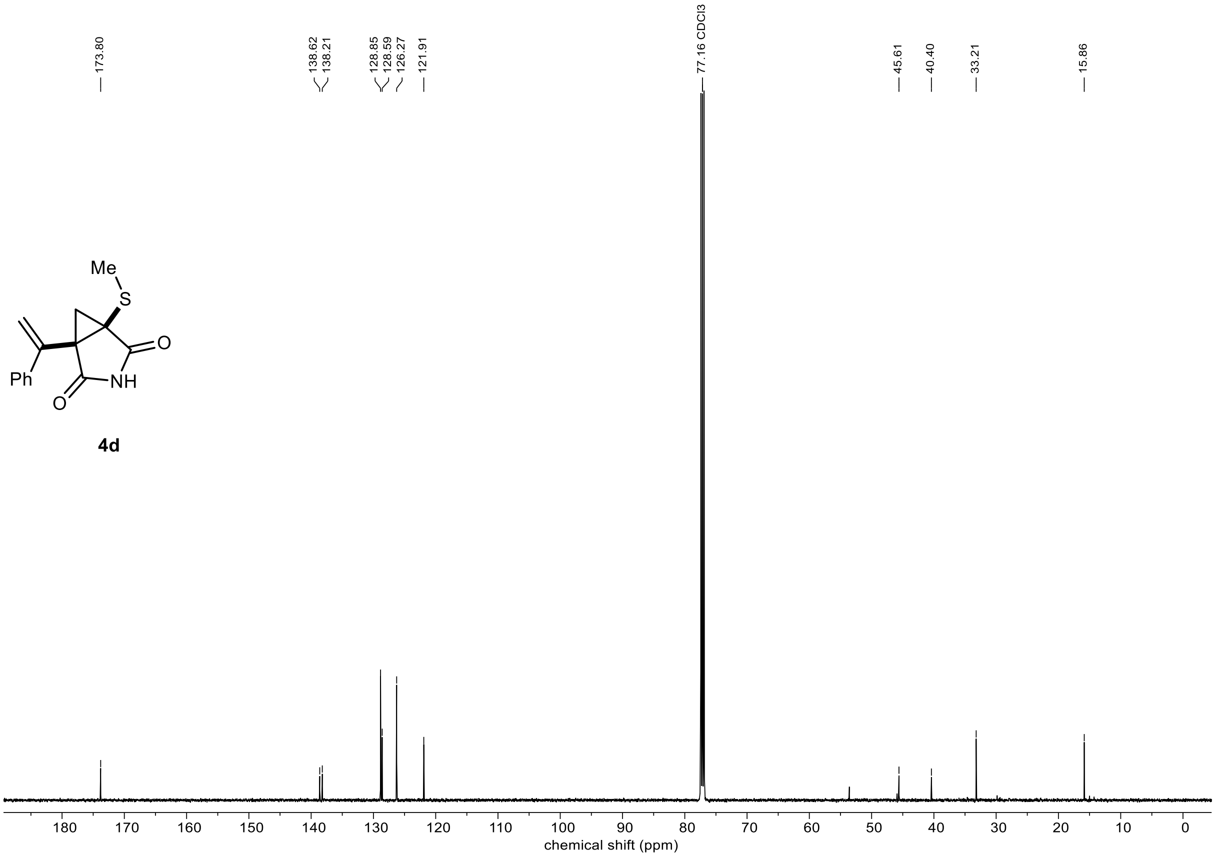

3-Methyl-1-(methylthio)-5-phenyl-3-azabicyclo[3.1.0]hexane-2,4-dione (4e)

^{1}H NMR (500 MHz, $CDCl_3$)

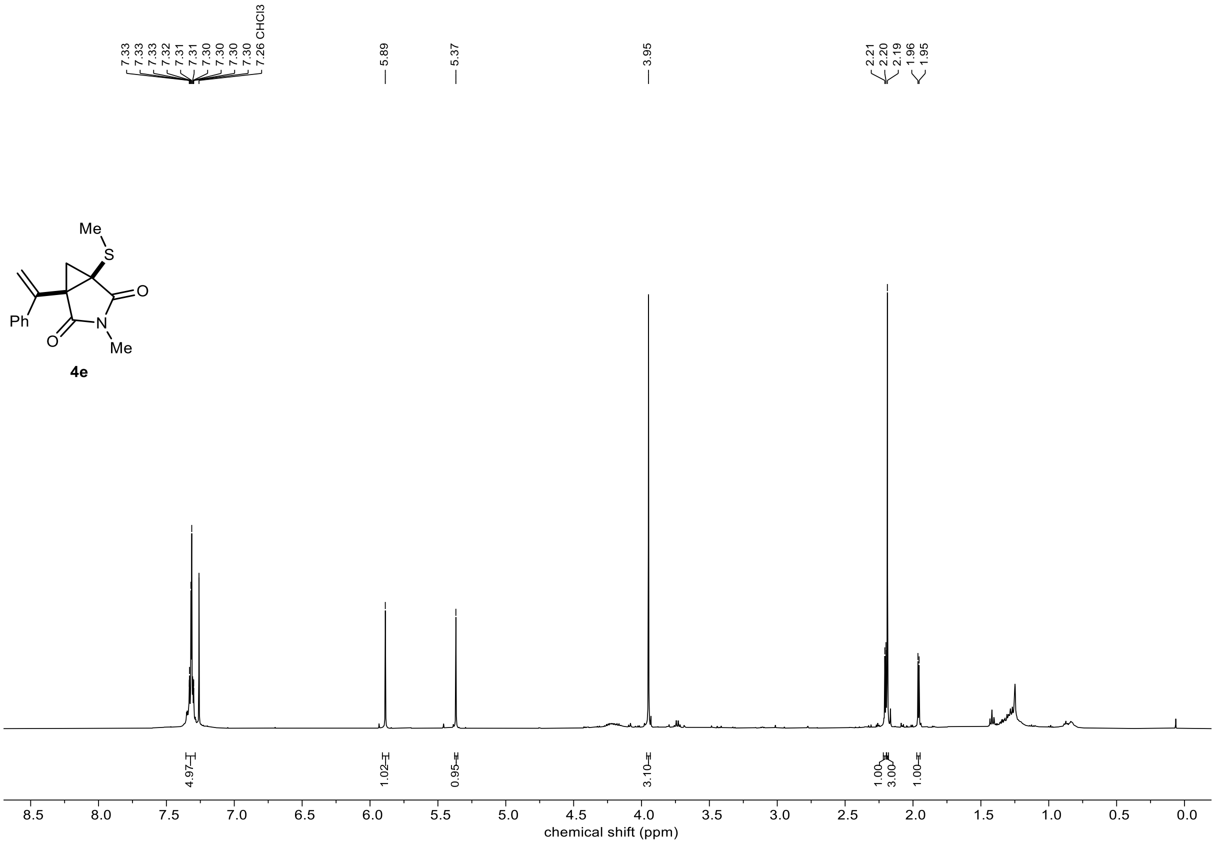

^{13}C NMR (126 MHz, $CDCl_3$)

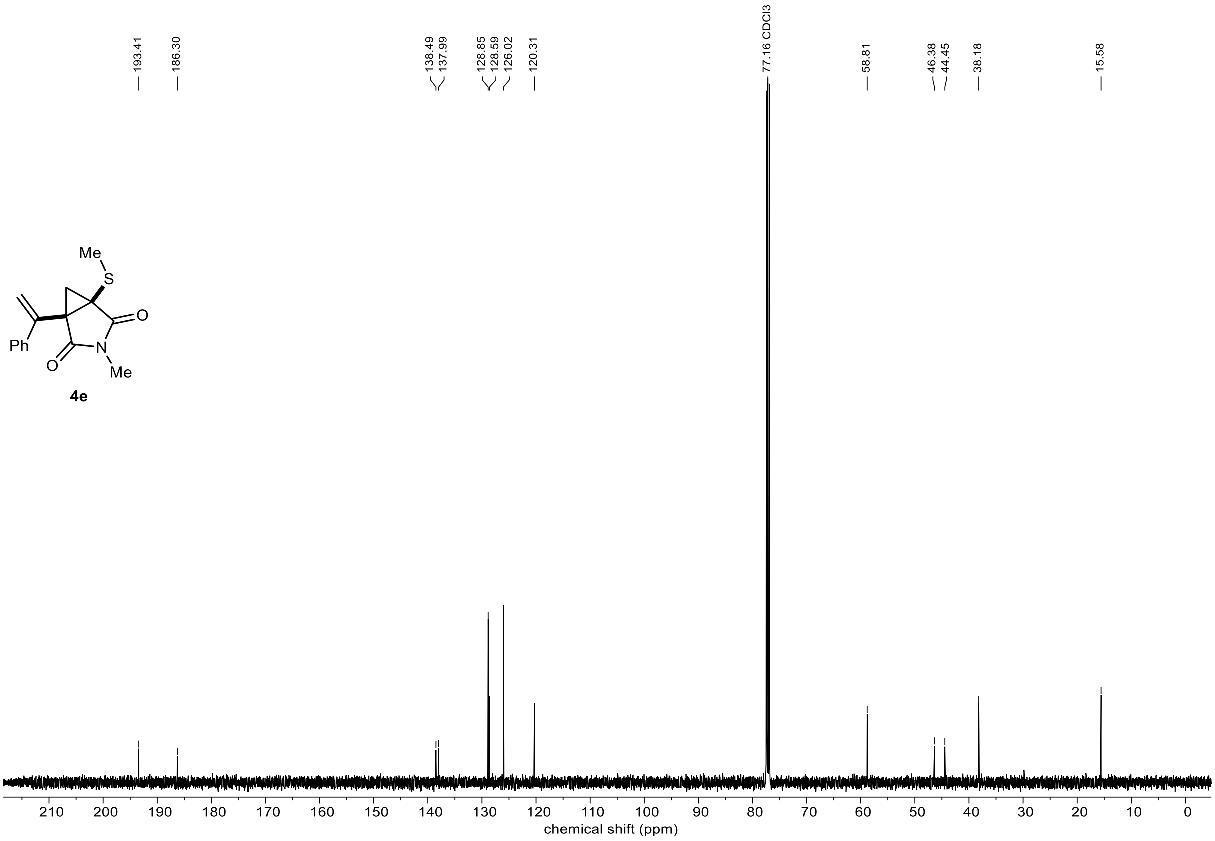

1-(Methylthio)-5-phenyl-3-oxabicyclo[3.1.0]hexan-2-one (4g)

^{1}H NMR (500 MHz, CDCl$_3$)

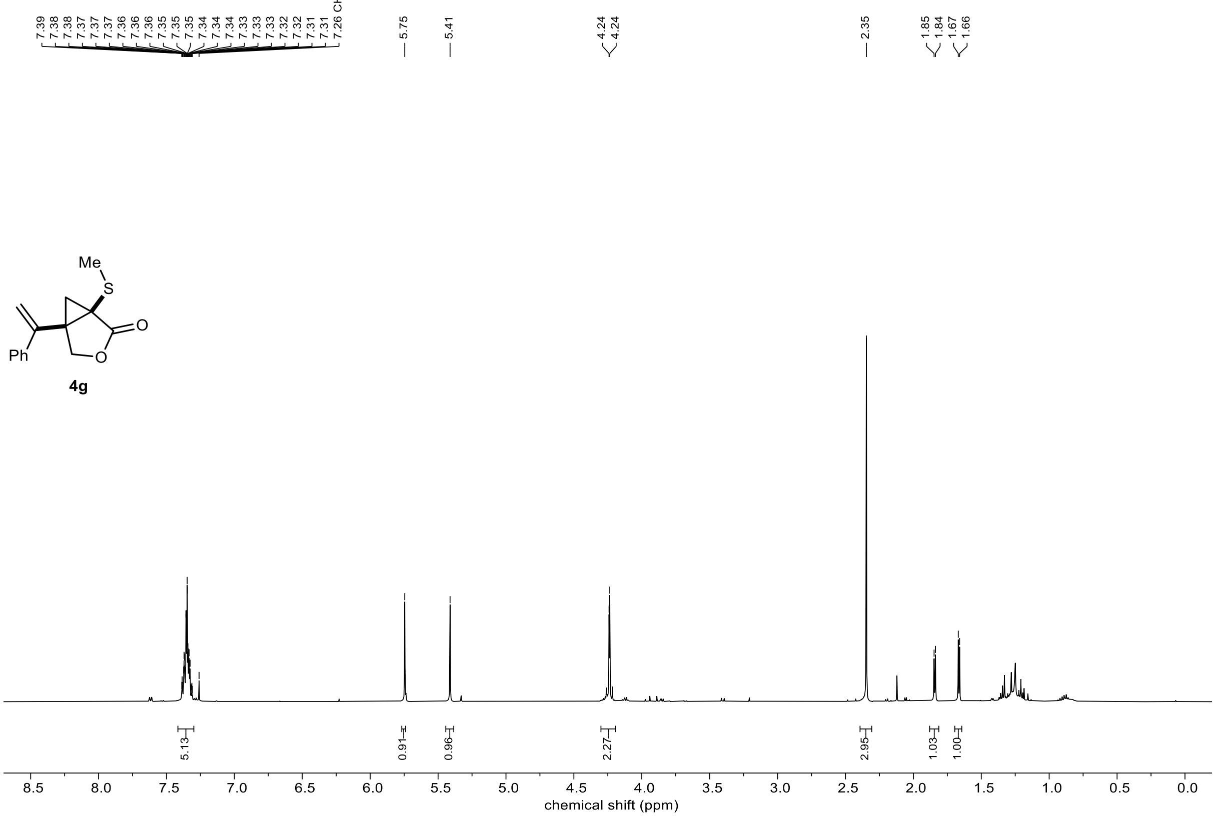

^{13}C NMR (126 MHz, CDCl$_3$)

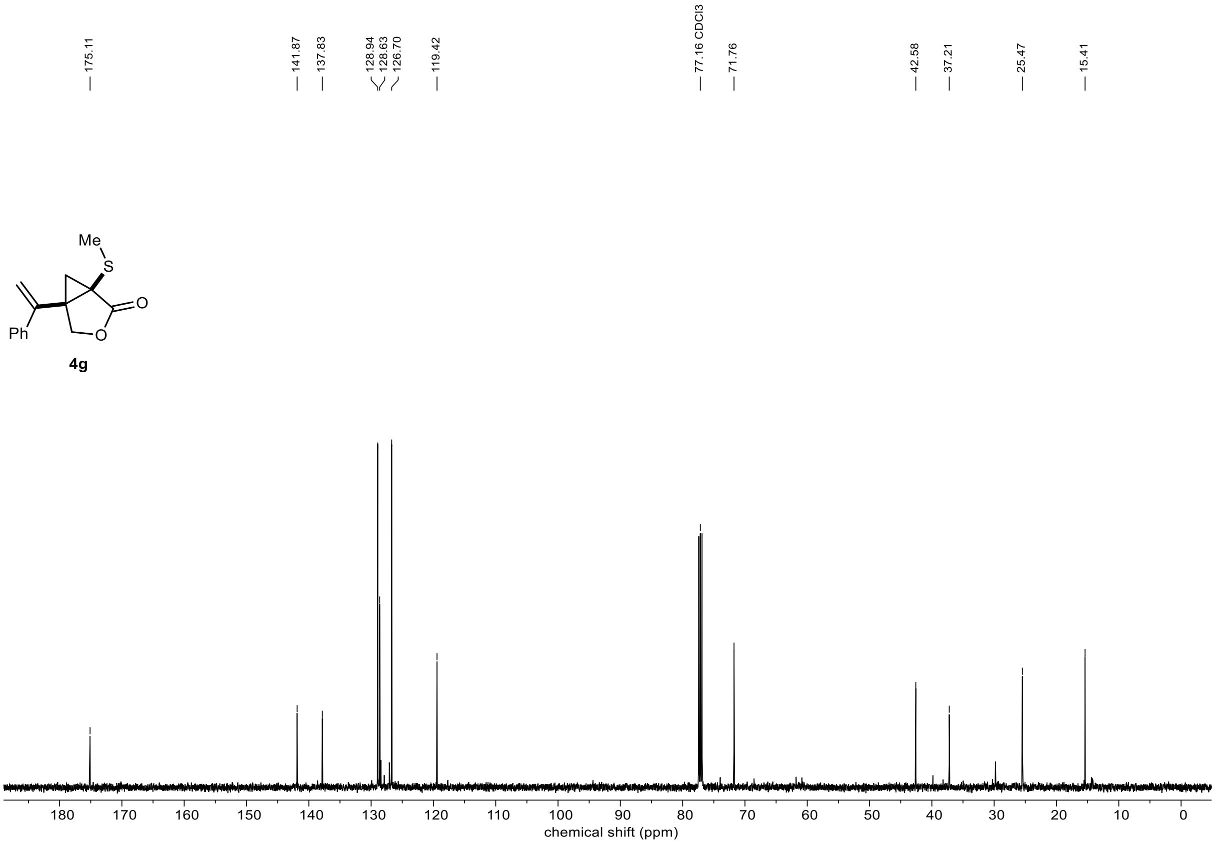

Ethyl 2-(methoxymethyl)-1-(methylthio)-2-(1-phenylvinyl)cyclopropane-1-carboxylate (4h)

^{1}H NMR (500 MHz, $CDCl_3$)

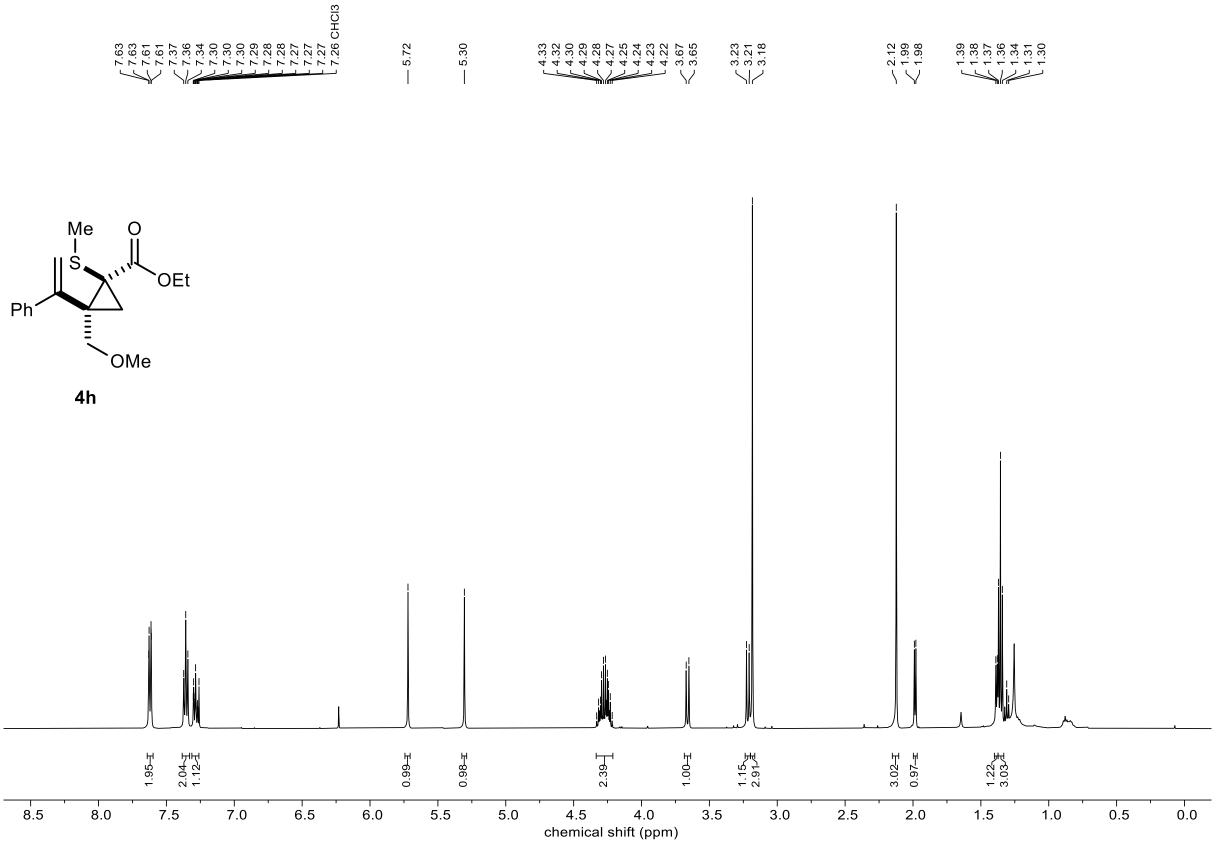

^{13}C NMR (126 MHz, $CDCl_3$)

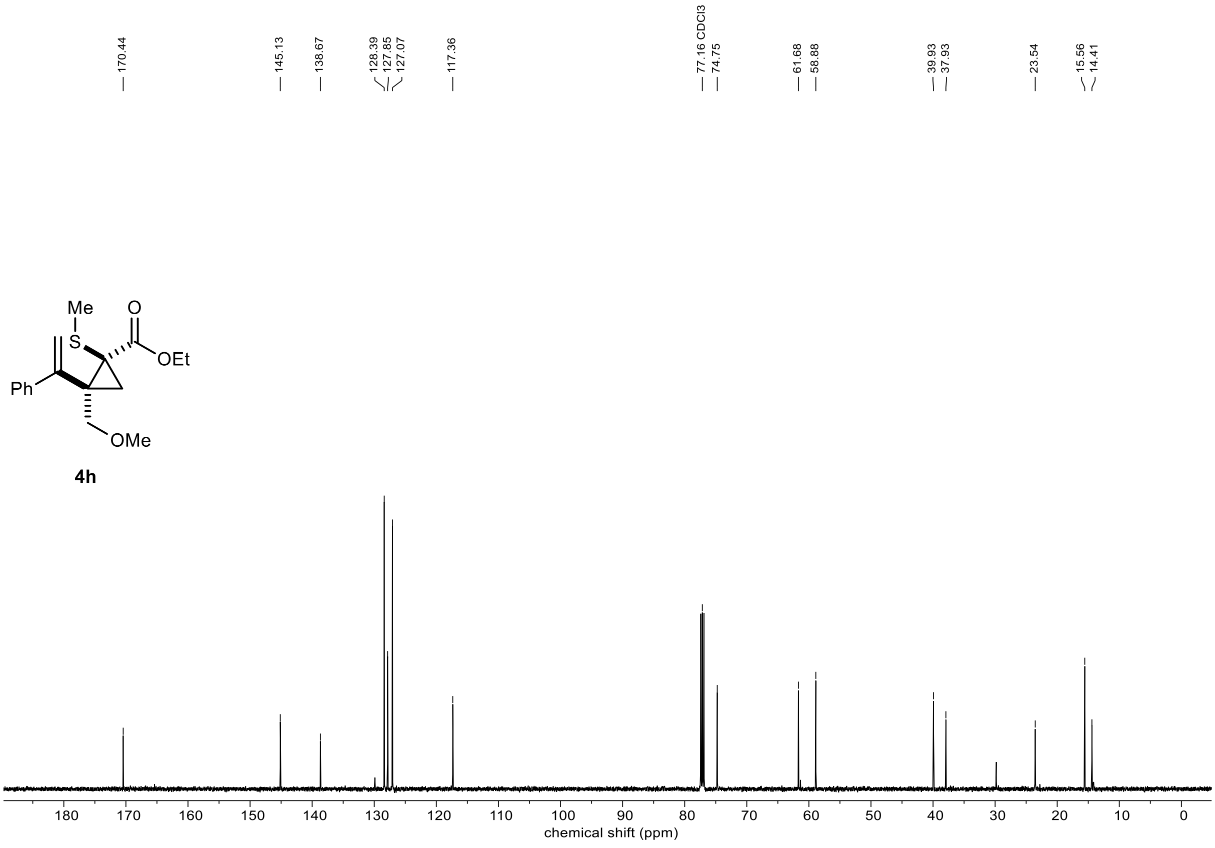

Ethyl 7-(methylthio)-2-oxo-1-vinylbicyclo[4.1.0]heptane-7-carboxylate (4i)

^{1}H NMR (700 MHz, $CDCl_3$)

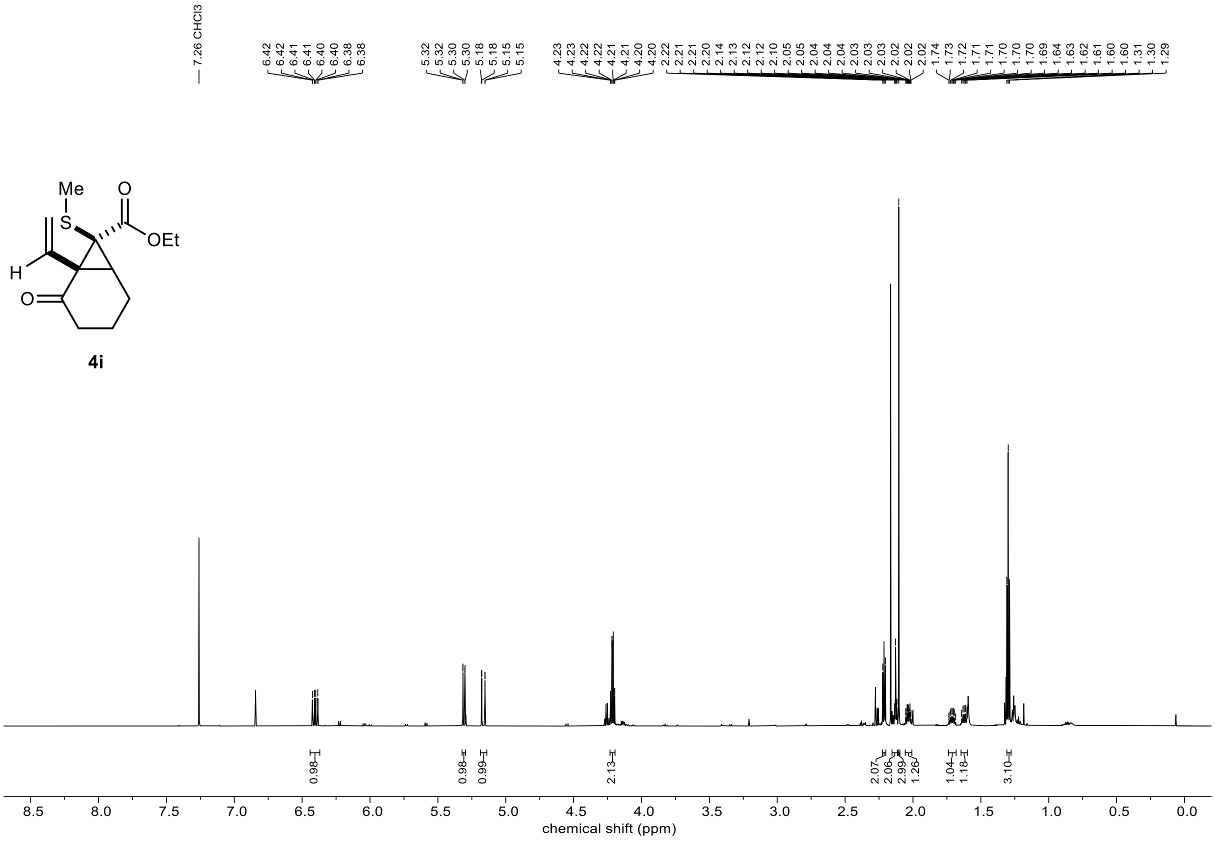

^{13}C NMR (176 MHz, $CDCl_3$)

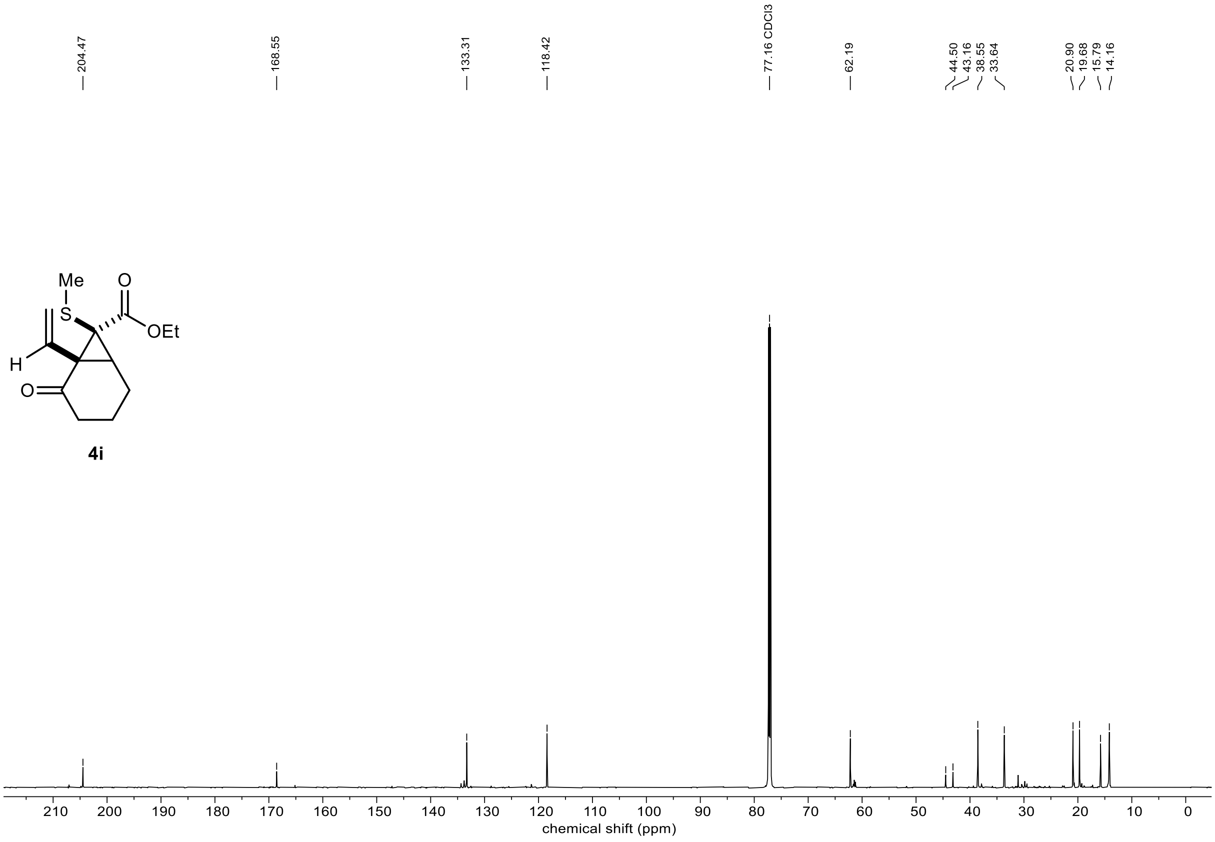

Ethyl 7-(methylthio)-2-oxo-1-(prop-1-en-2-yl)bicyclo[4.1.0]heptane-7-carboxylate (4j)

^{1}H NMR (500 MHz, $CDCl_3$)

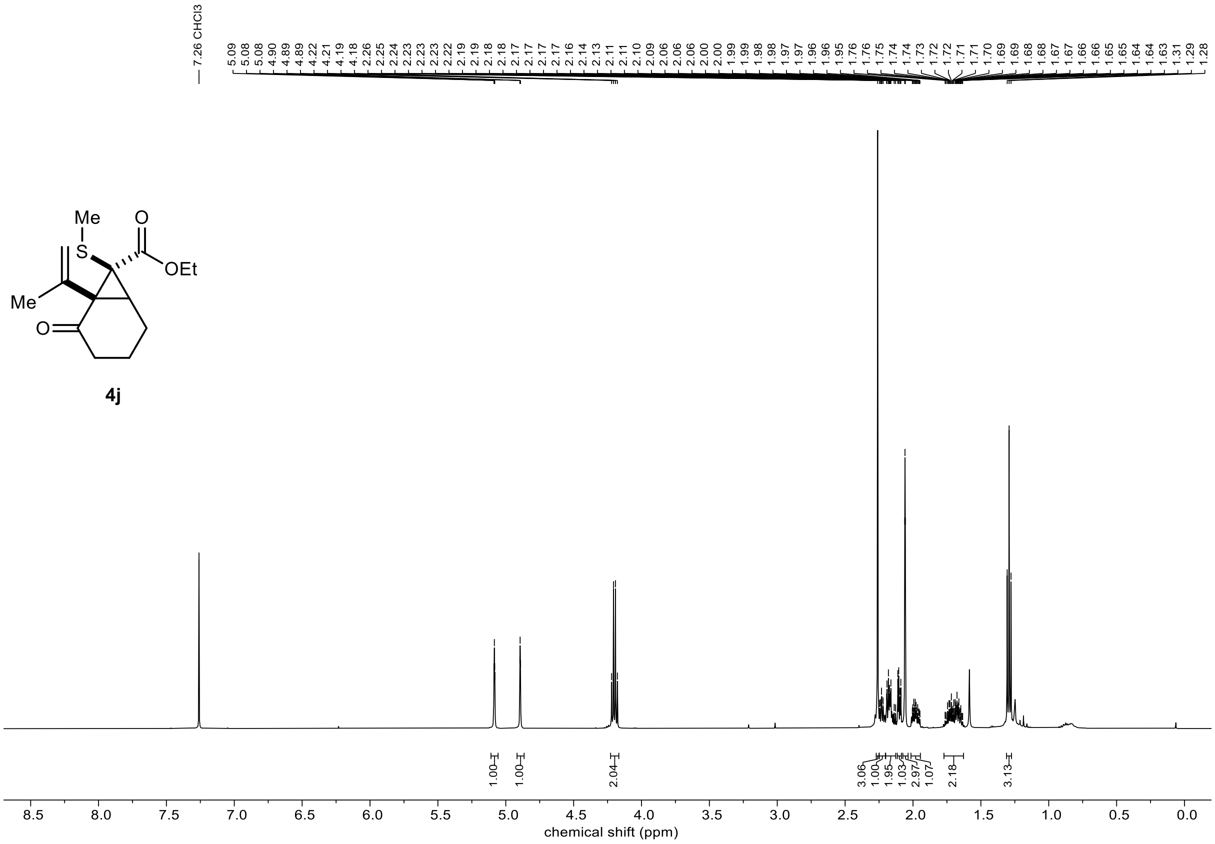

4j

^{13}C NMR (126 MHz, $CDCl_3$)

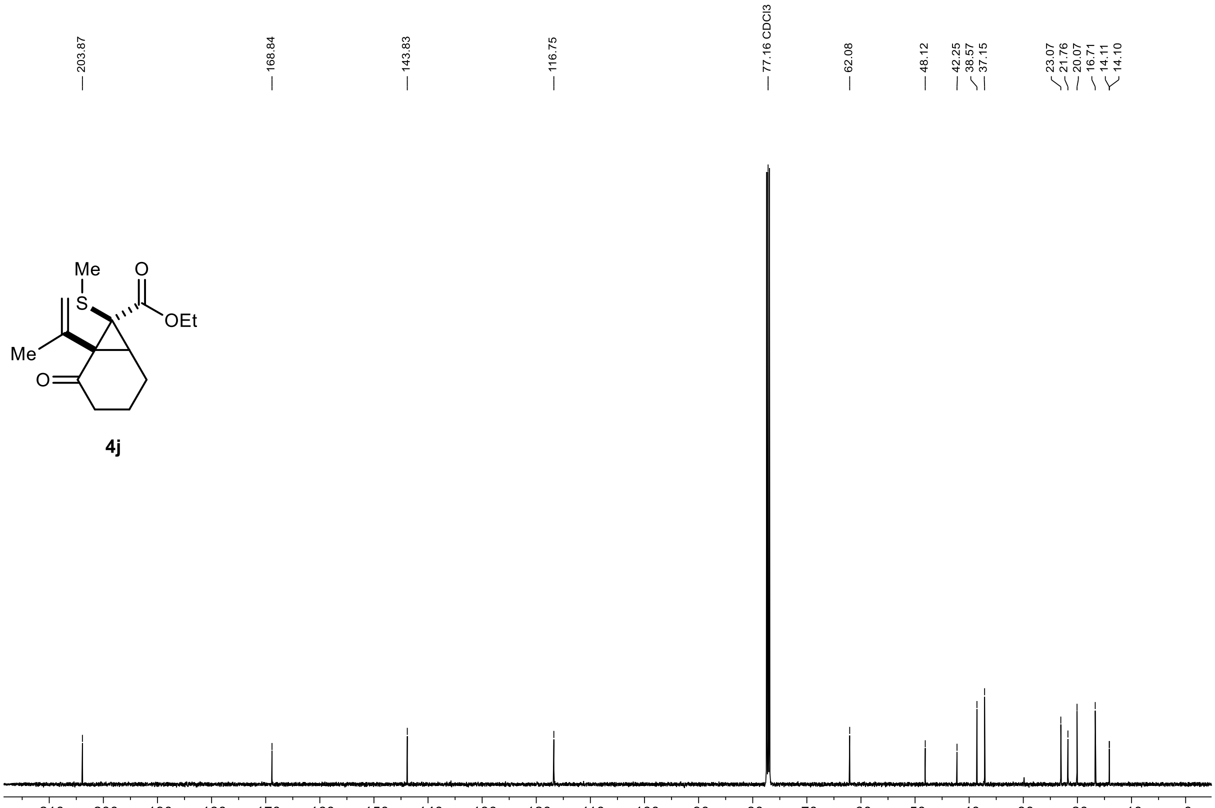

4j

1-(*Tert*-butyl) 2-methyl 1-(methylthio)-2-(1-phenylvinyl)cyclopropane-1,2-dicarboxylate (5a)

^{1}H NMR (500 MHz, $CDCl_3$)

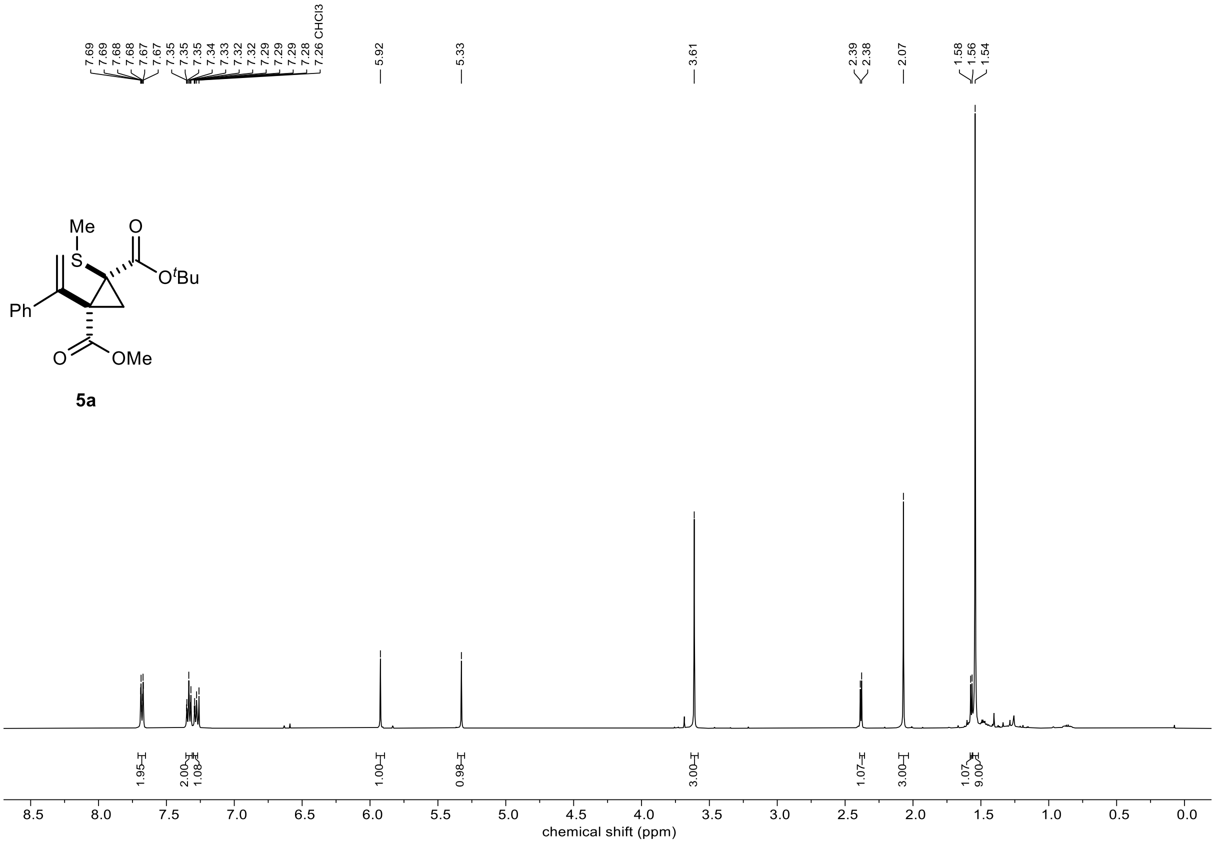

^{13}C NMR (126 MHz, $CDCl_3$)

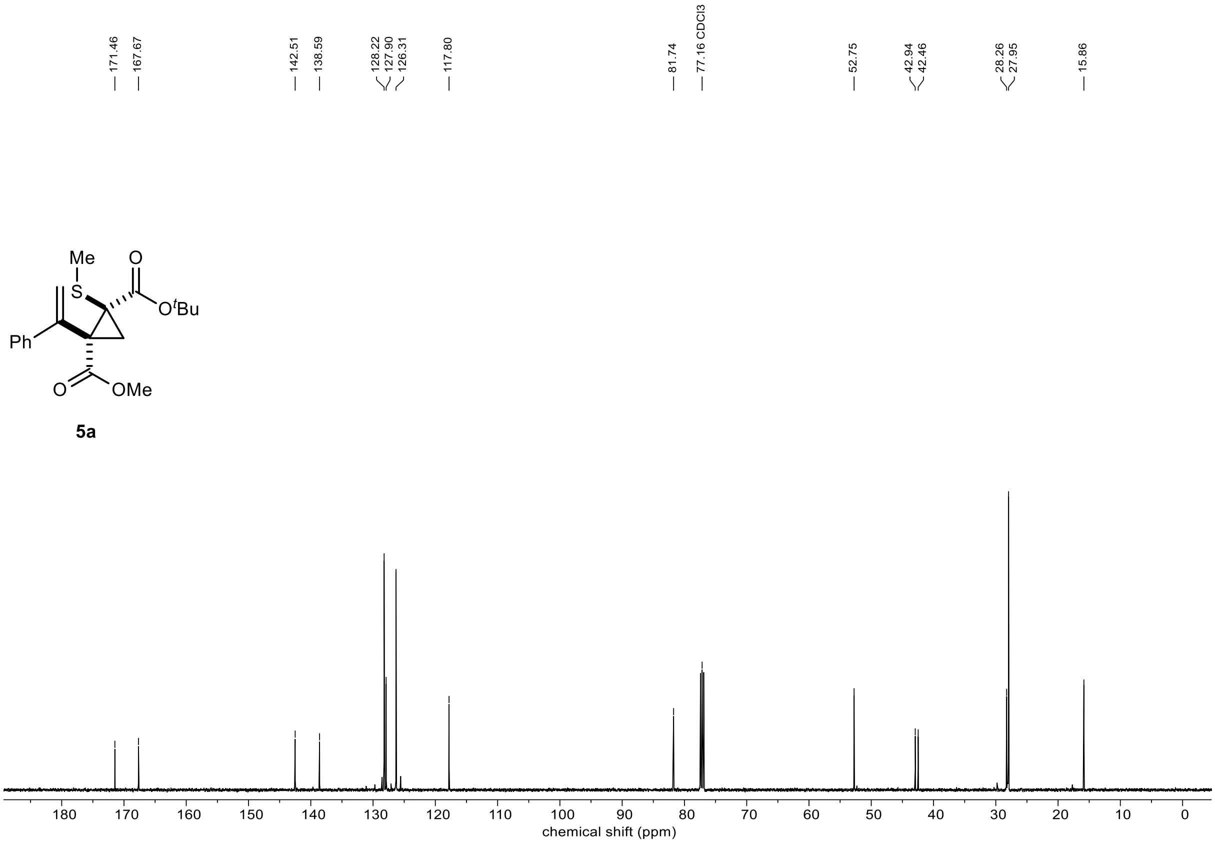

1-Benzyl 2-methyl 1-(methylthio)-2-(1-phenylvinyl)cyclopropane-1,2-dicarboxylate (5b)

^{1}H NMR (500 MHz, $CDCl_3$)

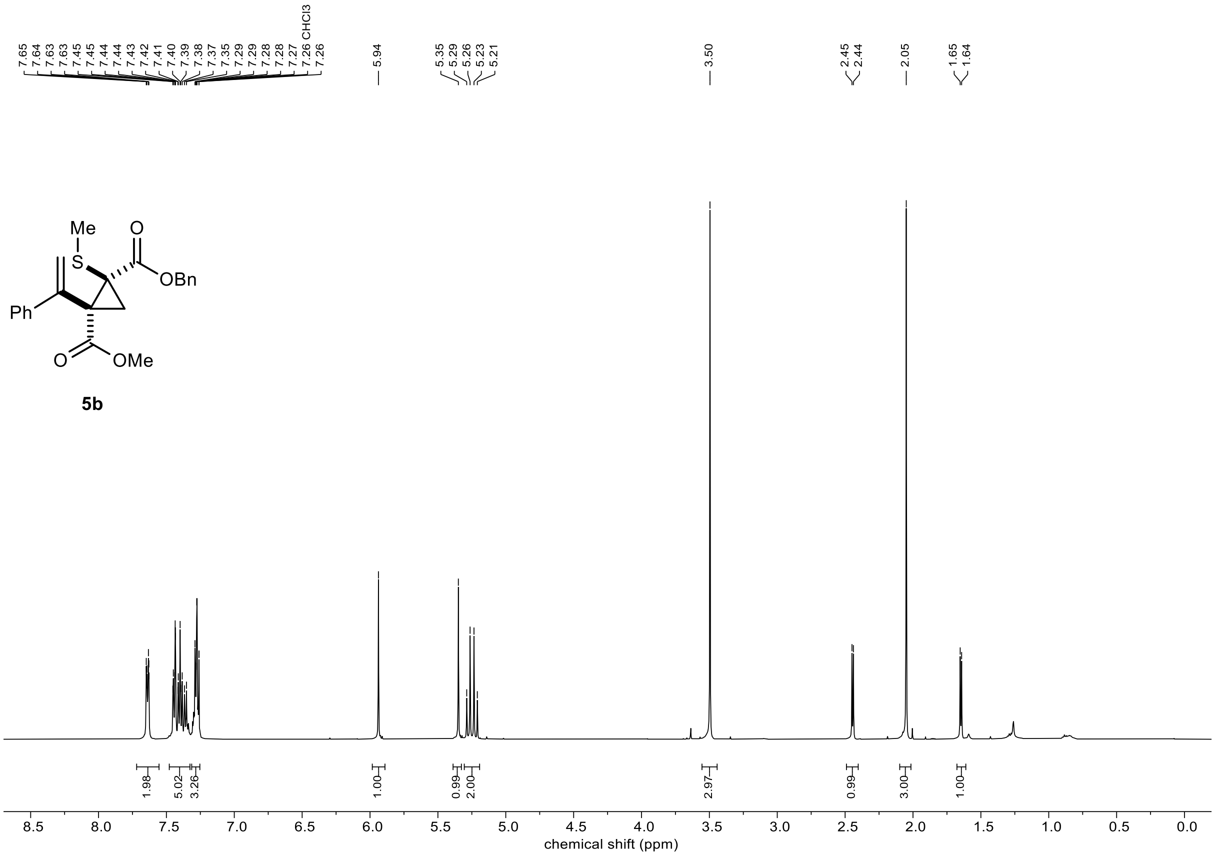

^{13}C NMR (126 MHz, $CDCl_3$)

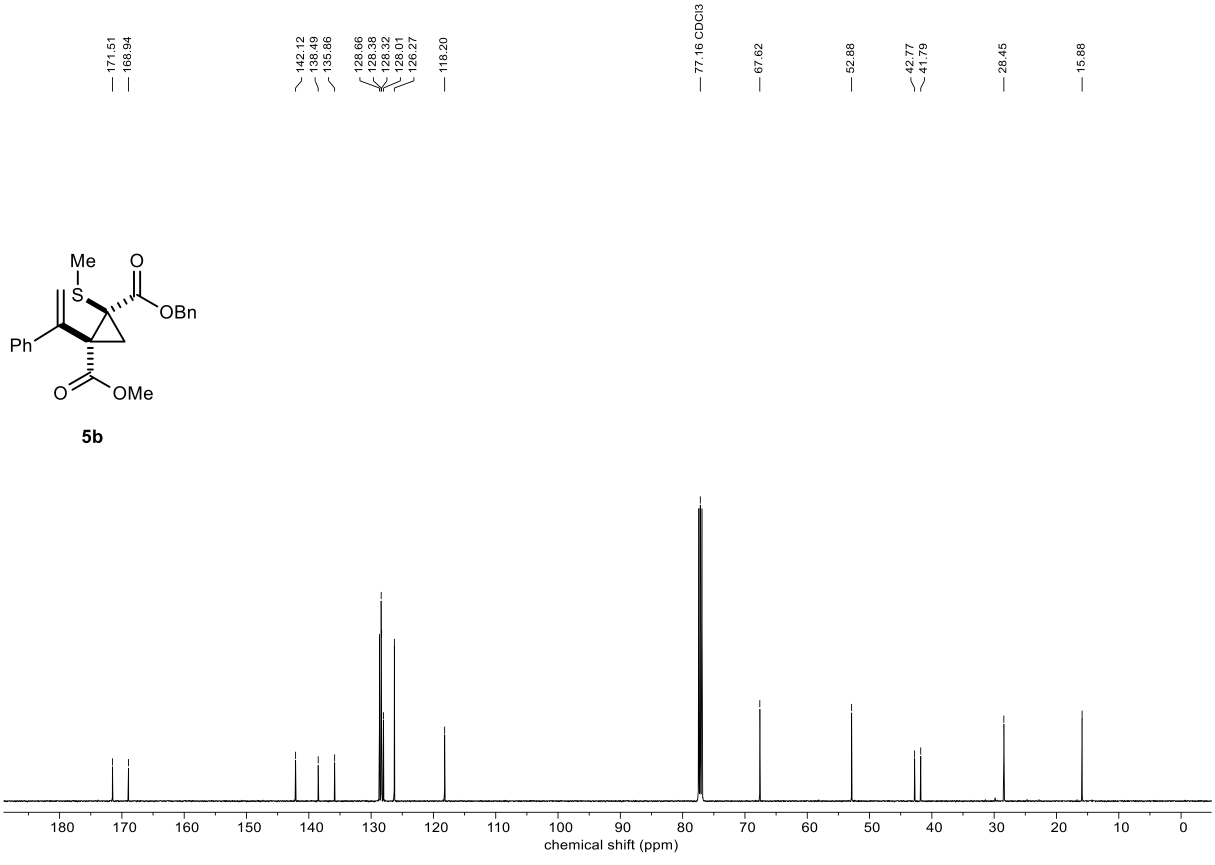

Methyl 2-acetyl-2-(methylthio)-1-(1-phenylvinyl)cyclopropane-1-carboxylate (5c)

^{1}H NMR (500 MHz, $CDCl_3$)

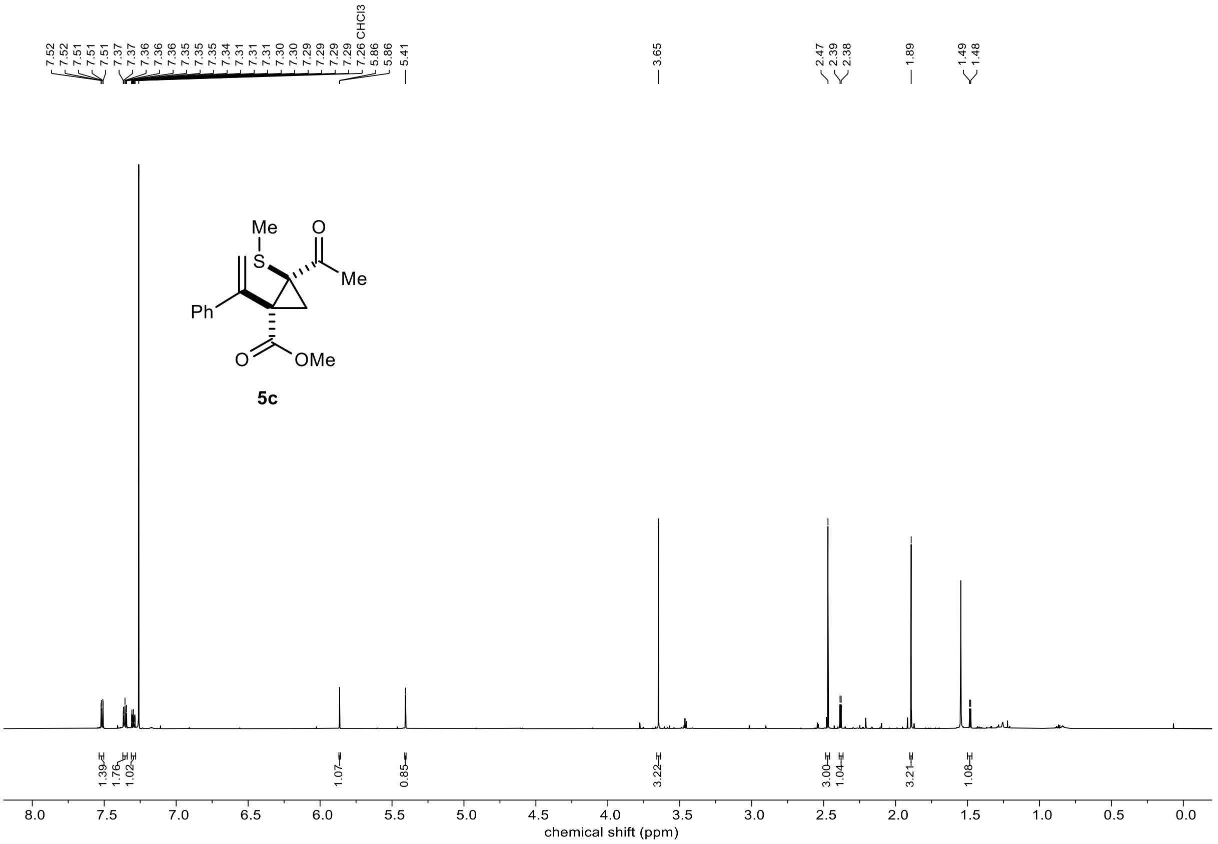

^{13}C NMR (126 MHz, $CDCl_3$)

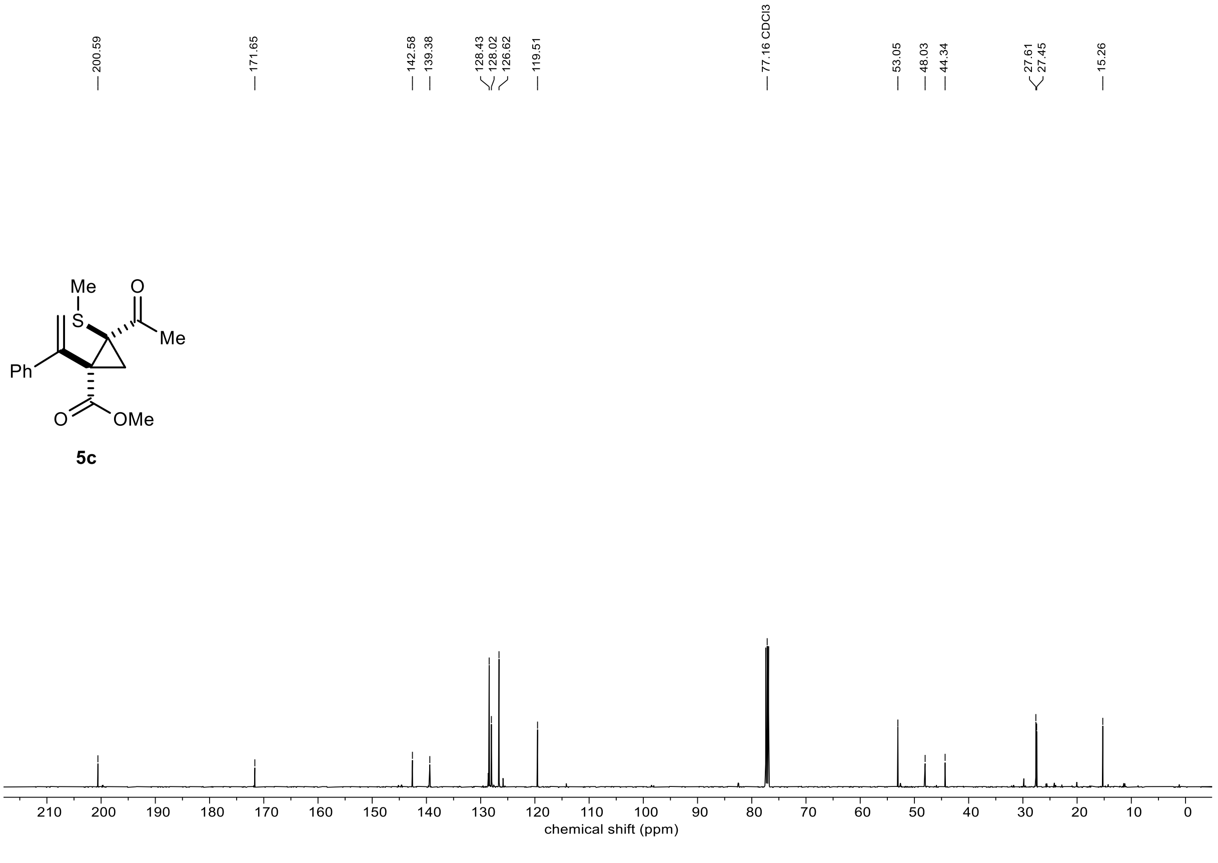

Methyl 2-benzoyl-2-(methylthio)-1-(1-phenylvinyl)cyclopropane-1-carboxylate (5d)

^{1}H NMR (500 MHz, $CDCl_3$)

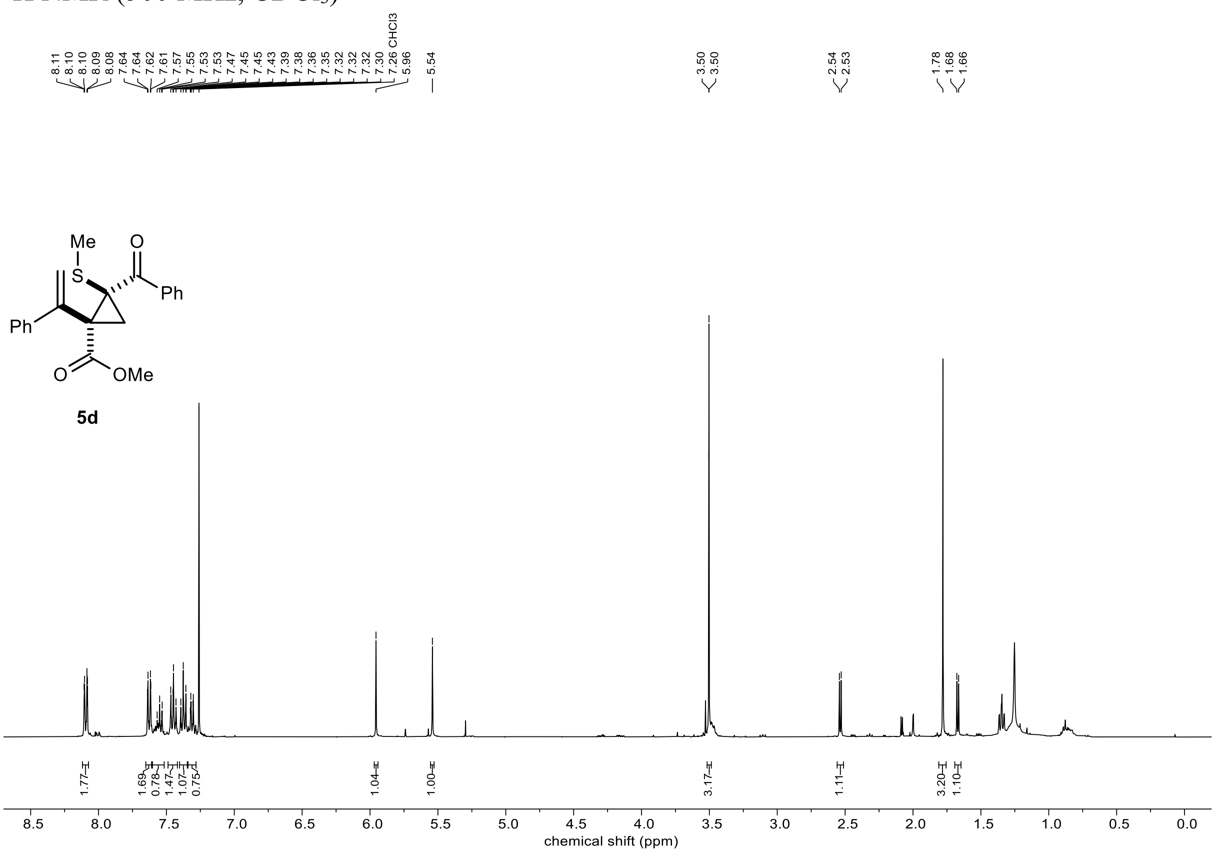

^{13}C NMR (126 MHz, $CDCl_3$)

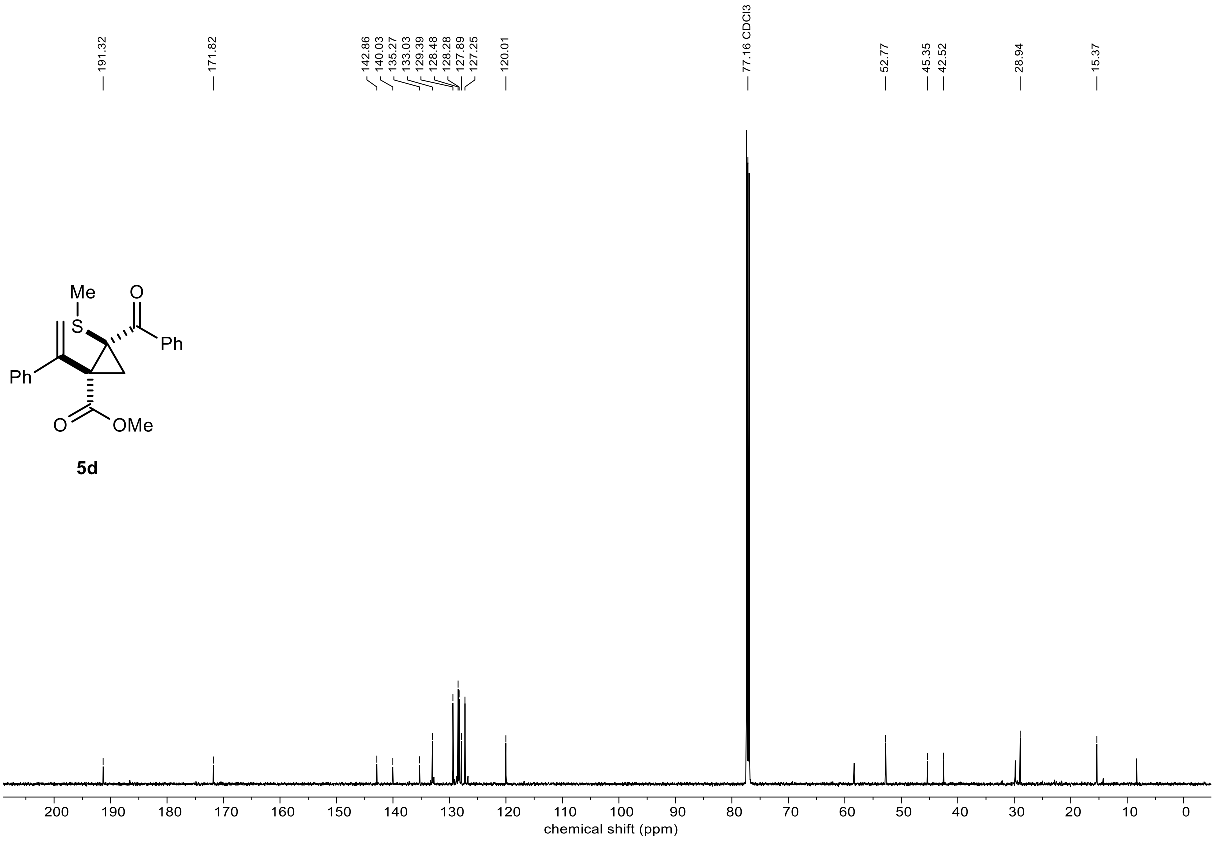

Methyl 2-(methylthio)-1-(1-phenylvinyl)cyclopropane-1-carboxylate (5e)

^{1}H NMR (500 MHz, $CDCl_3$)

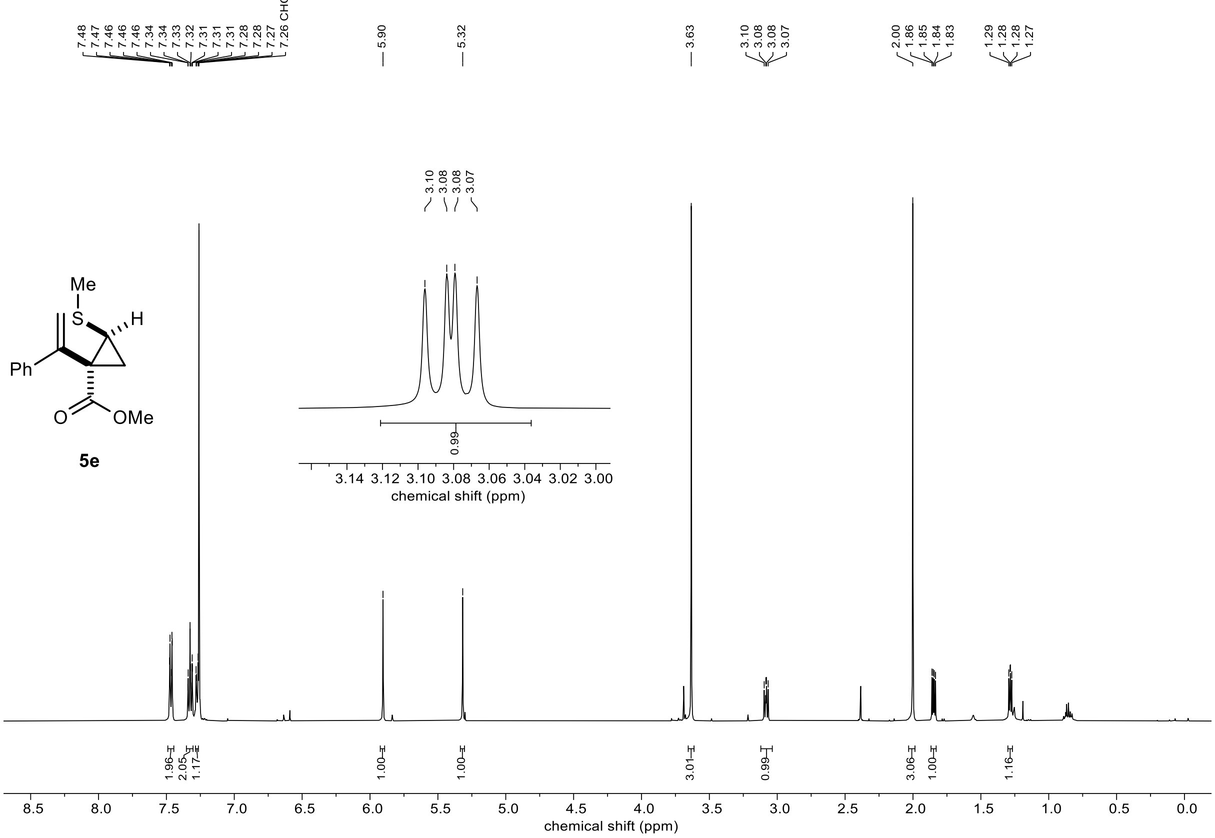

^{13}C NMR (126 MHz, $CDCl_3$)

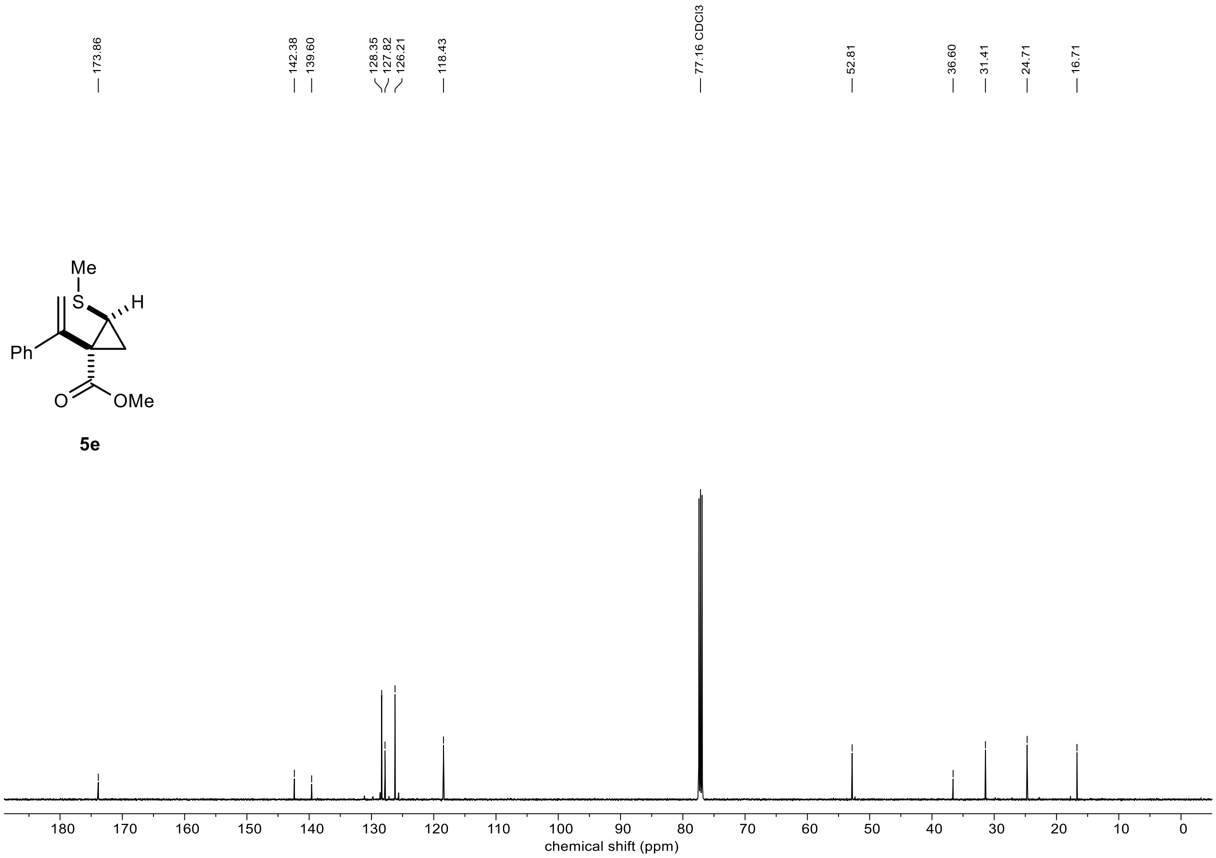

1-Ethyl 2-methyl 1-(ethylthio)-2-(1-phenylvinyl)cyclopropane-1,2-dicarboxylate (6a)

^{1}H NMR (500 MHz, $CDCl_3$)

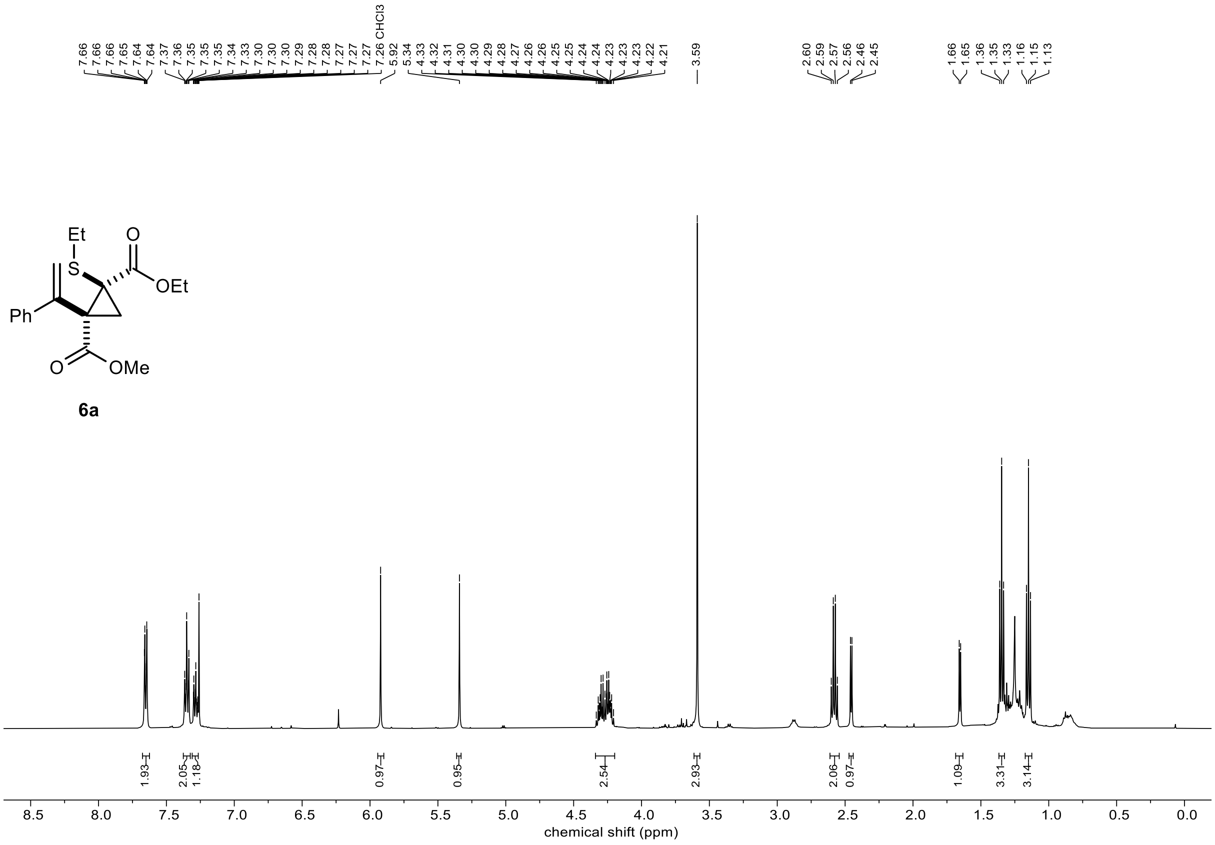

^{13}C NMR (126 MHz, $CDCl_3$)

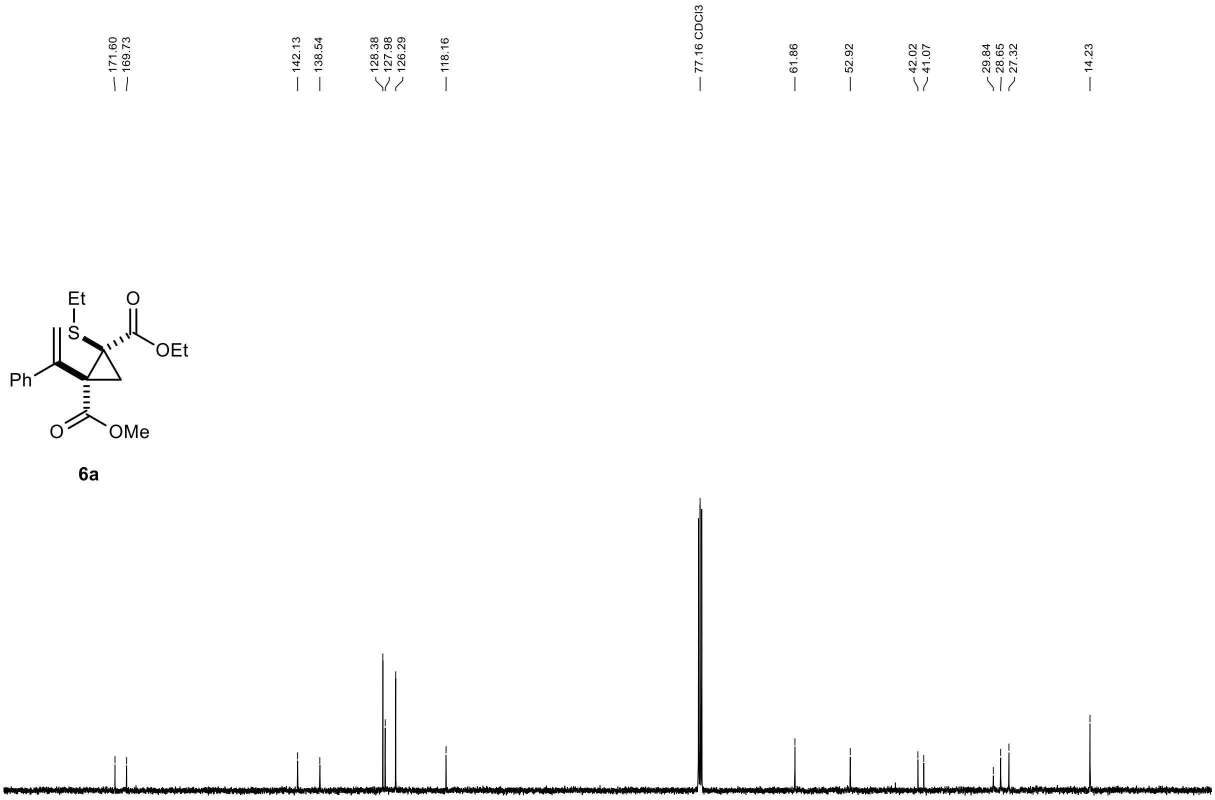

1-Ethyl 2-methyl 1-((2-ethoxy-2-oxoethyl)thio)-2-(1-phenylvinyl)cyclopropane-1,2-dicarboxylate (6b)

^{1}H NMR (500 MHz, $CDCl_3$)

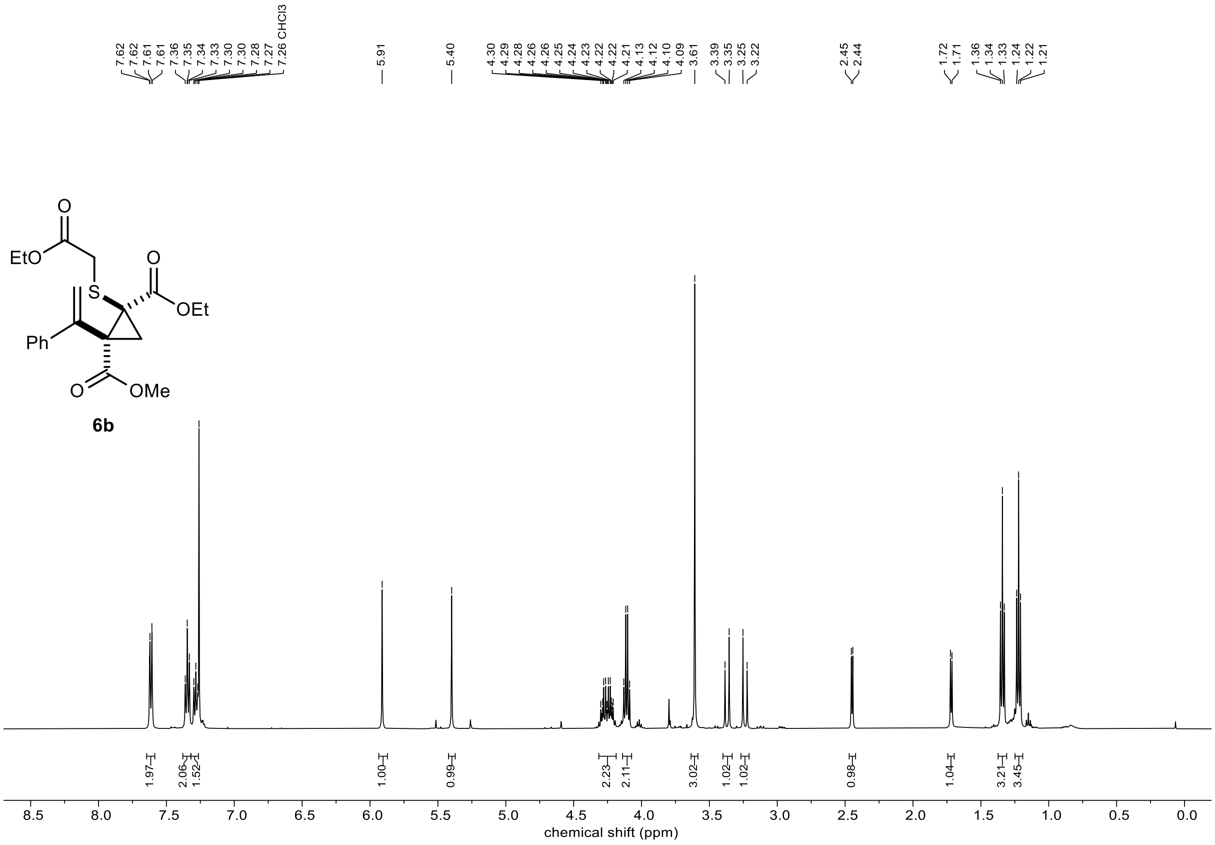

^{13}C NMR (126 MHz, $CDCl_3$)

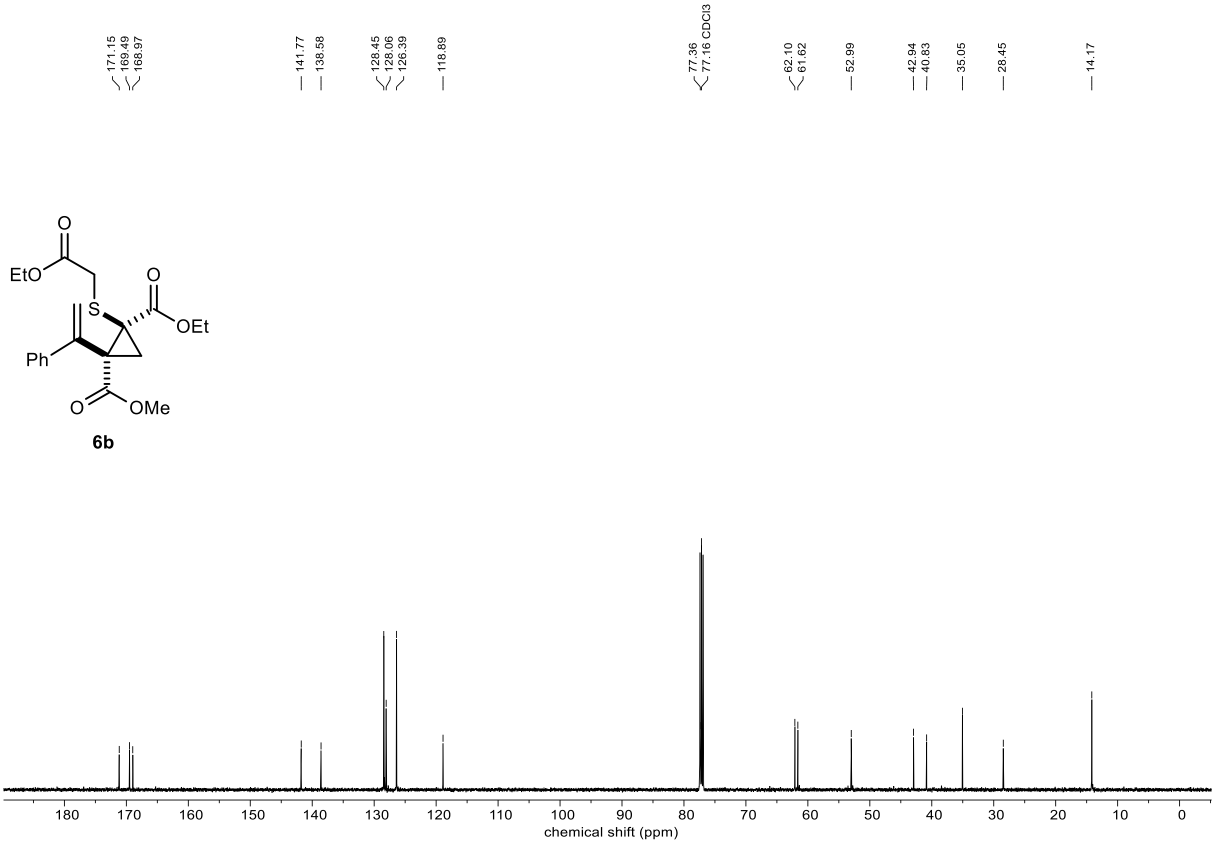

1-Ethyl 2-methyl 1-(benzylthio)-2-(1-phenylvinyl)cyclopropane-1,2-dicarboxylate (6c)

^{1}H NMR (500 MHz, $CDCl_3$)

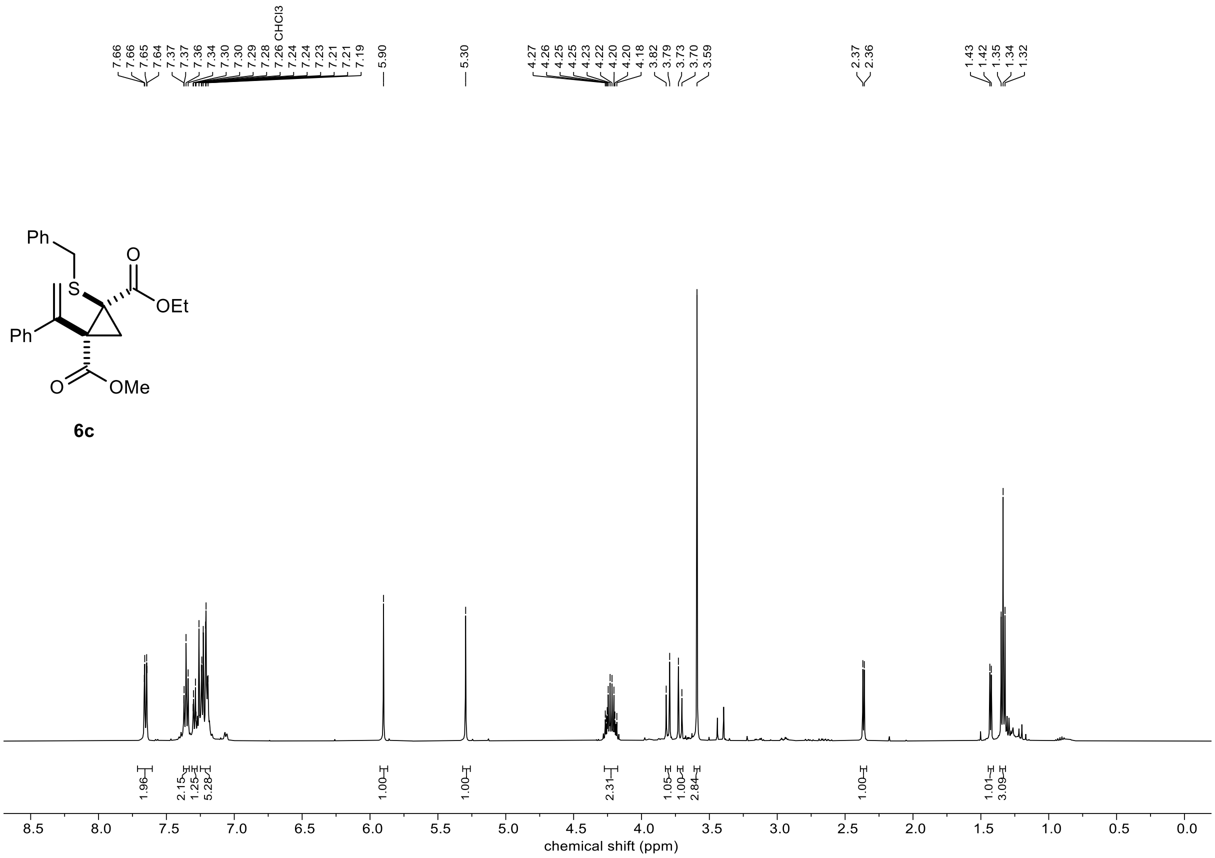

^{13}C NMR (126 MHz, $CDCl_3$)

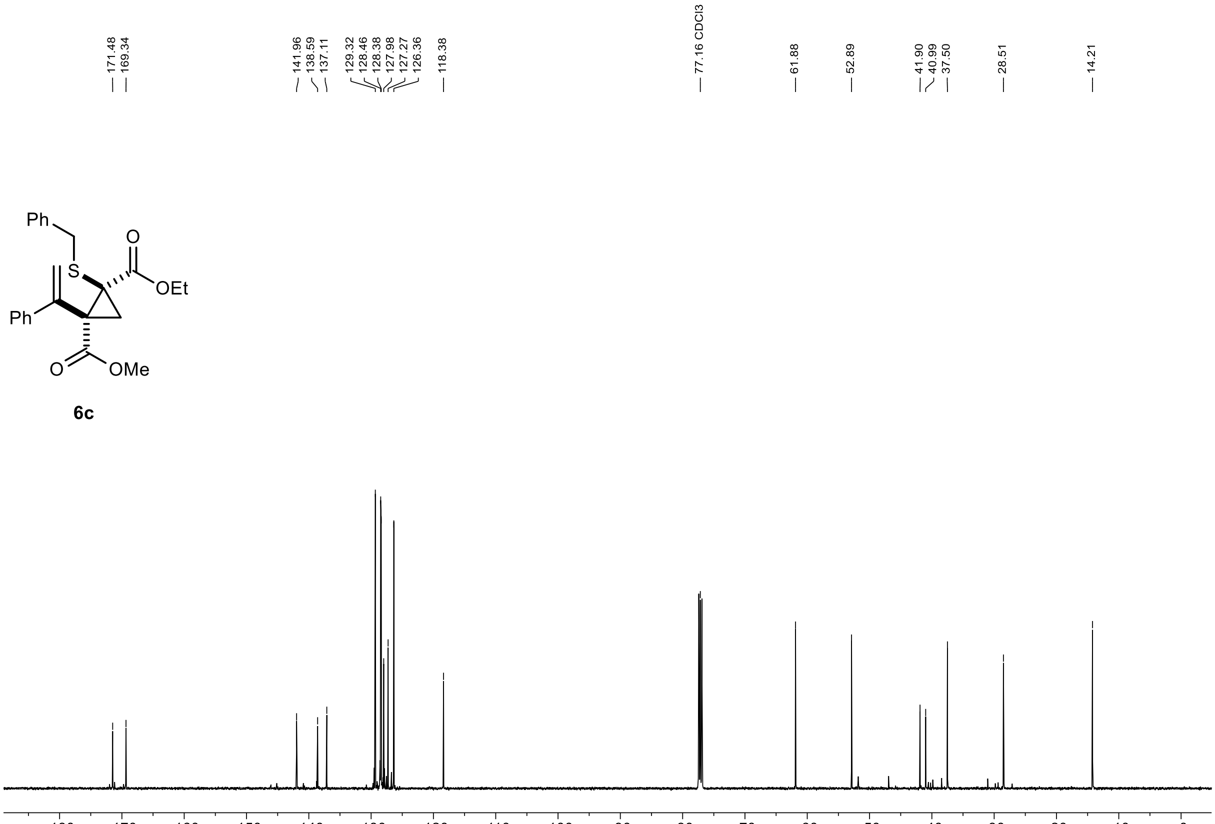

1-Ethyl 2-methyl (phenylthio)-2-(1-phenylvinyl)cyclopropane-1,2-dicarboxylate (6d)

^{1}H NMR (500 MHz, $CDCl_3$)

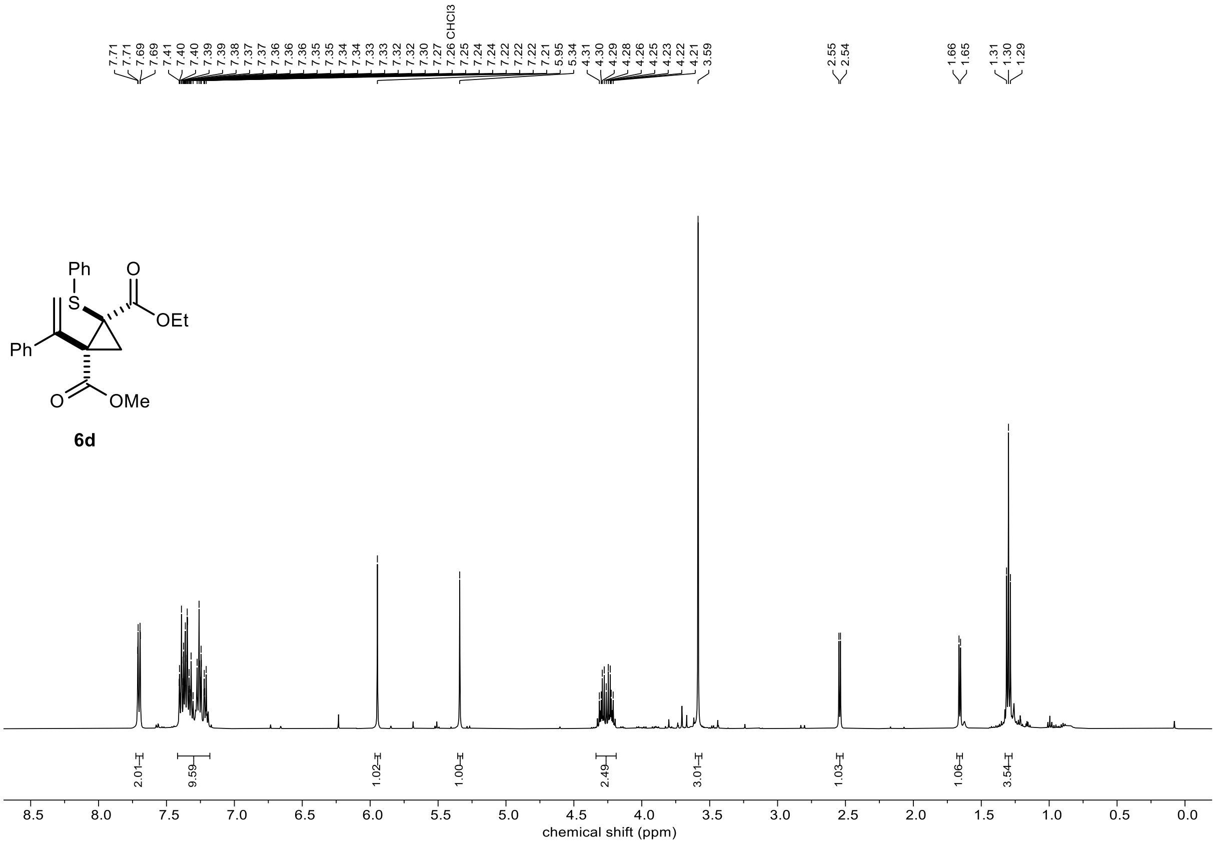

^{13}C NMR (126 MHz, $CDCl_3$)

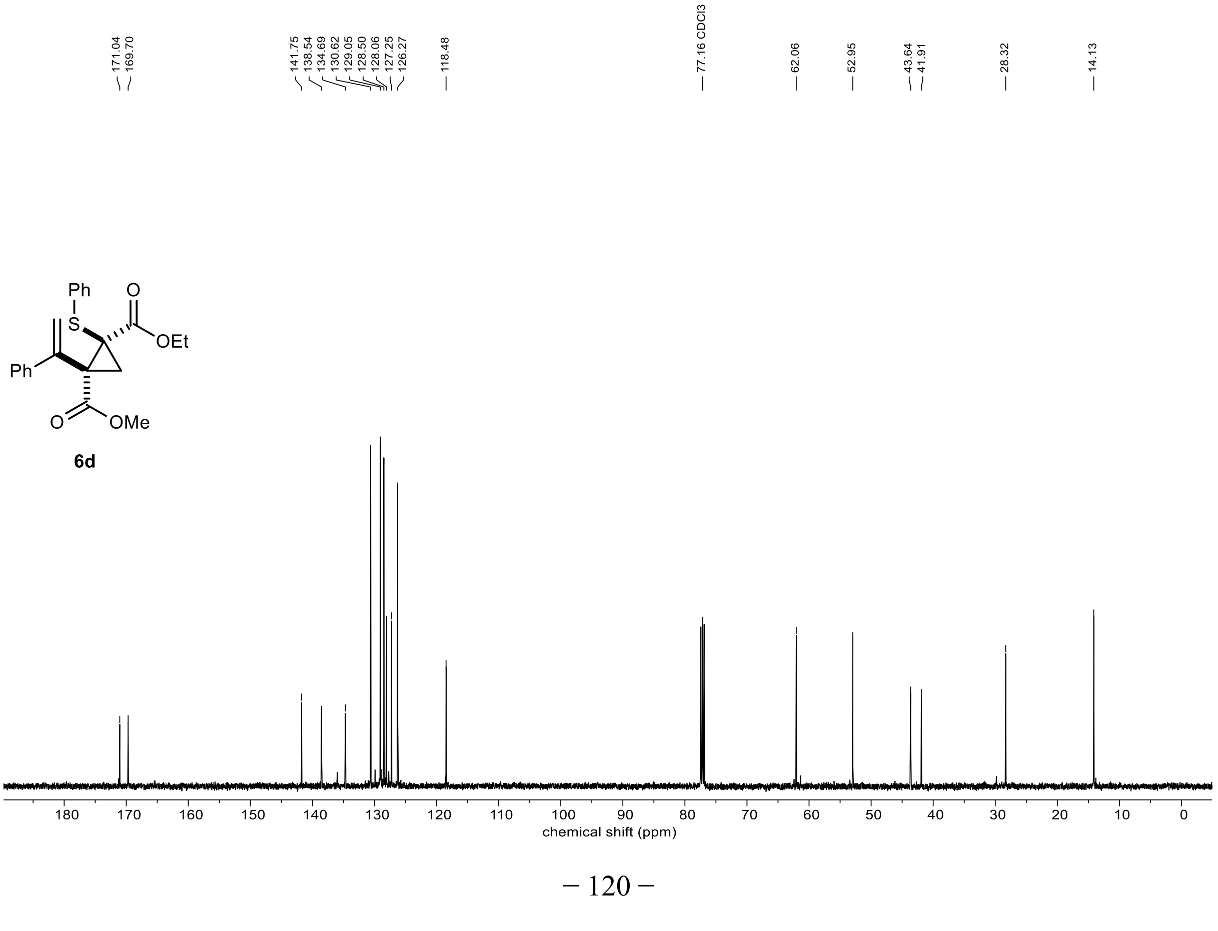

Ethyl (Z)-2-((3-(methoxycarbonyl)-2-phenylbuta-1,3-dien-1-yl)thio)pent-4-enoate (7)

^{1}H NMR (500 MHz, $CDCl_3$)

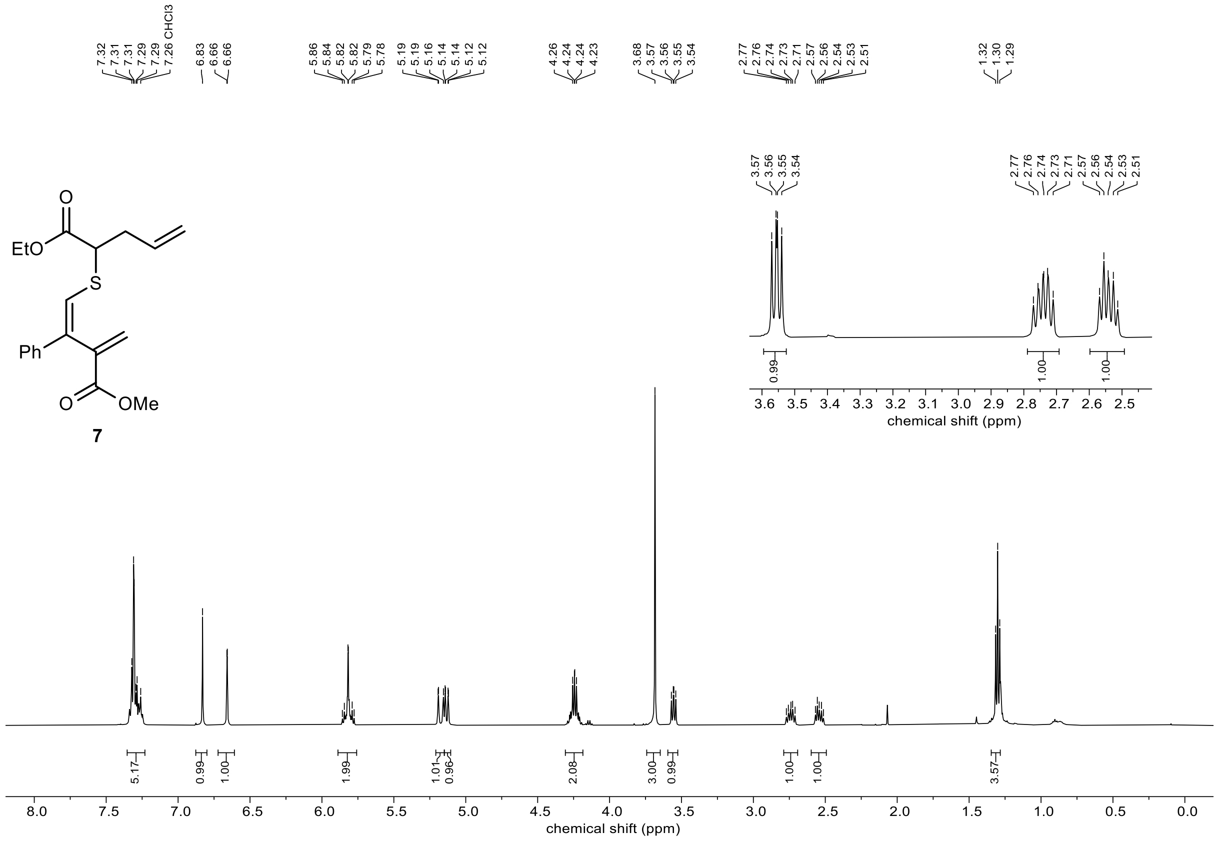

^{13}C NMR (126 MHz, $CDCl_3$)

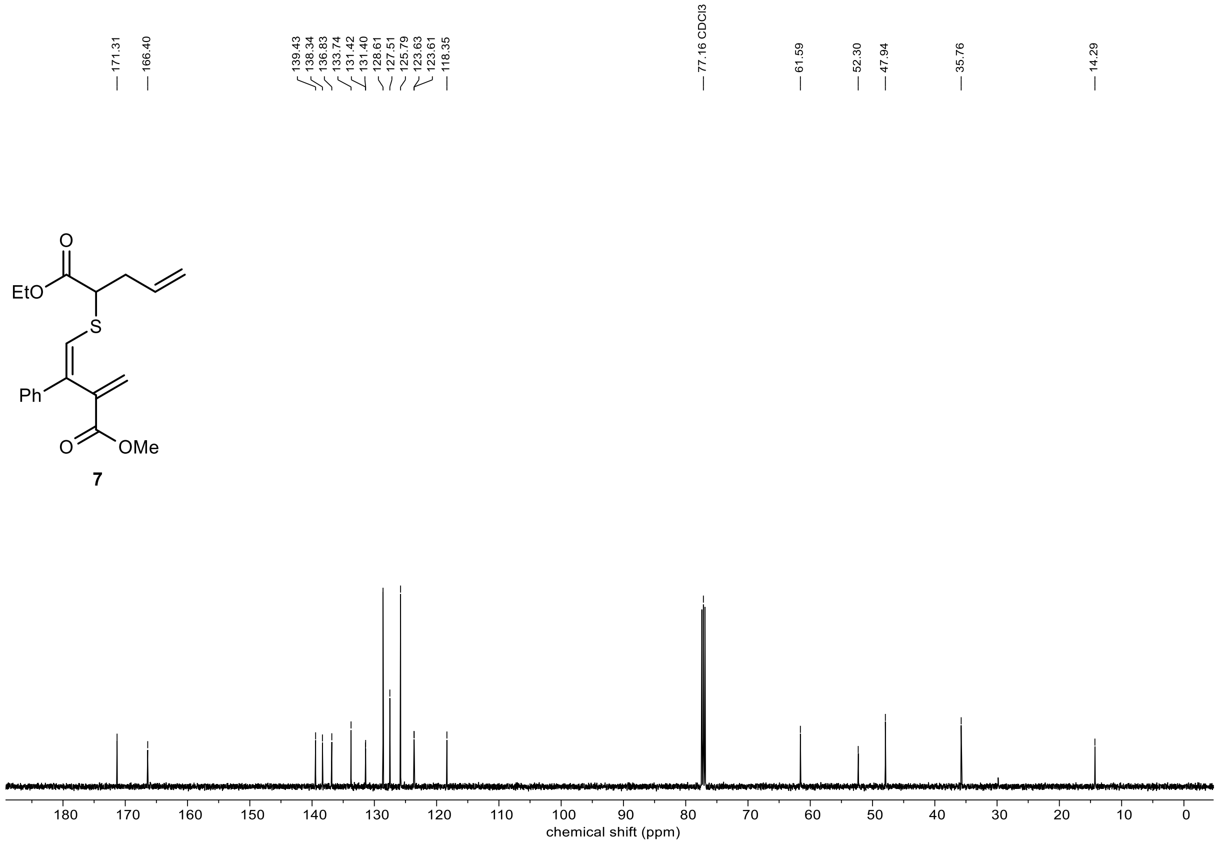

(*Z*)-(2-Ethoxy-2-oxoethyl)(3-(methoxycarbonyl)-2-phenylbuta-1,3-dien-1-yl)(methyl)sulfonium triflate salt (8)

^{1}H NMR (500 MHz, $CDCl_3$)

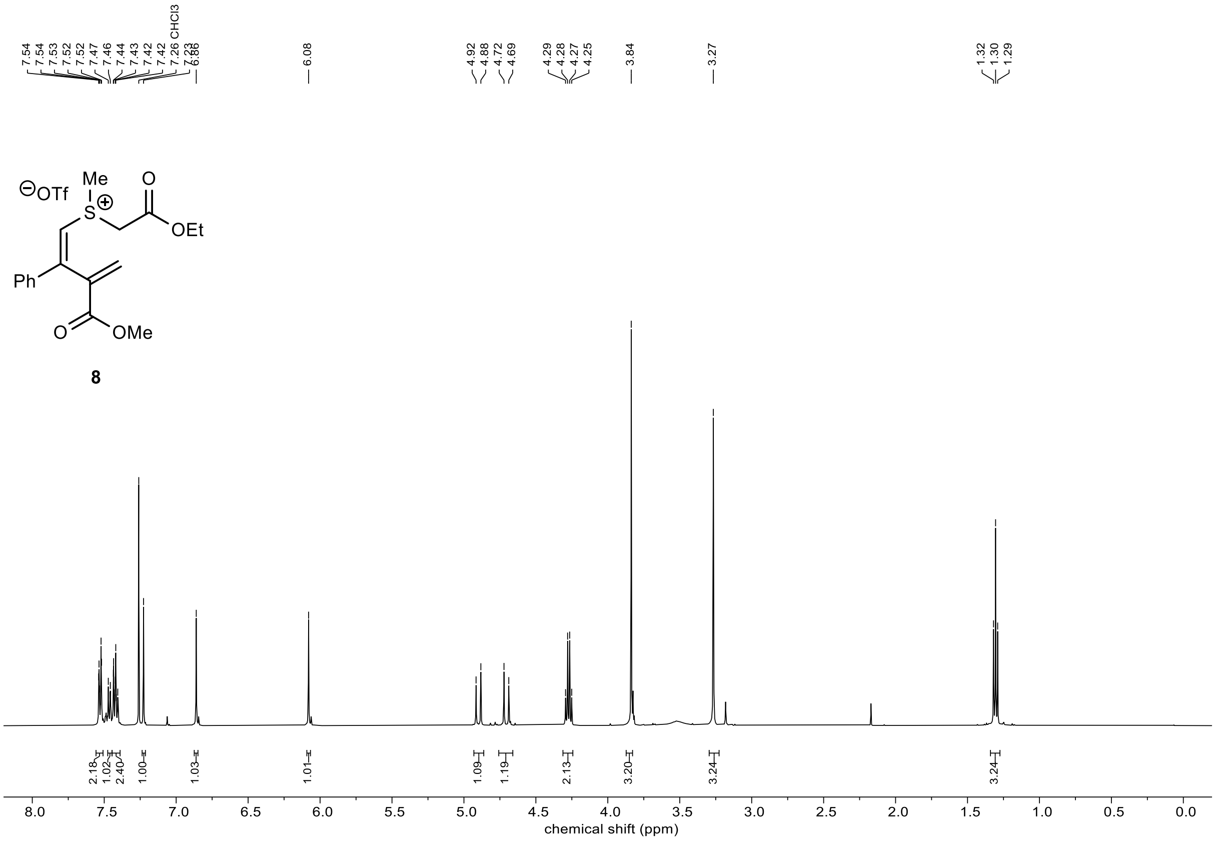

^{13}C NMR (126 MHz, $CDCl_3$)

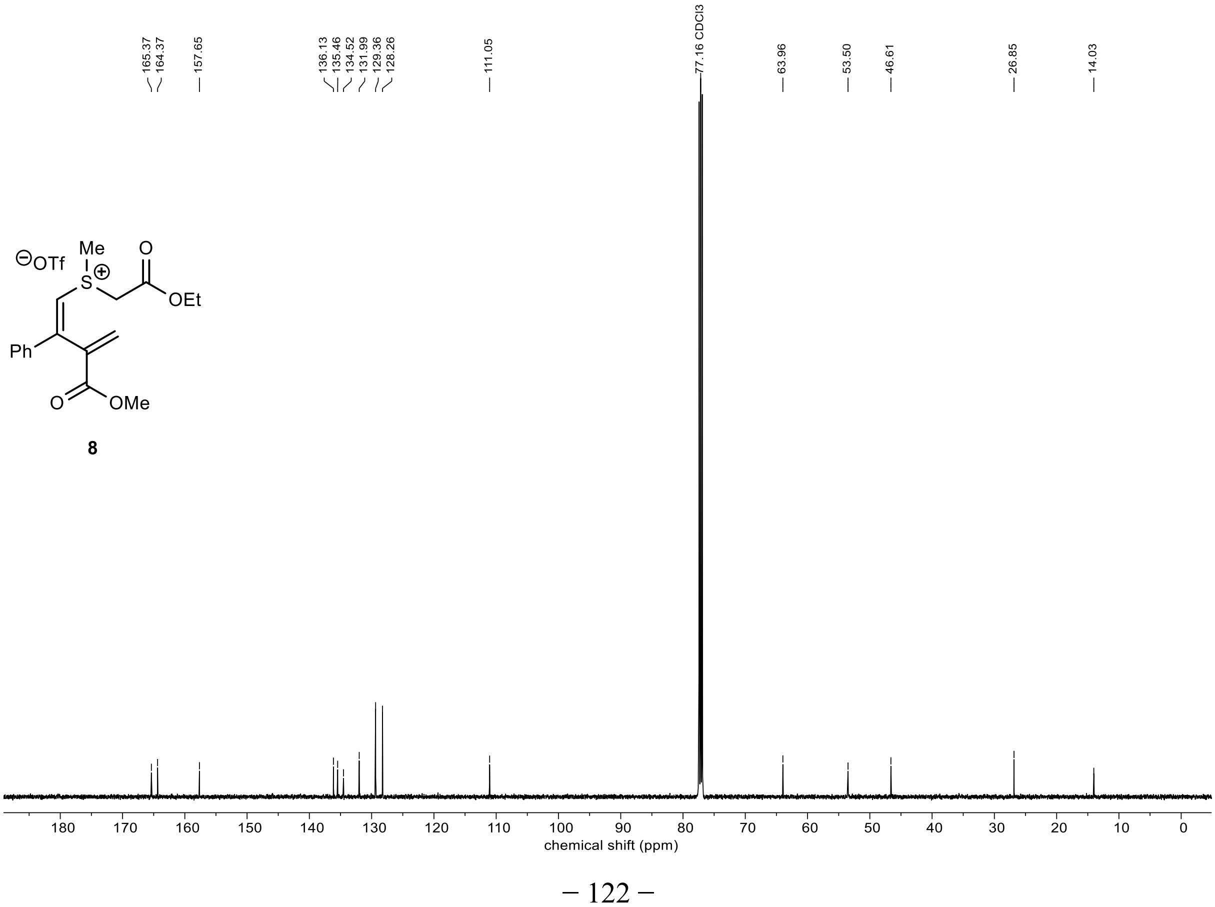

^{19}F NMR (376 MHz, $CDCl_3$)

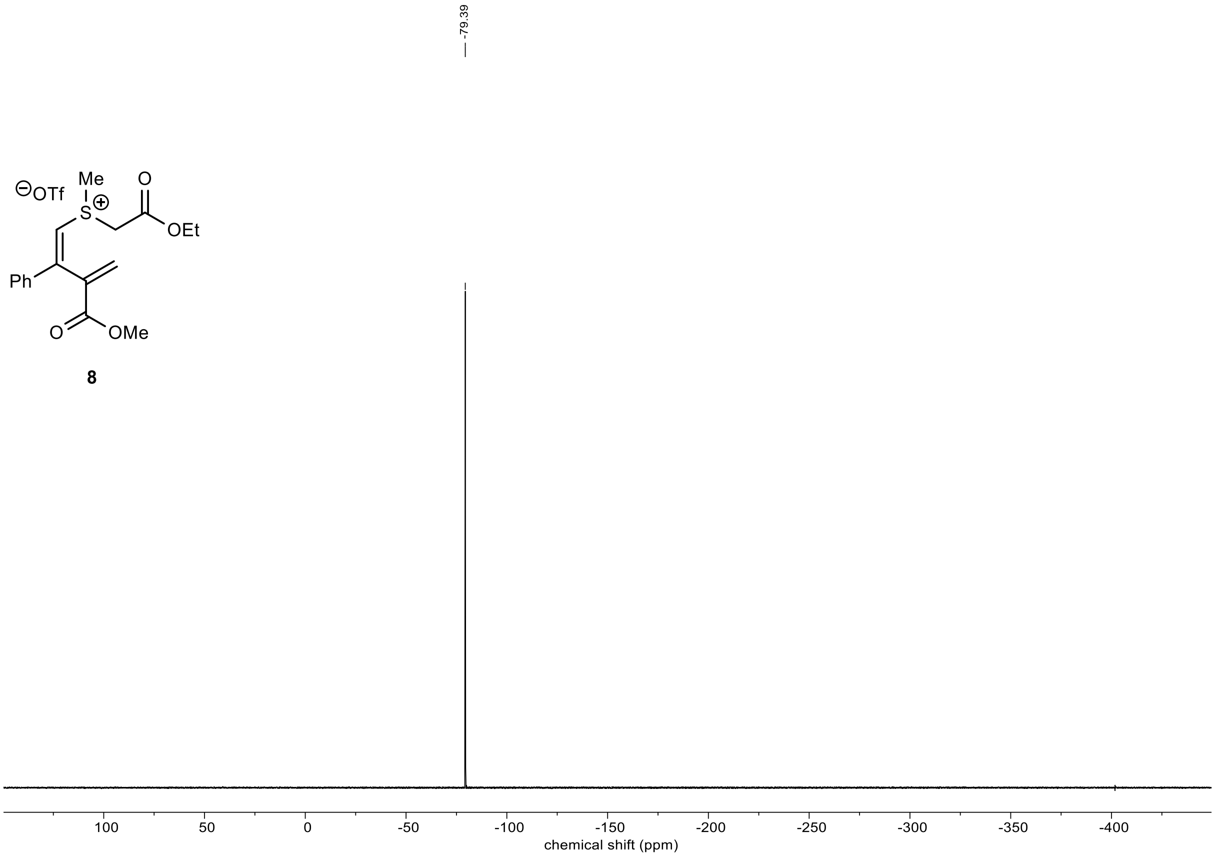

Methyl benzoyl-5-phenyl-3,4-dihydro-2H-thiopyran-4-carboxylate (10c)

^{1}H NMR (500 MHz, $CDCl_3$)

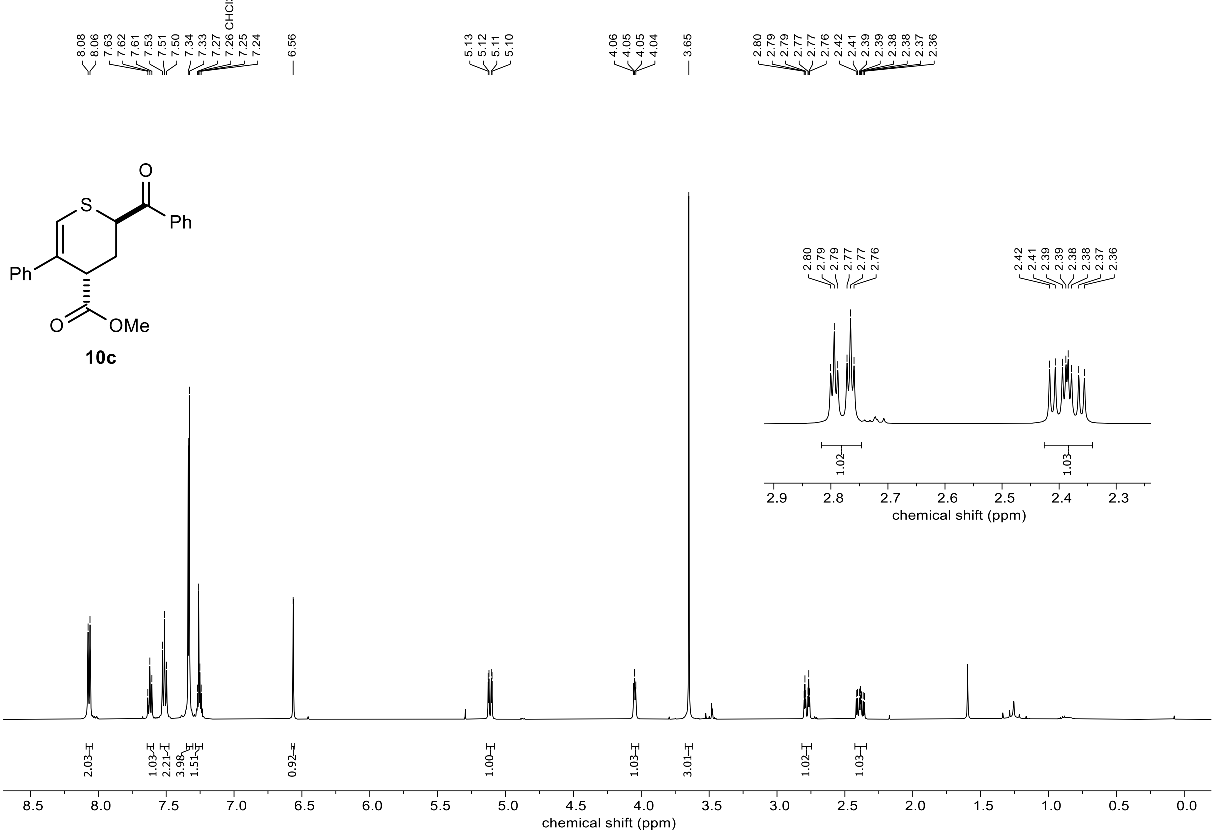

^{13}C NMR (126 MHz, $CDCl_3$)

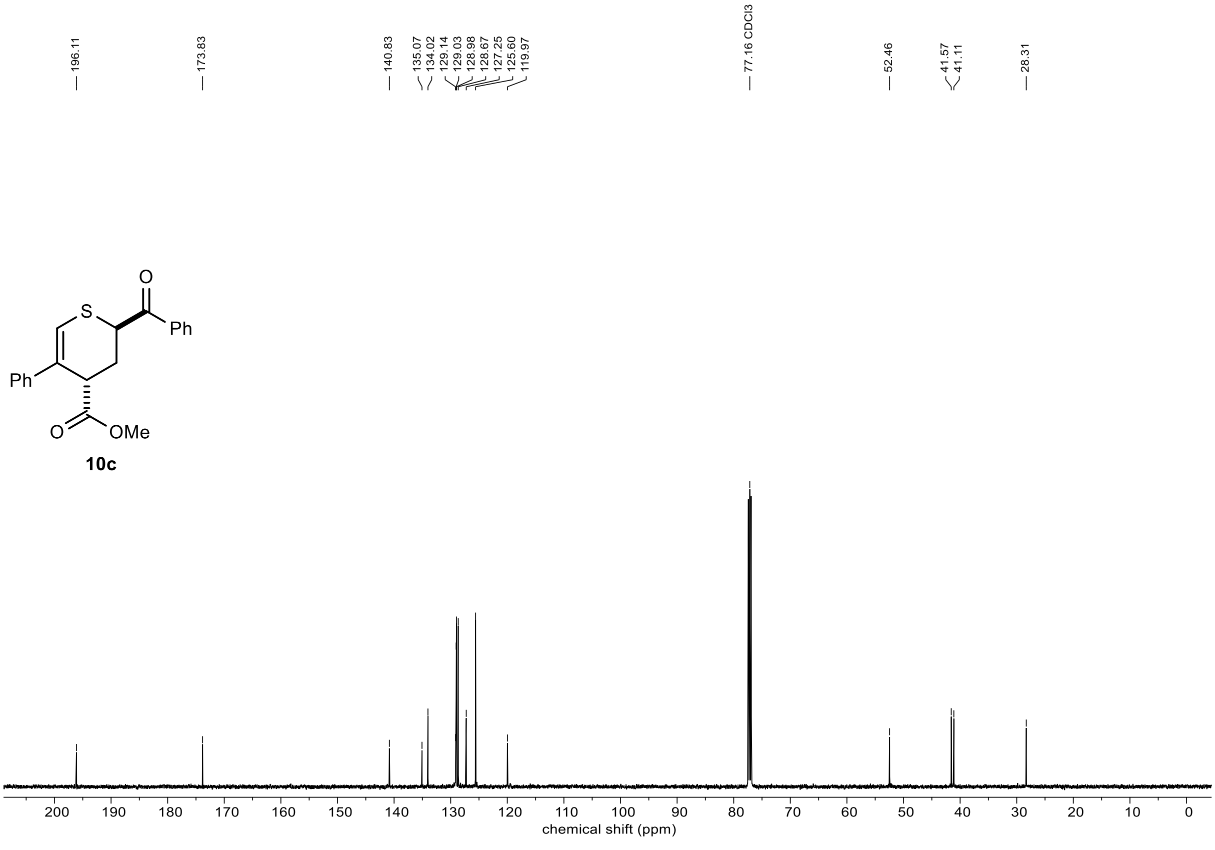

Methyl 4-phenyl-2,5-dihydrothiophene-3-carboxylate 1,1-dioxide (S-15a)

^{1}H NMR (500 MHz, $CDCl_3$)

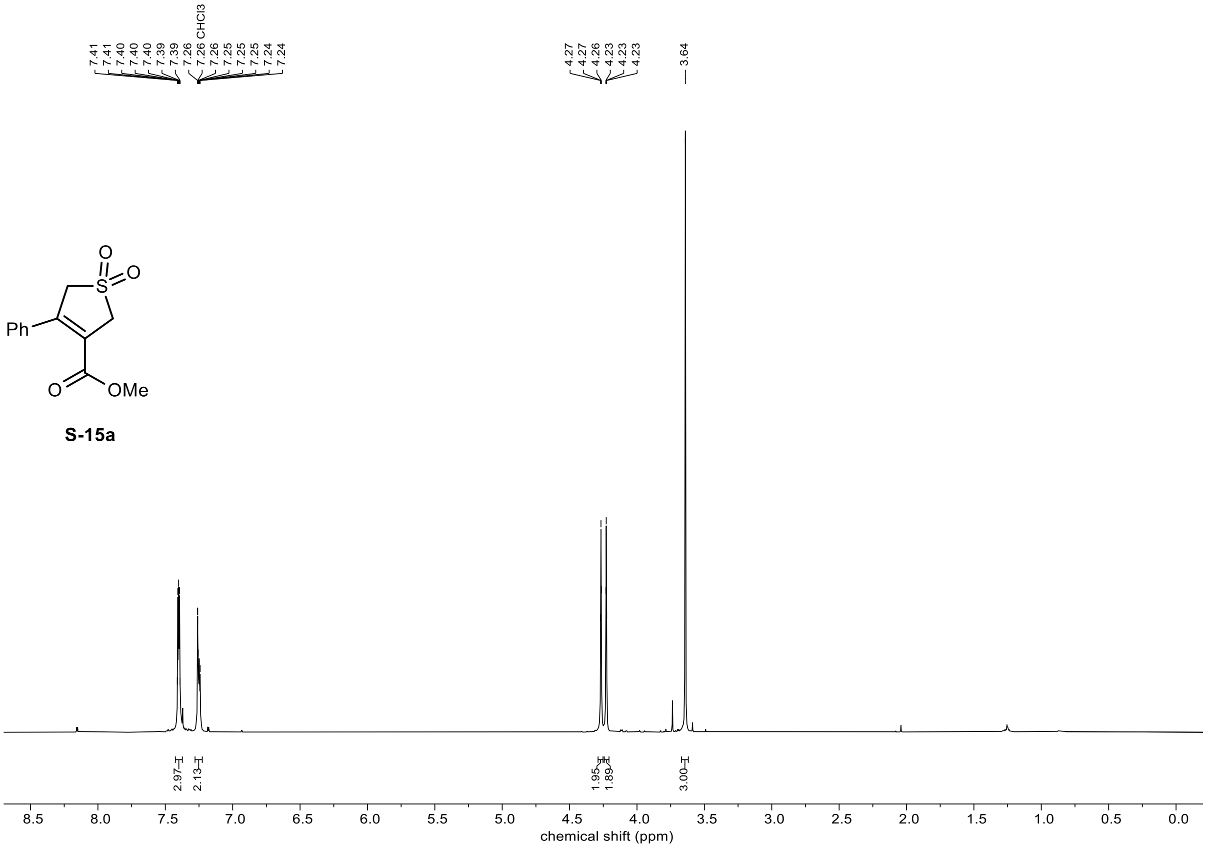

^{13}C NMR (126 MHz, $CDCl_3$)

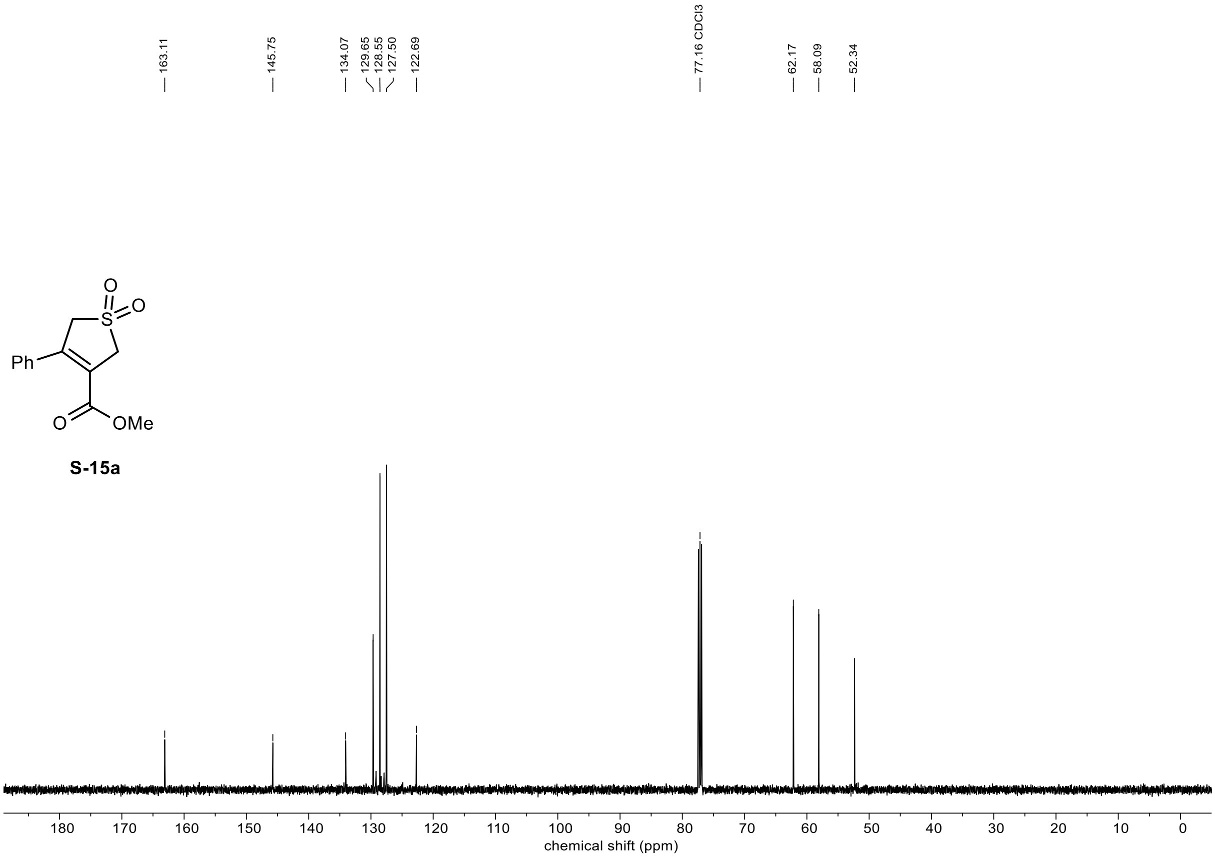

Methyl 2-methylene-3-phenylbut-3-enoate (S-15b)

^{1}H NMR (500 MHz, $CDCl_3$)

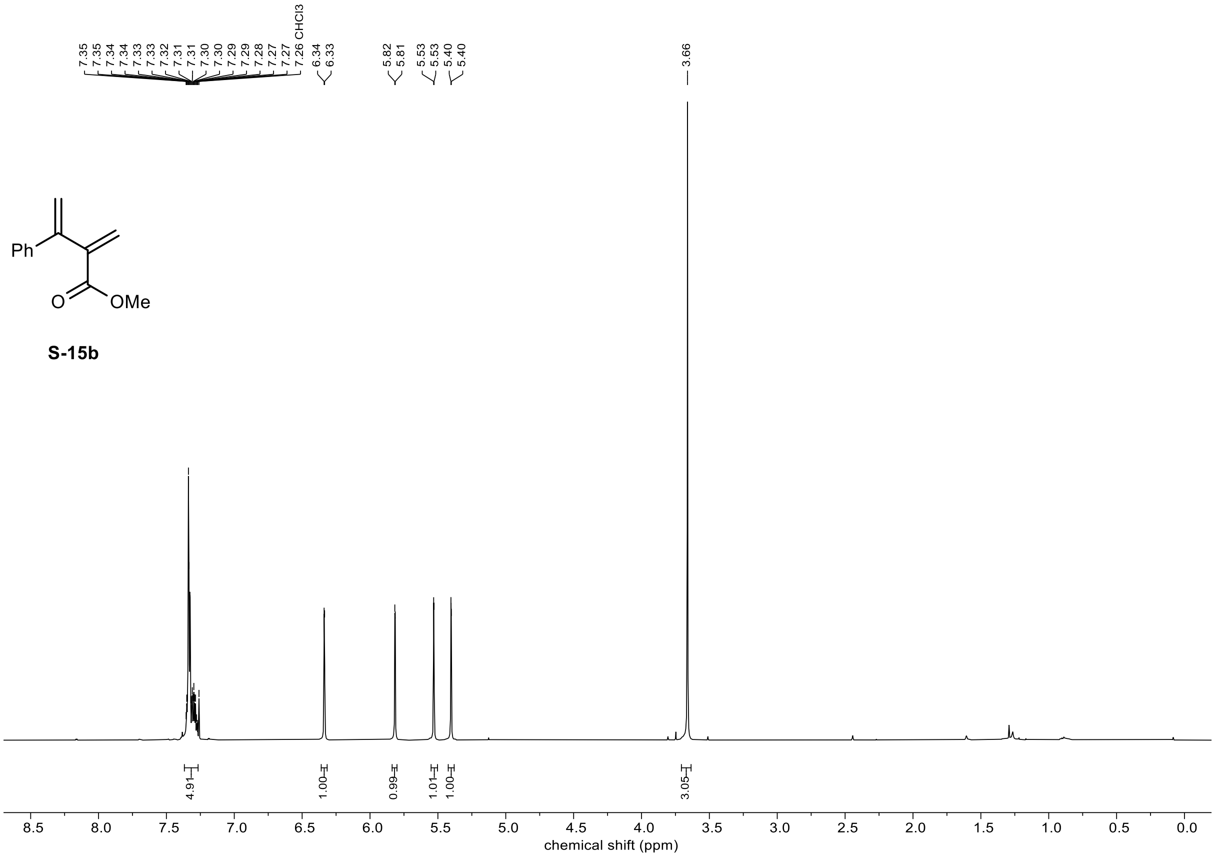

^{13}C NMR (126 MHz, $CDCl_3$)

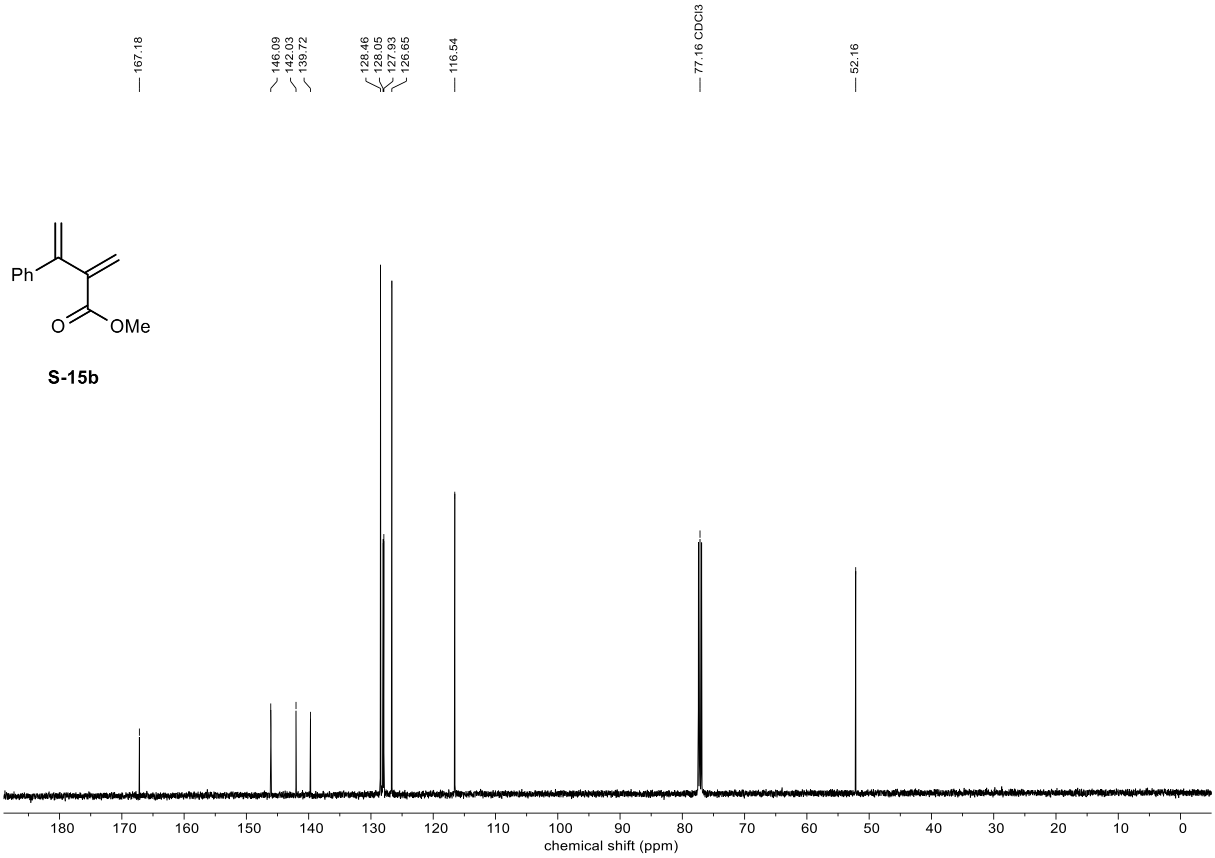

Methyl 2-(dimethoxyphosphoryl)-2-(methylthio)-1-(1-phenylvinyl)cyclopropane-1-carboxylate (11a)

^{1}H NMR (500 MHz, $CDCl_3$)

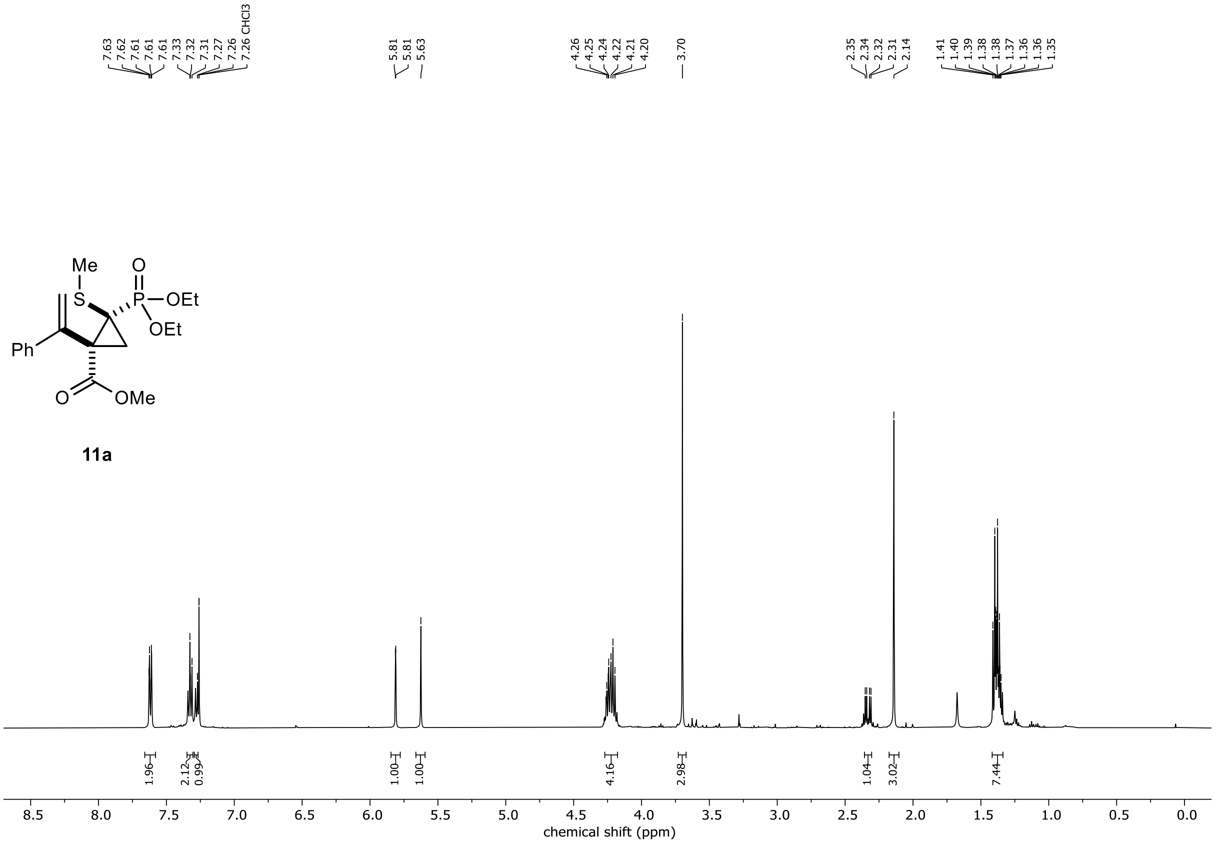

^{13}C NMR (126 MHz, $CDCl_3$)

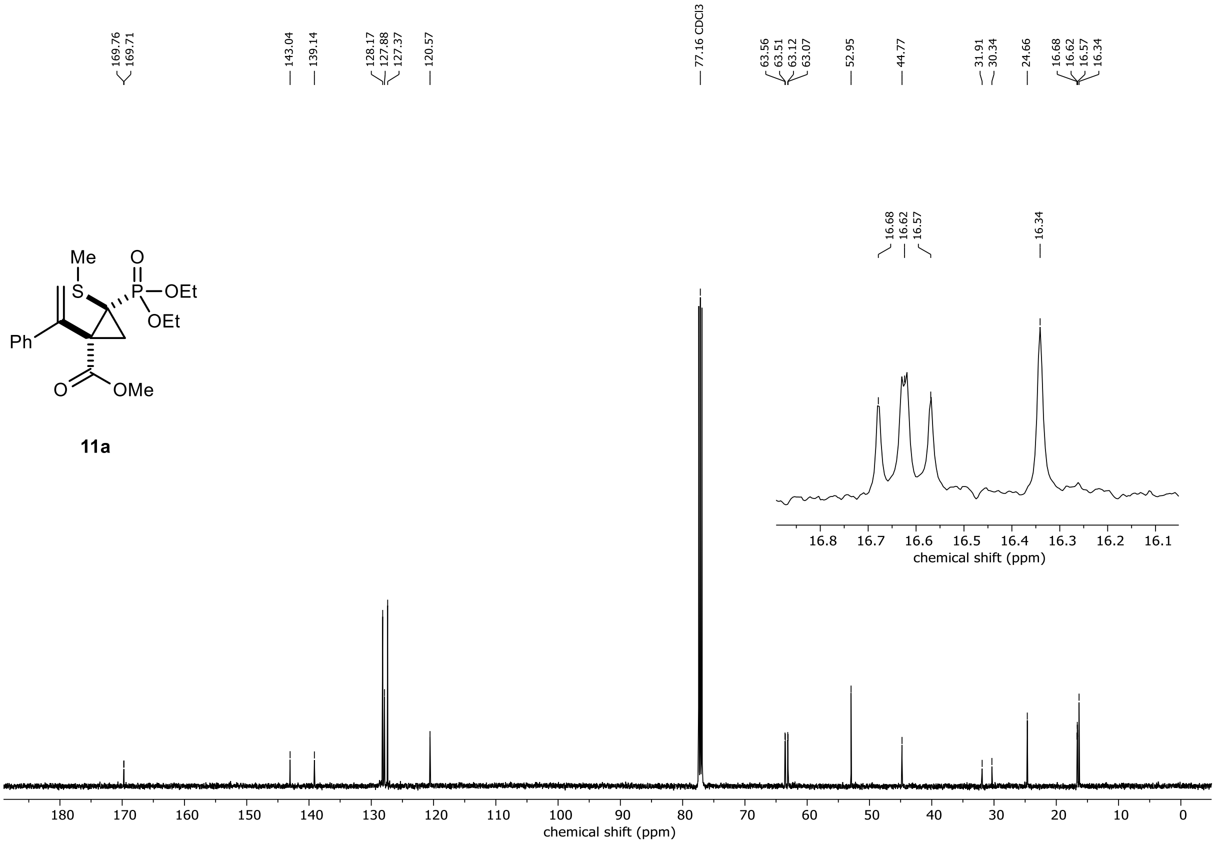

$^{31}P\{^{1}H,^{13}C\}$ NMR (162 MHz, $CDCl_3$)

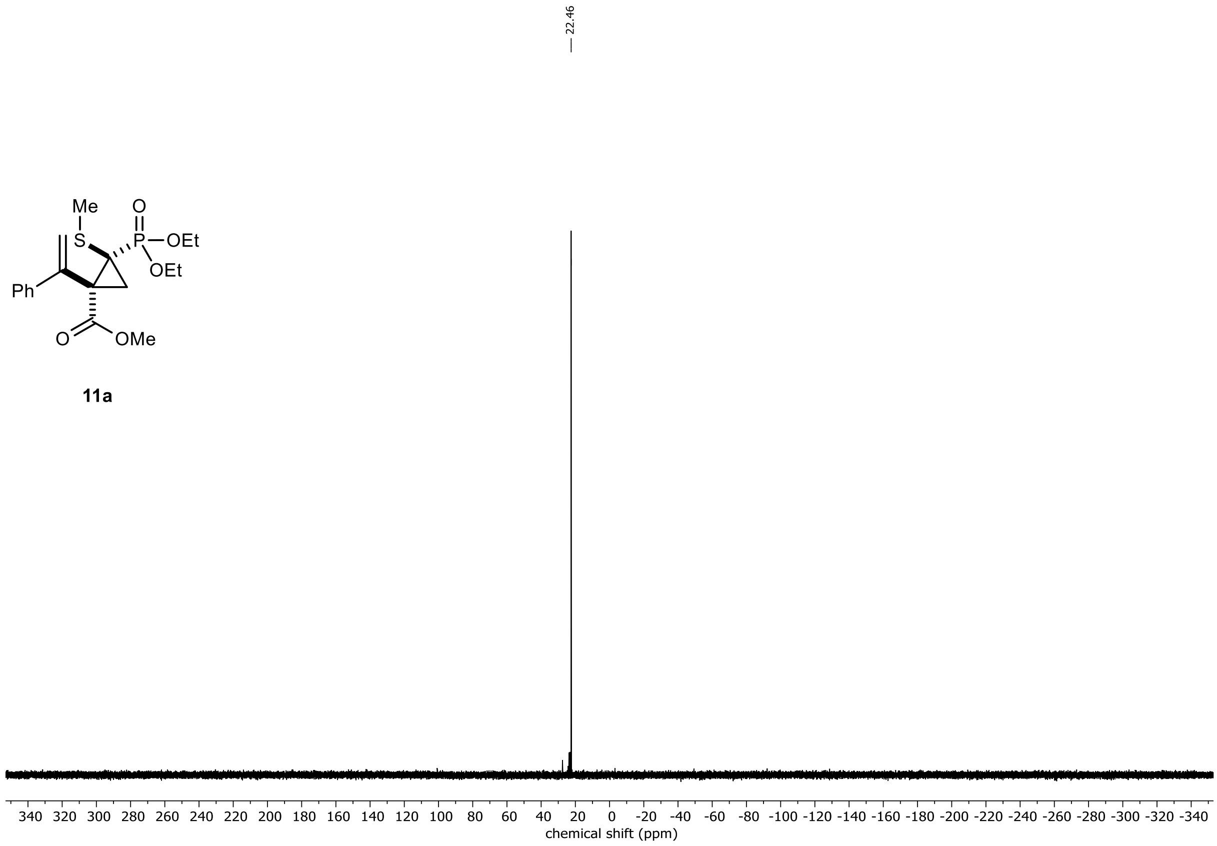

Methyl 2-(methylthio)-1-(1-phenylvinyl)-2-(trifluoromethyl)cyclopropane-1-carboxylate (11b)

^{1}H NMR (500 MHz, $CDCl_3$)

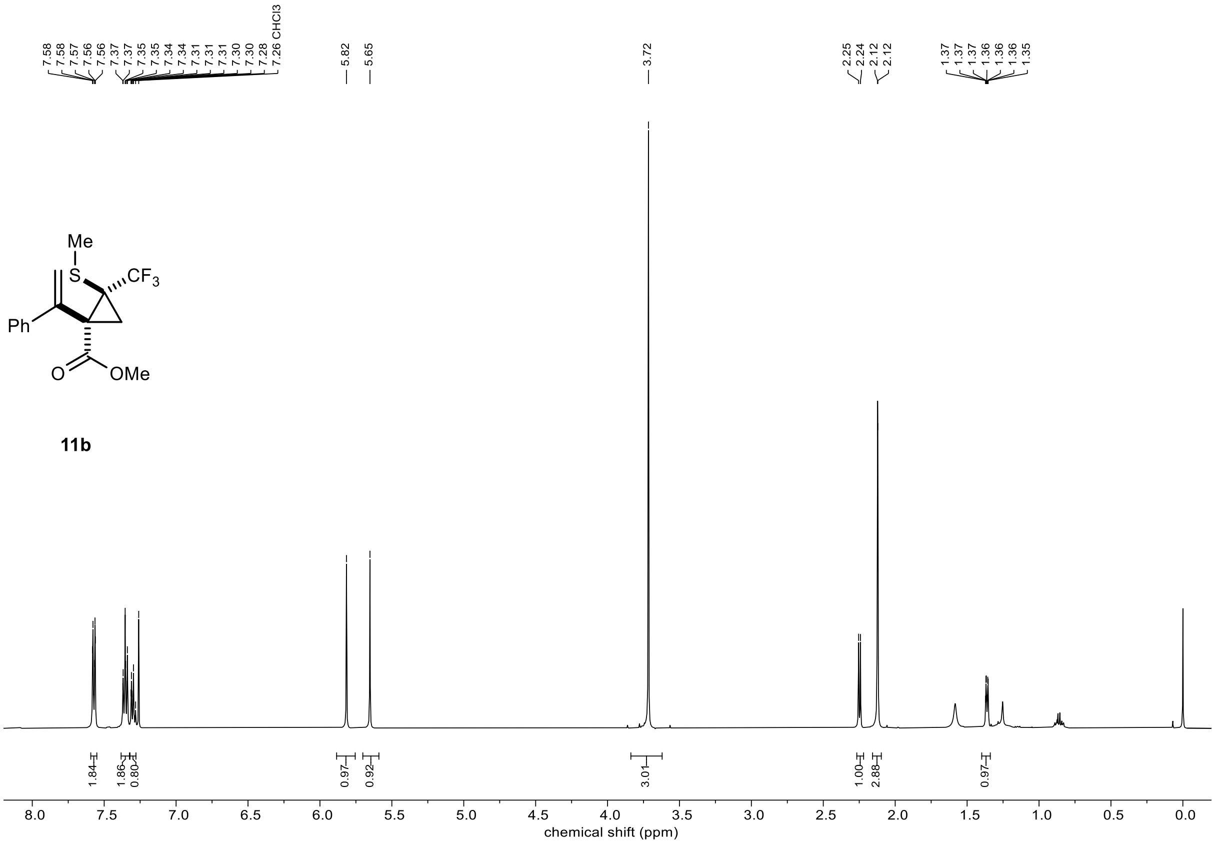

^{13}C NMR (126 MHz, $CDCl_3$)

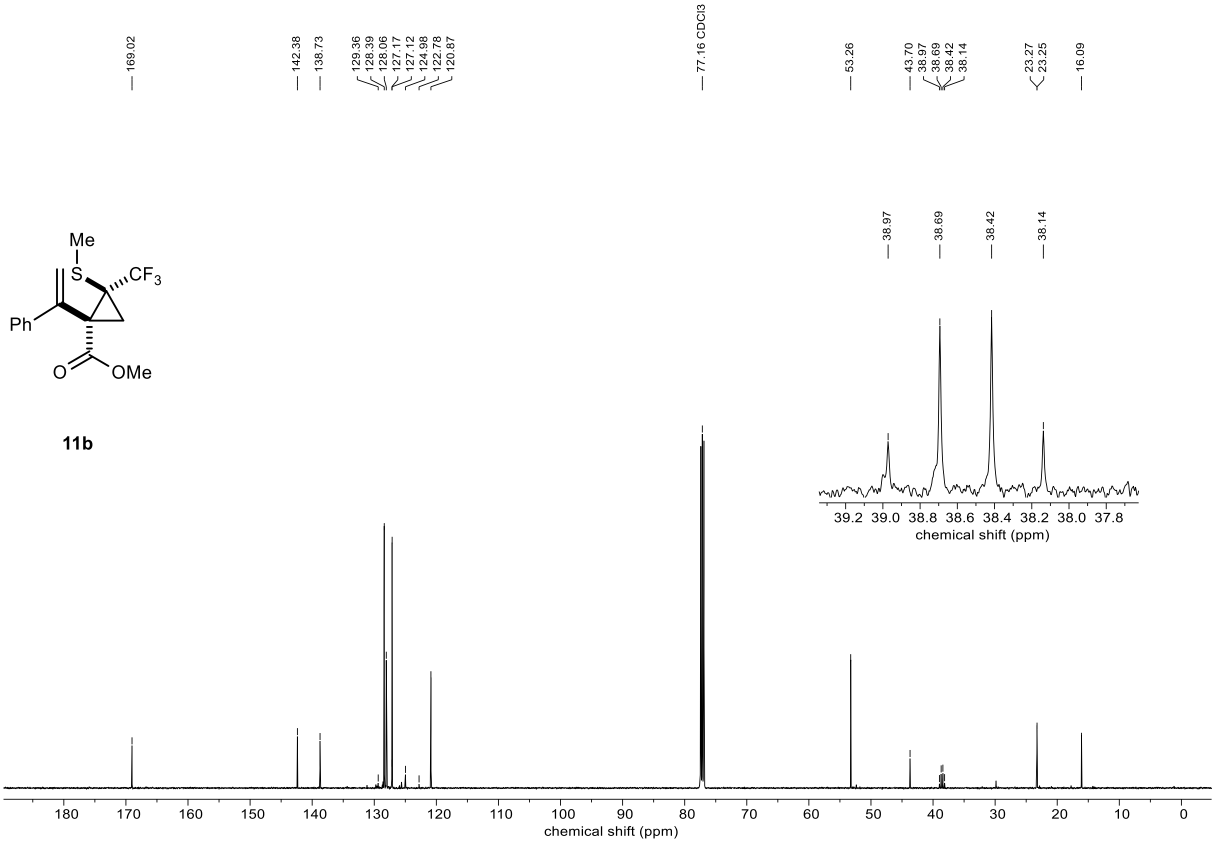

^{19}F NMR (376 MHz, $CDCl_3$)

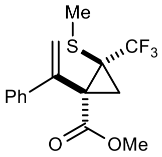

11b

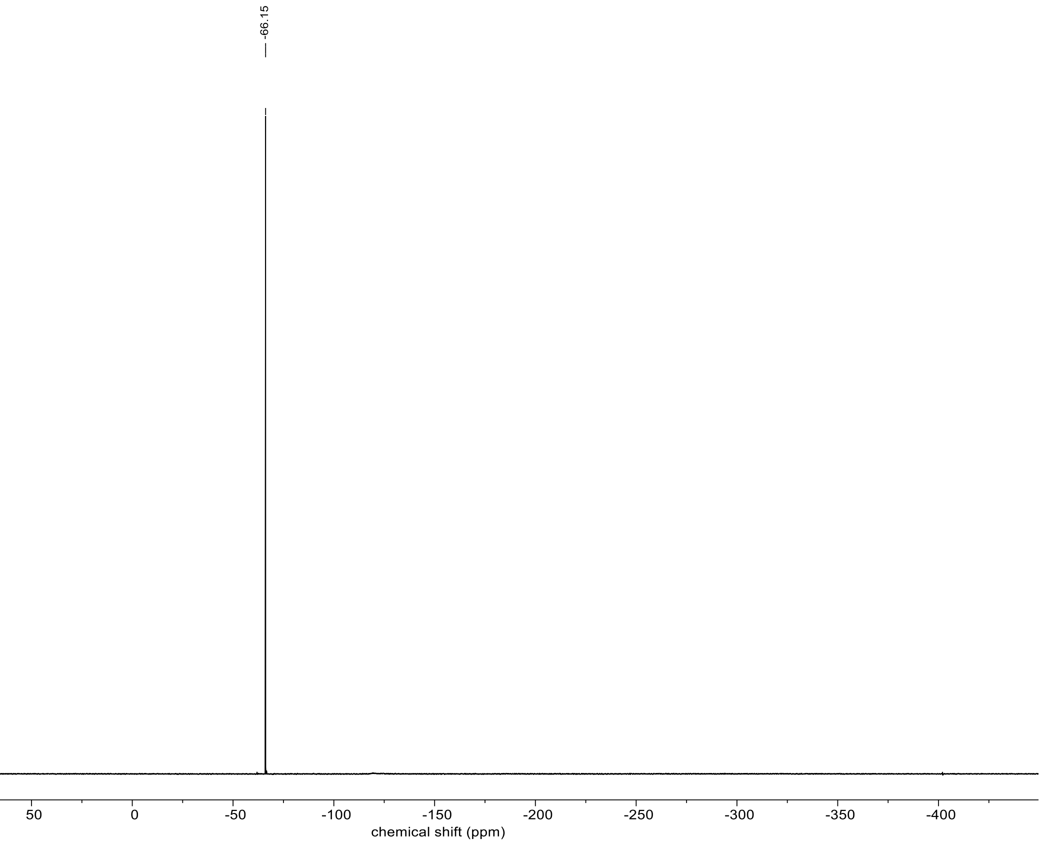

Methyl 2-cyano-2-(methylthio)-1-(1-phenylvinyl)cyclopropane-1-carboxylate (11c)

^{1}H NMR (500 MHz, CDCl$_3$)

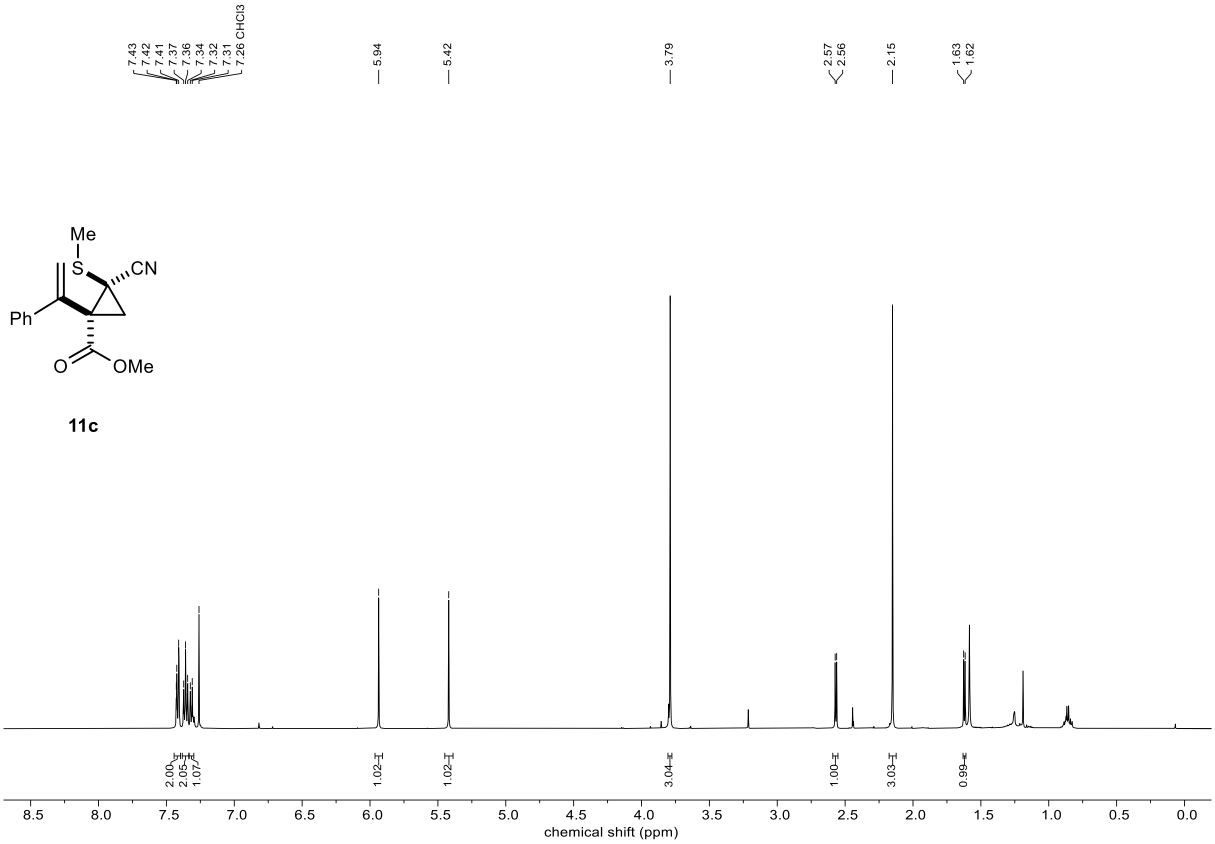

^{13}C NMR (126 MHz, CDCl$_3$)

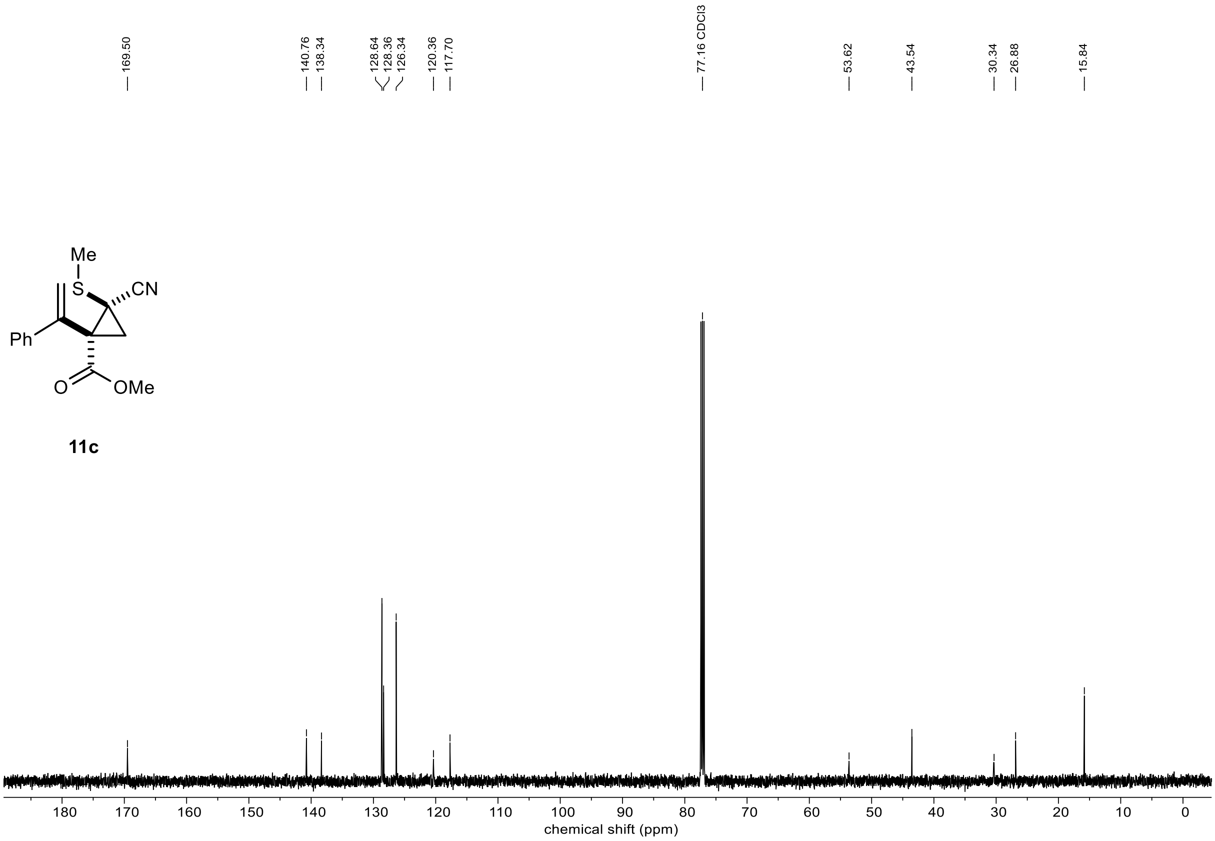

1-Ethyl 2-methyl 1-(methylsulfinyl)-2-(1-phenylvinyl)cyclopropane-1,2-dicarboxylate (12b)

^{1}H NMR (500 MHz, $CDCl_3$)

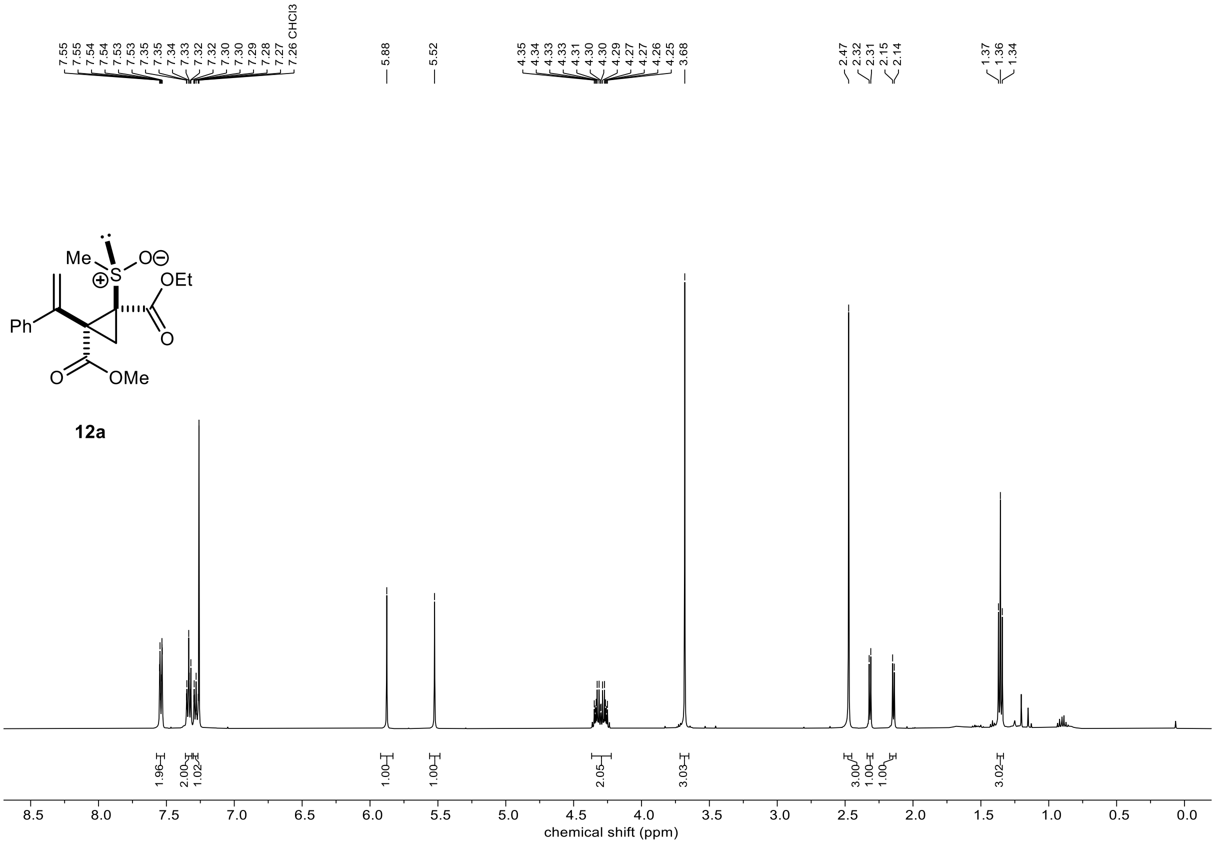

^{13}C NMR (126 MHz, $CDCl_3$)

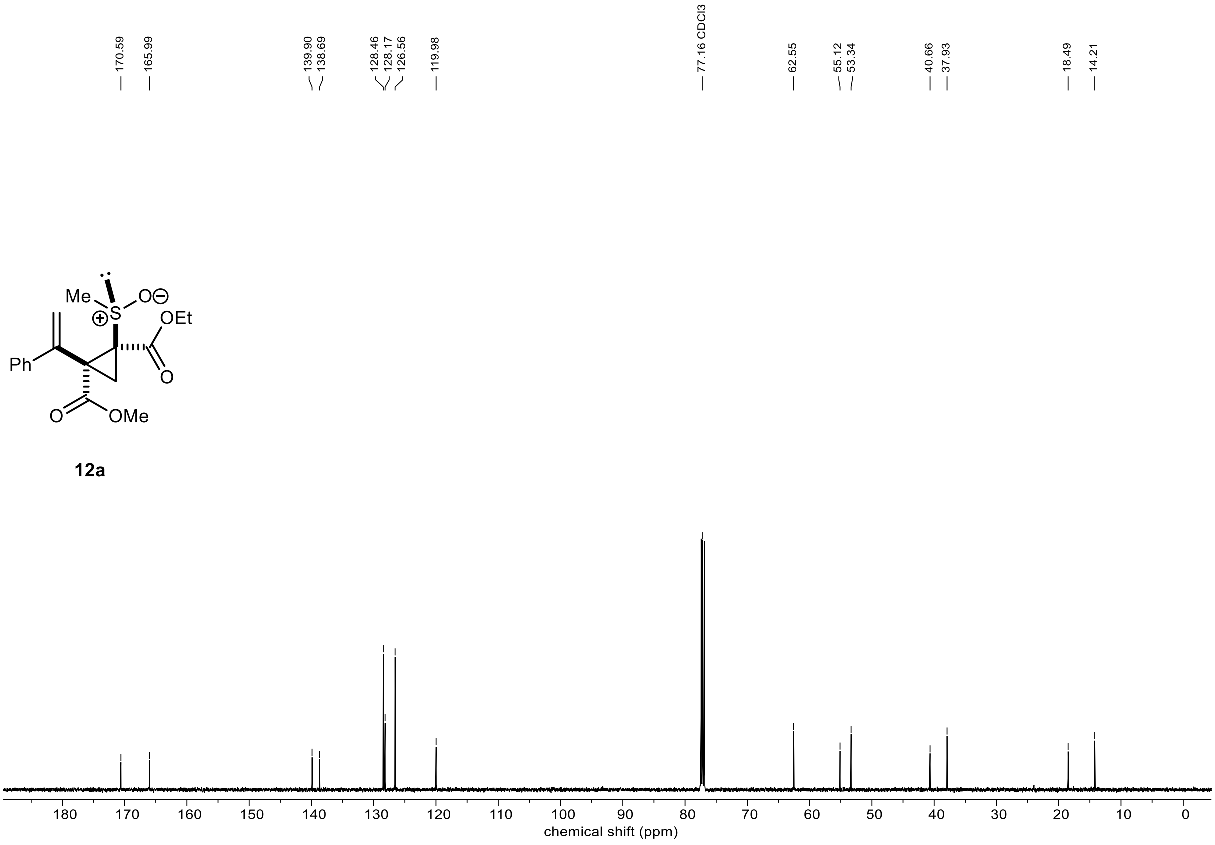

1-Ethyl 2-methyl 1-(methylsulfinyl)-2-(1-phenylvinyl)cyclopropane-1,2-dicarboxylate (12a)

^{1}H NMR (500 MHz, $CDCl_3$)

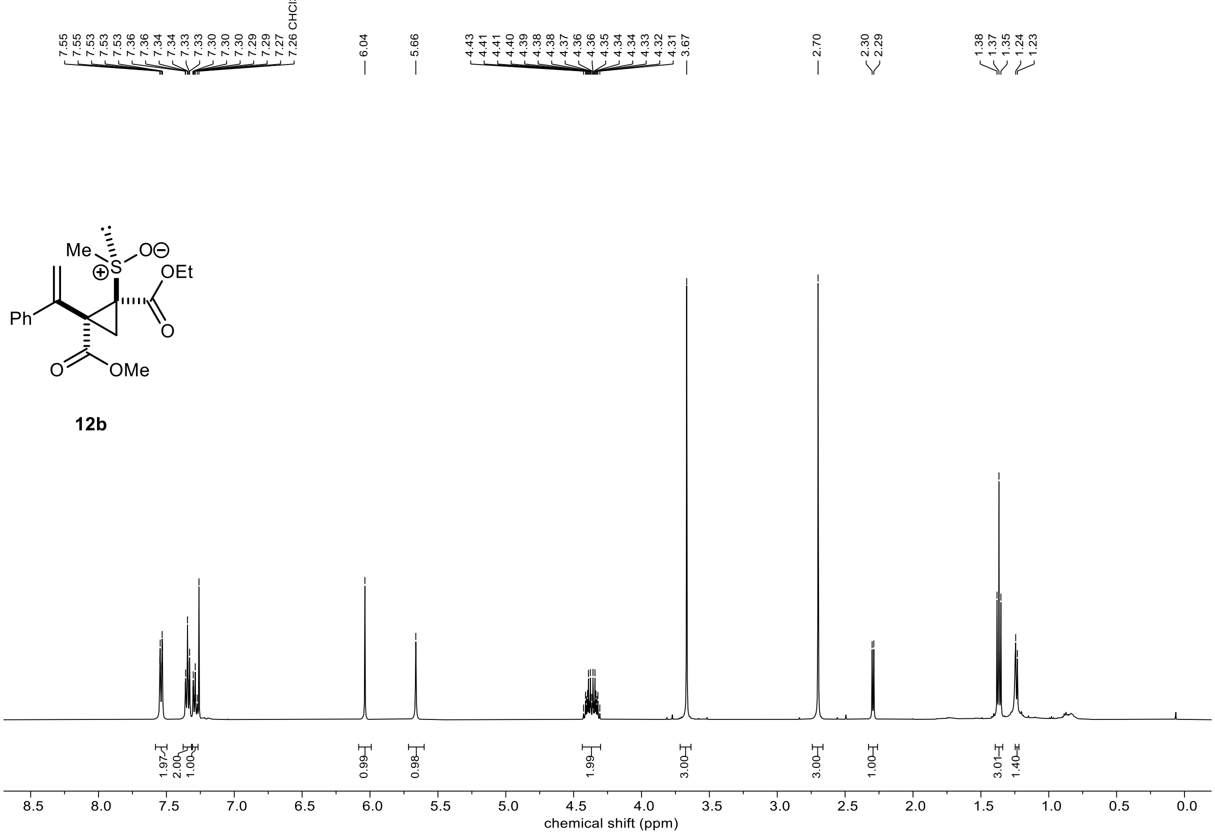

^{13}C NMR (126 MHz, $CDCl_3$)

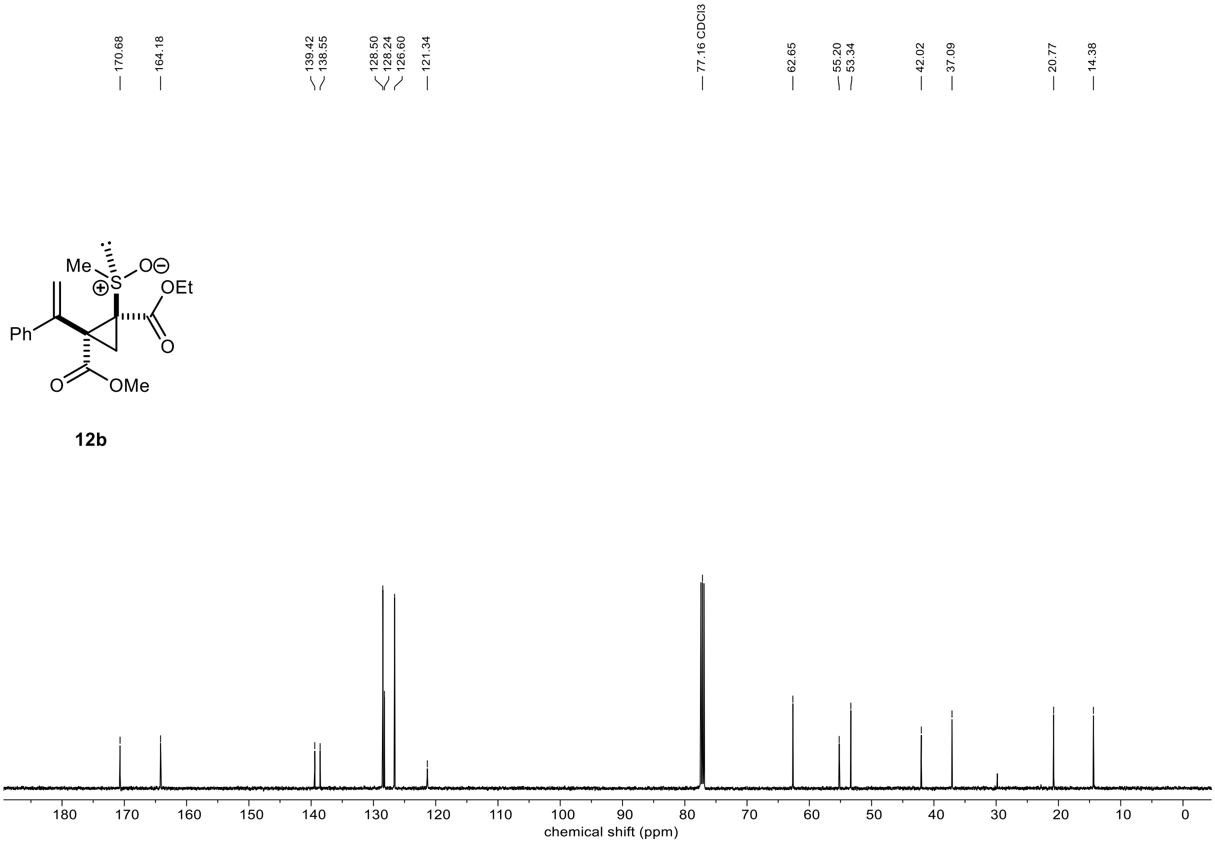

2-Ethyl 1-methyl 1-(1-phenylvinyl)cyclopropane-1,2-dicarboxylate (12c)

^{1}H NMR (500 MHz, $CDCl_3$)

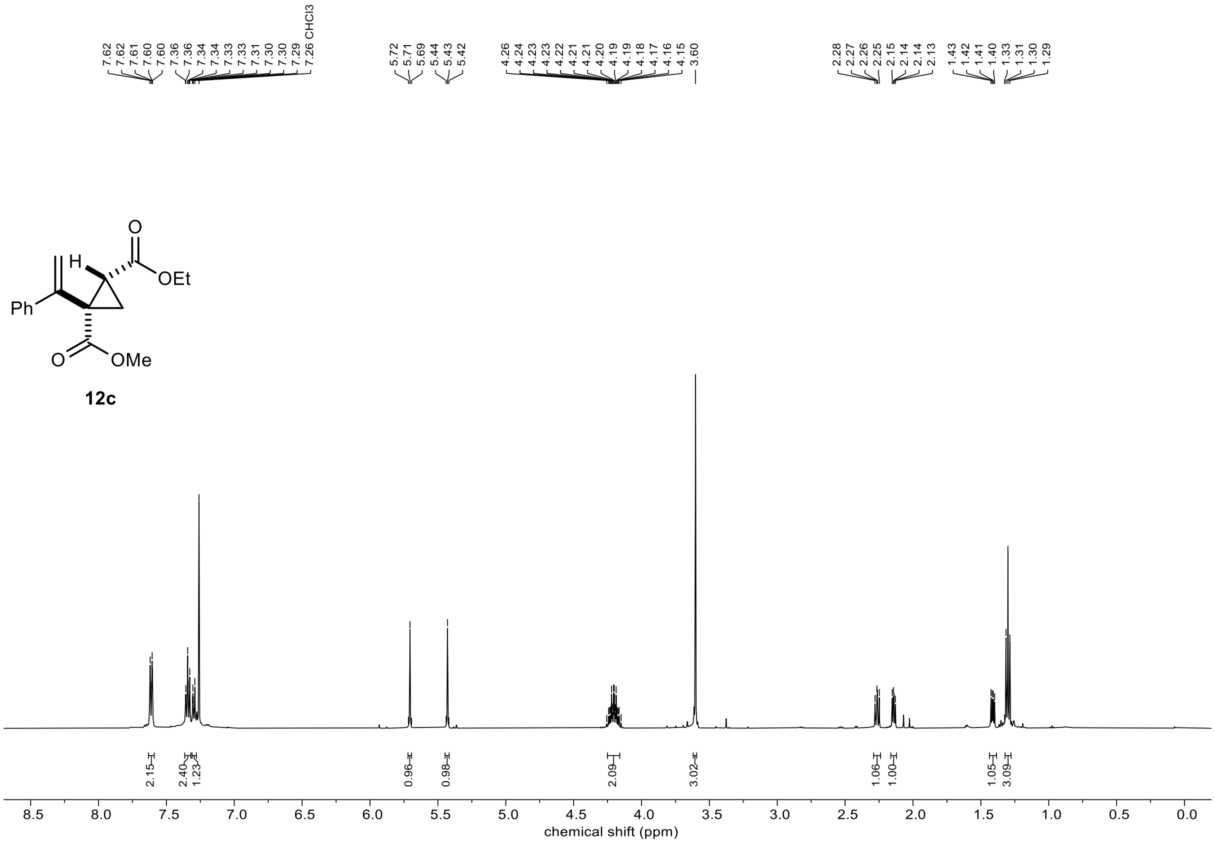

^{13}C NMR (126 MHz, $CDCl_3$)

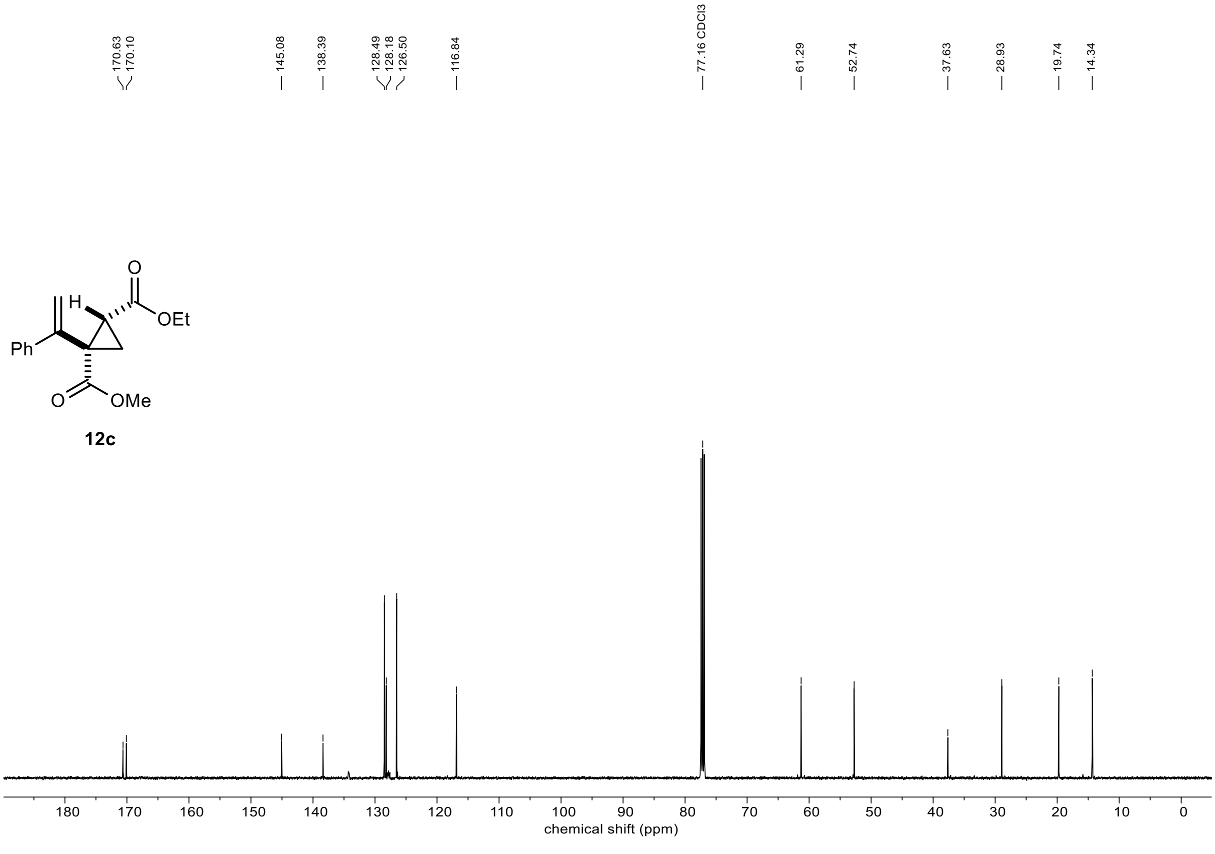

1-Ethyl 2-methyl 1-(methylsulfonyl)-2-(1-phenylvinyl)cyclopropane-1,2-dicarboxylate (12d)

^{1}H NMR (500 MHz, $CDCl_3$)

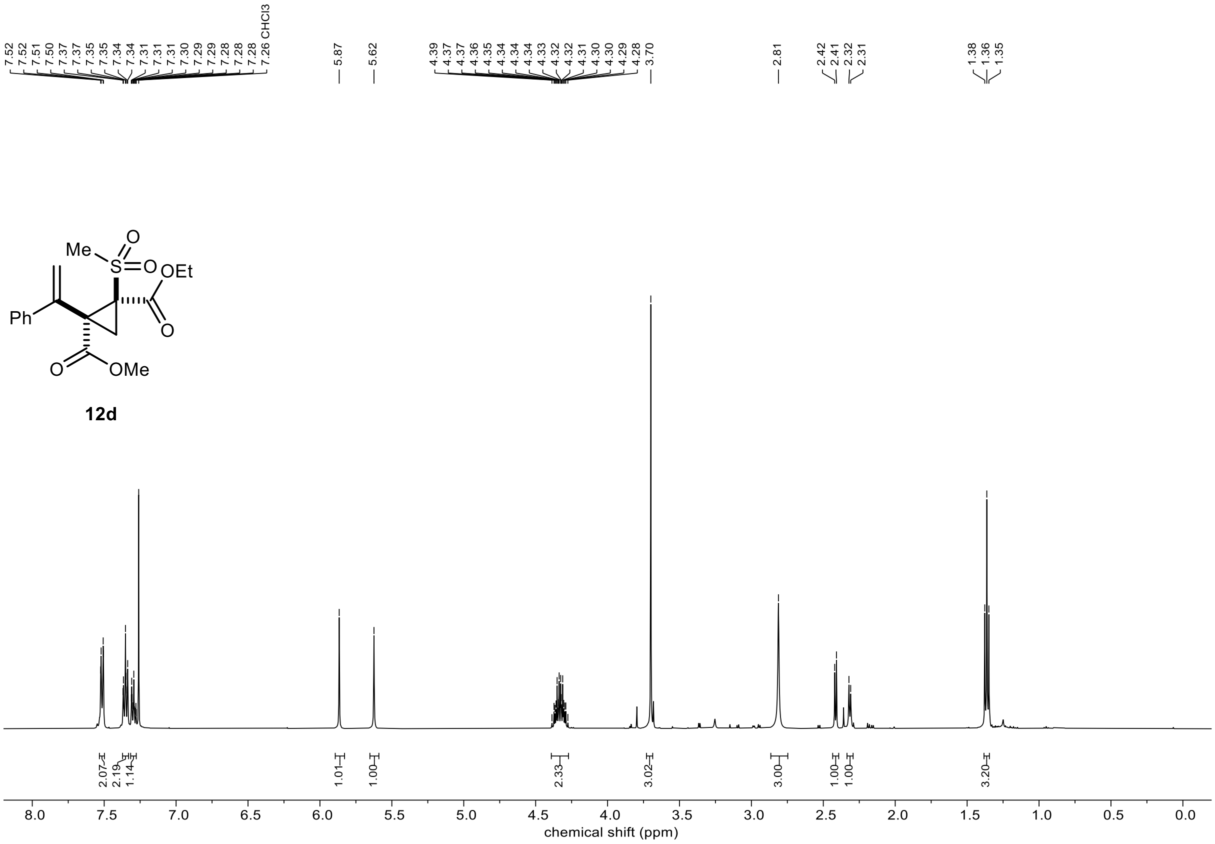

^{13}C NMR (126 MHz, $CDCl_3$)

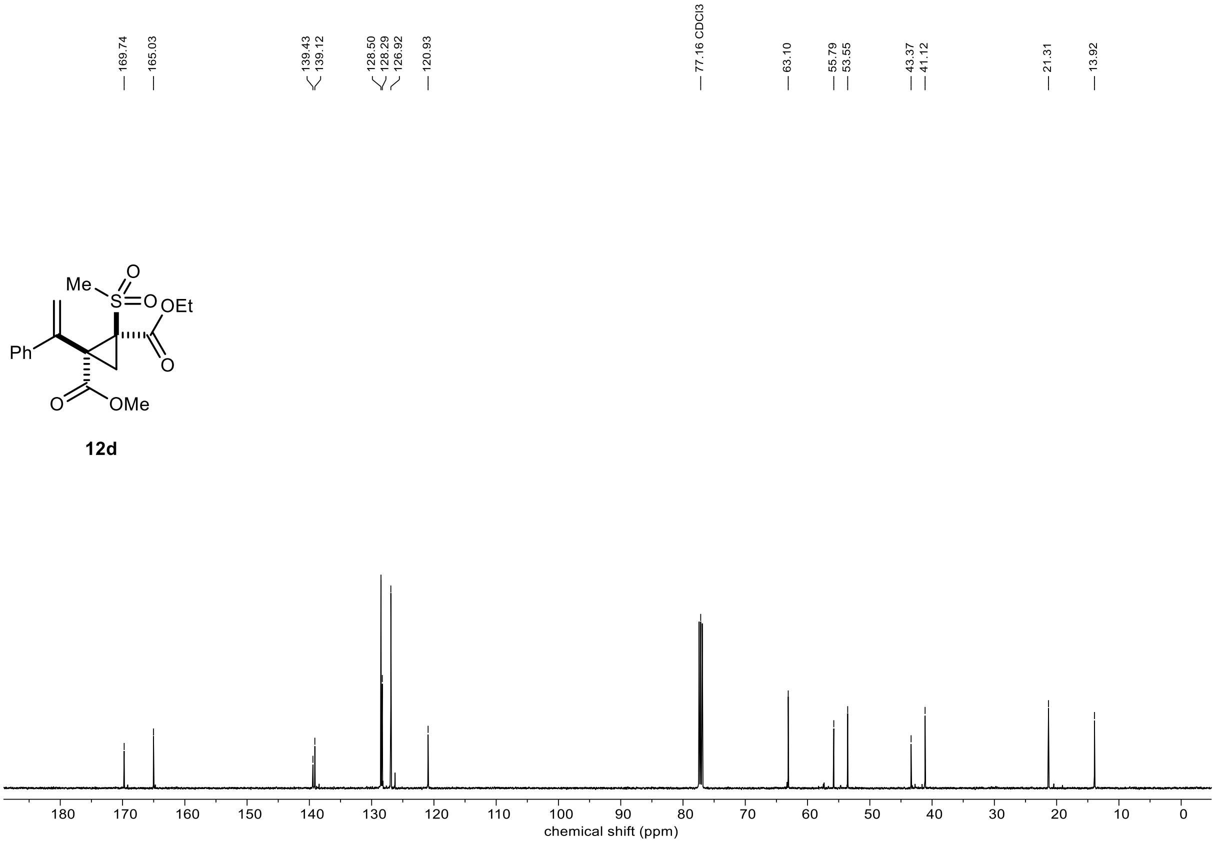

1-Ethyl 2-methyl 1-(*S*-methyl-*N*-tosylsulfinimidoyl)-2-(1-phenylvinyl)cyclopropane-1,2-dicarboxylate (12e)

^{1}H NMR (500 MHz, $CDCl_3$)

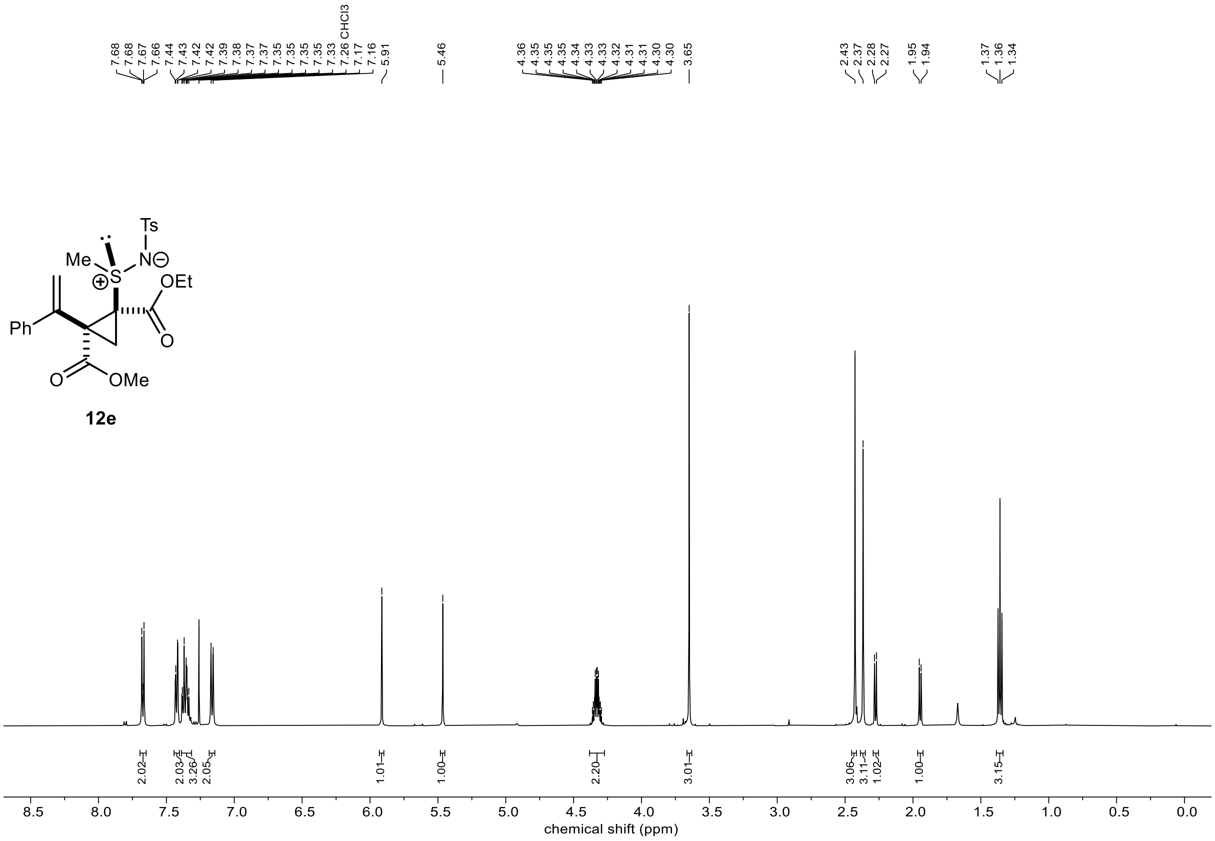

^{13}C NMR (126 MHz, $CDCl_3$)

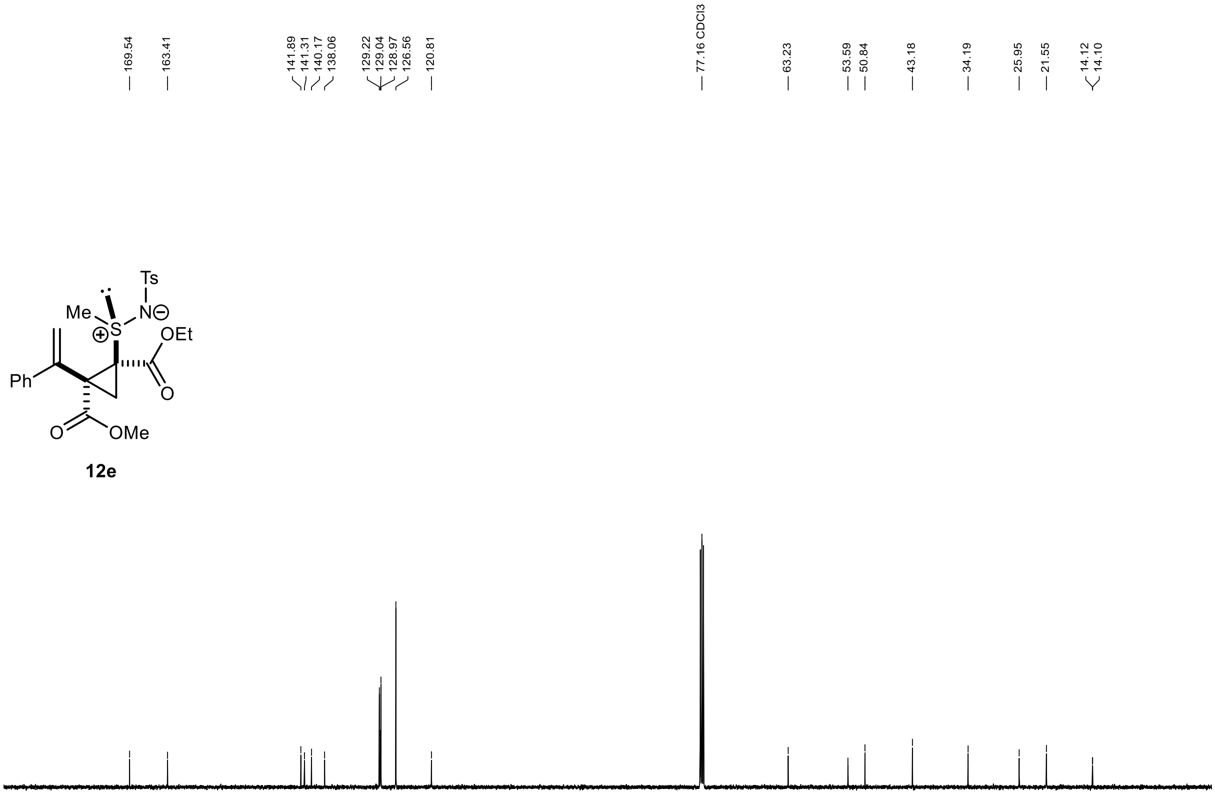

13. HRMS Spectra

Methyl 4-(4-methoxyphenyl)-2,5-dihydrothiophene-3-carboxylate (S-9b)

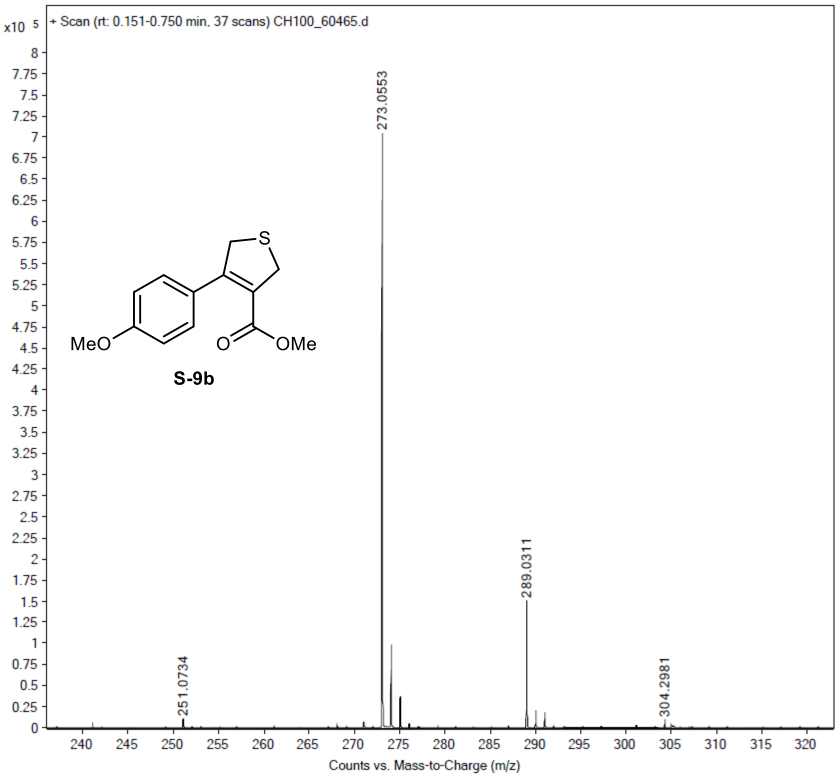

Methyl 4-(4-(trifluoromethyl)phenyl)-2,5-dihydrothiophene-3-carboxylate (S-9c)

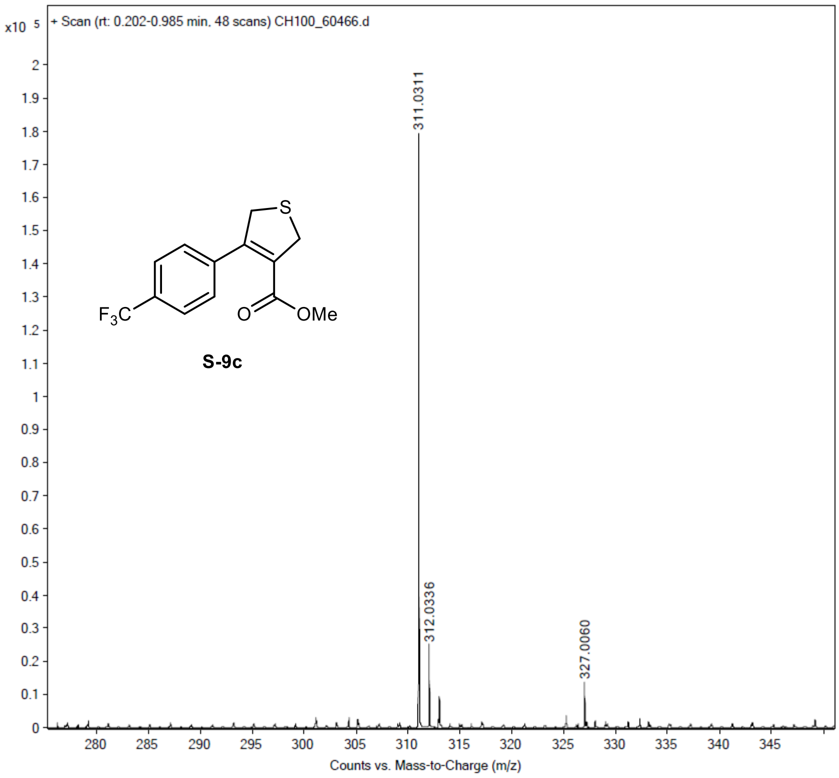

Methyl 4-(4-(ethoxycarbonyl)phenyl)-2,5-dihydrothiophene-3-carboxylate (S-9f)

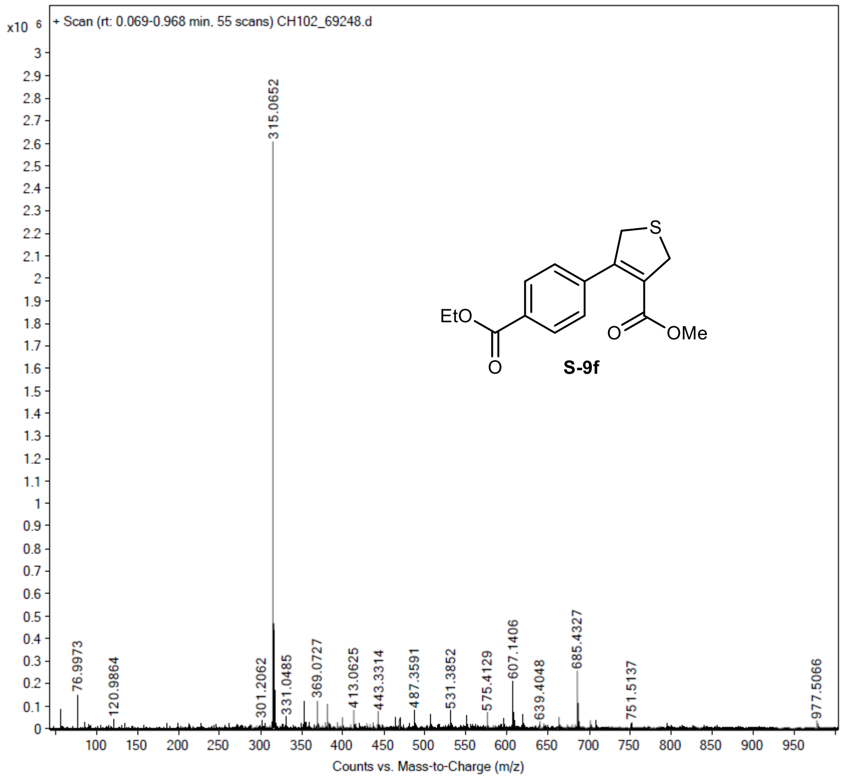

***Tert*-butyl 5-(4-(methoxycarbonyl)-2,5-dihydrothiophen-3-yl)-1H-indole-1-carboxylate (S-9k)**

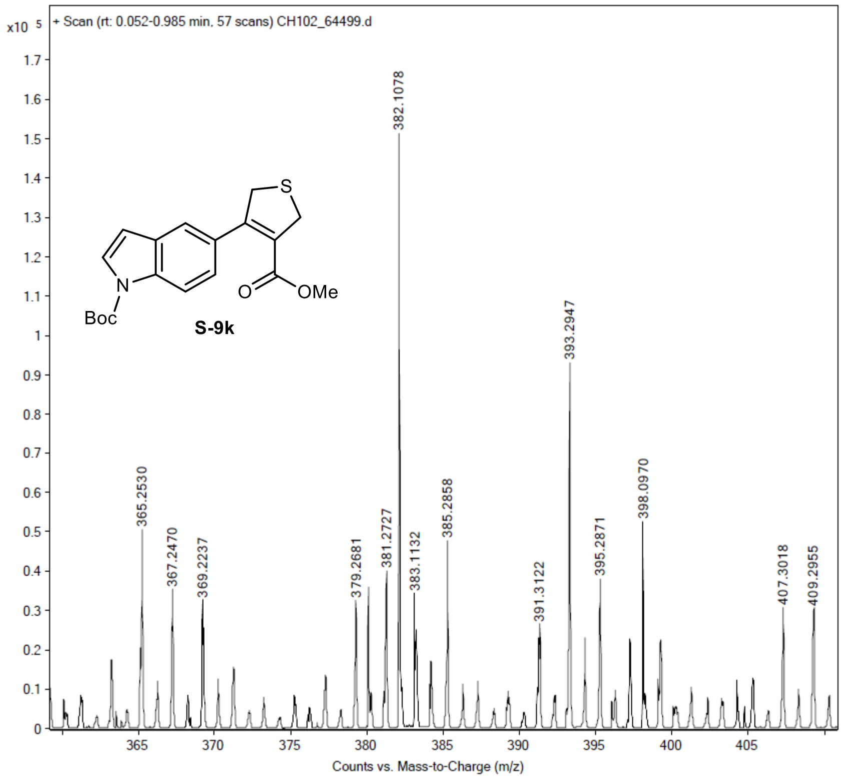

Methyl 4-((triisopropylsilyl)oxy)-2,5-dihydrothiophene-3-carboxylate (S-9m)

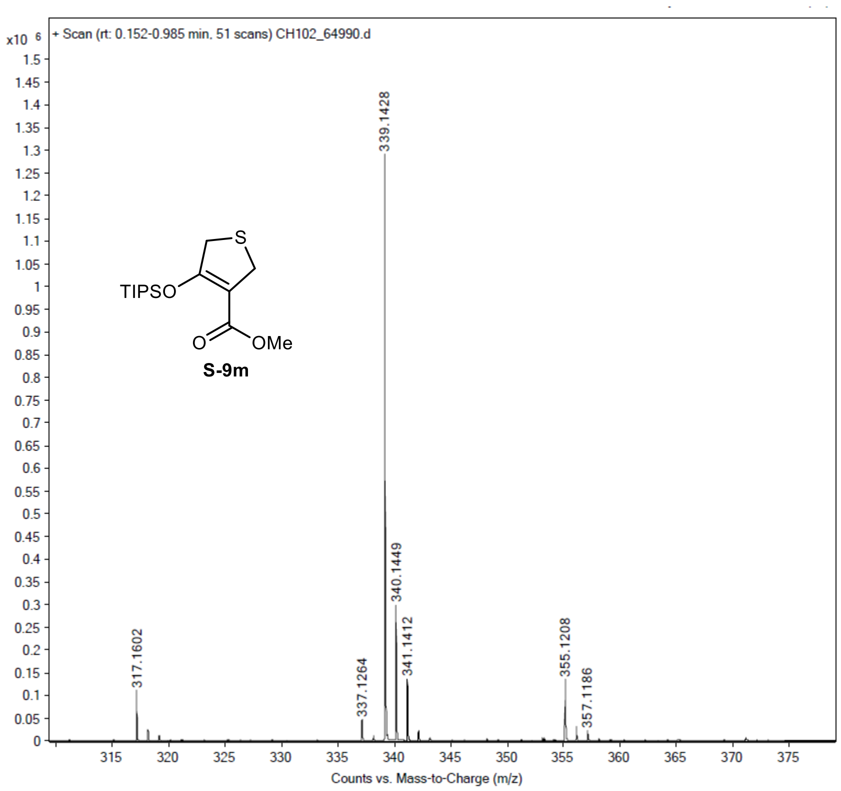

Methyl 4-hydroxy-5-methyl-2,5-dihydrothiophene-3-carboxylate (S-9q)

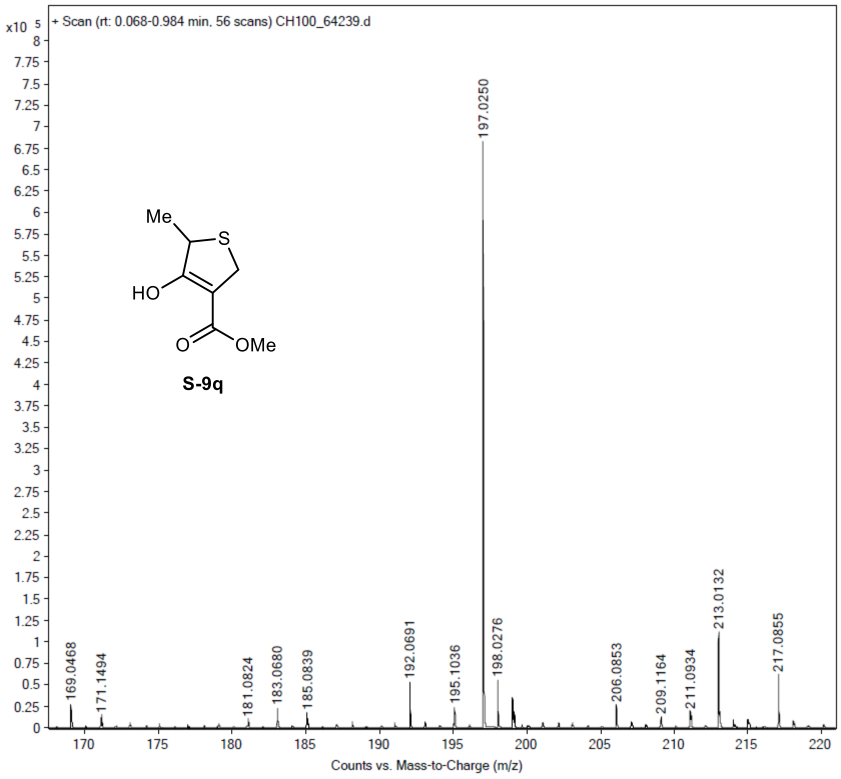

Methyl 5-methyl-4-(((trifluoromethyl)sulfonyl)oxy)-2,5-dihydrothiophene-3-carboxylate (S-9p)

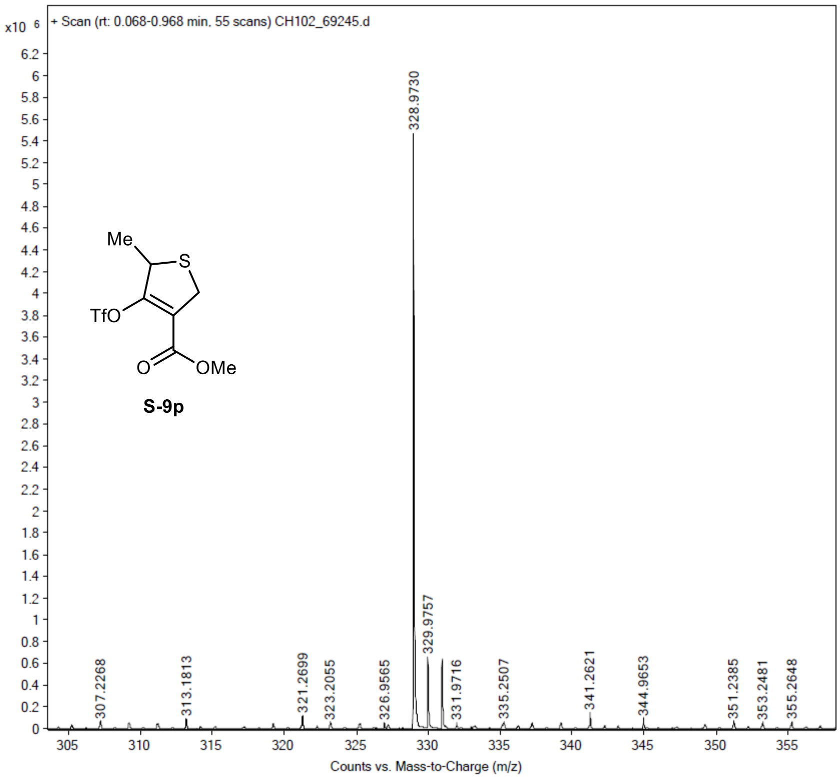

Methyl 5-methyl-4-phenyl-2,5-dihydrothiophene-3-carboxylate (S-9o)

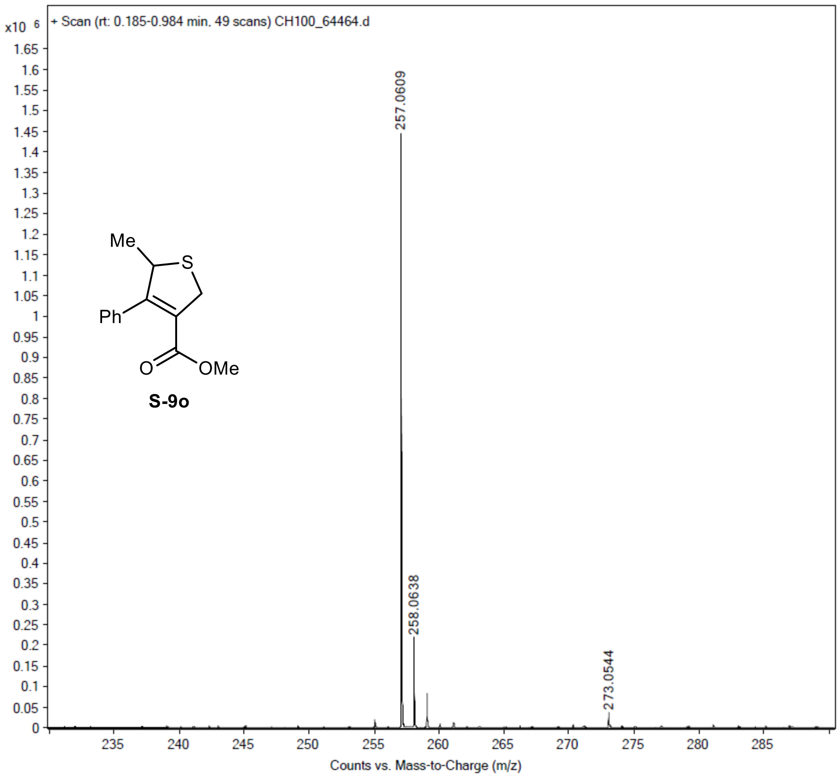

Methyl (*Z*)-3-(4-methoxyphenyl)-2-methylene-4-(methylthio)but-3-enoate (S-1b)

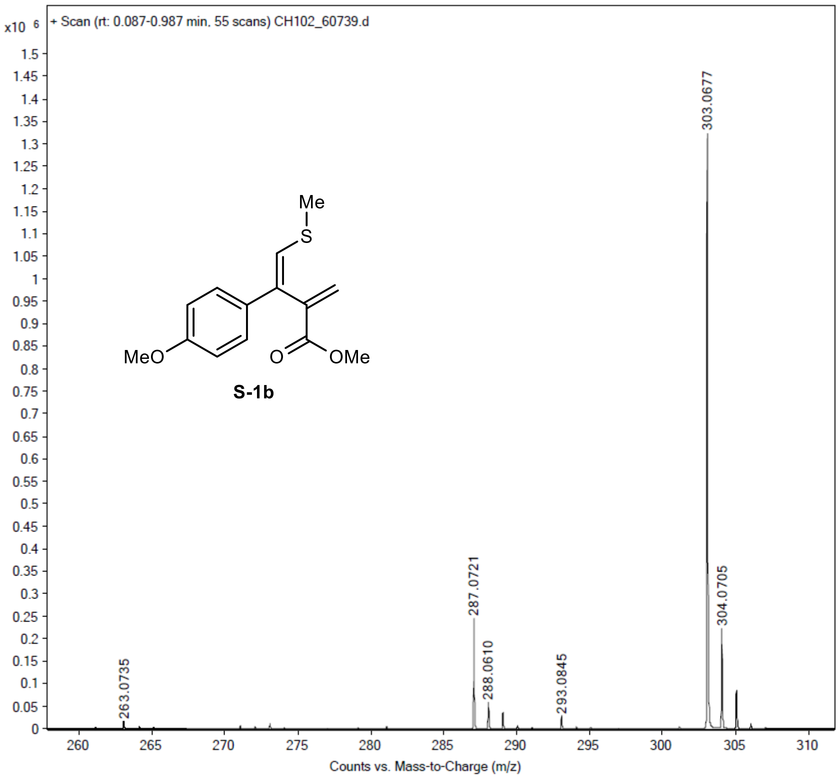

Methyl (*Z*)-2-methylene-4-(methylthio)-3-(4-(trifluoromethyl)phenyl)but-3-enoate (S-1c)

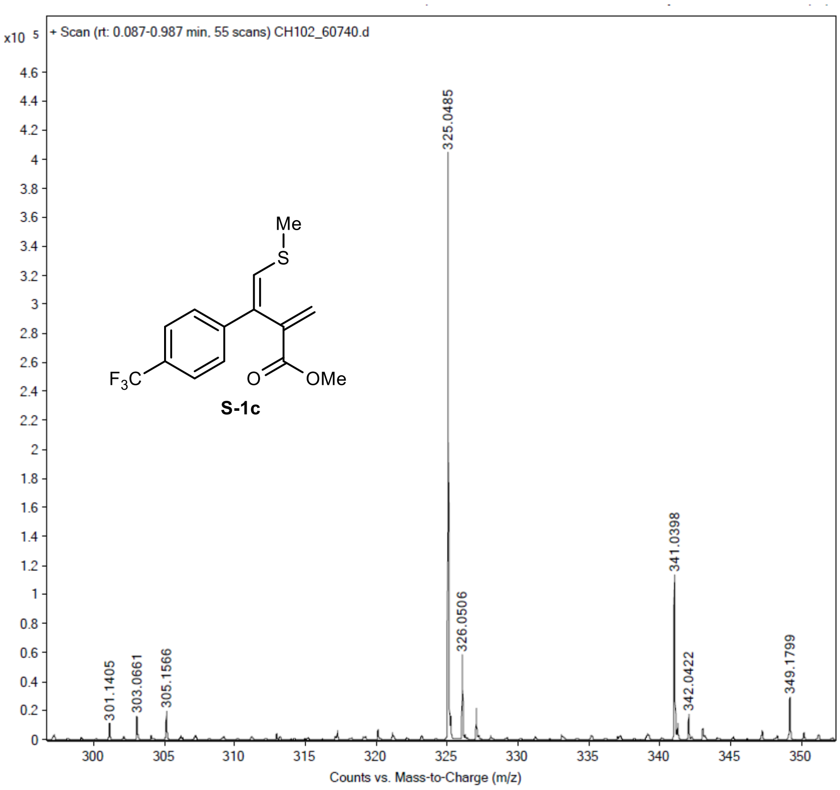

Ethyl (*Z*)-4-(3-(methoxycarbonyl)-1-(methylthio)buta-1,3-dien-2-yl)benzoate (S-1f)

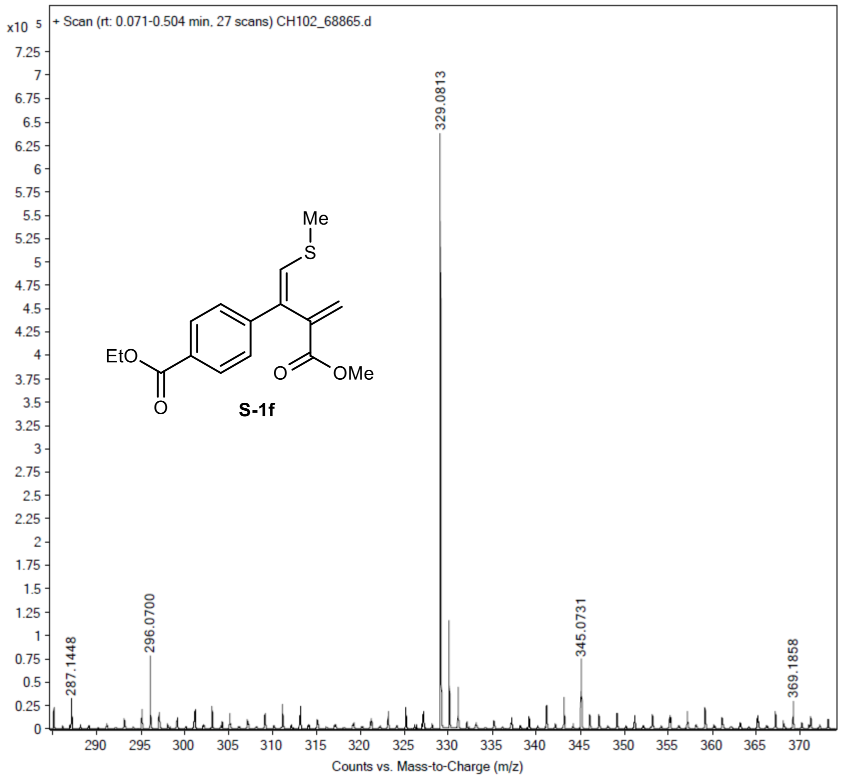

***Tert*-butyl (*Z*)-5-(3-(methoxycarbonyl)-1-(methylthio)buta-1,3-dien-2-yl)-1H-indole-1-carboxylate (S-1k)**

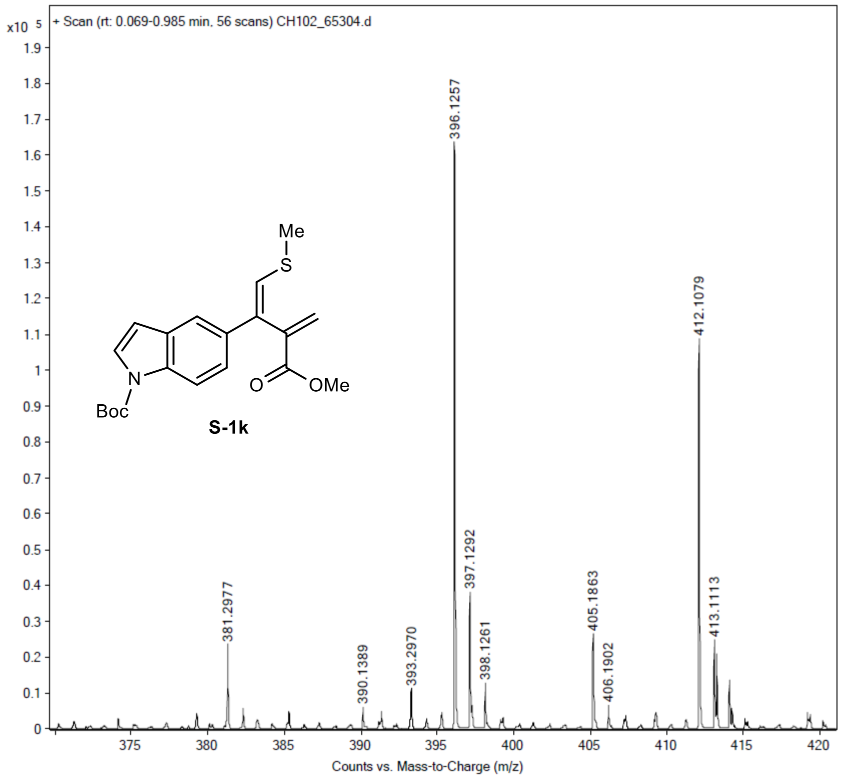

Methyl (*E*)-2-methylene-4-(methylthio)-3-((triisopropylsilyl)oxy)but-3-enoate (S-1m)

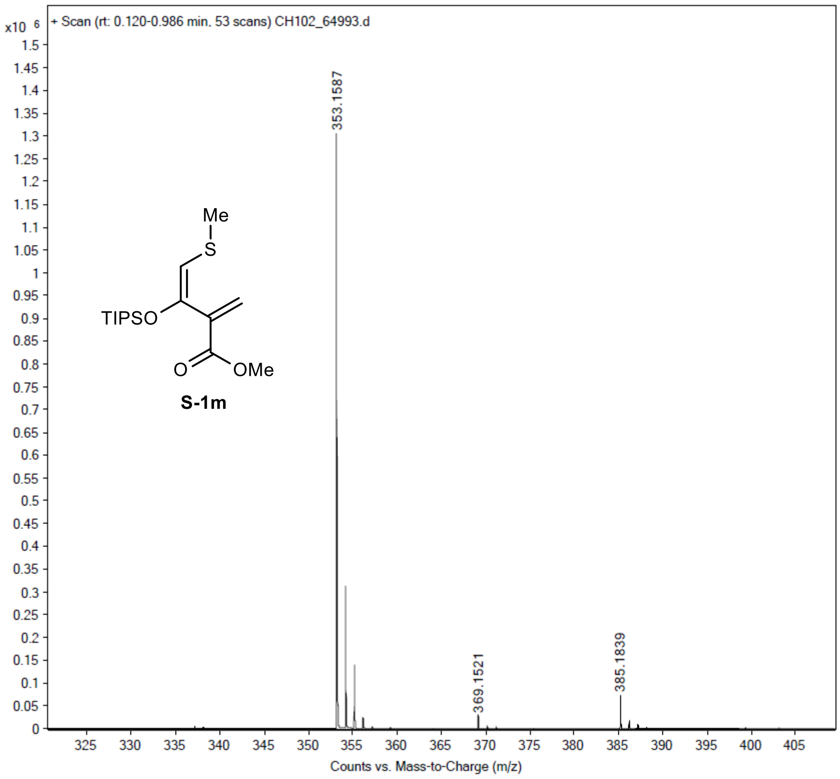

Methyl (*Z*)-2-methylene-4-(methylthio)-3-phenylpent-3-enoate (S-1o)

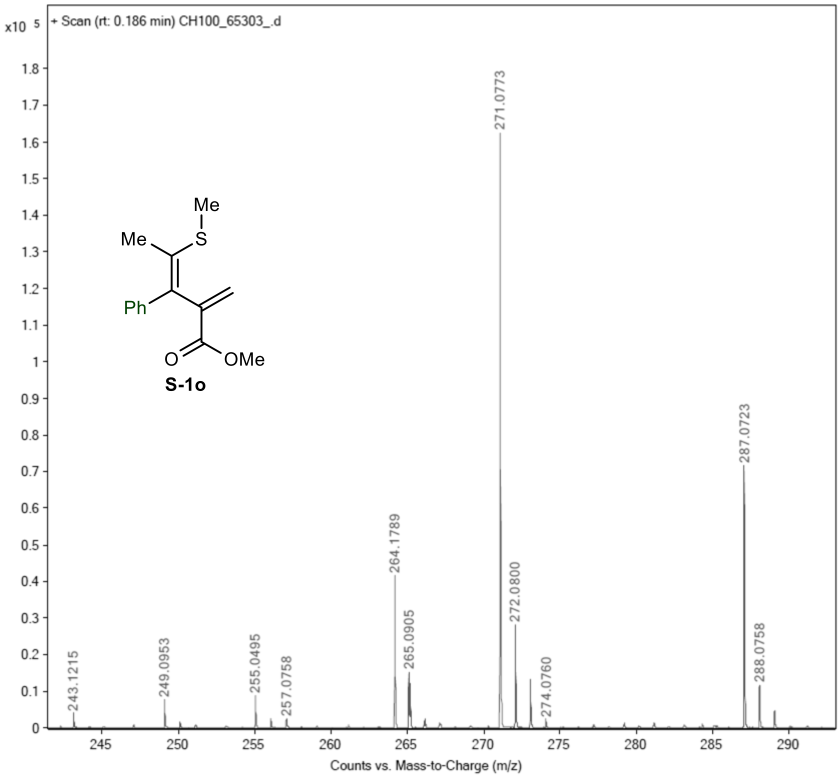

Methyl (*Z*)-4-((2-ethoxy-2-oxoethyl)thio)-2-methylene-3-phenylbut-3-enoate (S-1bb)

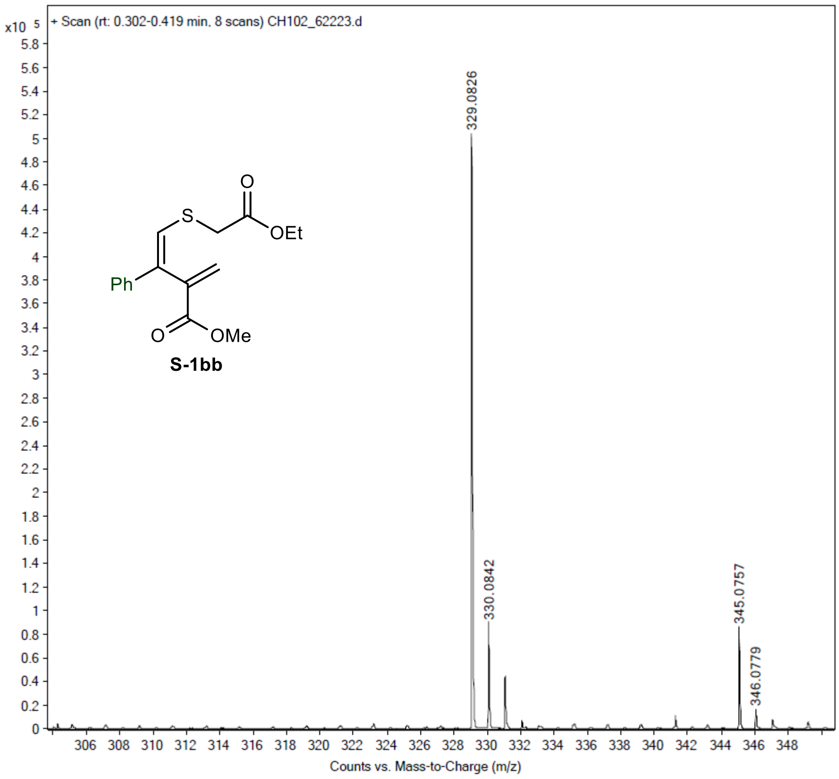

Methyl (*Z*)-4-(benzylthio)-2-methylene-3-phenylbut-3-enoate (S-1bc)

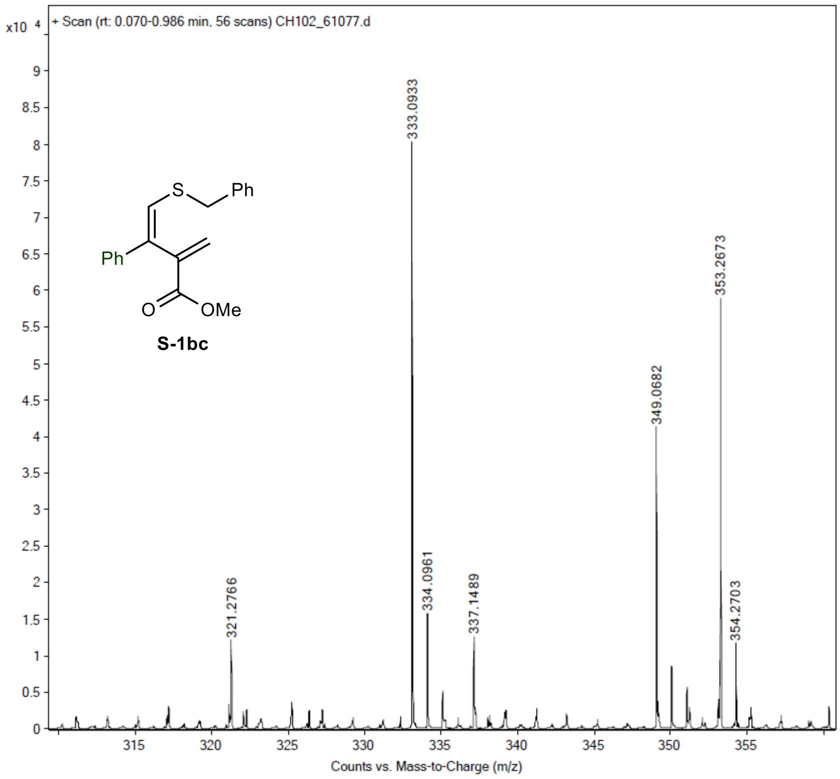

Methyl (*Z*)-4-(allylthio)-2-methylene-3-phenylbut-3-enoate (S-1be)

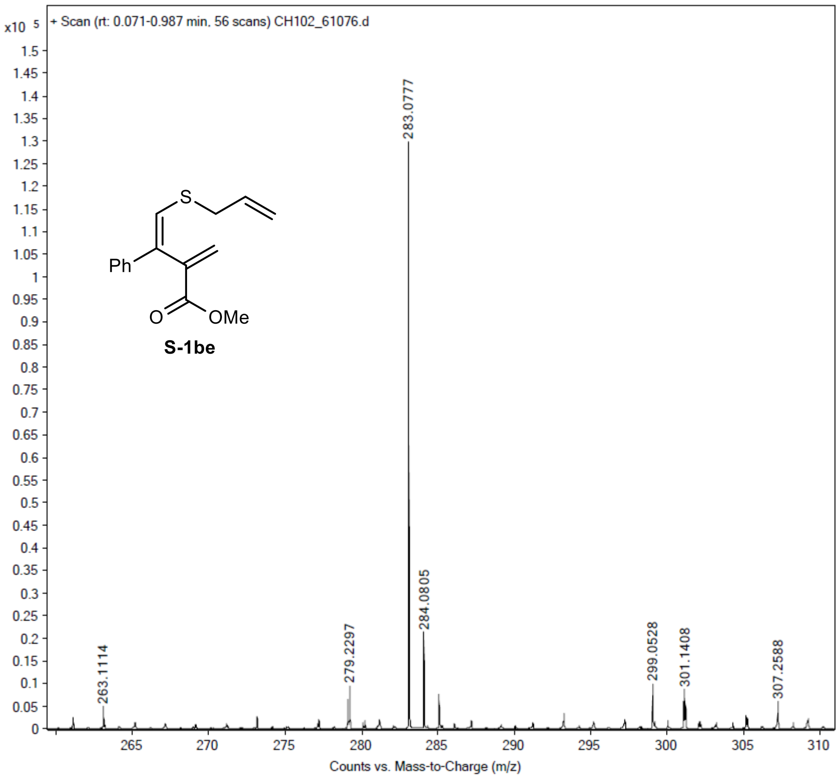

Methyl (*Z*)-4-(((diethoxyphosphoryl)methyl)thio)-2-methylene-3-phenylbut-3-enoate (S-1ca)

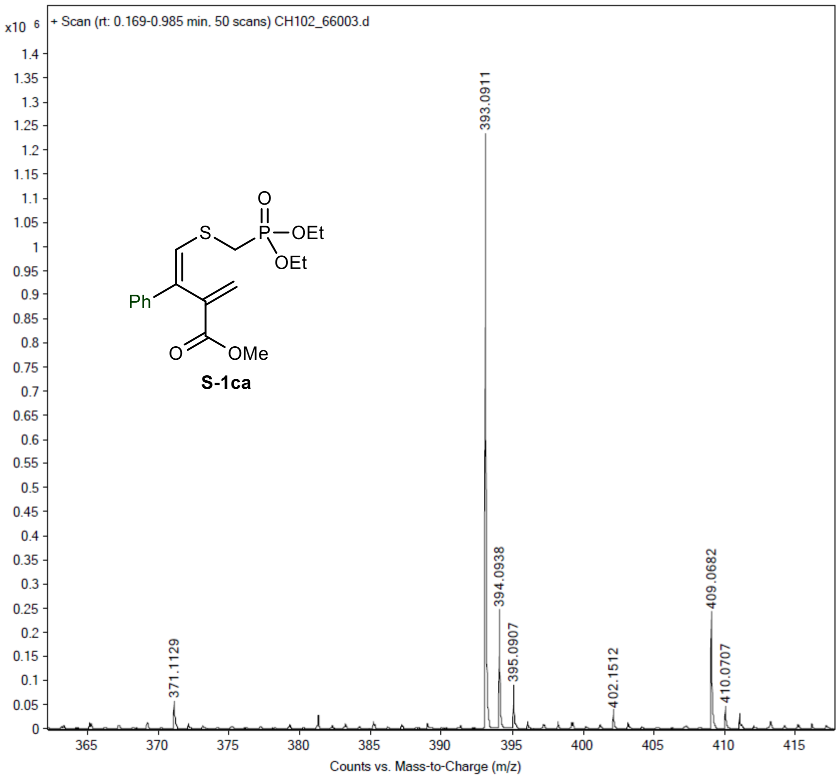

Methyl (*Z*)-2-methylene-3-phenyl-4-((2,2,2-trifluoroethyl)thio)but-3-enoate (S-1cb)

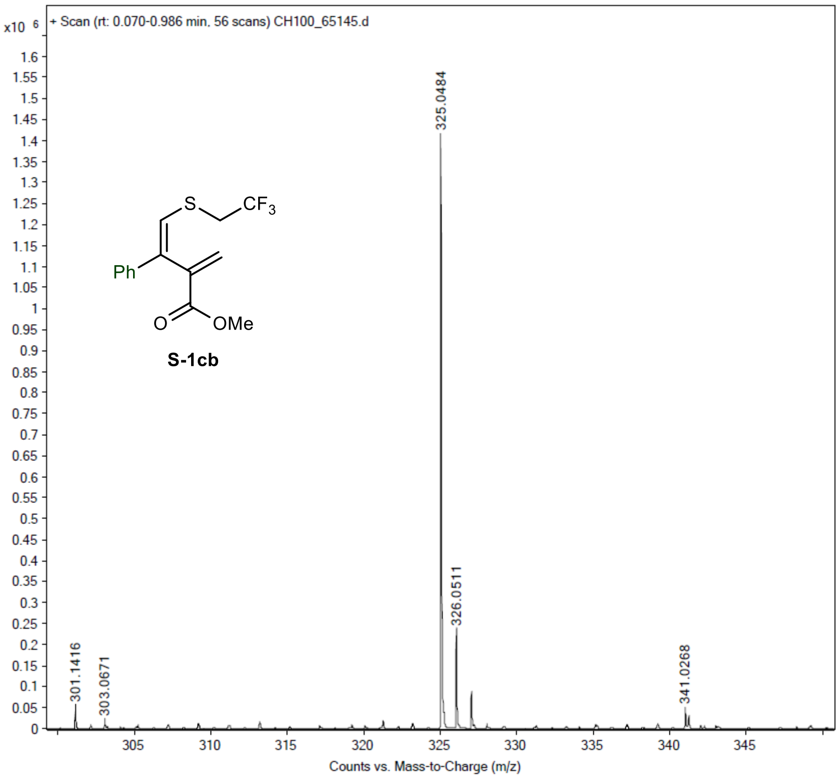

Methyl (*Z*)-4-((cyanomethyl)thio)-2-methylene-3-phenylbut-3-enoate (S-1cc)

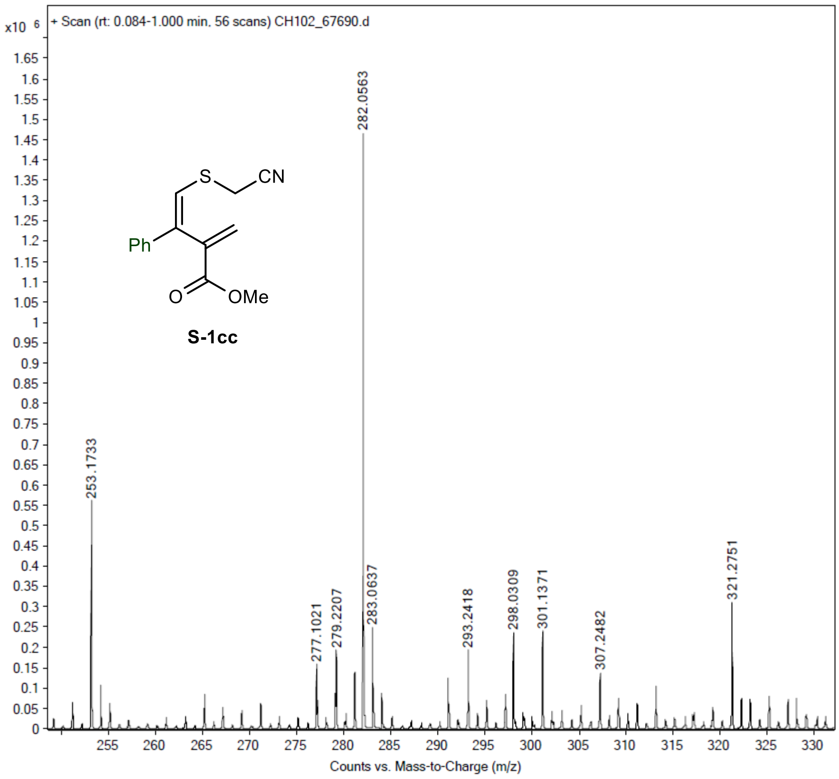

1-Ethyl 2-methyl 1-(methylthio)-2-(1-phenylvinyl)cyclopropane-1,2-dicarboxylate (3a)

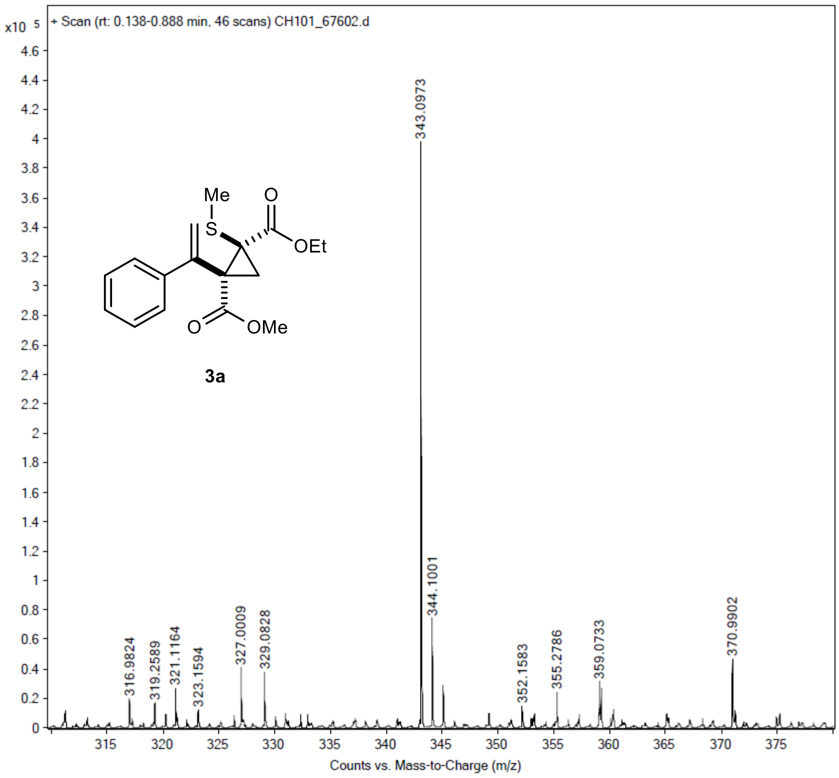

1-Ethyl 2-methyl 2-(1-(4-methoxyphenyl)vinyl)-1-(methylthio)cyclopropane-1,2-dicarboxylate (3b)

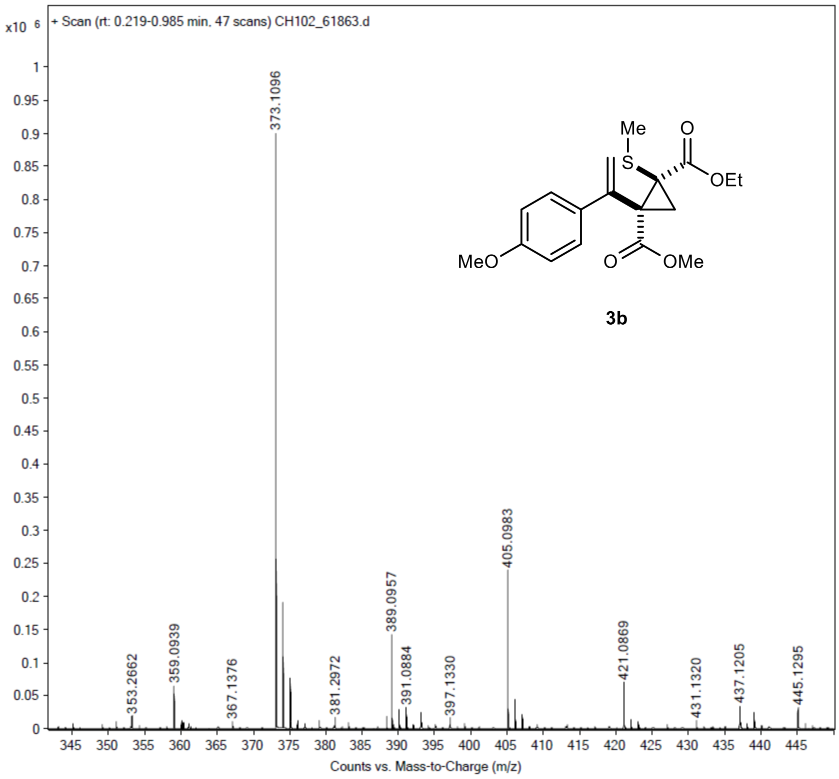

1-Ethyl 2-methyl 1-(methylthio)-2-(1-(4-(trifluoromethyl)phenyl)vinyl)cyclopropane-1,2-dicarboxylate (3c)

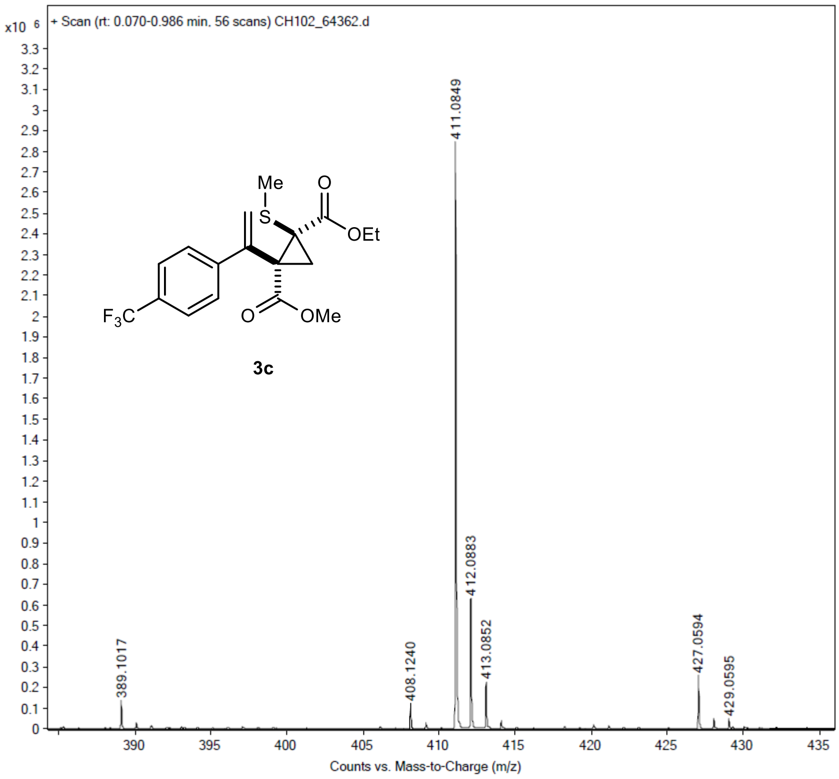

2-Ethyl 1-methyl 1-(1-(4-bromophenyl)vinyl)-2-(methylthio)cyclopropane-1,2-dicarboxylate (3d)

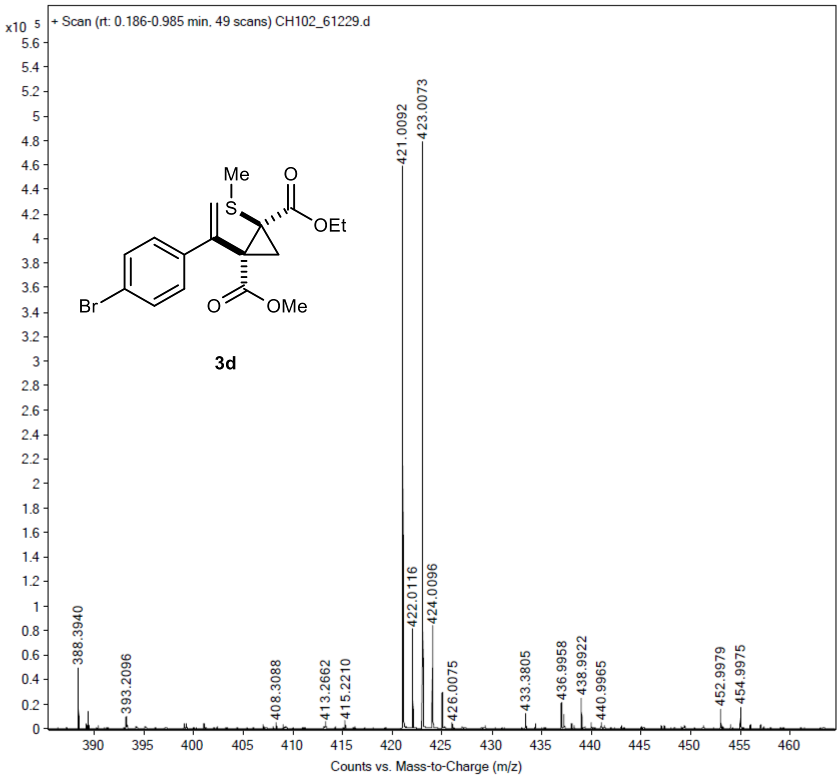

1-Ethyl 2-methyl 2-(1-(4-formylphenyl)vinyl)-1-(methylthio)cyclopropane-1,2-dicarboxylate (3e)

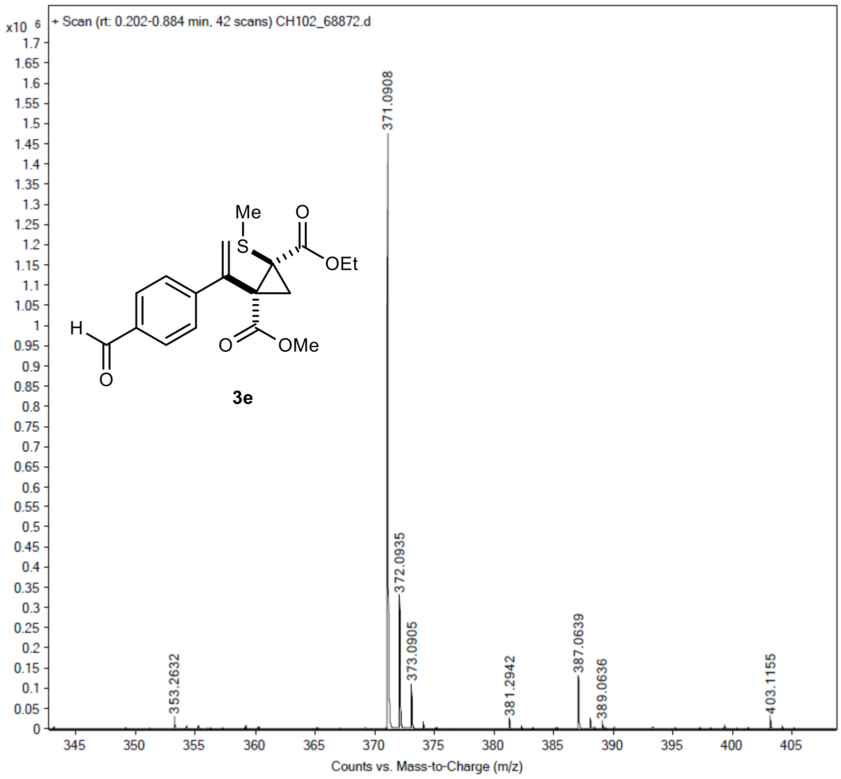

2-Ethyl 1-methyl 1-(1-(4-(ethoxycarbonyl)phenyl)vinyl)-2-(methylthio)cyclopropane-1,2-dicarboxylate (3f)

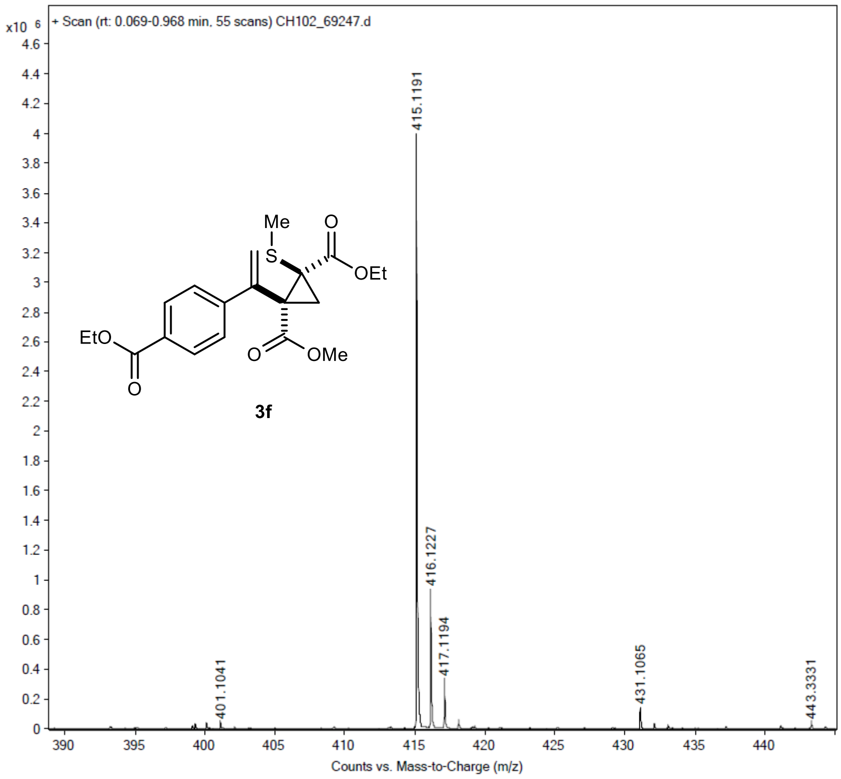

2-Ethyl 1-methyl 1-(1-(4-(dimethylcarbamoyl)phenyl)vinyl)-2-(methylthio)cyclopropane-1,2-dicarboxylate (3g)

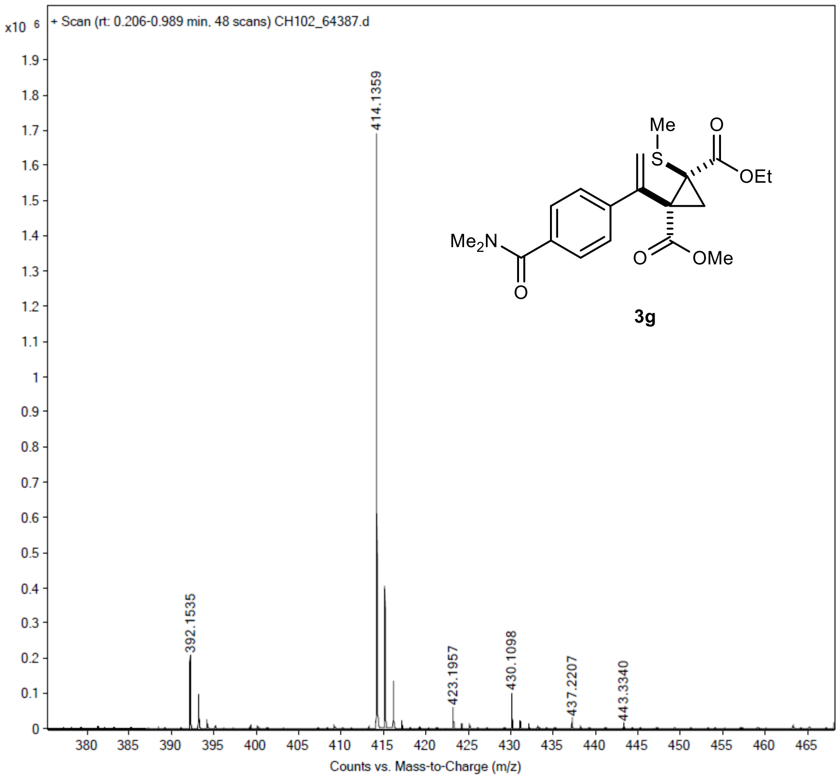

2-Ethyl 1-methyl 1-(1-(2-chlorophenyl)vinyl)-2-(methylthio)cyclopropane-1,2-dicarboxylate (3h)

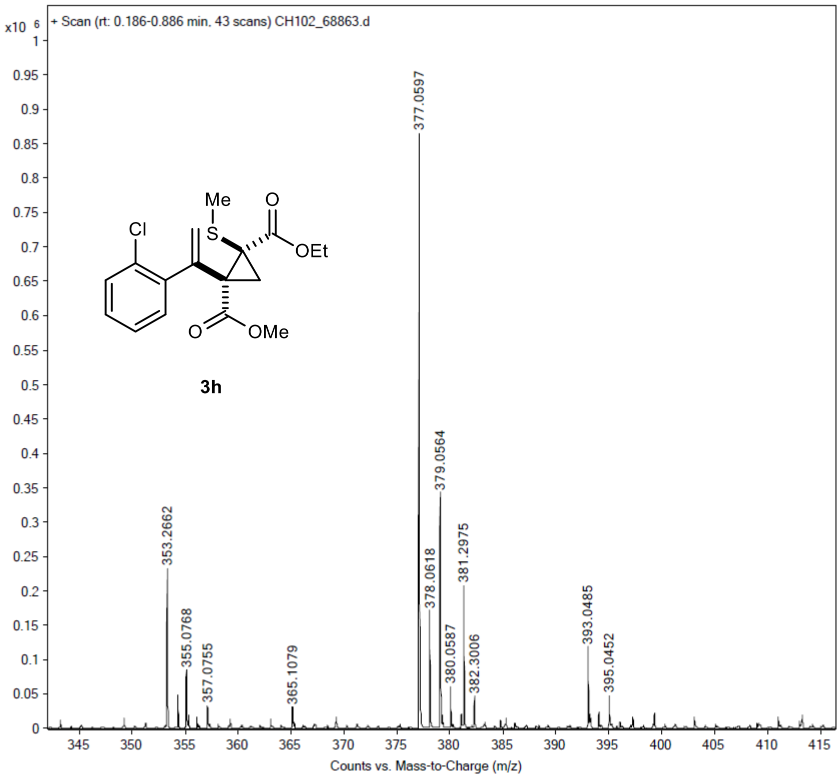

1-Ethyl 2-methyl 1-(methylthio)-2-(1-(3-nitrophenyl)vinyl)cyclopropane-1,2-dicarboxylate (3i)

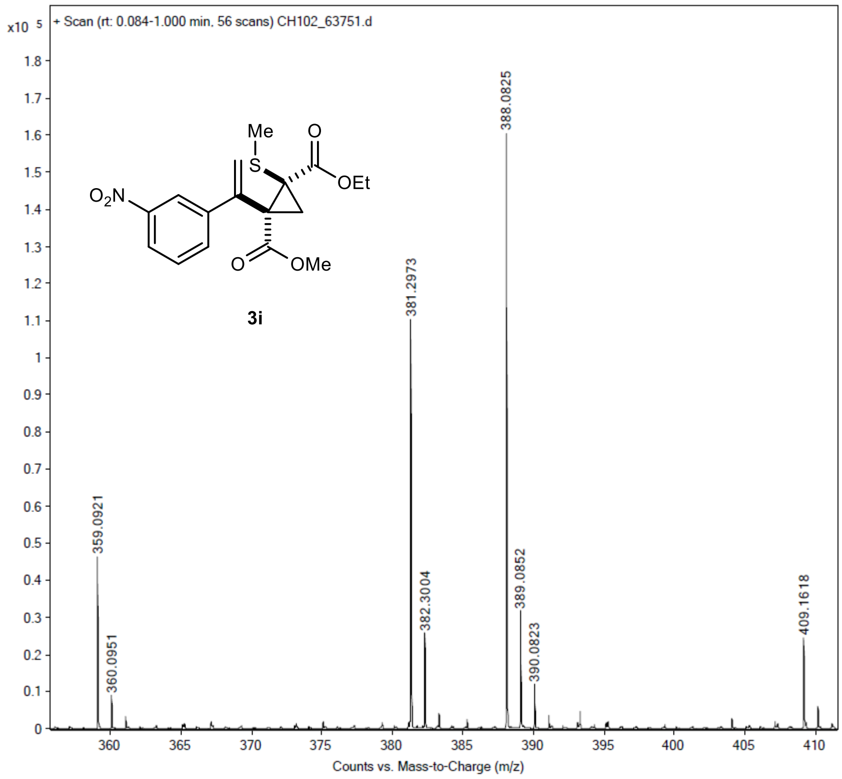

1-Ethyl 2-methyl 1-(methylthio)-2-(1-(thiophen-2-yl)vinyl)cyclopropane-1,2-dicarboxylate (3j)

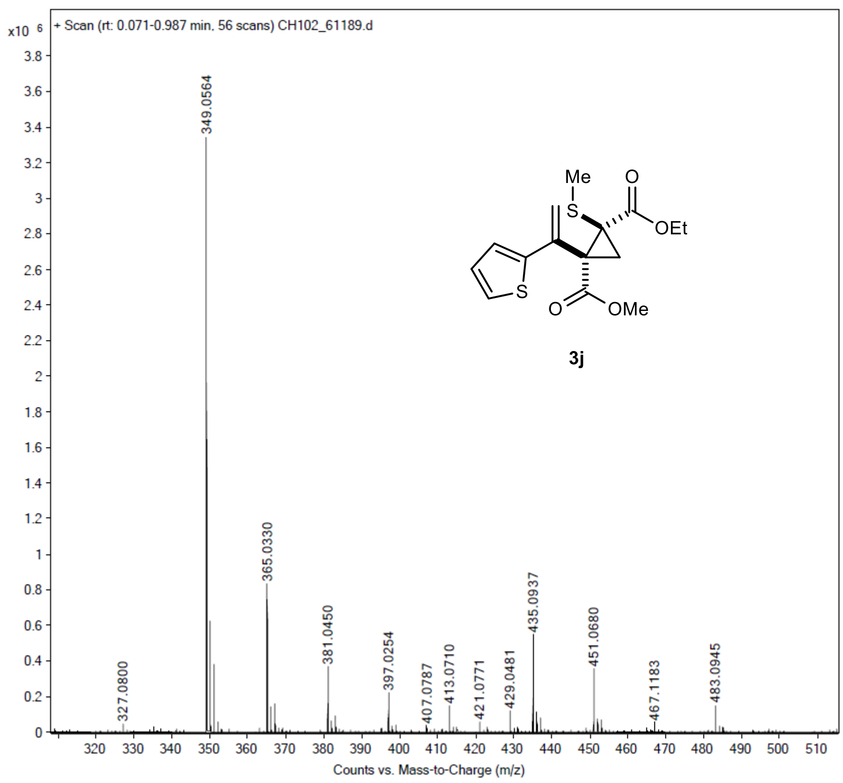

2-Ethyl 1-methyl 1-(1-(1-(*tert*-butoxycarbonyl)-1H-indol-5-yl)vinyl)-2-(methylthio)cyclopropane-1,2-dicarboxylate (3k)

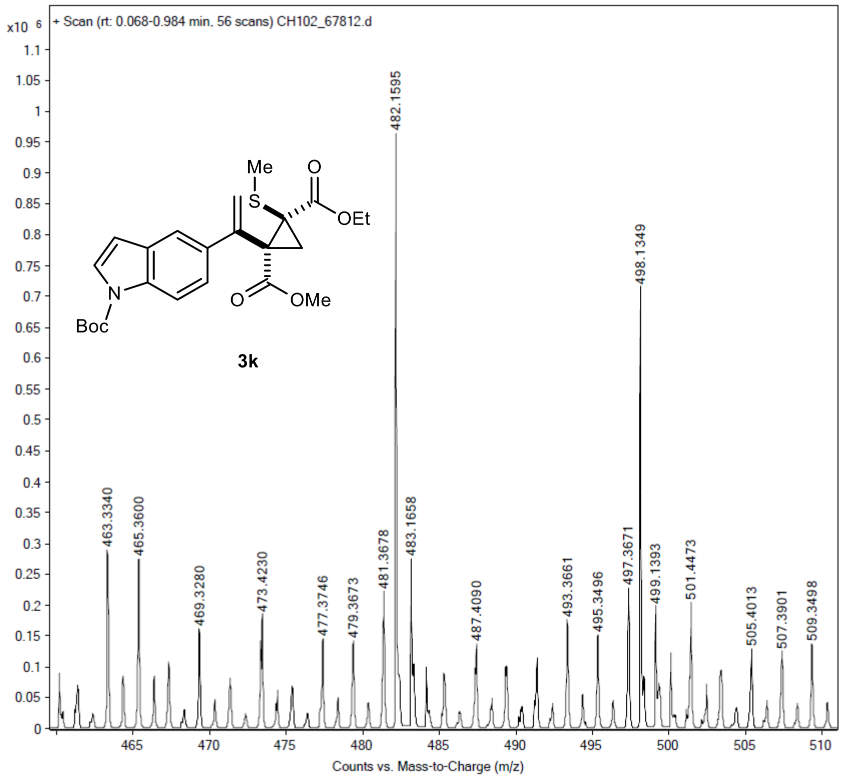

1-Ethyl 2-methyl 1-(methylthio)-2-(prop-1-en-2-yl)cyclopropane-1,2-dicarboxylate (3l)

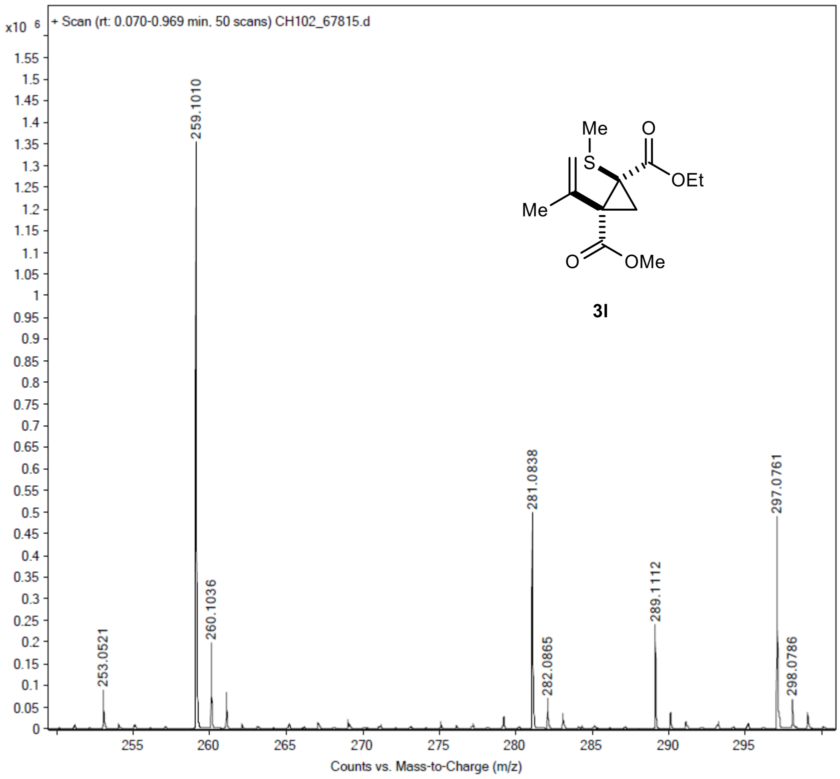

1-Ethyl 2-methyl 1-(methylthio)-2-(1-((triisopropylsilyl)oxy)vinyl)cyclopropane-1,2-dicarboxylate (3m)

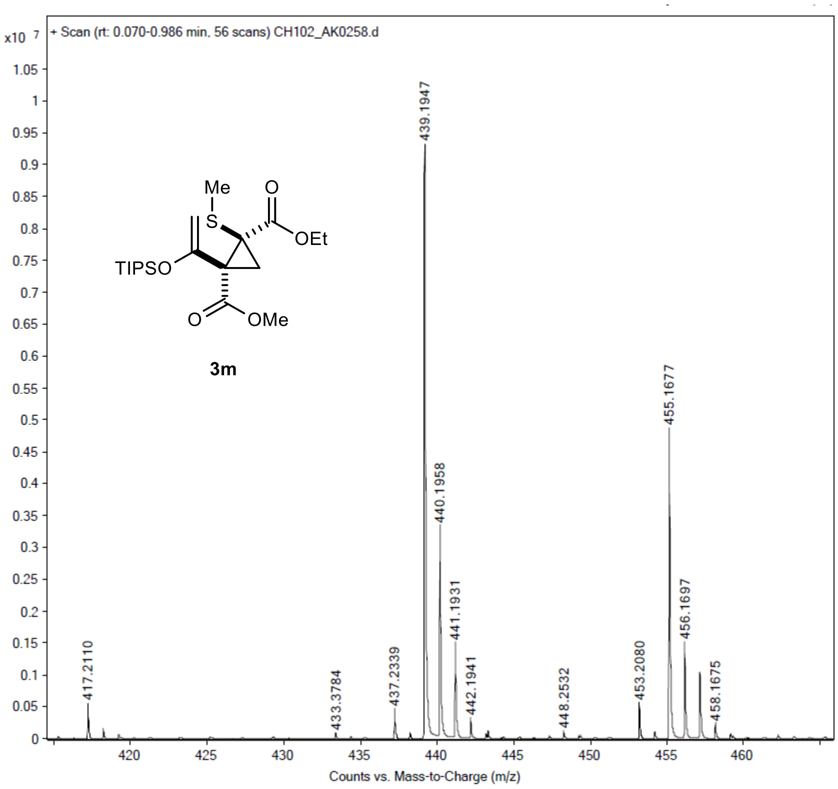

2-Ethyl 1-methyl 1-acetyl-2-(methylthio)cyclopropane-1,2-dicarboxylate (3n)

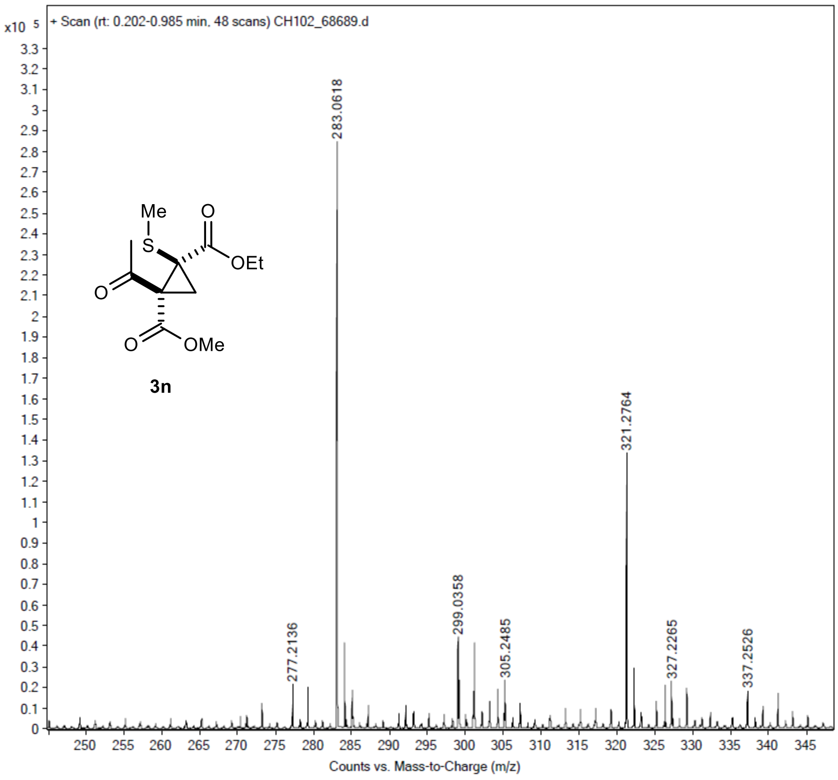

1-Ethyl 2-methyl 1-(methylthio)-2-((*E*)-1-phenylprop-1-en-1-yl)cyclopropane-1,2-dicarboxylate (3o)

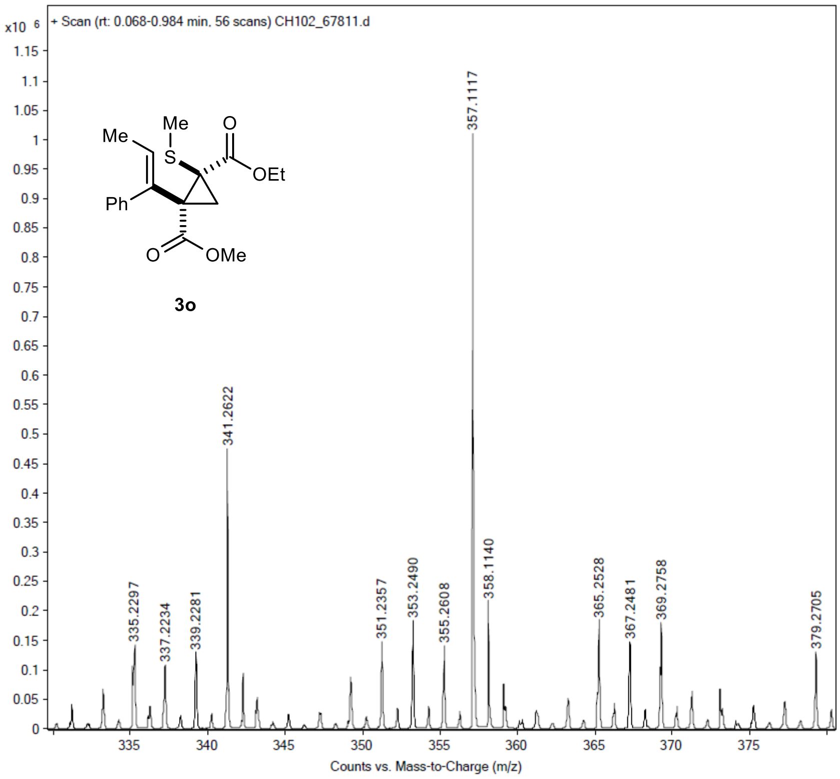

Ethyl 2-acetyl-1-(methylthio)-2-(1-phenylvinyl)cyclopropane-1-carboxylate (4a)

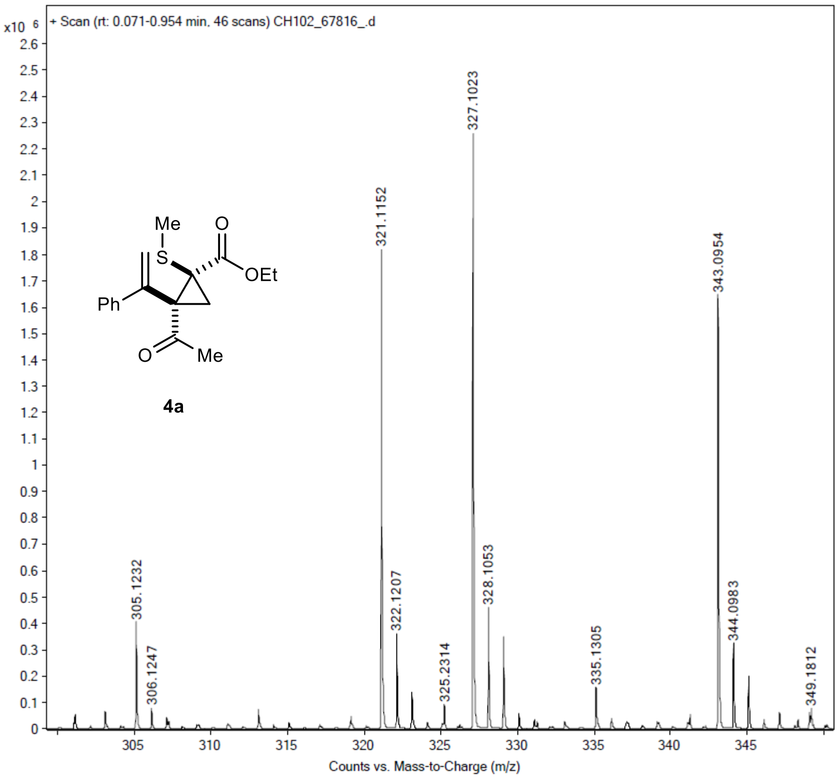

Ethyl 2-(methoxy(methyl)carbamoyl)-1-(methylthio)-2-(1-phenylvinyl)cyclopropane-1-carboxylate (4b)

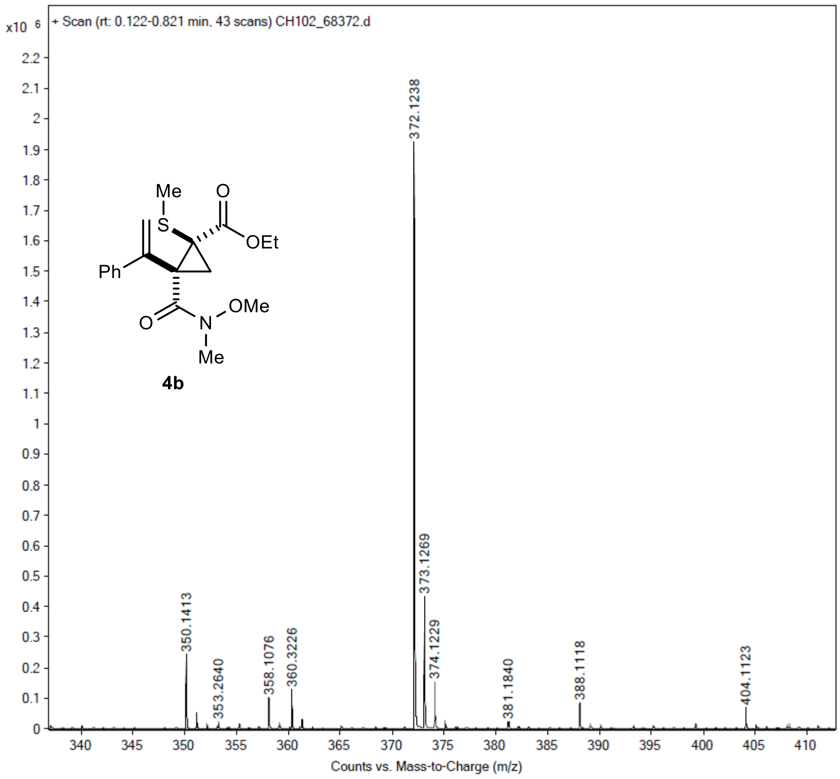

1-(Methylthio)-5-phenyl-3-azabicyclo[3.1.0]hexane-2,4-dione (4d)

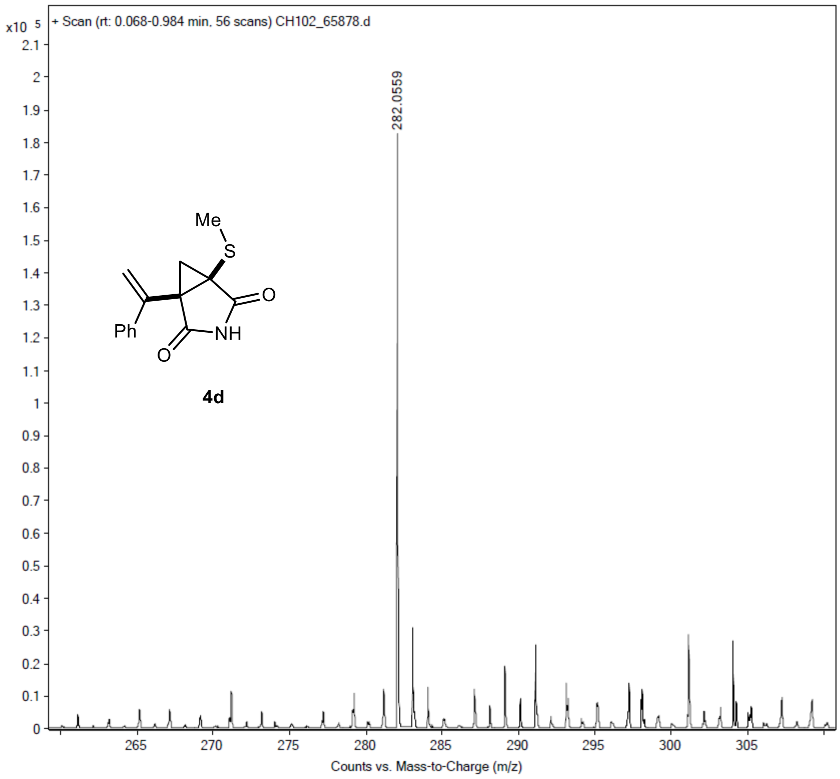

3-Methyl-1-(methylthio)-5-phenyl-3-azabicyclo[3.1.0]hexane-2,4-dione (4e)

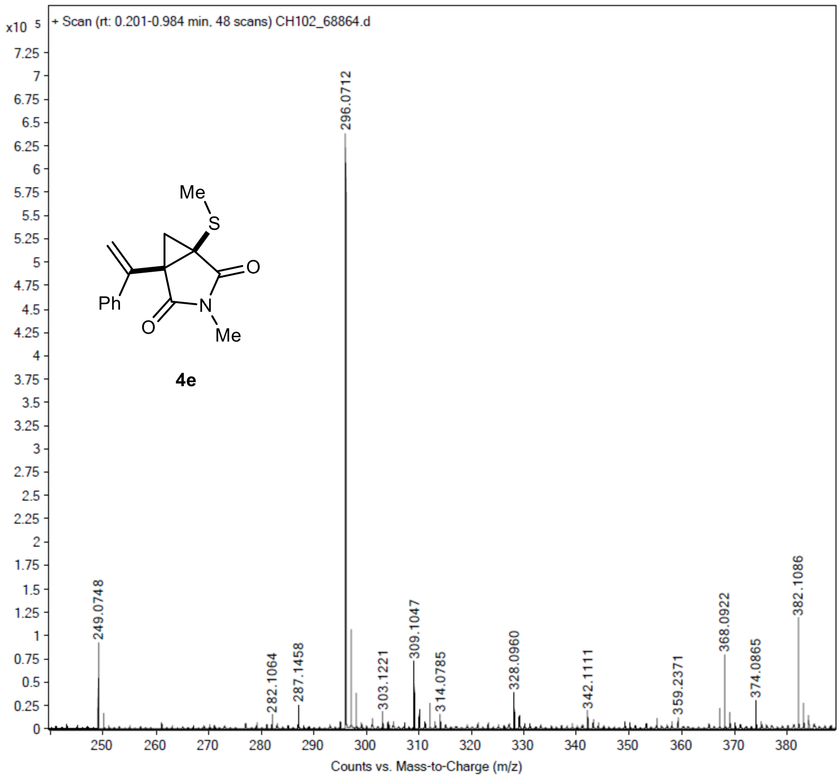

1-(Methylthio)-5-phenyl-3-oxabicyclo[3.1.0]hexan-2-one (4g)

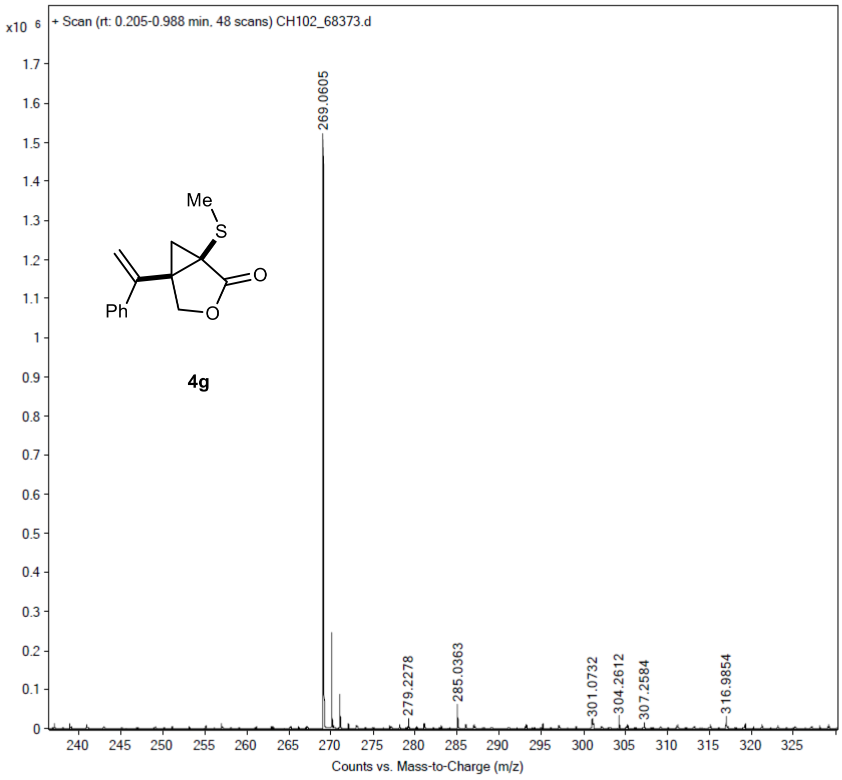

Ethyl 2-(methoxymethyl)-1-(methylthio)-2-(1-phenylvinyl)cyclopropane-1-carboxylate (4h)

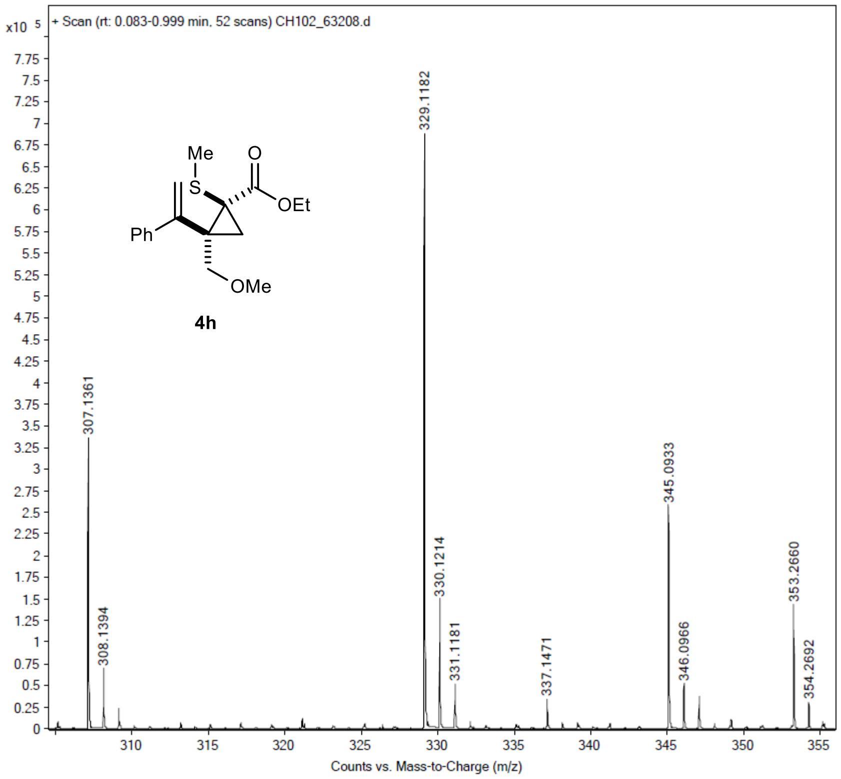

Ethyl 7-(methylthio)-2-oxo-1-vinylbicyclo[4.1.0]heptane-7-carboxylate (4i)

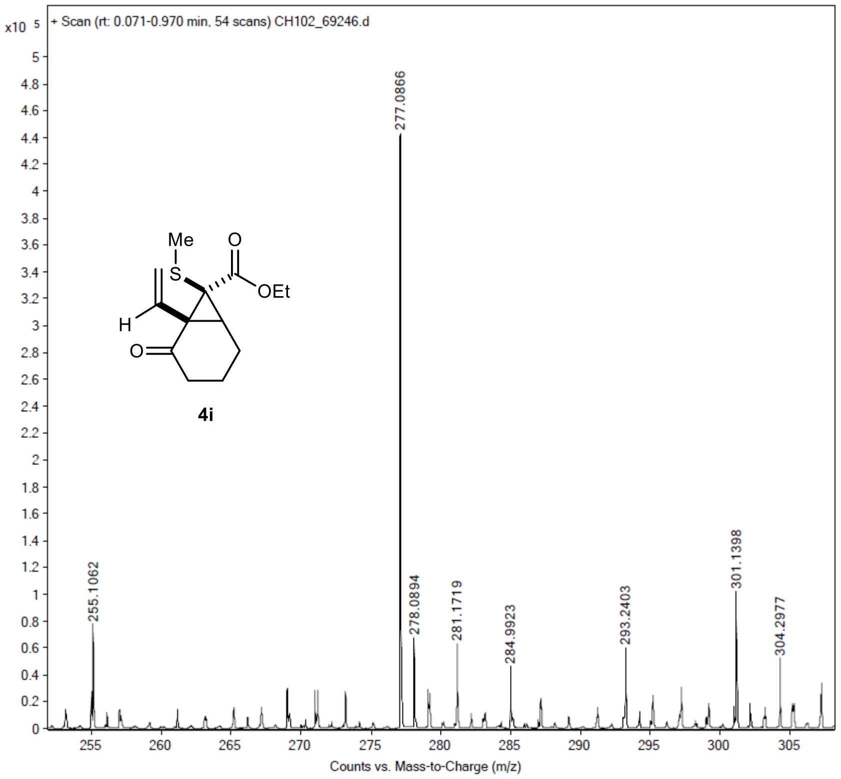

Ethyl 7-(methylthio)-2-oxo-1-(prop-1-en-2-yl)bicyclo[4.1.0]heptane-7-carboxylate (4j)

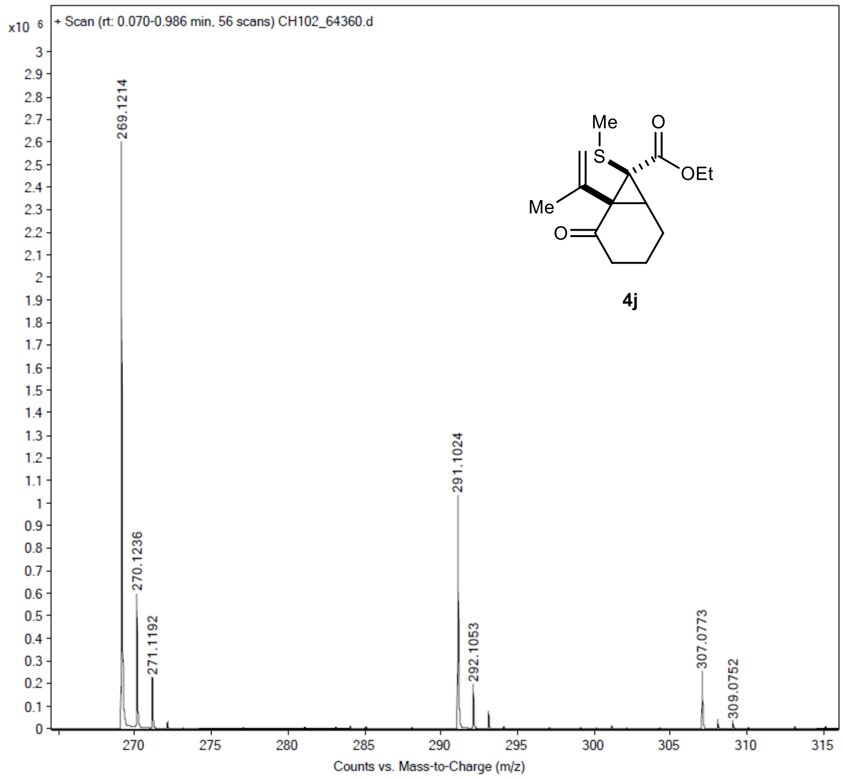

1-(*Tert*-butyl) 2-methyl 1-(methylthio)-2-(1-phenylvinyl)cyclopropane-1,2-dicarboxylate (5a)

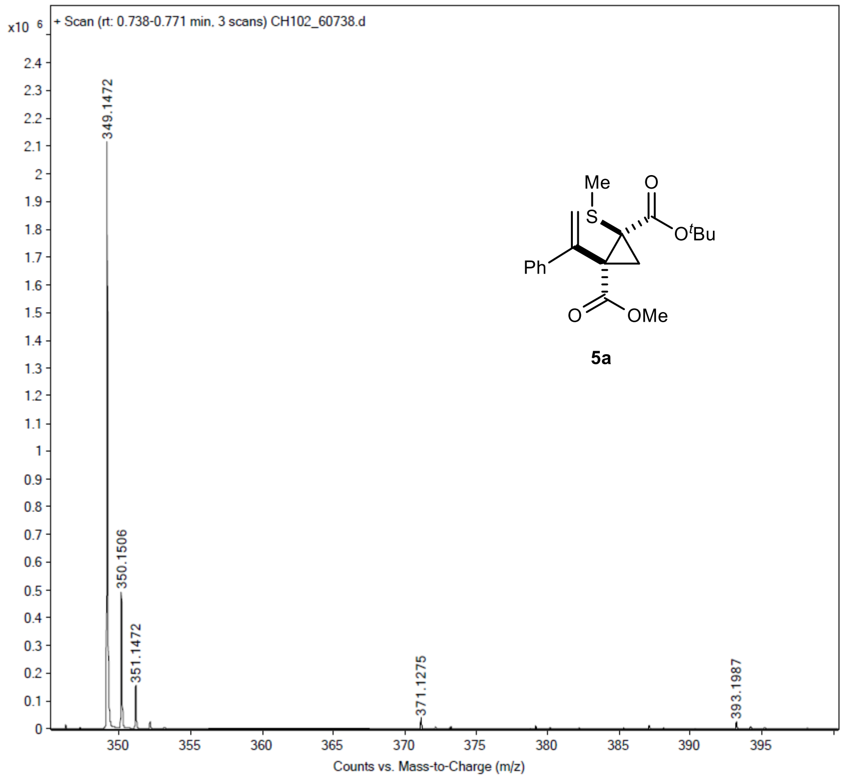

1-Benzyl 2-methyl 1-(methylthio)-2-(1-phenylvinyl)cyclopropane-1,2-dicarboxylate (5b)

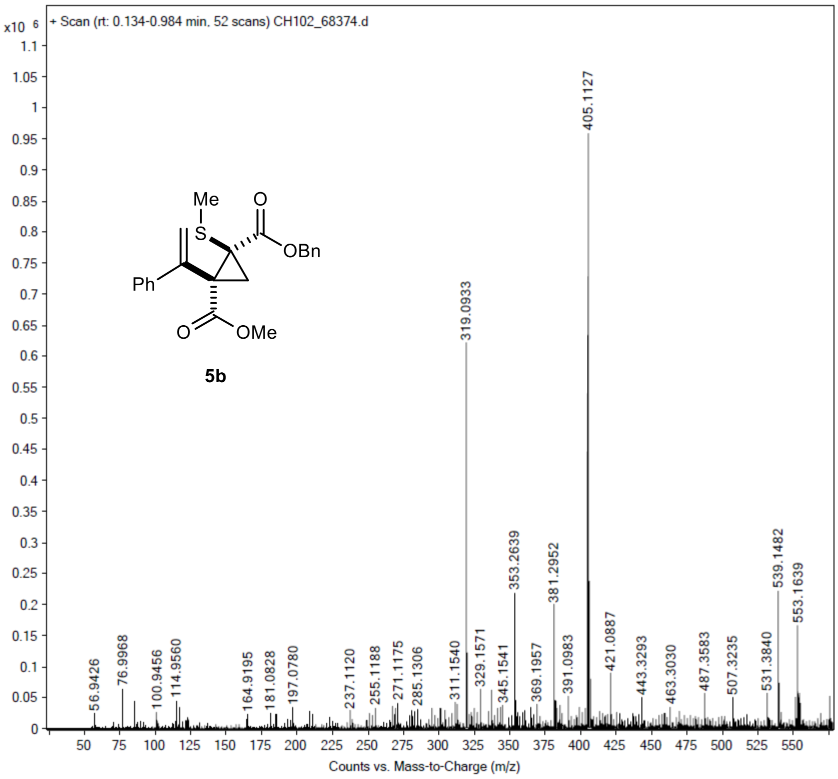

Methyl 2-acetyl-2-(methylthio)-1-(1-phenylvinyl)cyclopropane-1-carboxylate (5c)

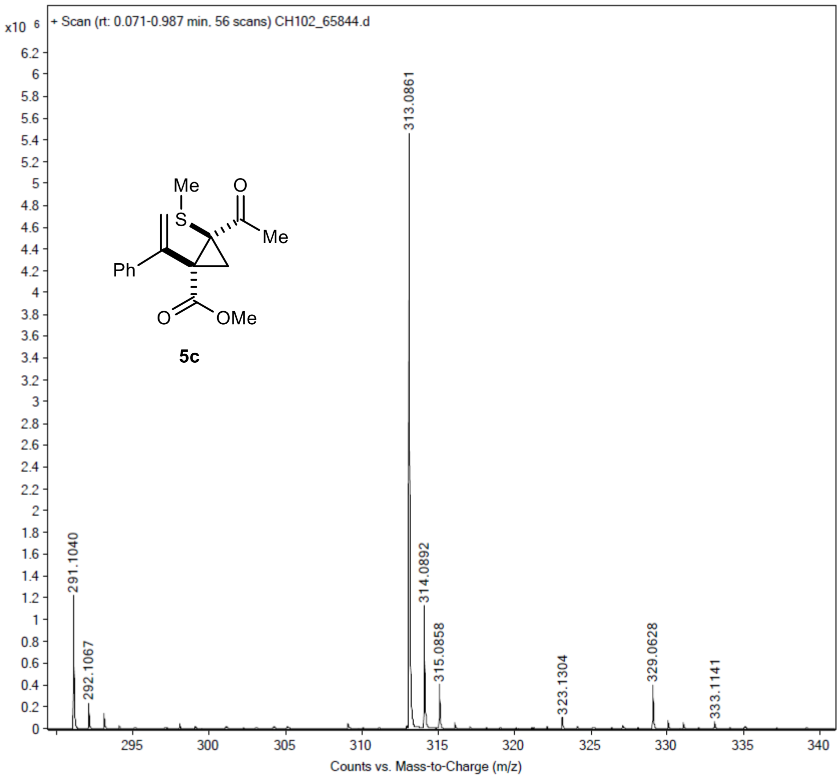

Methyl 2-benzoyl-2-(methylthio)-1-(1-phenylvinyl)cyclopropane-1-carboxylate (5d)

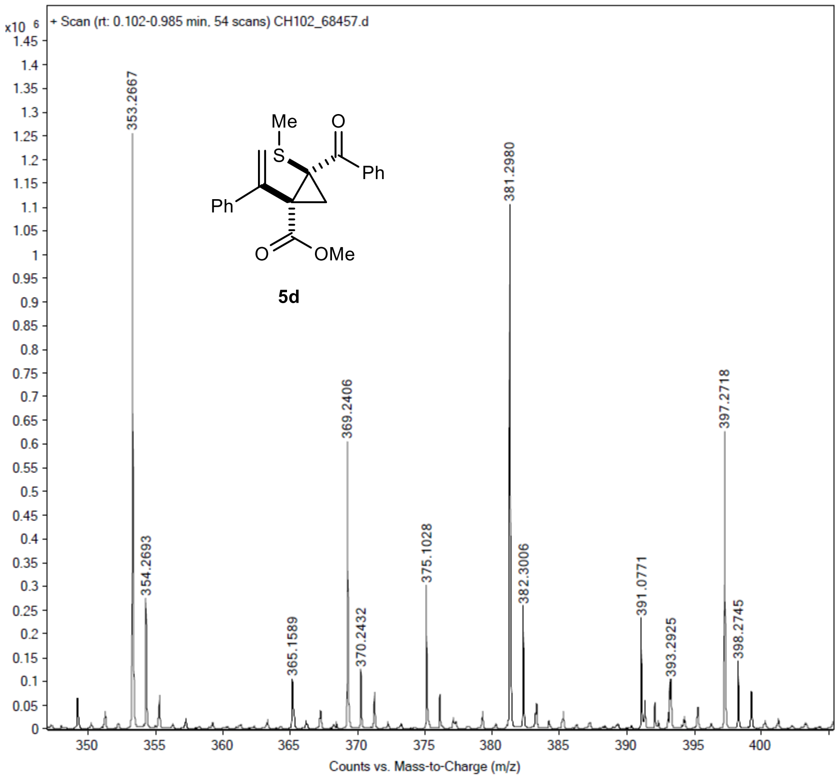

Methyl 2-(methylthio)-1-(1-phenylvinyl)cyclopropane-1-carboxylate (5e)

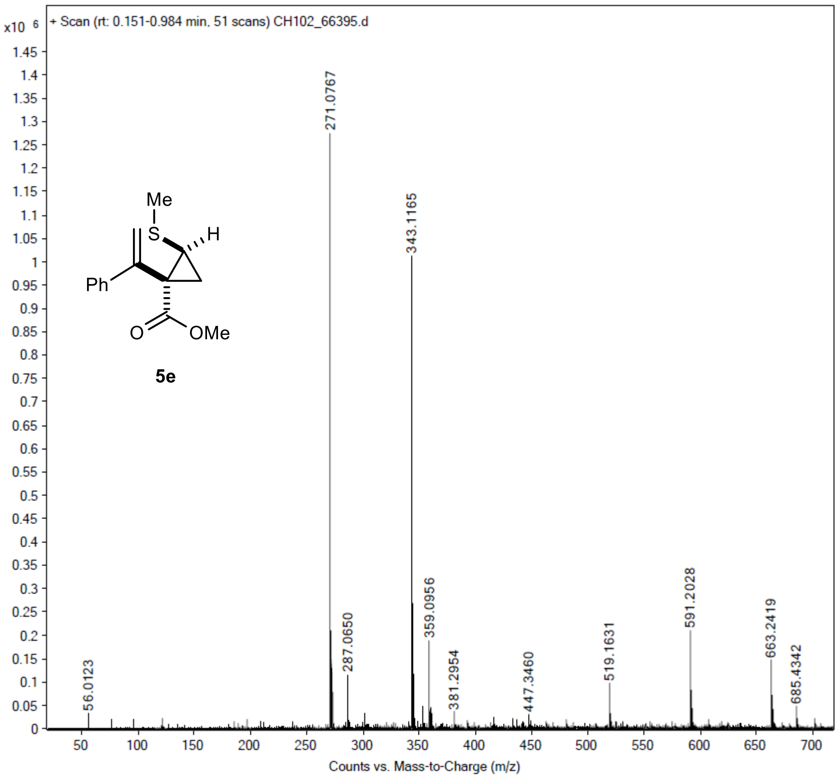

1-Ethyl 2-methyl 1-(ethylthio)-2-(1-phenylvinyl)cyclopropane-1,2-dicarboxylate (6a)

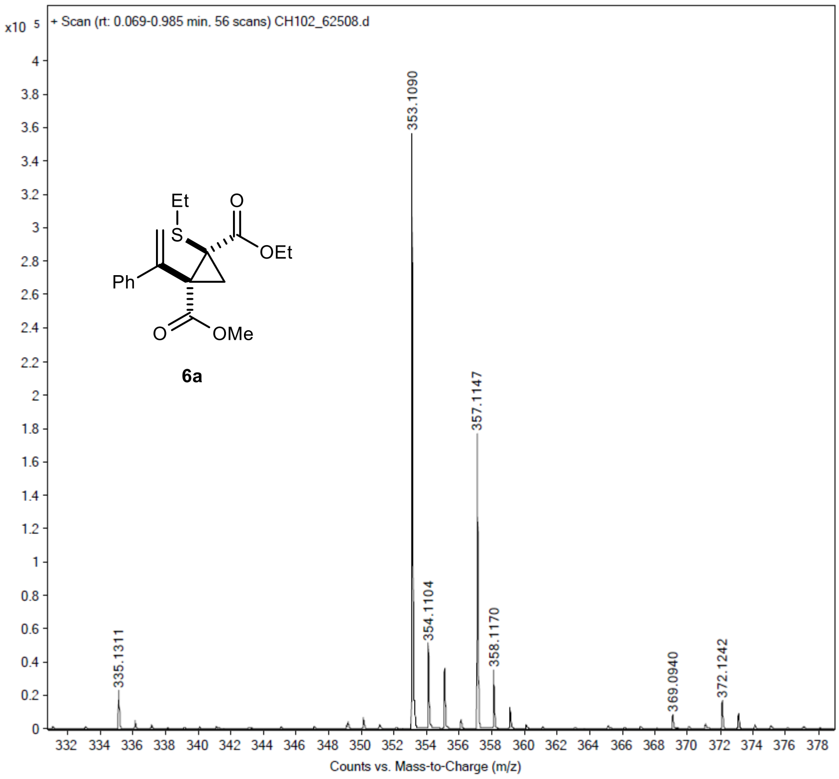

1-Ethyl 2-methyl 1-((2-ethoxy-2-oxoethyl)thio)-2-(1-phenylvinyl)cyclopropane-1,2-dicarboxylate (6b)

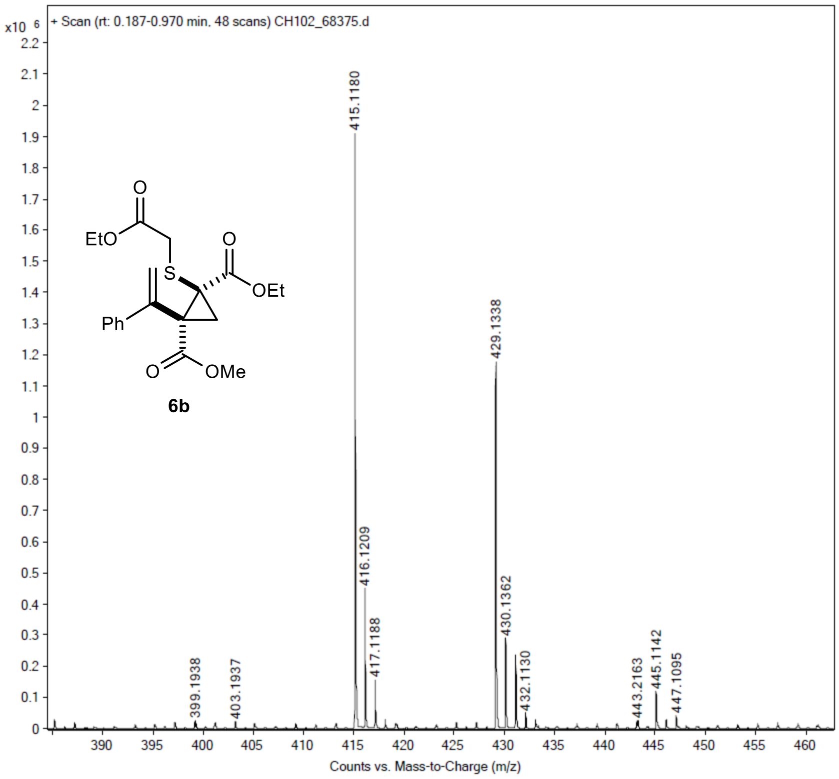

1-Ethyl 2-methyl 1-(benzylthio)-2-(1-phenylvinyl)cyclopropane-1,2-dicarboxylate (6c)

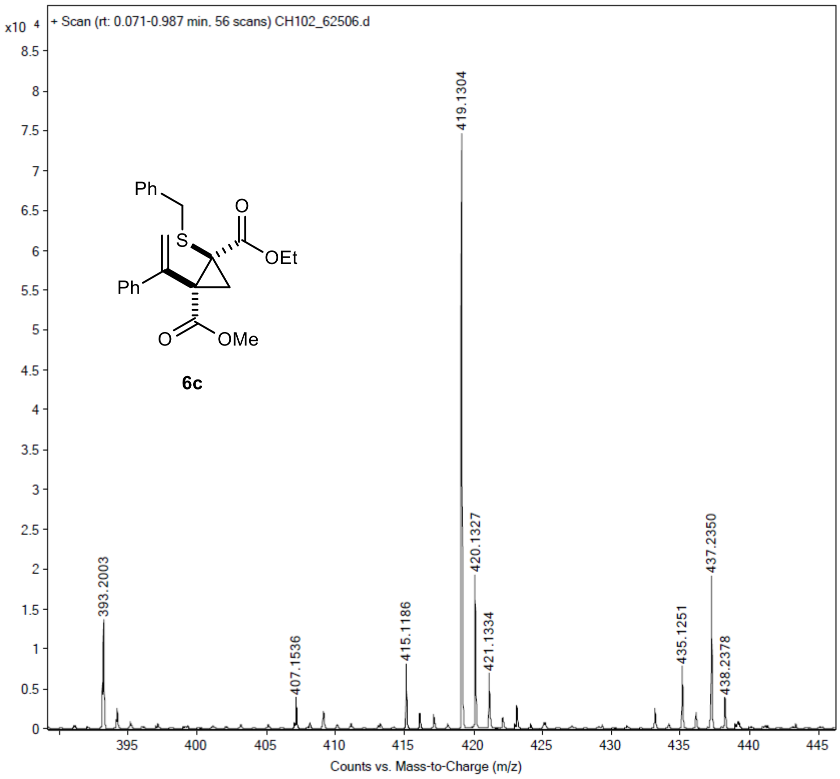

1-Ethyl 2-methyl 1-(phenylthio)-2-(1-phenylvinyl)cyclopropane-1,2-dicarboxylate (6d)

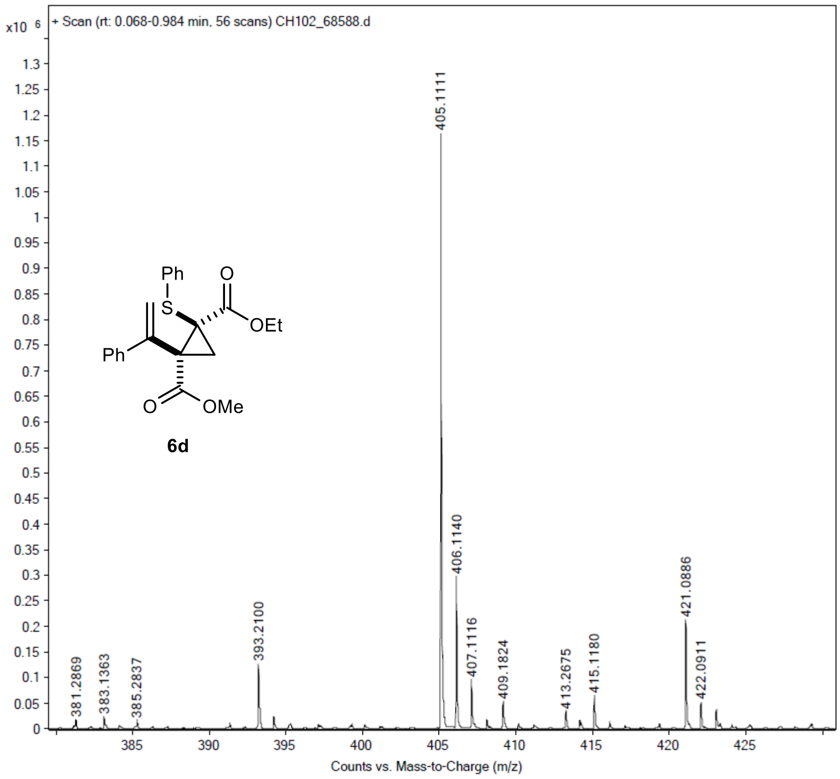

Ethyl (Z)-2-((3-(methoxycarbonyl)-2-phenylbuta-1,3-dien-1-yl)thio)pent-4-enoate (7)

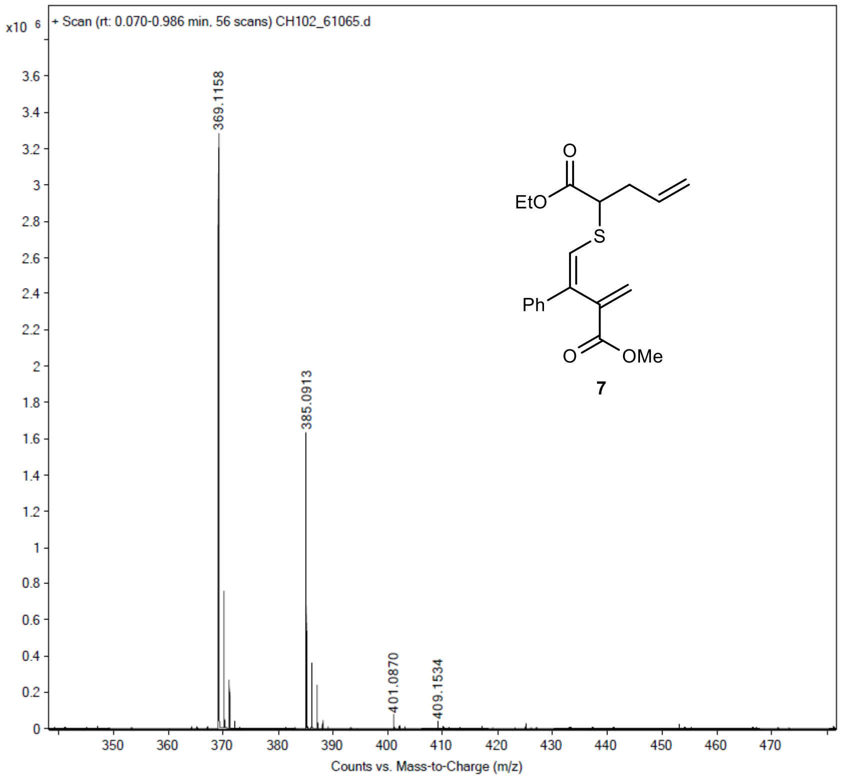

(*Z*)-(2-Ethoxy-2-oxoethyl)(3-(methoxycarbonyl)-2-phenylbuta-1,3-dien-1-yl)(methyl)sulfonium triflate salt (8)

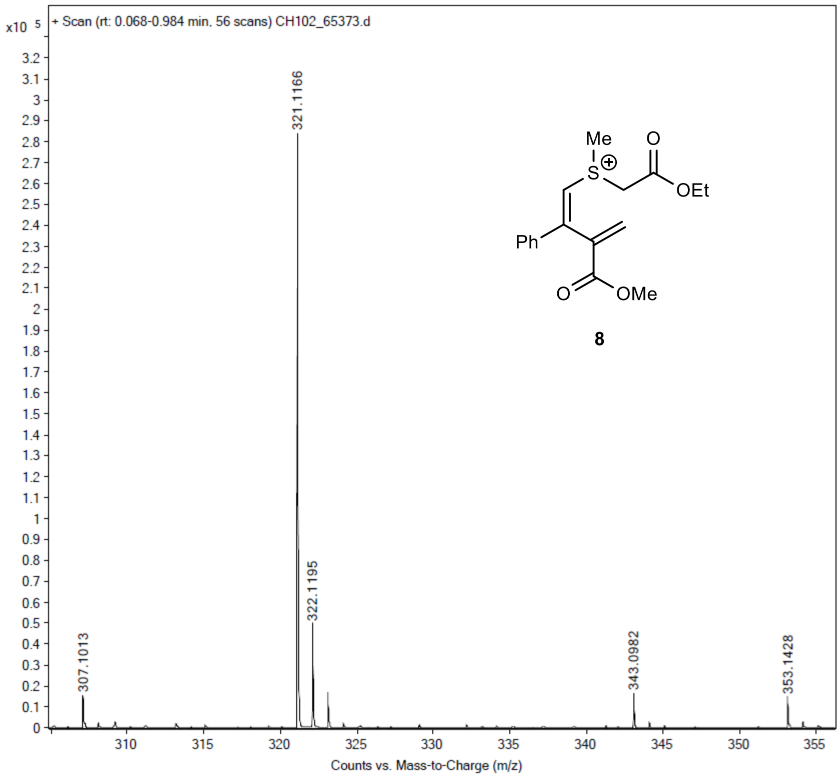

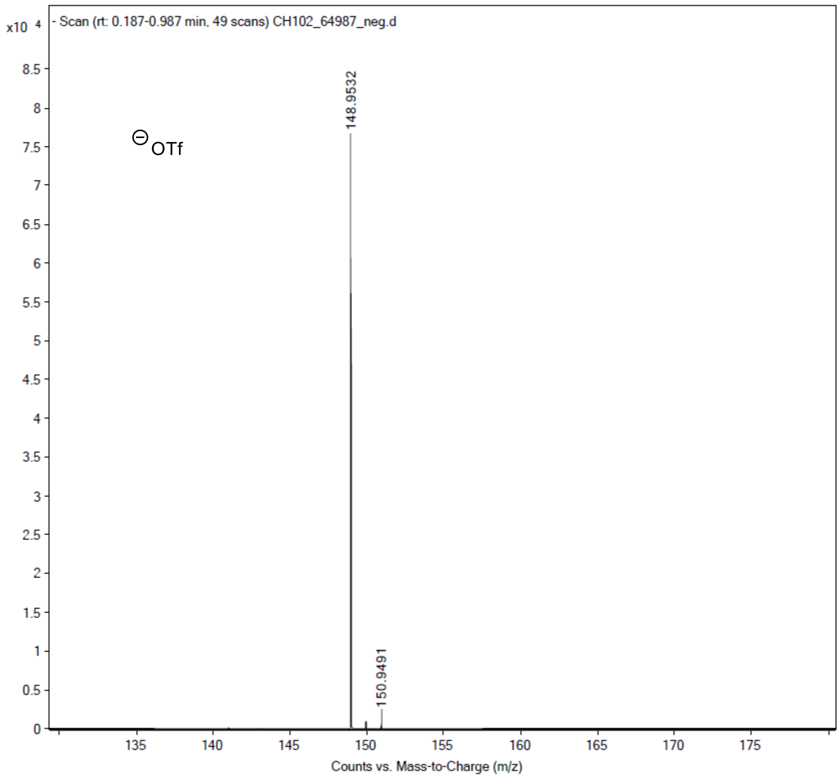

1-Ethyl 2-methyl 1-(methylthio)-2-(1-phenylvinyl-2-*d*)cyclopropane-1,2-dicarboxylate (*D*-3a)

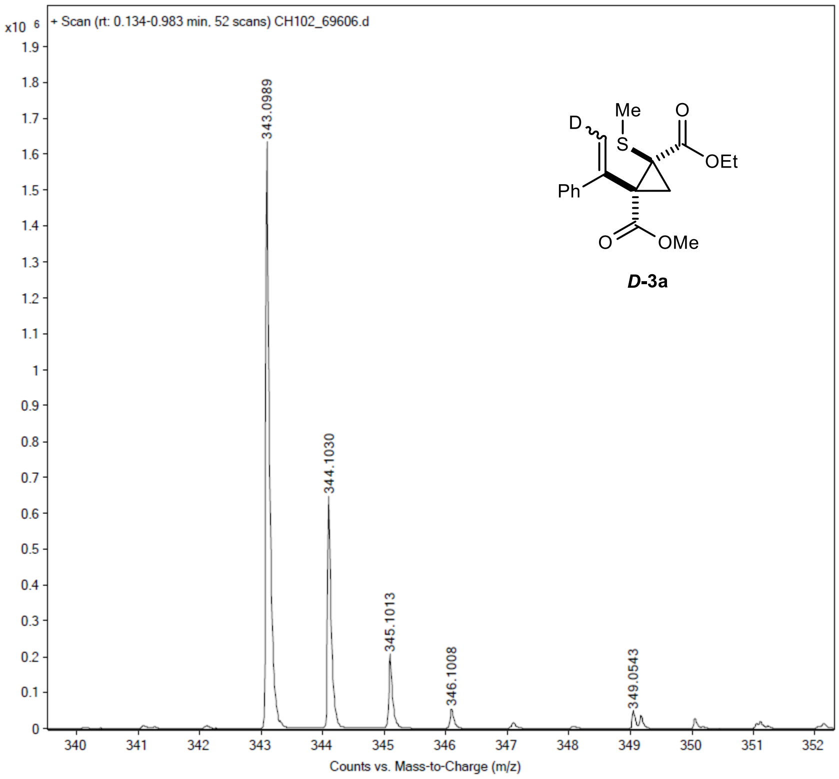

Methyl 2-benzoyl-5-phenyl-3,4-dihydro-2*H*-thiopyran-4-carboxylate (10c)

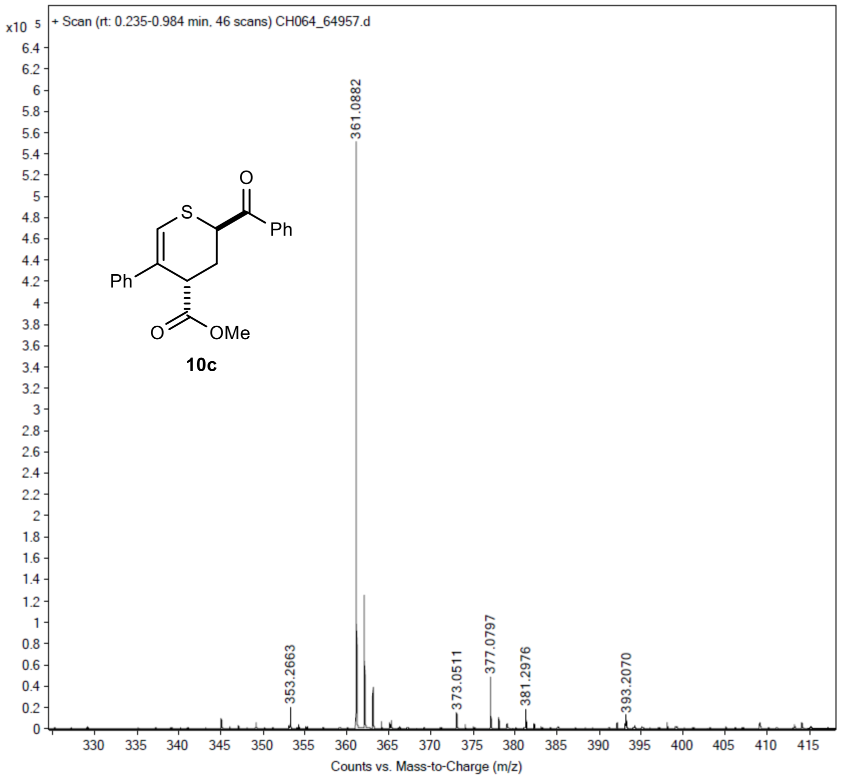

Methyl 4-phenyl-2,5-dihydrothiophene-3-carboxylate 1,1-dioxide (S-15a)

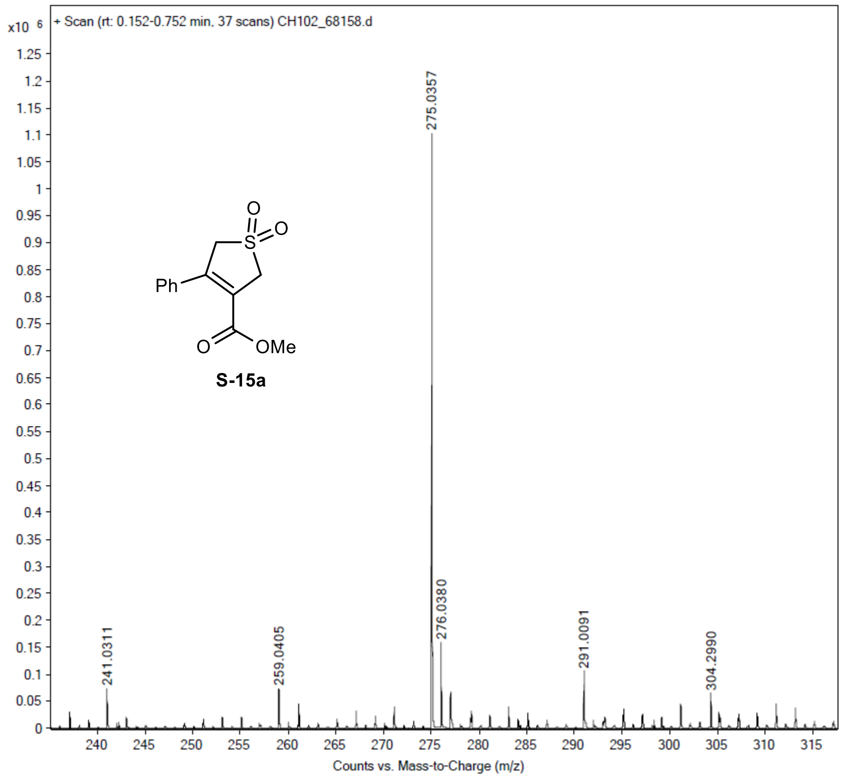

Methyl 2-methylene-3-phenylbut-3-enoate (S-15b)

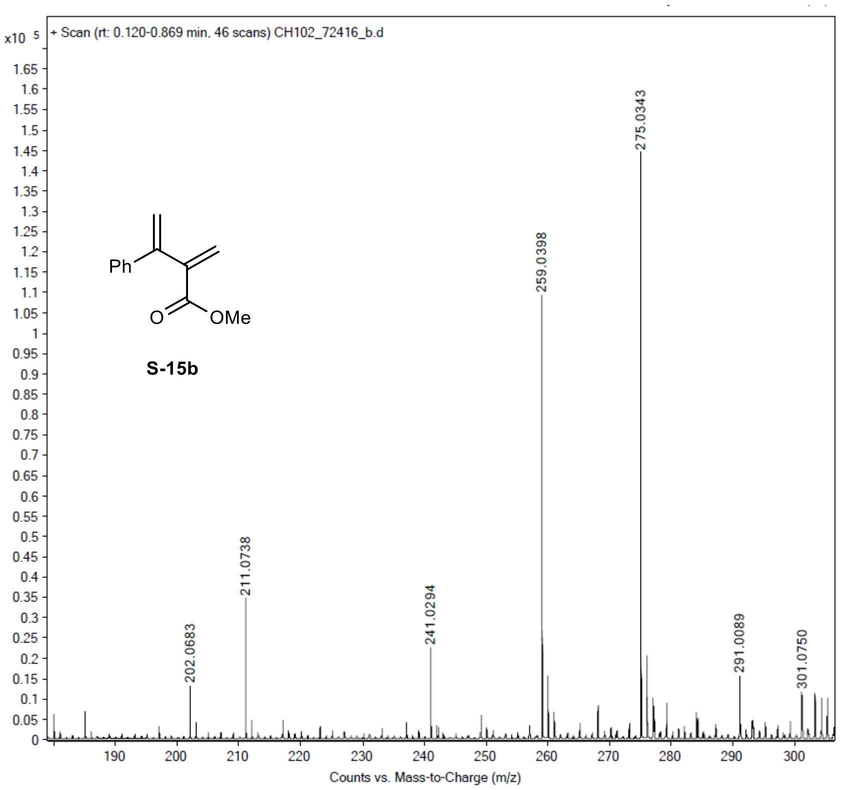

Methyl 2-(dimethoxyphosphoryl)-2-(methylthio)-1-(1-phenylvinyl)cyclopropane-1-carboxylate (11a)

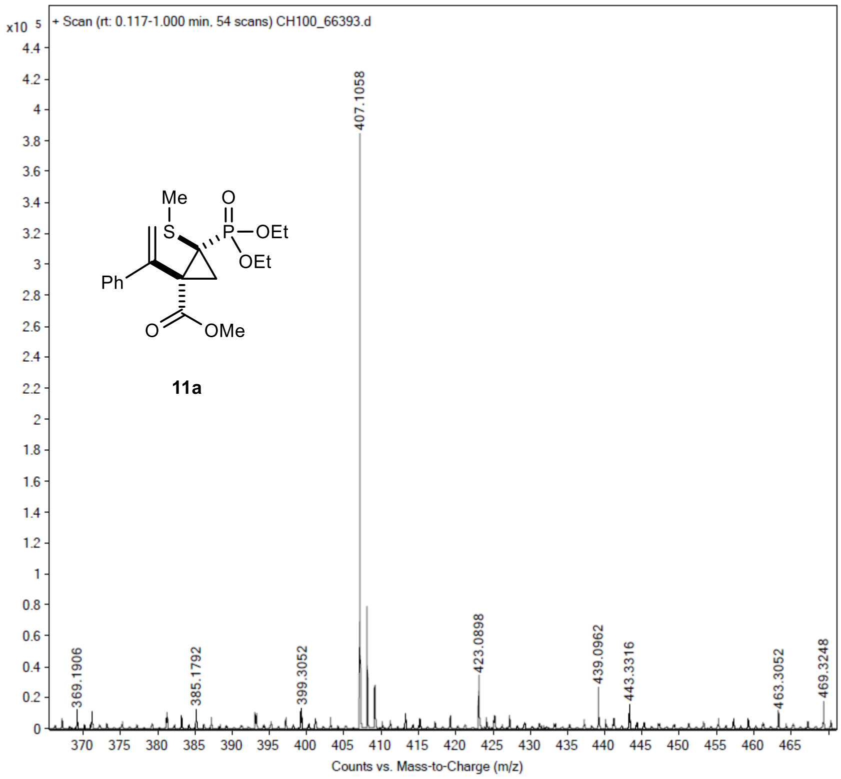

Methyl 2-(methylthio)-1-(1-phenylvinyl)-2-(trifluoromethyl)cyclopropane-1-carboxylate (11b)

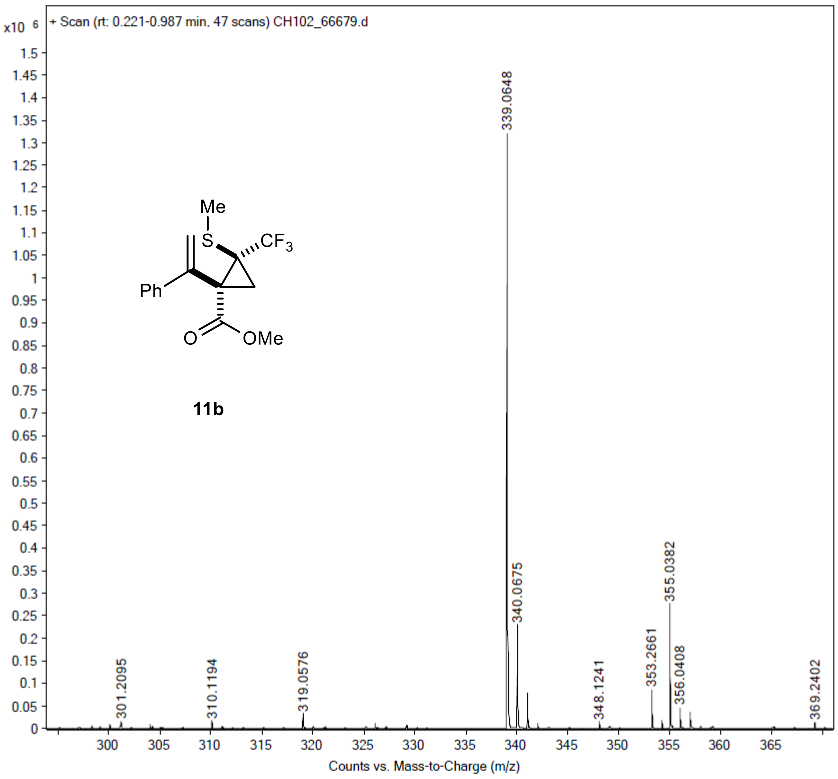

Methyl 2-cyano-2-(methylthio)-1-(1-phenylvinyl)cyclopropane-1-carboxylate (11c)

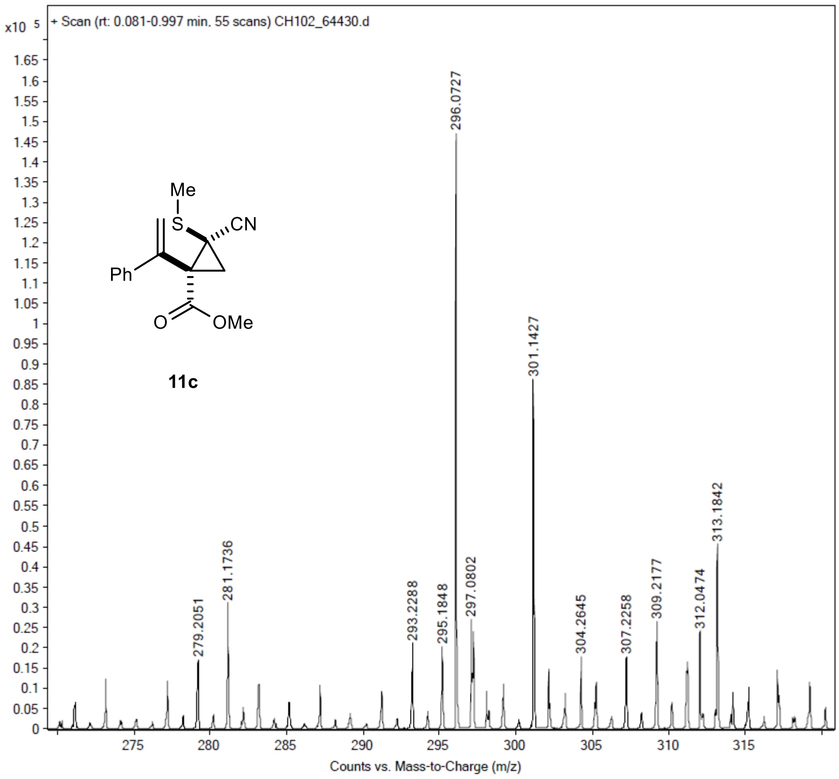

1-Ethyl 2-methyl 1-(methylsulfinyl)-2-(1-phenylvinyl)cyclopropane-1,2-dicarboxylate (12a)

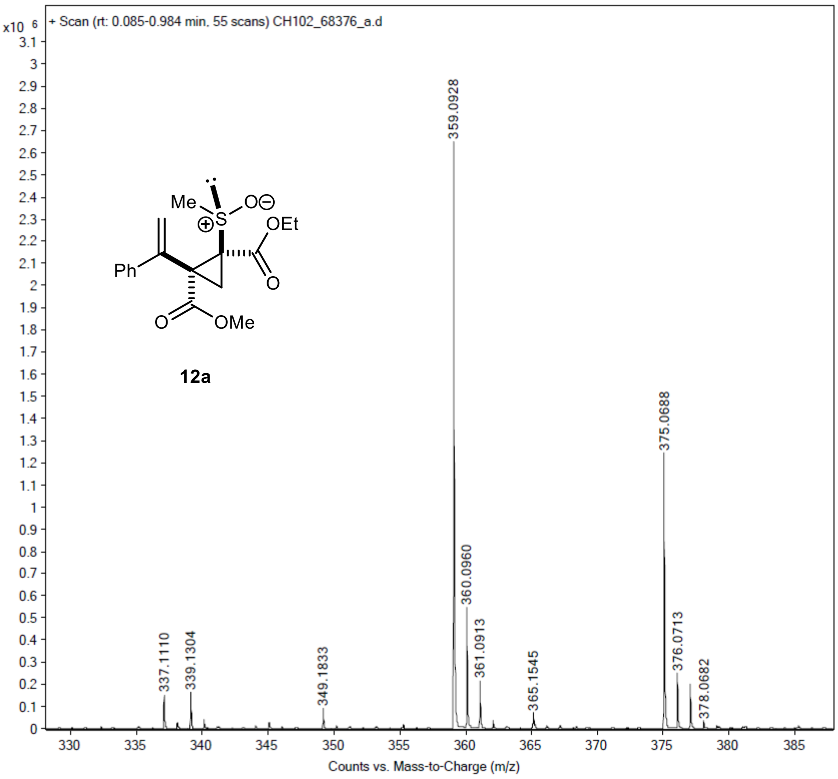

1-Ethyl 2-methyl 1-(methylsulfinyl)-2-(1-phenylvinyl)cyclopropane-1,2-dicarboxylate (12b)

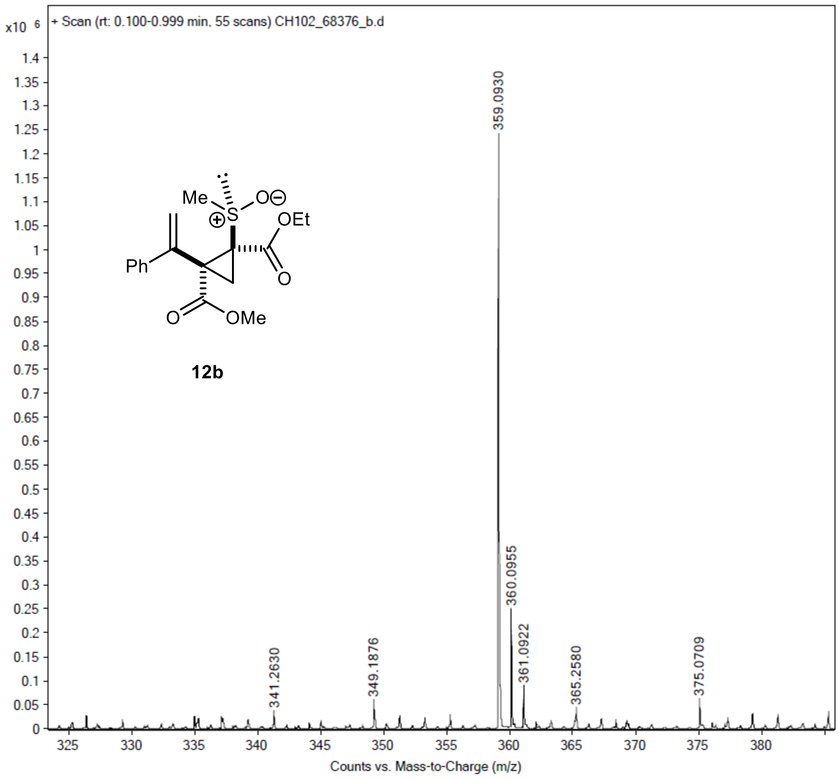

2-Ethyl 1-methyl 1-(1-phenylvinyl)cyclopropane-1,2-dicarboxylate (12c)

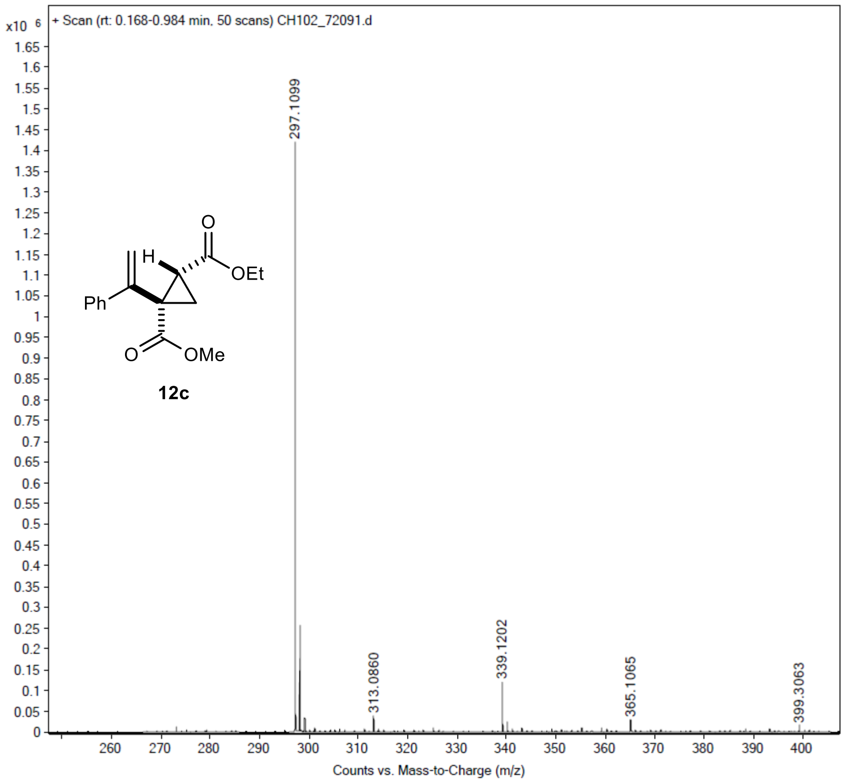

1-Ethyl 2-methyl 1-(methylsulfonyl)-2-(1-phenylvinyl)cyclopropane-1,2-dicarboxylate (12d)

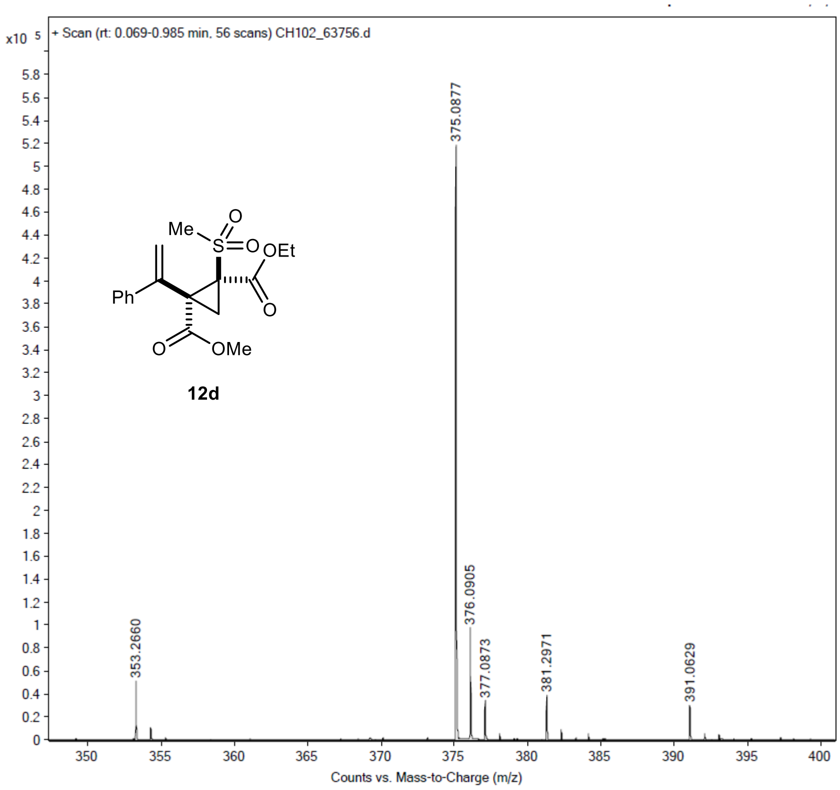

1-Ethyl 2-methyl 1-(*S*-methyl-*N*-tosylsulfinimidoyl)-2-(1-phenylvinyl)cyclopropane-1,2-dicarboxylate (12e)

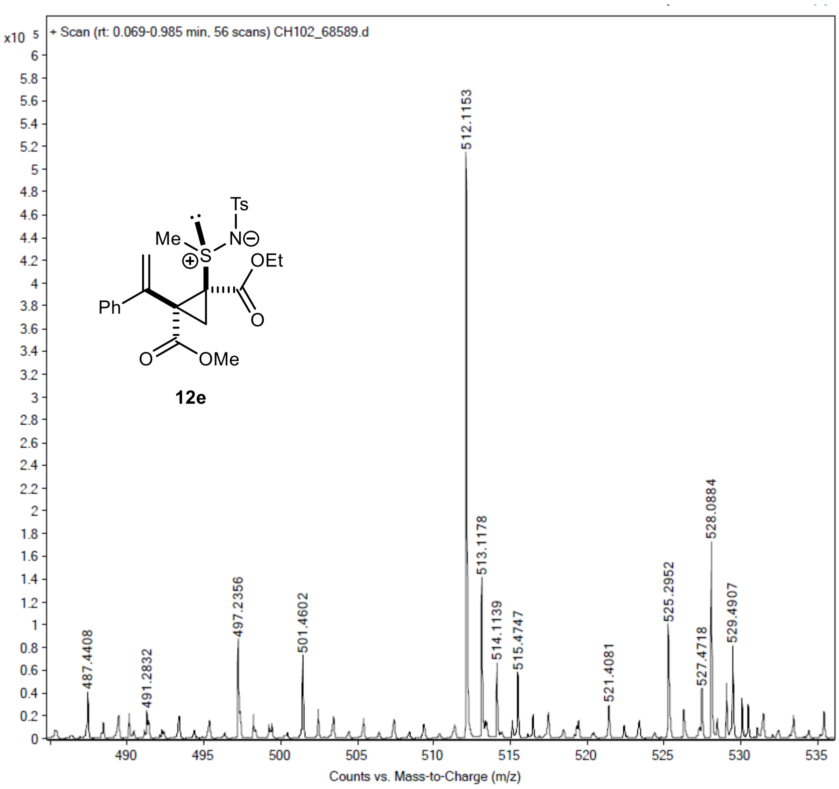

Supplement: Supplementary file 2 — Supporting File: anie72273‐sup‐0002‐SuppMat.Pdf. [file ANIE-65-e4468205-s001.pdf]
